# Genomic surveillance reveals co-occurrence of *Plasmodium falciparum* drug resistance variants across diverse transmission settings in Ethiopia

**DOI:** 10.1038/s41564-026-02420-5

**Published:** 2026-08-05

**Authors:** Alemayehu Letebo, Leen N. Vanheer, Mengst Engdaw, Legesse Alamerie Ejigu, Dawit Hailu Alemayehu, Jimma Dinsa Deressa, Bethlehem Adnew, Abaysew Ayele, Migbaru Keffale Bezabih, Mulugeta Demisse, Tamrayehu Seyoum, Meklit Shiferaw, Heven Sime, Atsbeha Gebreegziabxier Weldemariam, Amanuel Shimelash, Elias B. Tafa, Wakweya Chali, Lina Alemayehu, Fikregabrail Aberra Kassa, Ayalew Jejaw Zeleke, Tajudin Abdurhaman Hamza, Abel Beliyu Tamirat, Tadesse Misganaw, Alayu Bogale, Zufan Yiheyis Abriham, Yasin Nasir, Gutema Jebessa, Metmiku Yohannes, Betelhem Akililu Kebede, Matiwos Kidane Ayele, Melat Abdo, Getinet Habtamu, Addisu Gizat, Jody Phelan, Bryce Matlock, David A. Fidock, Susana Campino, Ashenafi Assefa, Cristian Koepfli, Fekadu Massebo, Fitsum G. Tadesse

**Affiliations:** 1 Armauer Hansen Research Institute, Addis Ababa, Ethiopia; 2 Department of Biology, Arba Minch University, Arba Minch, Ethiopia; 3 Department of Infection Biology, London School of Hygiene and Tropical Medicine, London, UK; 4 Malaria Centre, London School of Hygiene and Tropical Medicine, London, UK; 5 Radboud University Medical Centre, Nijmegen, the Netherlands; 6 Ethiopian Public Health Institute, Addis Ababa, Ethiopia; 7 University of Gondar, Gondar, Ethiopia; 8 University of Werabe, Werabe, Ethiopia; 9 Madda Walabu University, College of Medicine and Health Science, Bale Robe, Ethiopia; 10 Woldia University, Woldia, Ethiopia; 11 Dilla University, Dilla, Ethiopia; 12 Oak Ridge Institute for Science and Education, Oak Ridge, TN USA; 13 Department of Microbiology and Immunology, Columbia University Irving Medical Center, New York, NY USA; 14 Center for Malaria Therapeutics and Antimicrobial Resistance, Division of Infectious Diseases, Department of Medicine, Columbia University Irving Medical Center, New York, NY USA; 15 Institute for Global Health and Infectious Diseases, University of North Carolina, Chapel Hill, NC USA; 16 University of Notre Dame, Notre Dame, IN USA

**Keywords:** Parasite genomics, Antimicrobial resistance

## Abstract

The emergence of antimalarial drug resistance threatens malaria control and elimination efforts in Africa. Ethiopia, once a success story in case reduction, is now experiencing a resurgence. Here we examine key drug resistance genes (*Pfmdr1*, *Pfcrt*, *Pfk13*, *Pfdhfr* and *Pfdhps*) and mitochondrial genomes from 605 *Plasmodium falciparum* isolates collected across 15 districts in Ethiopia with varying transmission intensity and *Plasmodium vivax* co-endemicity. Although chloroquine was withdrawn for *P. falciparum* long ago, it remains the first-line treatment for *P. vivax*; this overlapping use may shape selection pressure in co-endemic settings that influences resistance markers to artemether–lumefantrine, the current first-line therapy for *P. falciparum*. A dominant *Pf*MDR1 NFSND haplotype, associated with reduced lumefantrine susceptibility, was identified alongside near fixation of the chloroquine-resistant *Pf*CRT CVIET haplotype in specific areas. Concerningly, *Pf*K13 variants associated with partial artemisinin resistance, R622I (10%), A675V (1.7%) and P441L (1.1%), were expanding. Multilevel models demonstrated robust, independent associations of R622I with *Pf*CRT CVIET and *Pf*DHFR AICNI, while ecological predictors were weaker and less consistent. These findings highlight genetic co-occurrence of *Pfcrt* and *Pfk13* mutations in *P. vivax*–*P. falciparum* co-endemic settings and can inform antimalarial policy in Ethiopia.

## Abstract (web summary)

Artemisinin partial resistance is expanding in Ethiopia and, together with a dominant lumefantrine-tolerant *Pf*MDR1 haplotype and co-occurring *Pfcrt* and *Pfk13* mutations, defines a complex multidrug-resistance landscape that may reflect overlapping treatment pressures in *Plasmodium vivax* and *Plasmodium falciparum* co-endemic settings.

## Main

Malaria, predominantly caused by *Plasmodium falciparum*, remains a major public health burden in sub-Saharan Africa, despite two decades of progress in diagnostics, vector control and artemisinin-based combination therapies (ACTs)^1^. Chemotherapy remains the cornerstone of malaria prevention, yet *P. falciparum* has developed resistance to nearly all antimalarials used over the past 70 years^2^, including artesunate monotherapy^3^ and several ACT partner drugs^4,5^. Artemisinin partial resistance (ART-R), first identified in Southeast Asia over 15 years ago^3,6^, has now been reported in multiple African countries^7–9^, posing a serious threat to malaria control and elimination.

In Africa, ART-R was first confirmed in Rwanda^10^, with subsequent reports from Uganda^11,12^, Tanzania^9^ and Eritrea^13^. These cases, characterized by delayed parasite clearance after ACT treatment, are linked to mutations in the *Pfkelch13* (*Pfk13*) beta-propeller domain^14–16^. Variants such as R561H (Rwanda)^10,17^, C469Y (Uganda)^12,18^ and R622I (Eritrea)^13^ are now established markers of ART-R. Although ACTs remain broadly effective in Africa, increasing reports of persistent parasitaemia after treatment with artemether–lumefantrine (AL)^19^, the most widely used ACT in Africa^20^, raise concerns of declining efficacy. Preserving lumefantrine’s efficacy is urgent, particularly given its inclusion in emerging regimens such as triple ACT^21^, and the non-ACT drug, ganaplacide–lumefantrine^22^, nearing the final stages of prequalification^23^.

Ethiopia adopted AL in 2004^24,25^, achieving major reductions in malaria cases through expanded health services, rapid diagnostics and vector control^26,27^. However, recent resurgence^28^ threatens elimination goals^29^. Potential contributing factors include climate variability and other human and environmental drivers^30^, and a range of biological threats such as insecticide resistance^31^, the spread of invasive mosquito species^32,33^, *Pfhrp2/3* gene deletion affecting rapid diagnostic test performance^34,35^ and the emergence of ART-R^36^.

One particularly concerning development is the increasing prevalence and spread of the *Pf*K13 R622I variant, first identified in northwestern Ethiopia in 2016^36,37^. Ethiopia’s malaria ecology is further complicated by *Plasmodium* *vivax* co-endemicity, for which chloroquine remains first-line therapy. Residual chloroquine exposure^38^, misdiagnosis^39^ and overlapping drug pressures^40,41^ may sustain selection for *P. falciparum* resistance. These dynamics raise the possibility of interactions between chloroquine resistance and emerging ACT tolerance, shaping a distinct resistance profile. This requires an in-depth genomic surveillance to track *P. falciparum* drug resistance and to inform treatment policies.

To address this, we conducted a nationwide genomic surveillance of *P. falciparum* between 2019 and 2023 across 15 Ethiopian districts with diverse disease ecology and risk status, focusing on markers of resistance to chloroquine, sulfadoxine–pyrimethamine (SP), ART-R and ACT partner drugs.

## Results

### Study site characteristics and demography of participants

**Fig. 1:**
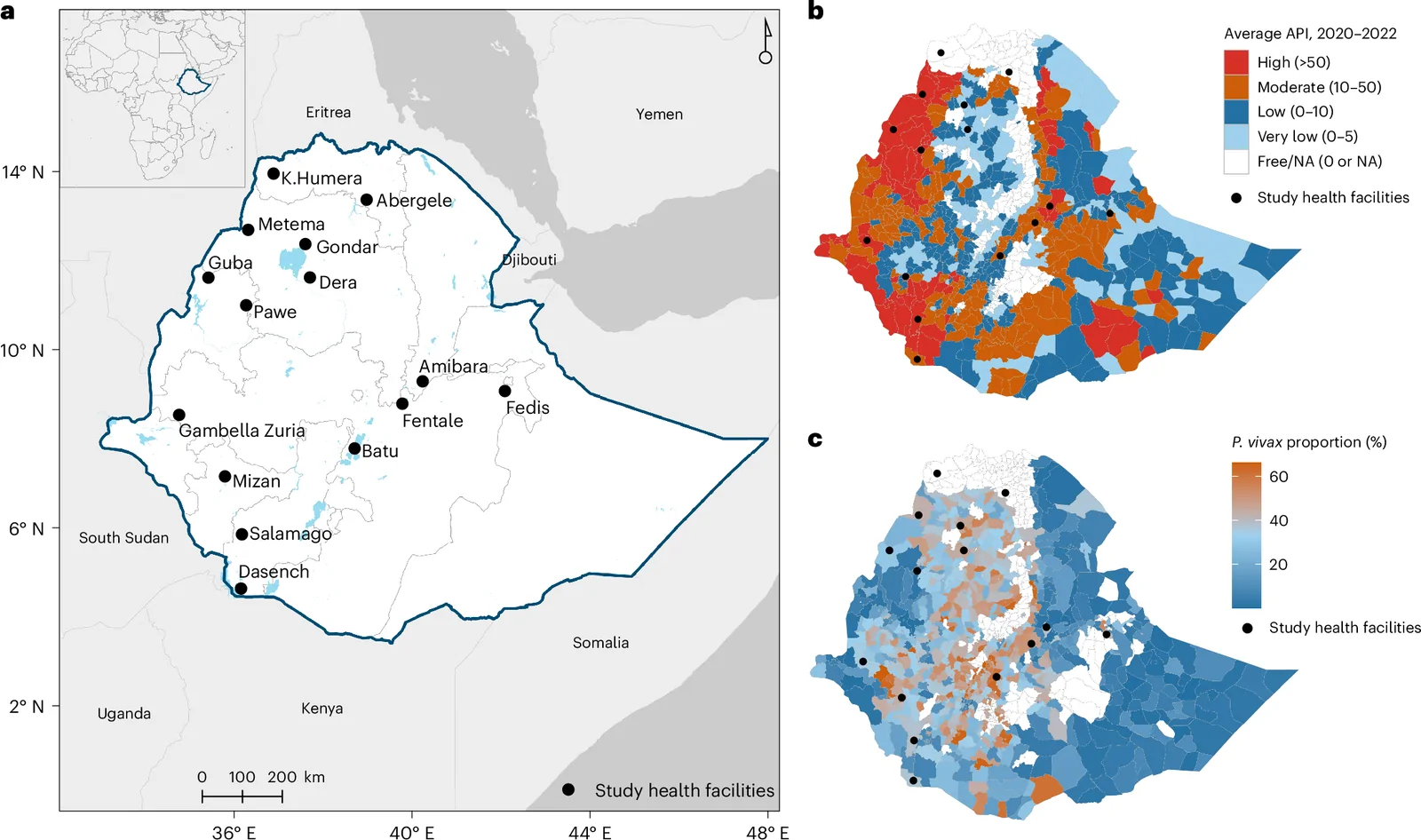
Map showing surveillance sites for antimalarial drug resistance (study health facilities) across Ethiopia are depicted in varying malaria transmission settings. **a**, A map showing study districts for antimalarial drug resistance surveillance across Ethiopia. **b**, The average API from 2020 to 2022, categorizing areas by transmission intensity: very low (light blue), low (dark blue), moderate (light orange) and high (orange). **c**, The proportion of *P. vivax* infections co-endemic with *P. falciparum* populations, with the colour scale indicating the proportion of *P. vivax* cases (ranging from 0% to >60%) per region. Study health facilities were chosen to represent diverse transmission conditions and *P. vivax* co-endemicity for robust antimalarial drug resistance surveillance. To assess disease hotspots and malaria transmission intensity, as well as characterize the distribution of *P. vivax*, we utilized malaria surveillance data from the Public Health Emergency Management system. The dataset covers confirmed malaria cases at the district level in Ethiopia from 2020 to 2022. We analysed district-level malaria incidence rates (cases/1,000 persons at risk/year) based on total confirmed malaria cases detected and population projections from the Ethiopia Office of the United Nations for the Coordination of Humanitarian Affairs^79^ to serve as denominators in incidence calculations. API was calculated as: API = (total confirmed malaria cases per year/population at risk) × 1,000. Malaria risk classification was based on the Ethiopian Ministry of Health stratification: malaria free (API 0), very low risk (0 < API ≤ 5), low risk (5 < API ≤ 10), moderate risk (10 < API ≤ 50) and high risk (API >50). The proportion of *P. vivax* cases was calculated as the ratio of *P. vivax* confirmed cases to the total confirmed malaria cases within each district. These proportions were aggregated and spatially mapped to illustrate geographic variation across the country. Source data

Malaria transmission intensity in Ethiopia varied widely across the 15 sites (2020–2022), with an annual parasite index (API) ranging from 1.0 in Fedis to 124.0 in Salamago. The proportion of *P. vivax* infections ranged from 2.4% in Gambella Zuria to 54.0% in Batu (Fig. 1 and Table 1). This diverse epidemiology provided a framework to investigate the distribution of antimalarial drug resistance markers and their potential interactions.

**Table 1:** Demographic characteristics and study site settings

Region	District	Female sex, % (*n*/*N*)	Median age, years (IQR)	Average API (2020–2022)	*P. vivax* proportion	Collected samples					Successfully sequenced samples						
						2019	2021	2022	2023	Total	*PfMit*	*Pfcrt*	*Pfdhps*	*Pfmdr1*	*Pfdhfr*	*Pfk13*	Total (≥1 successful target)
Afar	Amibara	41.0% (16/39)	21 (17.5–29)	34.3	4.4	–	39	–	–	39	33	33	33	33	33	33	33
Amhara	Dera	25.9% (7/27)	14 (7.5–20)	28.6	25.7	–	28	–	–	28	28	25	27	28	27	28	28
Amhara	Metema	23.8% (10/42)	20 (16–28)	66.0	18.4	–	27	15	–	42	38	42	41	36	42	42	42
Benishangul	Guba	37.2% (16/43)	15 (8.5–20.5)	82.2	8.8	–	29	14	–	43	30	41	43	43	43	43	43
Benishangul	Pawe	45.0% (18/40)	20 (14–25.2)	34.9	7.7	–	9	32	–	41	14	35	41	41	41	41	41
Gambella	Gambella Zuria	35.4% (17/48)	21 (11.8–25.2)	71.1	2.4	–	25	25	–	50	44	27	36	47	48	49	49
Gondar Zuriya	Gondar	18.6% (8/43)	18 (18–24.5)	48.6	32.7	–	–	30	17	47	44	32	33	33	33	35	44
Oromia	Batu/Zeway	25.6% (11/43)	25 (20–31)	5.5	54.0	–	45	–	–	45	40	40	40	40	40	40	40
Oromia	Fedis	23.4% (11/47)	25 (19.5–30)	1.0	15.6	–	47	–	–	47	45	44	45	45	45	45	45
Oromia	Fentale	27.3% (12/44)	18 (10.8–25)	8.7	23.7	–	44	–	–	44	44	42	44	34	44	42	44
SNNP	Dasenech	34.2% (13/38)	18.5 (10.5–26.5)	36.2	9.5	–	38	–	–	38	26	36	36	34	38	37	38
SNNP	Salamago	38.9% (14/36)	18 (9.5–25)	124.0	19.9	–	37	–	–	37	33	32	33	34	33	34	34
Southwest	Mizan	40.5% (17/42)	28 (20–32)	44.5	41.4	–	–	42	–	42	35	40	42	42	42	42	42
Tigray	K. Humera	16.7% (7/42)	23.5 (20–31.8)	14.2	23.8	–	42	–	–	42	30	42	40	36	42	42	42
Tigray	Tanqua Abergelle	22.2% (4/18)	17.5 (8.5–28.8)	6.6	14.9	19	–	–	–	19	19	19	19	19	19	19	19
Overall	Overall	30.57% (181/592)	20.0 (15.0–28.0)	40.4	20.2	19	410	158	17	604	503	530	553	545	570	572	584

API represents the number of confirmed malaria cases detected per 1,000 individuals per year (average from 2020 to 2022). API was calculated as: (total confirmed malaria cases per year ÷ population at risk) × 1,000. Ten participants with incomplete demographic data were excluded from the analysis. SNNP, South Nations and Nationalities and People Region.

**Extended Data Fig. 1:**
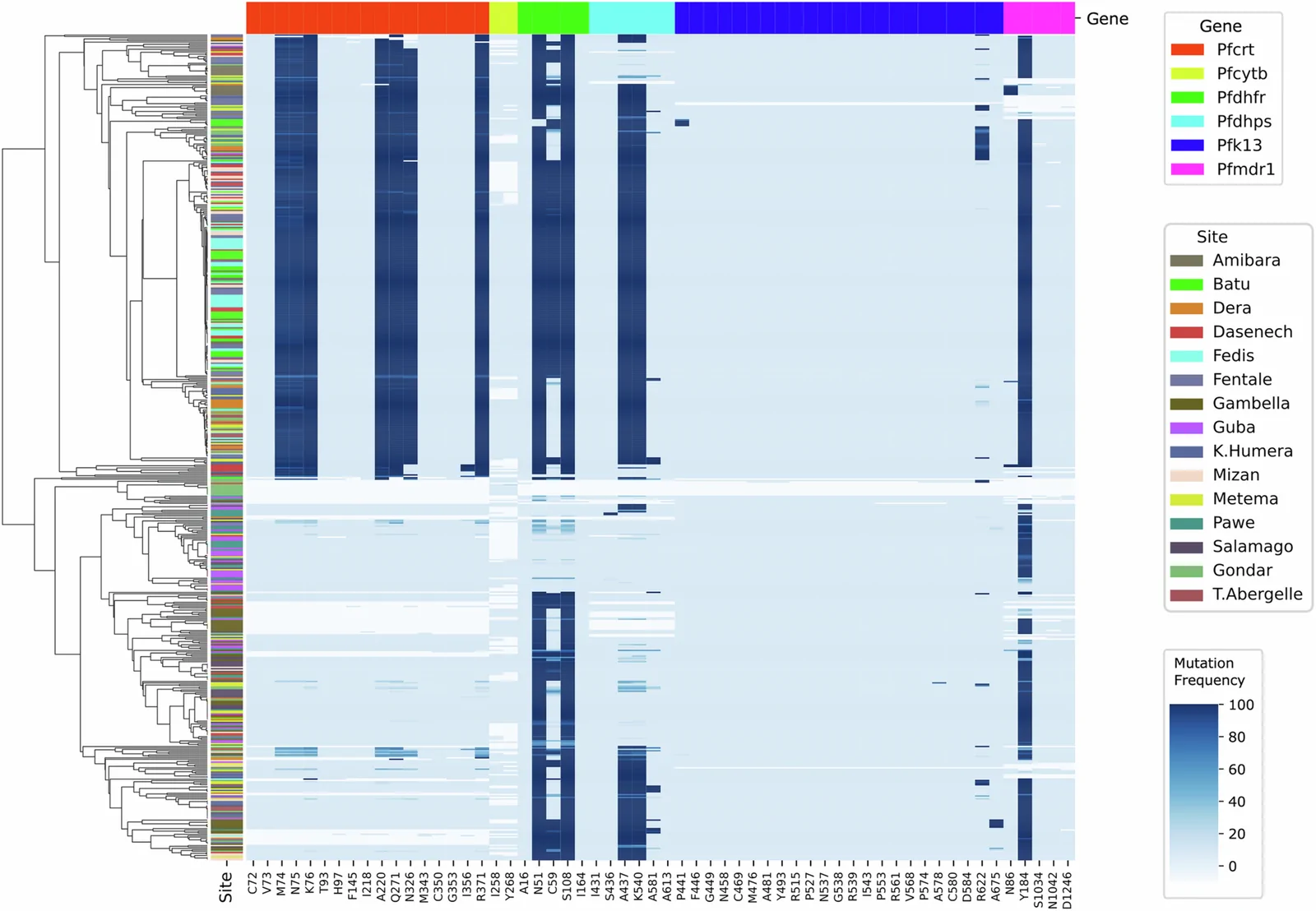
Cluster heatmap representing the frequency of mutations. Frequency is indicated by 0% light blue to 100% dark blue for each sample (row) and at each selected amino acid for a given gene (column). A white color (valued at -20 arbitrary units) indicates that no reads were available for a certain sample at a certain position. Rows are hierarchically clustered based on row composition, indicating the relatedness between samples in the dataset according to the frequency of mutation(s) for that sample, and colored to indicate the district to which the sample belongs. Each visualized amino acid is denoted at the bottom of the heatmap in numerical order, and the gene to which the amino acid belongs is color-coded at the top of the heatmap. Source data

**Extended Data Fig. 2:**
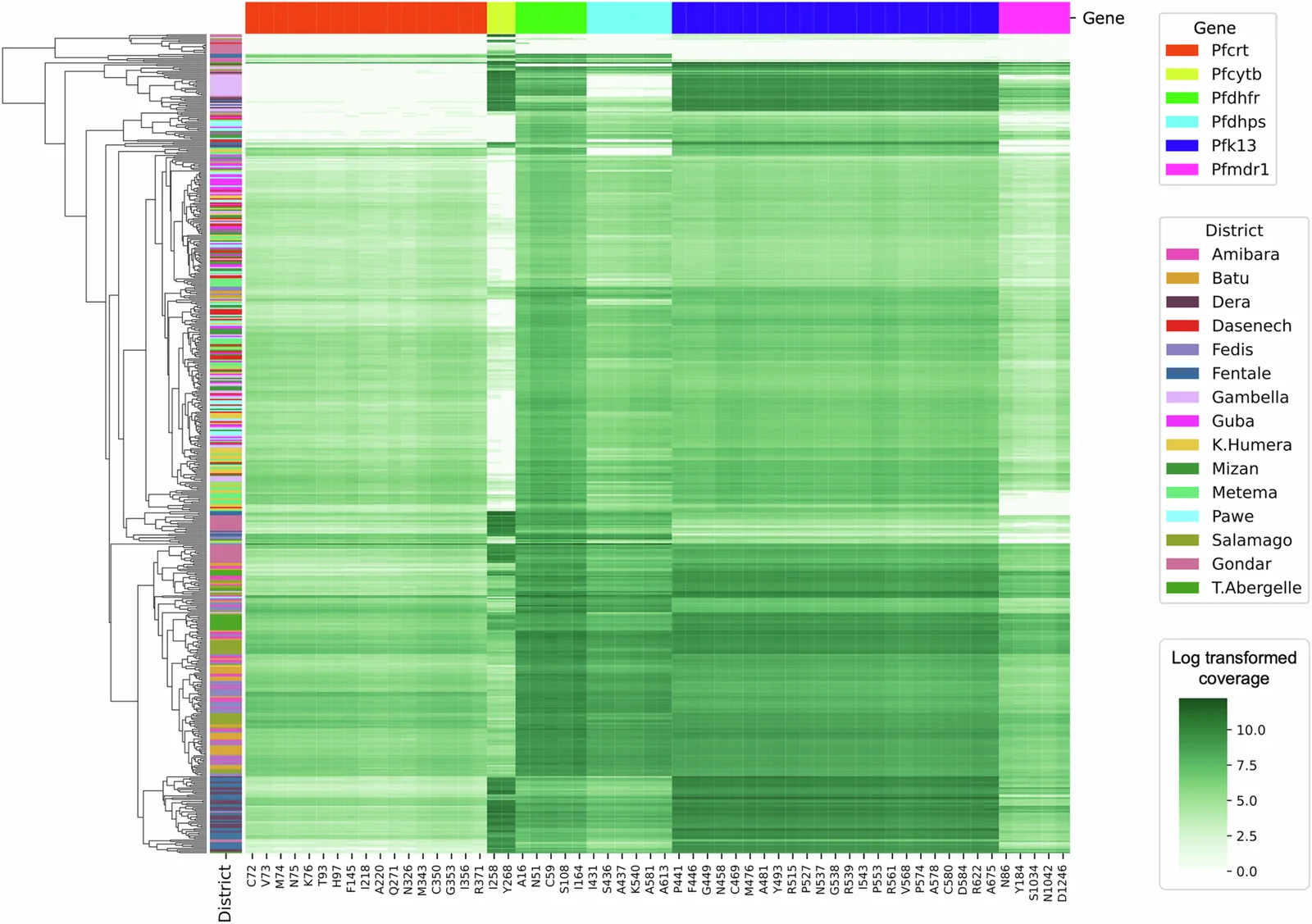
Cluster heatmap representing log-transformed coverage. Coverage is indicated by green color scale for each sample (row) and at each selected amino acid for a given gene (column). White indicates that no reads were available for a certain sample at a certain position. Rows are hierarchically clustered based on row composition, indicating the relatedness between samples in the dataset according to the frequency of mutation(s) for that sample, and colored to indicate the district to which the sample belongs. Each visualized amino acid is denoted at the bottom of the heatmap in numerical order, and the gene to which the amino acid belongs is color-coded at the top of the heatmap. Source data

**Extended Data Fig. 3:**
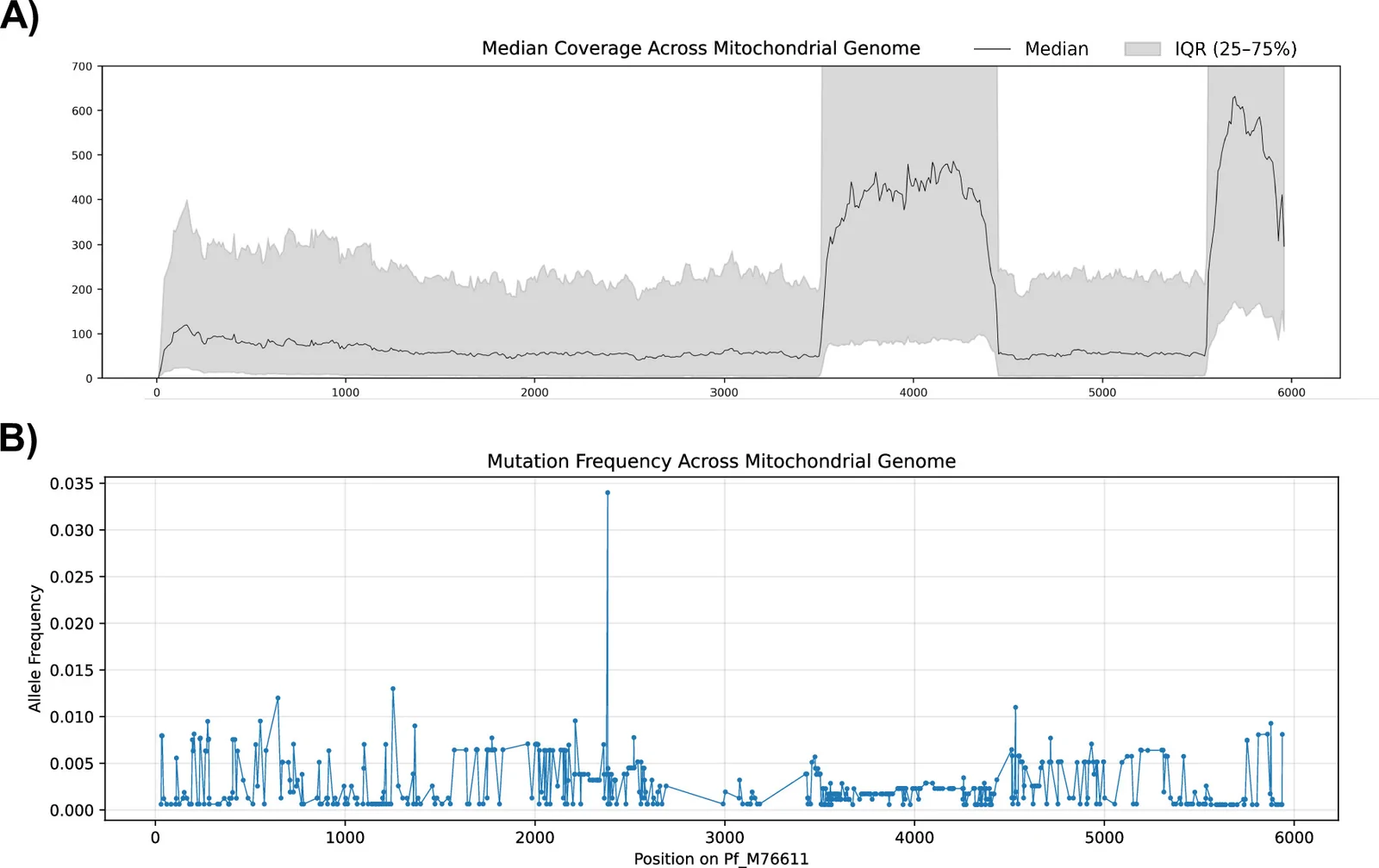
Coverage and variation across mitochondrial genome. **A)** Median coverage across the entire mitochondrial genome. Grey shading represents IQR (25%-75%). **B)** Allele frequency for each variant observed on the mitochondrial genome. Source data

Targeted amplicon sequencing of five *P. falciparum* drug resistance genes (*Pfcrt, Pfdhps, Pfmdr1, Pfdhfr* and *Pfk13*) and the mitochondrial genome was performed using 605 patient samples collected between 2019 and 2023. After quality control and removal of low-depth coverage samples, 584 samples were retained for variant analysis. Sample counts for successful variant analysis varied for each sequenced gene: mitochondrial genome (*n* = 504), *Pfcrt* (*n* = 533), *Pfdhps* (*n* = 553), *Pfmdr1* (*n* = 556), *Pfdhfr* (*n* = 570) and *Pfk13* (*n* = 575) (Table 1). Variant calling identified 349 synonymous and 684 non-synonymous mutations across the dataset. While the mitochondrial genome was sequenced with adequate coverage, genetic variation was limited (Extended Data Figs. 1–3).

Overall, 13 participants had incomplete demographic data. The remaining 592 participants (Table 1) had a median age of 20.0 years (interquartile range (IQR) 15.0–28.0), with 30.6% of them being female (181/592). Female representation varied across sites (16.7–45.0%), as did median age (14.0–28.0 years).

### The persistence of *Pfdhfr* and *Pfdhps* mutations associated with antifolate resistance

**Fig. 2:**
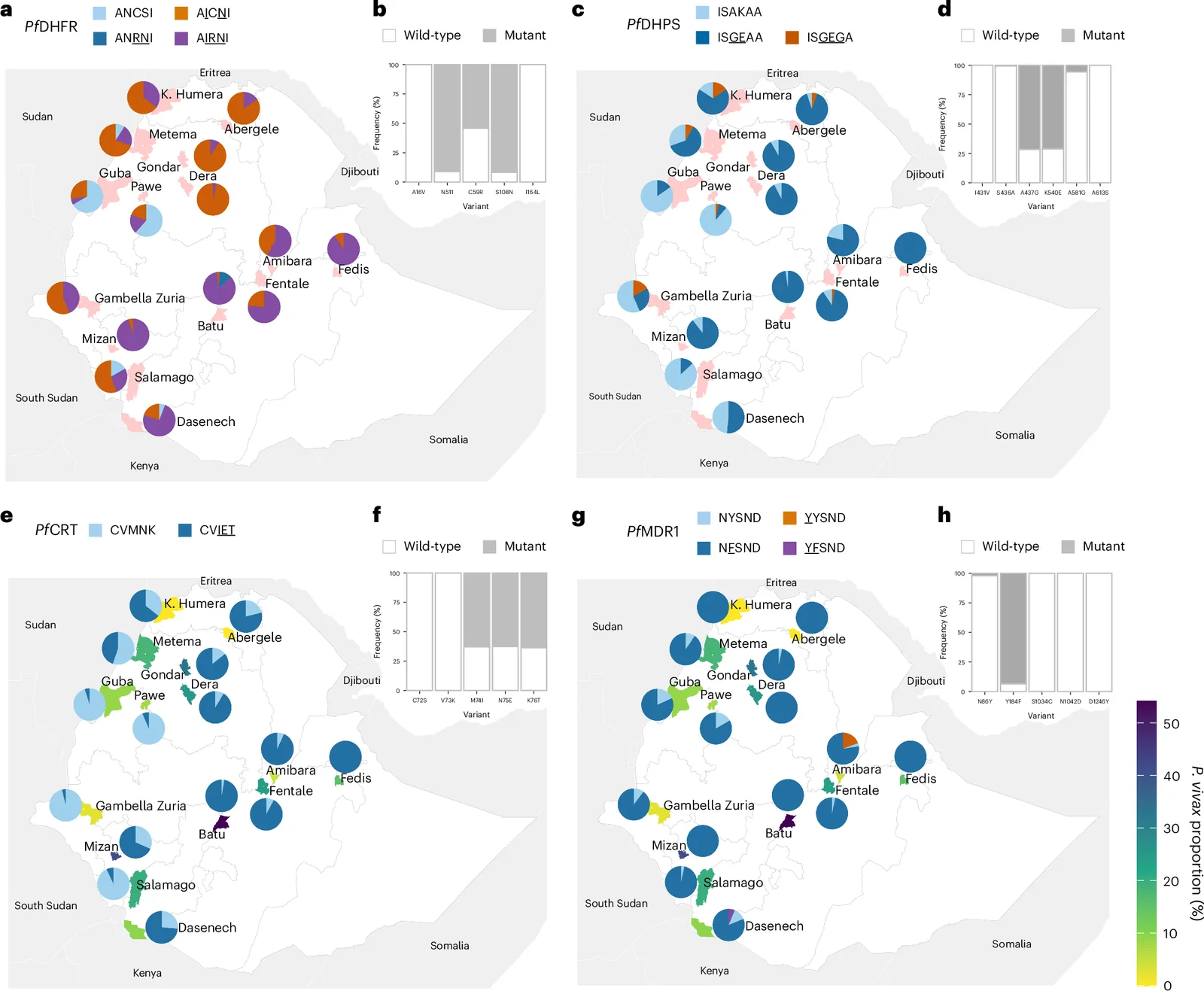
Geographic distribution and prevalence of *Pf*DHFR, *Pf*DHPS, *Pf*CRT and *Pf*MDR1 haplotypes and mutations in Ethiopian *P. falciparum* populations. **a**,**c**,**e**,**g**, Geographic distribution and proportions of observed haplotypes as pie charts are shown for *Pf*DHFR (**a**), *Pf*DHPS (**c**), *Pf*CRT (**e**) and *Pf*MDR1 (**g**). The pink colour in **a** and **c** denotes the districts represented by the pie charts. In **e** and **g**, the districts are colour-coded to represent proportions of observed *P. vivax* in that district (see the legend at the bottom right of the figure). **b**,**d**,**f**,**h**, Bar charts representing the proportion of mutant (grey) and wild-type (white) alleles for each variant are shown for *Pf*DHFR (**b**), *Pf*DHPS (**d**), *Pf*CRT (**f**) and *Pf*MDR1 (**h**). Source data

Although SP was discontinued over two decades ago, resistance-associated mutations in *Pfdhfr* and *Pfdhps* remain widespread. The most common *Pf*DHFR haplotypes were triple mutant AIRNI (A16-51I-59R-108N-I164, 245/490, 50.0%) and double mutant AICNI (A16-51I-C59-108N-I164, 194/490, 39.6%), with spatial variation across districts (Fig. 2a,b, Extended Data Table 1 and Supplementary Table 1). Among the 569 isolates, N51I (91.0%) and S108N (91.7%) were the most prevalent variants, while C59R appeared in 54.1% of isolates, and no mutations were observed at codons 16 and 164 (Fig. 2c and Supplementary Table 1). In the *Pfdhps* gene, the double mutant ISGEAA dominated (I431-S436-437G-540E-A581-A613, 330/502, 65.7%), followed by the wild-type haplotype ISAKAA (30.9%) and the triple mutant ISGEGA (I431-S436-437G-540E-581G-A613, 17/502, 3.4%), with all showing site-specific patterns (Fig. 2c,d, Extended Data Table 1 and Supplementary Table 1). High frequencies of A437G (395/552, 71.6%) and K540E (392/552, 71.0%) drove most of the variation, while A581G was rare (31/552, 5.6%), and S436, A613 and I431 remained wild-type.

**Extended Data Fig. 4:**
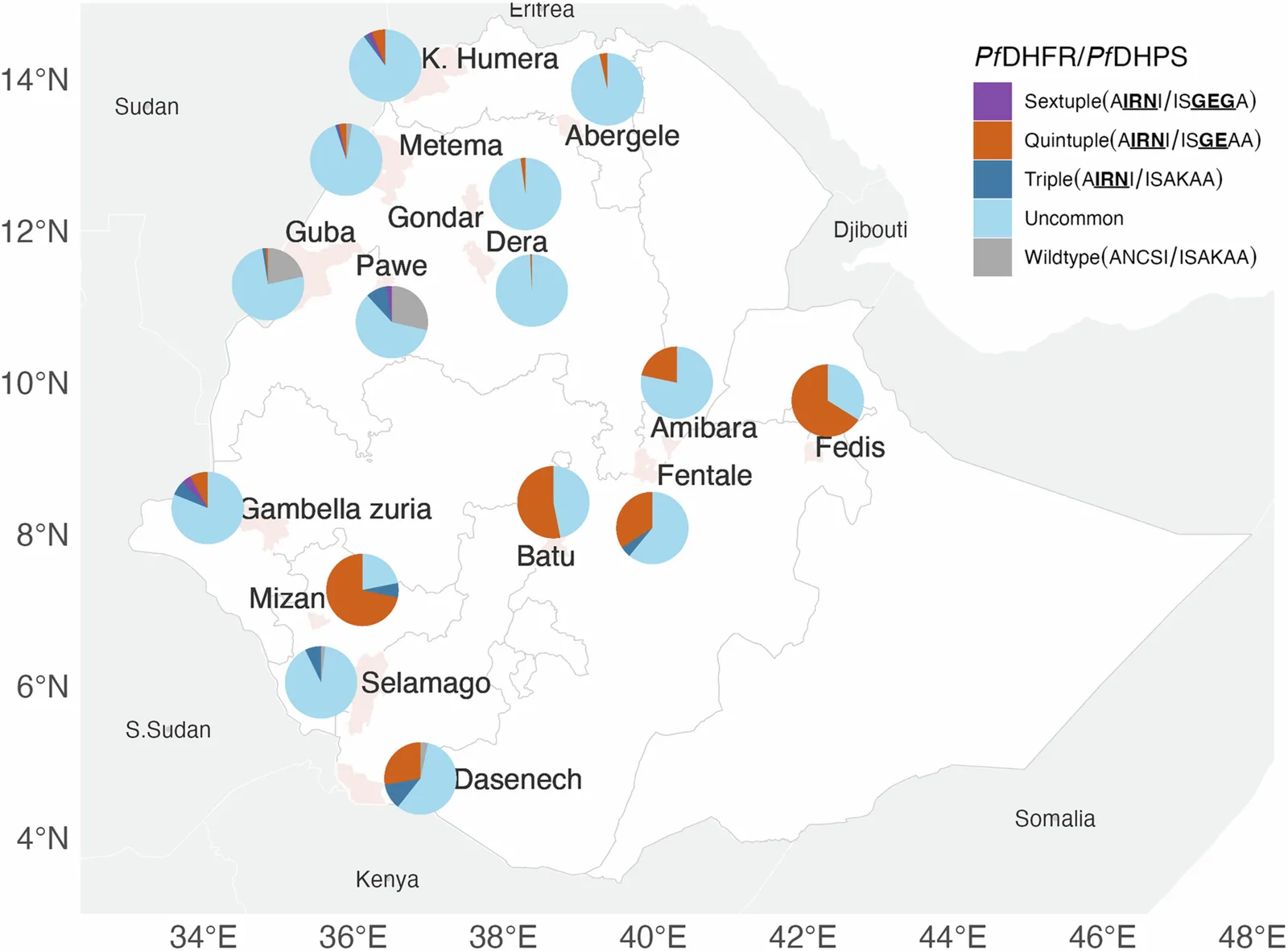
Geographic distribution and prevalence of combined *Pf*DHFR and *Pf*DHPS haplotypes in Ethiopian *P. falciparum* populations. Geographic distribution and proportions of observed haplotypes as pie charts for combined *Pf*DHFR and *Pf*DHPS haplotypes. The districts which the pie charts represent are annotated in pink. Haplotypes are indicated by the color scale. The underlying data is presented in Supplementary Table 1, and a list of combined *Pf*DHFR and *Pf*DHPS haplotypes observed in this study with resistance phenotype from the literature is presented in Supplementary Table 2. Source data

The *Pf*DHFR–*Pf*DHPS combined quintuple haplotype (AIRNI/ISGEAA), which is linked with high-level SP resistance, was commonly detected in 194 of 453 samples (42.8%). The sextuple haplotype (AIRNI/ISGEGA) was rare (1.8%, 8/453), while the triple (AIRNI/ISAKAA, 6.8%, 31/453) and wild-type (ANCSI/ISAKAA, 7.7%, 35/453) haplotypes were also observed. A notable finding was the widespread presence of the otherwise uncommon *Pf*DHFR AICNI (A16/N51I/C59/S108N/I164) haplotypes, which appeared in combination with wild-type (ISAKAA), double (ISGEAA) or triple (ISGEGA) *Pf*DHPS mutants. The most frequent combinations were AICNI/ISGEAA (112/453, 24.7%) and AICNI/ISAKAA (54/453, 11.9%) (Supplementary Table 1). These haplotype combinations were categorized as ‘uncommon’ (Supplementary Table 2). Distinct geographic clustering of combined antifolate resistance haplotypes was observed across Ethiopia (Extended Data Fig. 4). Quintuple haplotype (N51I + C59R + S108N + A437G + K540E) dominated along major transportation corridors (Djibouti–Addis Ababa and Kenya–Addis Ababa), whereas AICNI-containing variants were concentrated in northwestern and southwestern regions that experienced recent malaria resurgence.

**Extended Data Fig. 5:**
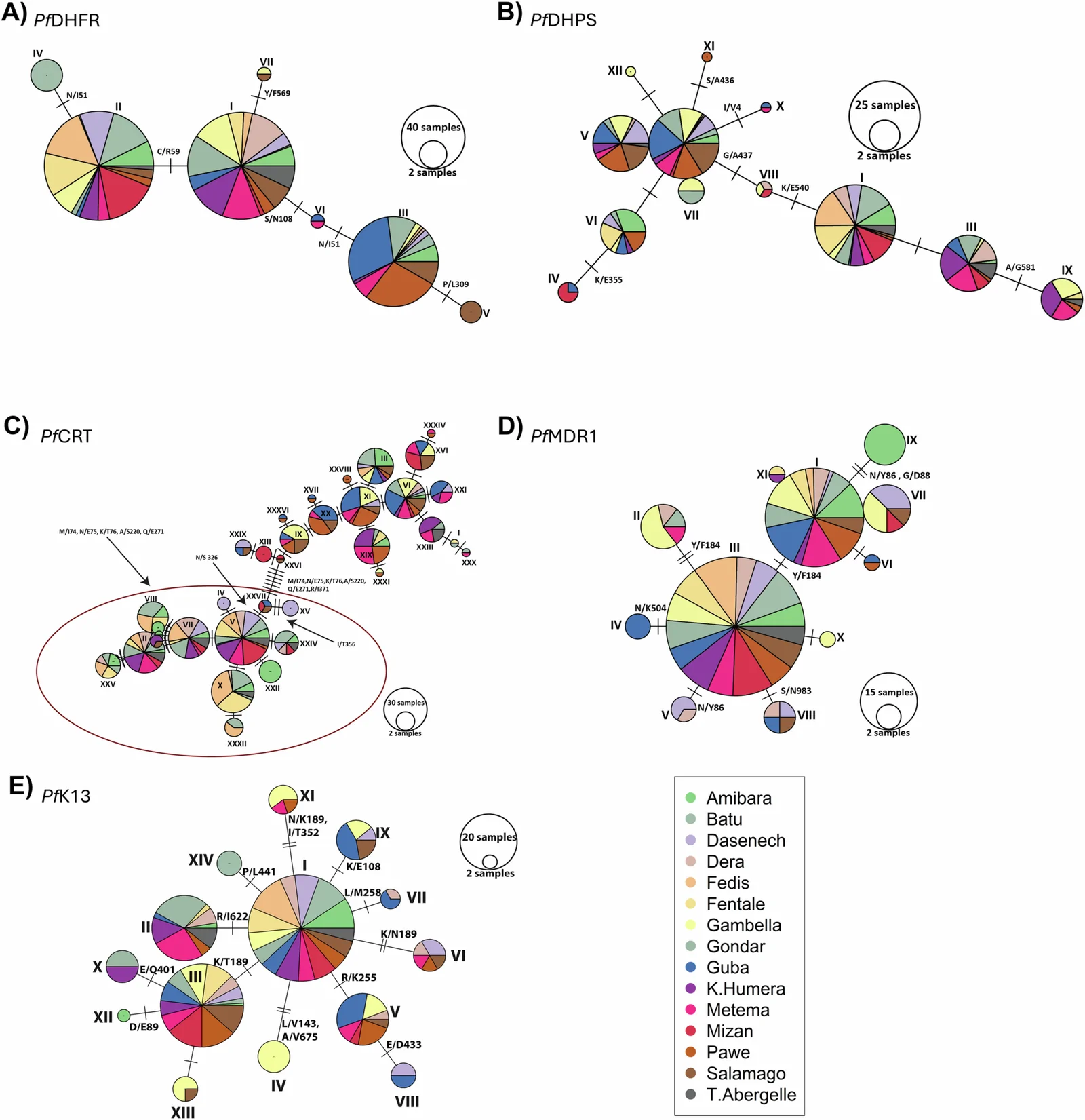
Median-joining networks. **A**) *Pf*DHFR (N=596), **B**) *Pf*DHPS (N=582), **C**) *Pf*CRT (N=488), **D**) *Pf*MDR1 (N=546) and **E**) *Pf*K13 (N=584) haplotypes, including all detected non-synonymous and synonymous polymorphisms, present in more than one sample. Circle size represents haplotype frequency and colors indicate geographic origin. Connecting lines indicate single nucleotide changes and arrows denote key mutations associated with resistance. Haplotypes are indicated using Roman numerals. *Pf*DHFR cluster II (the triple mutant A**IRN**I, 43.1%)) was predominantly found in Batu, Fedis, Fentale, Mizan, Amibara, Dasenech and Salamago, while cluster I (the double mutant A**I**C**N**I, 38.7%) was predominantly found in Dera, Gondar, Gambella, Metema, K. Humera and Abergele, and the wild-type (Cluster III, 16.1%) in Guba and Pawe. *Pf*DHPS cluster I containing K540E was frequent in Fentale, Batu, and Fedis, whereas G437A-dominant Clusters II and V were more common in Guba, Pawe and Salamago. *Pf*CRT haplotype clusters V (n=117) and X (n=42), along with *Pf*MDR1 haplotype clusters III (n=434) and I (n=75) were found across several districts. Major *Pf*K13 haplotypes were I (n=375) and III (n=104). *Pf*K13 R622I (n=57) concentrated in Gondar (n=14), K. Humera (n=7), Amibara (n=1), Pawe (n=2), Dera (n=9), Metema (n=10). R255K (n=40): Guba (n=11), Dera (n=7), Pawe (n=7), Salamago (n=4). A675V (n=10): Gambella, Mizan, Salamago. P441L (n=6): Batu/Zeway (n=5), Salamago (n=1). R622I, R255K, A675V, P441L clustered in north-central, west, southwest, south-central regions, respectively.

Minimal-spanning networks incorporating both synonymous and non-synonymous single nucleotide polymorphisms (SNPs) revealed three major *Pf*DHFR clusters: AIRNI (43.1%), AICNI (38.7%) and the wild-type (16.1%) (Extended Data Fig. 5a and Supplementary Table 4). For *Pf*DHPS, K540E-containing cluster I was frequent in central and eastern districts, whereas G437A-dominant clusters II and V were more common in western and southern districts (Extended Data Fig. 5b).

### Prevalence of mutant haplotypes of chloroquine resistance markers (*Pfcrt* and *Pfmdr1*) remains high

Analysis of *Pf*CRT variants in 533 isolates revealed that the chloroquine resistance-associated CVIET (C72-V73-74I-75E-76T) haplotype was predominant (61.2%, 301/492) compared with the wild-type haplotype CVMNK (38.8%, 191/492) (Fig. 2e,f). CVIET was heterogeneously distributed, reaching fixation in Fedis, and near fixation in Batu, Amibara, Fentale and Dera. High frequencies were also observed in Gondar, T. Abergelle, Dasenech, Mizan and K. Humera. Conversely, CVMNK was predominant (>90%) in Gambella, Guba, Pawe and Salamago. Metema exhibited nearly equal distributions of CVIET and CVMNK (Extended Data Table 1). Codon variants 74–76 showed similar frequencies (62.5–63.5%), whereas codons 72 and 73 remained wild-type. High mutation prevalence was also observed at codons 220 (64.4%, 335/520), 271 (64.3%, 336/523), 326 (60.2%, 310/515), 371 (64.4%, 334/519) and I356T (1.7%, 9/524) (Supplementary Table 1). A minimal-spanning haplotype network revealed two distinct haplotype groups separated by seven nucleotides (Extended Data Fig. 5c).

In *Pf*MDR1, a known secondary modulator of chloroquine resistance, the NFSND haplotype (N86-184F-S1034-N1042-D1246) containing the Y184F variant and the reference N86 allele, was highly prevalent, detected in 93.0% of samples (449/483), whereas the wild-type NYSND haplotype was much less common (26/483, 5.4%; Fig. 2g,h). YFSND (86Y-184F-S1034-N1042-D1246, 2/483, 0.4%) and YYSND (86Y-Y184-S1034-N1042-D1246, 6/483, 1.2%) were detected infrequently. The NFSND haplotype was dominant across all surveyed districts (Fig. 2g,h and Extended Data Table 1) ranging from 77.4% (24/31) in Amibara to 100% in several districts (Batu, Dera, Fedis, K. Humera, Mizan and T. Abergelle). The wild-type NYSND haplotype was observed at a relatively high prevalence in Guba (6/33, 18.2%). Variation was predominantly driven by codon 184, with Y184F present in 93.5% (503/538) of samples and detected in all districts, ranging from 75.8% in Amibara (25/33) to 100% in several districts (Batu, Dera, Fedis, Guba, K. Humera, Mizan and Tanqua Abergelle) (Supplementary Table 1). Codon 86 remained predominantly wild-type (98.0%, 531/542), while codons S1034, N1042 and D1246 were entirely wild-type (Fig. 2h and Supplementary Table 1). A minimal-spanning haplotype network identified two major haplotypes (Extended Data Fig. 5d).

### Transmission intensity and *P. vivax* co-endemicity shape distribution of CQ and SP resistance markers

**Extended Data Fig. 6:**
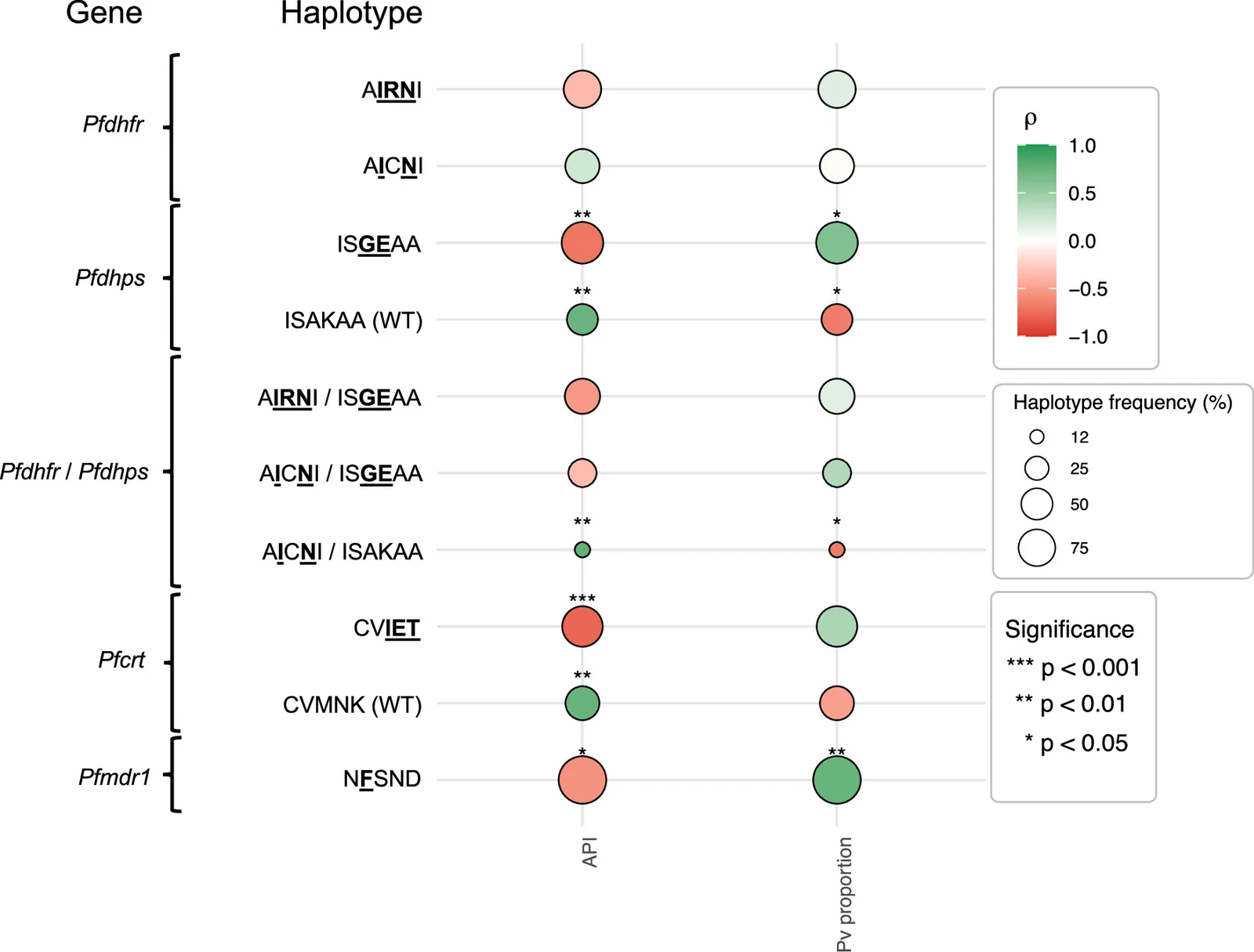
Correlation between haplotypes and the Annual Parasite Index (API) and the proportion of *P. vivax* infections. Bubble heat map showing positive (green scale) and negative (red scale) Spearman correlation coefficients between the proportion of isolates with the respective genetic variants (wild-type and mutant) and both the API (left column) and the proportion of *P. vivax* infections out of the total infections detected in the district (right column). Haplotype frequency is indicated by the size of the bubble, and statistical significance is denoted by asterisks. The mutant variants are highlighted in bold and underlined. Statistical significance was assessed using a two-sided Spearman rank-correlation test. Source data

The marked spatial heterogeneity observed in our study is particularly noteworthy. Spatial distributions in SP- and chloroquine (CQ)-resistance markers were strong, with distributions of SP- and CQ-resistance markers strongly linked to transmission intensity (API) and *P. vivax* co-endemicity (Extended Data Fig. 6). The *Pf*DHPS ISGEAA haplotype (*ρ* = −0.7, *P* = 0.004), *Pf*CRT CVIET (*ρ* = −0.78, *P* = 0.0006) and *Pf*MDR1 NFSND (*ρ* = −0.57, *P* = 0.0252) were more frequent in low transmission areas. Conversely, *Pf*DHPS ISGEAA (*ρ* = 0.61, *P* = 0.0163) and *Pf*MDR1 NFSND (*ρ* = 0.73, *P* = 0.0022) were positively associated with higher *P. vivax* co-endemicity.

### *Pfk13* mutations associated with artemisinin resistance are spatially clustered

**Fig. 3:**
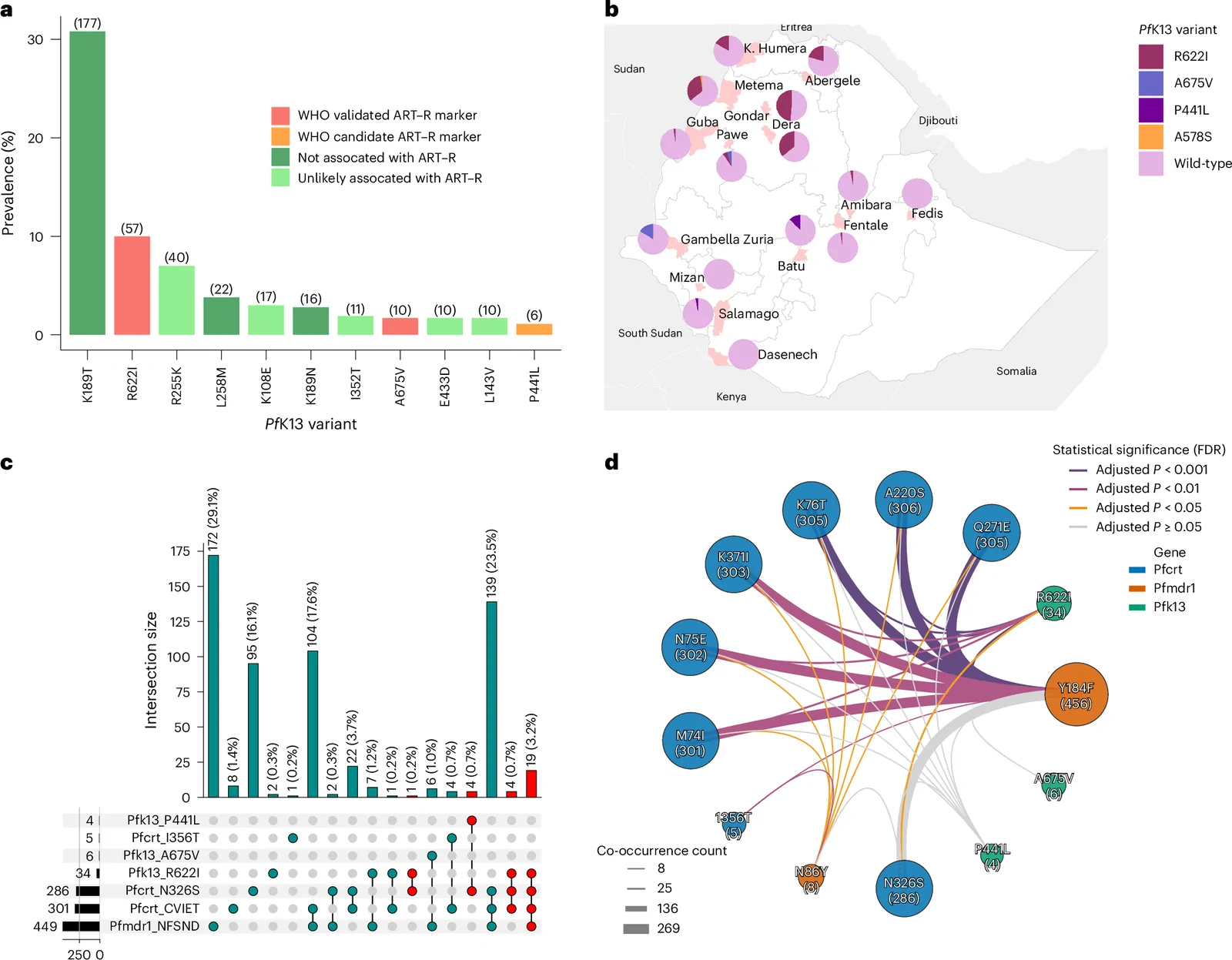
Prevalence, distribution and co-occurrence of *P. falciparum* drug resistance mutations in Ethiopia. **a**, *Pf*K13 variant prevalence, presenting all non-synonymous variants with overall frequency of >1%. Variants are labelled by WHO-validated ART-R markers (R622I and A675V), WHO candidate ART-R markers (P441L), variants not associated with ART-R (K189T, K189N and L258M) and variants unlikely to be associated with ART-R (R255K, I352T, E344D and L134V). **b**, The geographic distribution of *Pf*K13 variants. Pie charts show mutation proportions per location. R622I was most prevalent in Gondar (17/35, 48.57%). **c**, An upset intersection plot: co-occurrence of prevalent *P. falciparum* mutations. The most prevalent haplotype was *Pf*MDR1 NFSND (*n* = 449), commonly found with *Pf*K13 R622I (*n* = 48), A675V (*n* = 10) and P441L (*n* = 6). Red highlighting indicates the co-occurrence of *Pf*K13 variants (both the validated artemisinin resistance marker R622I and the candidate marker P441L) with the *Pf*CRT N326S background variant, a known facilitator of chloroquine resistance and a lumefantrine susceptibility marker. Specifically, *Pf*CRT N326S co-occurred with *Pf*K13 R622I in 45 isolates and with *Pf*K13 P441L in 6 isolates. *Pf*CRT CVIET and *Pf*K13 R622I were found together in 40 isolates. Notably, *Pf*CRT I356T and PfK13 R622I were never observed together. **d**, The chord diagram visualizes the pairwise co-occurrence relationships among mutations in the chloroquine resistance transporter gene (*Pfcrt*), the multidrug-resistance gene 1 (*Pfmdr1*) and the kelch13 gene (*Pfk13*). Each node represents a specific mutation, coloured by its gene of origin: *Pfcrt* (blue), *Pfmdr1* (orange) and *Pfk13* (green). The size of each node is proportional to the mutation’s frequency in the dataset. The ribbons (chords) connecting nodes indicate a co-occurrence relationship, with the width being proportional to the number of samples where both mutations were present. Pairwise isolate-level associations were assessed using 2 × 2 contingency tables, with Fisher’s exact or Yates-corrected *χ*^2^ tests applied as appropriate and Benjamini–Hochberg false discovery rate correction The colour of the ribbon denotes the statistical significance of the association based on false discovery rate-adjusted *P* values: purple (adjusted *P* < 0.001), red (adjusted *P* < 0.01), orange (adjusted *P* < 0.05) and grey (adjusted P ≥ 0.05, non-significant). To reduce the impact of mixed infections, positions with variant allele frequencies between 5% and 95% were excluded from the co-occurrence analysis. This ensures that the co-occurring mutations depicted are probably present within a single parasite genome. Samples with evidence of multiple genomes at a given position were therefore not included, minimizing potential bias from higher transmission areas where mixed infections are more common. Source data

Analysis of *Pfk13* in 572 isolates revealed diverse mutations linked with ART-R. The validated marker R622I was most prevalent (10.0%, 57/572), followed by A675V (1.7%, 10/572), while P441L (1.1%, 6/571) was rare (Fig. 3a and Supplementary Table 1). These markers exhibited strong regional variations. R622I was the most prevalent in the northwest, reaching up to 48.6% in Gondar, but was also detected at lower frequencies in central districts, such as Amibara (3%, 1/33) and Fentale (2.4%, 1/42). A675V was observed in western districts in Gambella Zuria (16.3%, 8/49) and Pawe (4.9%, 2/41), while P441L was primarily found in Batu (12.5%, 5/40), with an additional case detected in Salamago (Fig. 3b).

Several non-synonymous variants outside the beta-propeller domain were also detected, including K189N (16/575, 2.8%), K189T (177/575, 30.8%), R255K (40/575, 7.0%), L258M (22/575, 3.8%), I352T (11/575, 1.9%) and E433D (10/575, 1.7%) (Extended Data Fig. 5e and Supplementary Table 3). None of the remaining WHO-validated ART-R mutations were detected (Supplementary Table 5).

### *Pfk13* mutants co-occur with *Pfcrt* and *Pfmdr1* mutations

Analysis of mutation co-occurrence revealed complex, region-specific patterns involving *Pfk13, Pfcrt* and *Pfmdr1*. To ensure comparisons reflected single parasite genome associations, samples with mixed-genotype infections (variant allele frequency ≥5% and ≤95%) at relevant loci were excluded. The *Pfk*13 R622I variant significantly co-occurred with multiple *Pf*CRT variants (M74I, N75E, K76T, A220S, Q271E, N326S and R371I) and with *Pf*MDR1 NFSND (Fig. 3c,d). A675V (*n* = 6) was observed only with *Pf*MDR1 Y184F, while P441L (*n* = 4) was found exclusively with *Pf*CRT variants (M74I, N75E, K76T, A220S, Q271E, N326S and R371I) and *Pf*MDR1 Y184F.

**Extended Data Fig. 7:**
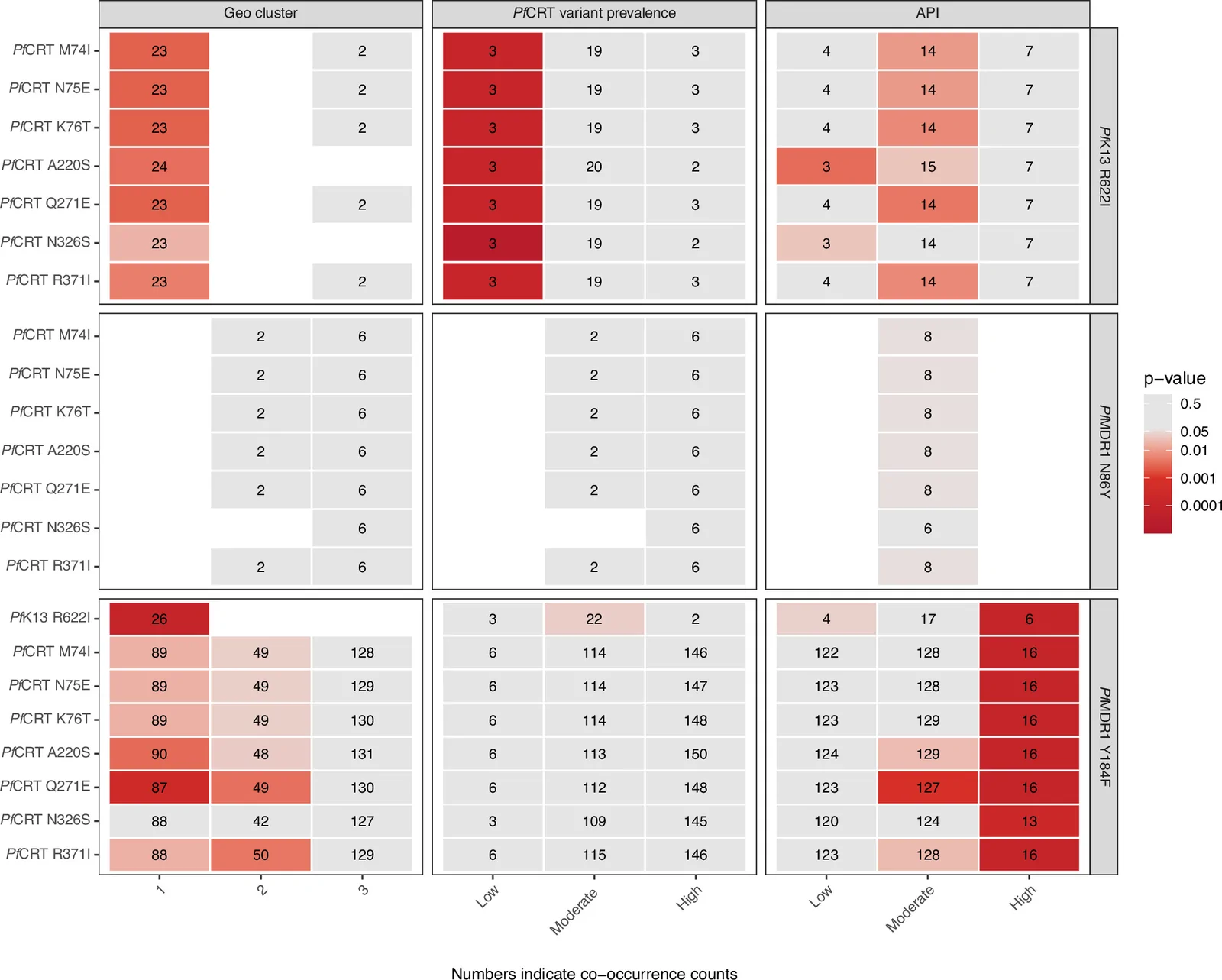
Heatmap illustrating the statistical significance of variant co-occurrence across different strata, including geographical cluster, *Pf*CRT variant prevalence and API. Each tile represents a specific variant pair within a stratum group, with variant 1 on the left-hand side and variant on the right-hand side of the heatmap. The color intensity of the tiles signifies the statistical significance (p-value) of the co-occurrence, with darker red indicating more significant P-values. Numbers indicate co-occurrence counts in each tile. Tiles where there is a co-occurrence count of ≤1 are shown in white. The x-axis displays the various groups within each stratum type, such as low, moderate, and high for API and *Pf*CRT variant prevalence (determined by seven key *Pf*CRT variants M74I, N75E, K76T, A220S, Q271E, N326S, and R371I), and clusters 1, 2, and 3 for geography. Geographical cluster 1 included areas in the North/West (districts Dera, Guba, K. Humera, Metema, Pawe, Gondar, and Tanqua Abergelle), cluster 2 includes districts Dasenech, Mizan, Salamago and Gambella zuria, while cluster 3 includes Amibara, Batu/Zeway, Fedis, and Fentale. API = Annual Parasite Index. Statistical significance of variant co-occurrence was assessed using a two-sided chi-square and/or fisher exact test when it is applicable. Source data

Stratified analyses demonstrated that these co-occurrence patterns were highly sensitive to population structure. Associations between R622I and *Pf*CRT/*Pf*MDR1 variants persisted only in the northwestern districts where R622I was present. Stratification by *Pf*CRT allele frequency (low *n* = 117, moderate *n* = 197 and high *n* = 185) revealed strong associations in low-prevalence strata and limited associations in high-prevalence strata. Stratification by transmission intensity (low API 0–10, moderate API 10–50 and high API ≥50) indicated that R622I co-occurred with *Pf*CRT markers primarily in moderate-API areas (Extended Data Fig. 7). Notably, *Pfk13* mutation prevalence itself was not correlated with API or *P. vivax* co-endemicity at a site level (Extended Data Fig. 6). To address confounding more formally, we applied mixed-effects logistic regression and Bayesian modelling, restricted to districts with confirmed circulation of the *Pfk13* R622I mutation. Across frameworks, R622I showed robust, independent associations with *Pf*CRT CVIET (adjusted odds ratio of 3.19, 95% CI 1.60–6.34; *P* = 0.001) and *Pf*DHFR AICNI (adjusted odds ratio of 5.16, 95% CI 1.60–16.70; *P* = 0.006), while associations with *Pf*DHPS ISGEAA and ecological predictors were weaker and imprecise. Bayesian estimates confirmed effect directions with high posterior probabilities, and site-level variance was modest, indicating that much of the geographic signal was explained by included genetic covariates (Extended Data Table 2).

### Ethiopian *P. falciparum* forms geographically and epidemiologically distinct clusters

**Fig. 4:**
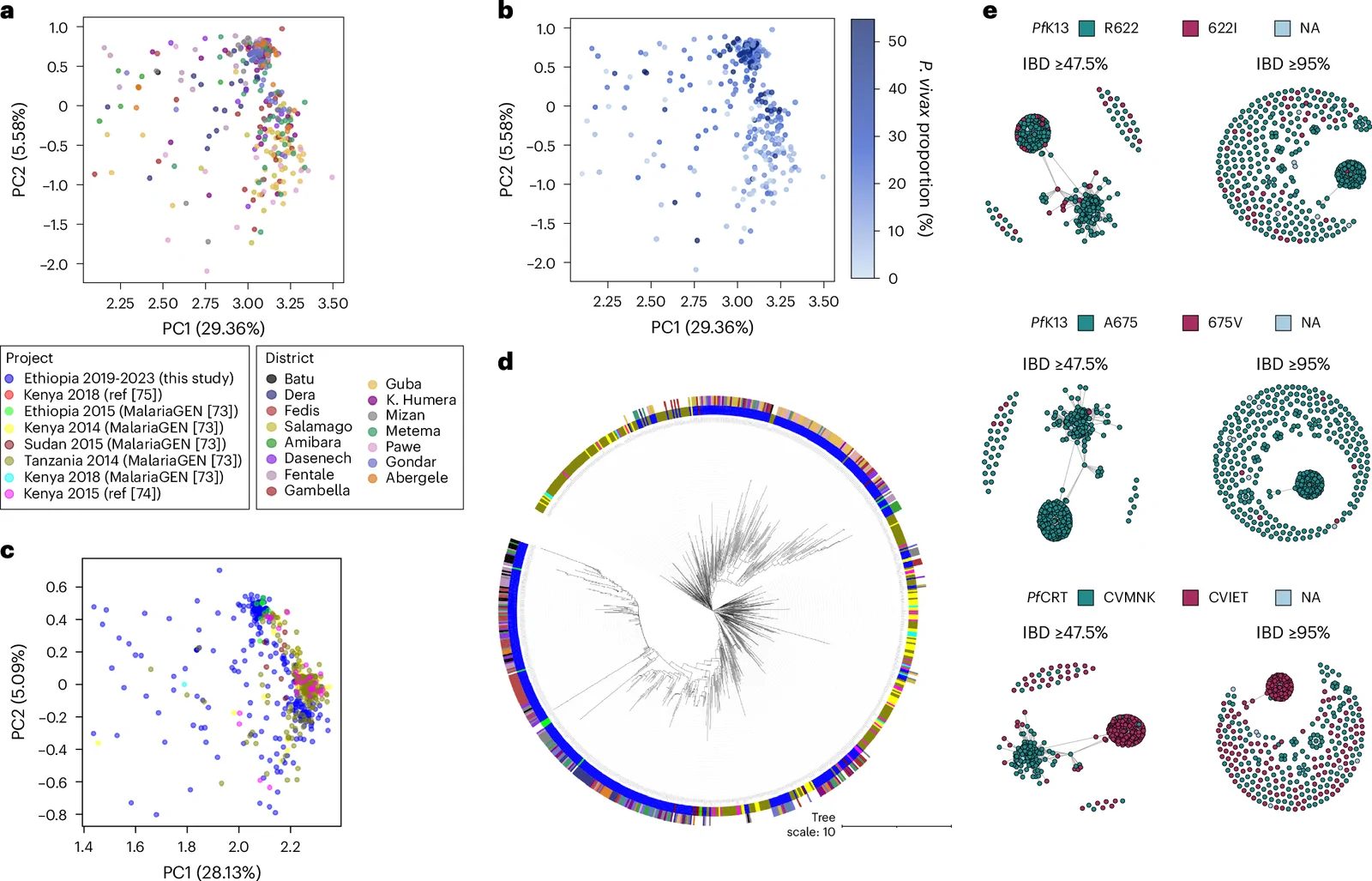
Population structure and ancestry of Ethiopian and East African *P. falciparum* isolates. **a**,**b**, PCA of the isolates collected in this study, coloured (see the legend in the ‘District’ box) by district of origin (**a**) and the proportion of *P. vivax* to *P. falciparum* infections in the corresponding district (**b**). **c**,**d**, A PCA plot (**c**) and neighbour-joining tree (**d**) of 966 *P. falciparum* isolates from Ethiopia and East Africa^73–75^, constructed using SNPs in the mitochondrial genome and drug resistance genes sequenced in this study. Dots in **c** are coloured by sequencing project (see the legend in the ‘Project’ box). Colours on the inner ring in **d** indicate the sequencing project, while the outer ring indicates the Ethiopian district for samples from the current study. **e**, IBD networks for *Pf*K13 R622I (top), *Pf*K13 A675V (middle) and *Pf*CRT CVIET (bottom) haplotypes. Each circle represents an isolate, coloured by haplotype status (mutant, wild-type or NA if there was no successful genotyping of this variant). Lines connecting circles indicate IBD sharing above the specified threshold, with IBD ≥95% considered clonal and IBD ≥47.5% considered indicative of sibling relationships. Only monoclonal infections were included in the IBD analysis. Source data

**Extended Data Fig. 8:**
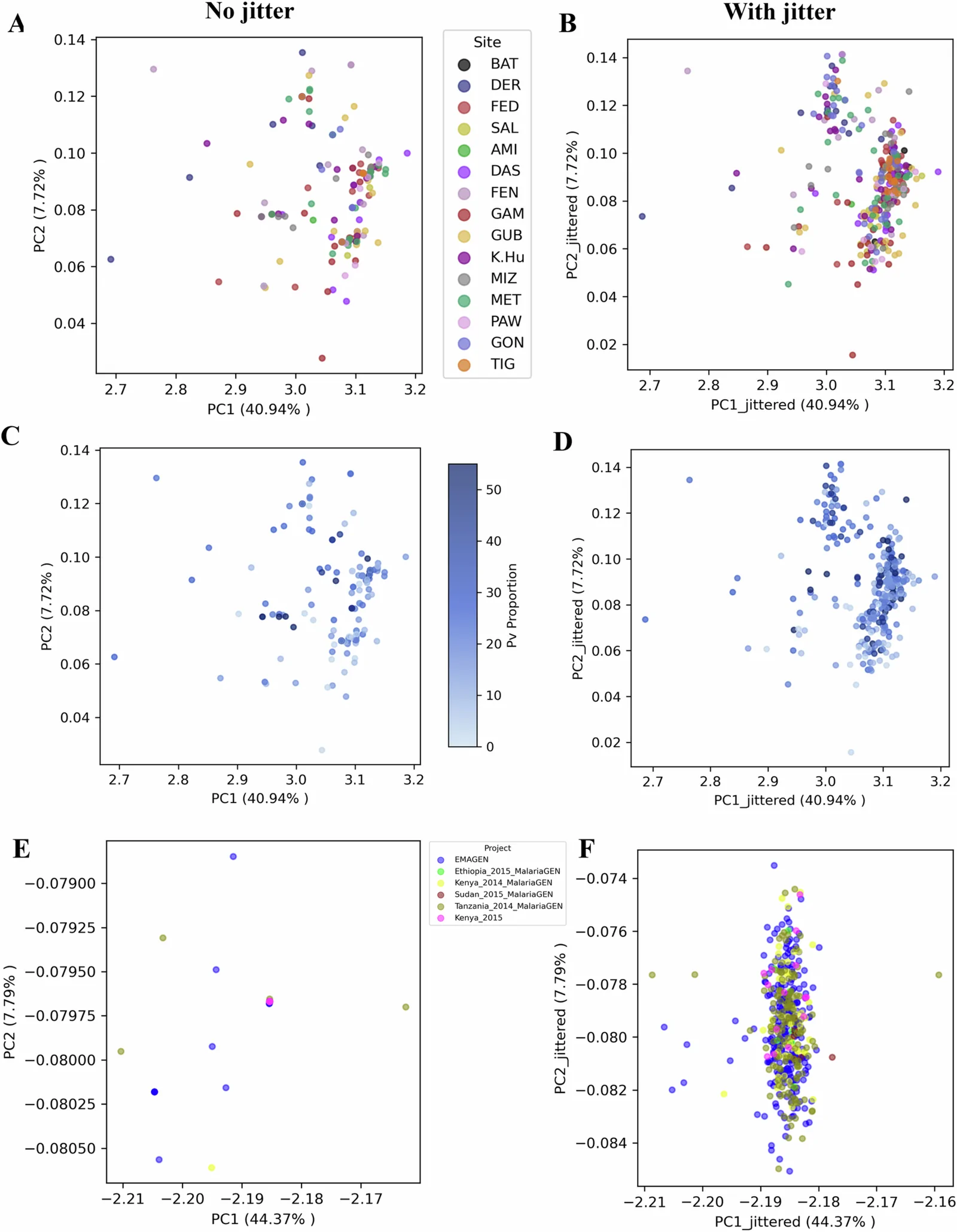
Principal Component Analysis (PCA) of mitochondrial genome. Plots include isolates collected in this study (**A**-**D**), coloured by district of origin (**A**-**B**) or the proportion of P. vivax to P. falciparum infections in the corresponding district (**C**-**D**), or 966 *P. falciparum* isolates from Ethiopia and East Africa (**E**-**F**). Plots are without jitter (**A**, **C**, **E**) and with jitter (0.002), adding a small amount of random noise to spread overlapping points slightly apart (**B**, **D**, **F**).

Population genetic analysis combining mitochondrial sequences with molecularly defined drug resistance profiles revealed two clusters of isolates using principal component analysis (PCA). These clusters aligned with geography (Fig. 4a) and *P. vivax* prevalence (Fig. 4b), suggesting that isolates from *P. vivax* co-endemic regions differ from those where *P. falciparum* predominates, consistent with certain haplotypes showing a significant association with *P. vivax* (Extended Data Fig. 6). By contrast, analysis of the mitochondrial genome alone revealed no meaningful variation and did not produce distinct clustering (Extended Data Fig. 8).

Comparison with 966 publicly available *P. falciparum* genomes from East and the Horn of Africa (Fig. 4c,d) further supported this conclusion. An SNP-based neighbour-joining tree incorporating mitochondrial genome and drug resistance loci again revealed two clusters: one containing isolates from multiple countries and years, and another dominated by Ethiopian isolates. Mitochondrial-only analysis again showed no clustering (Extended Data Fig. 8), reinforcing that the observed patterns reflect selection on resistance genes rather than underlying population structure.

### Independent emergence of *Pf*K13 variants, but distinct ancestry, among *Pf*CRT haplotypes

Ancestry analysis using identity-by-descent (IBD) of the mitochondrial genome and drug resistance genes showed that the *Pf*K13 variants R622I and A675V did not form distinct clonal clusters, but instead shared ancestry with wild-type isolates, suggesting that these mutations probably emerged independently within this population (Fig. 4e). By contrast, the *Pf*CRT CVIET haplotype appears to have originated from distinct ancestors compared with the wild-type CVMNK haplotype. However, the presence of some wild-type isolates sharing IBD segments with CVIET mutants, and vice versa, suggests potential historical or ongoing introgression between these lineages.

## Discussion

This study provides a comprehensive assessment of *P. falciparum* drug resistance across 15 Ethiopian districts, revealing a complex and heterogeneous landscape shaped by transmission intensity, species composition and historical drug use. We found widespread evidence of multidrug resistance, with a high prevalence of *PfCRT* CVIET and *PfMDR1* NFSND haplotypes driving high-level chloroquine resistance, alongside the spread of ART-R-associated *Pfk13* variants R622I and A675V. These findings underscore the dual challenge of persistent resistance to discontinued drugs and rising threats to current frontline therapies.

Despite the withdrawal of SP in 2005 and the absence of SP-based preventive treatments in Ethiopia, SP resistance mutations remain common. Their persistence is probably sustained by selective pressure from trimethoprim–sulfamethoxazole, widely used in HIV care^42^, with coverage reaching up to 80% among an estimated 610,000 people living with HIV in 2023^43^, and documented impact on malaria outcomes^44^. Additional drivers include minimal fitness costs^45,46^, enhanced mosquito infectivity^47–49^ and continued SP/CQ availability in the private sector^32^. The quintuple *Pf*DHFR–*Pf*DHPS haplotype clustered along major transport routes, probably reflecting human-mediated gene flow, while globally uncommon haplotypes were disproportionately prevalent (40.8% nationwide) in northwestern and southwestern regions with recent malaria resurgence. The dominance of the N51I + S108N *Pf*DHFR double mutant within these haplotypes supports a stepwise model of antifolate resistance evolution in which S108N arises first, followed by N51I and C59R, before fixation of the triple mutant^50,51^, highlighting the interplay of drug pressure, parasite adaptation and regional transmission.

Similarly, the high prevalence of *Pf*CRT CVIET (61.2%) reflects ongoing chloroquine pressure from *P. vivax* treatment in co-endemic areas^52^, while wild-type CVMNK persists in low *P. vivax* settings, reaching 95.1% in Guba. This geographic variation underscores the role of local drug pressure and parasite population structure. The widespread *Pf*MDR1 NFSND haplotype (93.0%), consistent with reports from Ethiopia^35,36,53^ and across Africa^54,55^, further complicates resistance dynamics.

The expansion of validated *Pfk*13 variants adds to growing evidence of ART-R in East Africa^8,13,35,36^. R622I was concentrated in the northwest, A675V in the southwest and P441L was detected at low prevalence in central districts of Ethiopia. The distinct geographic distributions mirror independent regional emergence seen in Asia and Uganda^8,56^, suggesting that local drug pressure and genetic background play key roles in shaping ART-R evolution. If ART-R spreads further, AL efficacy could be compromised, a risk heightened by heavy reliance on lumefantrine in current ACTs and next-generation combinations such as the triple ACT AL–amodiaquine and ganaplacide–lumefantrine^21,23^. Preserving lumefantrine effectiveness will require proactive strategies, including multiple first-line treatments with antagonistic resistance mechanisms, supported by timely policy and regulatory measures.

In our dataset, R622I frequently co-occurred with multiple *Pf*CRT variants (M74I, Q271E, R371I, K76T, A220S, N326S and N75E), echoing reports from Asia^16,57^ and suggesting a complex interplay between resistance mechanisms^35^. Although CVIET itself does not confer ART-R, it may create a permissive genetic background for *Pfk13* mutations^16,57,58^. These associations probably reflect overlapping drug pressures from lumefantrine and chloroquine rather than true epistasis. Fitness costs of maintaining multiple resistance alleles probably vary with transmission intensity, strain competition and immune pressures. Stratified analyses by geography, *Pf*CRT prevalence and API confirmed that co-occurrence patterns are strongly influenced by population structure, underscoring the need for whole-genome and longitudinal data to disentangle causal relationships.

*Pf*CRT variants also influence partner drug efficacy; for instance, CVIET is associated with increased lumefantrine sensitivity^57^. Resistance dynamics are further complicated by the high prevalence of *Pf*MDR1 NFSND. The very low prevalence of 86Y (2.0%) probably reflects fitness costs under AL pressure, consistent with its continued decline since AL introduction in 2004^35,36^ and in line with regional observations^35,36^. While the role of Y184F in lumefantrine and chloroquine susceptibility remains unclear, the 86Y allele has been linked to increased lumefantrine but reduced susceptibility to 4-aminoquinolines (amodiaquine and chloroquine)^59–62^, illustrating the complex interplay of drug pressures shaping resistance evolution. Whether NFSND contributes to lumefantrine resistance in areas with ongoing chloroquine use, such as Ethiopia, remains unresolved and requires further genomic and functional investigation. These uncertainties may affect decisions on which antimalarials to include in multiple first-line treatments, as shared resistance mechanisms could undermine outcomes.

In line with Ethiopia’s unique malaria ecology, we identified a distinct *P. falciparum* cluster compared with East African isolates, driven largely by molecularly defined drug resistance profiles rather than mitochondrial variation. Distinct clustering of *Pf*CRT and *Pfk*13 variants supports localized resistance evolution, probably shaped by regional drug use and transmission dynamics^63^. While our IBD analysis was restricted to drug resistance genes and the mitochondrial genome, genome-wide approaches will be needed for more robust inference of population relatedness and gene flow.

Taken together, Ethiopia’s distinct *P. falciparum* cluster, marked geographic heterogeneity in resistance markers and correlation with species composition highlight the need for region-specific control strategies. This study focused on known resistance markers in a limited set of genes, potentially overlooking novel mechanisms. The functional significance of the observed co-occurrence patterns among resistance markers remains unclear, highlighting the need for studies that establish causal relationships between these markers, ecological variables and epidemiological outcomes. Integrated surveillance systems that monitor both *P. falciparum* and *P. vivax*, coupled with whole-genome sequencing and longitudinal data linked to clinical outcomes, will be essential to track resistance evolution and guide tailored interventions.

In summary, this study provides a detailed assessment of the prevalence, distribution and co-occurrence of key *P. falciparum* drug resistance markers across diverse transmission settings in Ethiopia, amid the recent malaria resurgence. By including data from 2021, an inflection point in Ethiopia’s malaria resurgence, our study helps fill the temporal gap and may provide useful context for evolving resistance patterns. The persistence of *Pf*MDR1 NFSND and *Pf*CRT CVIET haplotypes, along with the notable emergence and spread of *Pfk13* mutations associated with ART-R, reflects overlapping drug pressures and complex parasite adaptation. Strong associations of R622I with *Pf*CRT CVIET highlight the importance of genomic surveillance in co-endemic settings. Sustaining ACT effectiveness will require region-specific control measures guided by integrated surveillance, and proactive diversification of the antimalarial arsenal to stay ahead of evolving resistance.

## Methods

### Surveillance sites and sample collection

In total, 15 sites across Ethiopia’s administrative districts were selected for genomic surveillance of antimalarial resistance (Fig. 1a). Site selection prioritized settings that reported malaria outbreaks^32,64,65^, markers of drug-resistant parasites^35^, high *pfhrp2/3* deletion rates^35^, representation of the transmission strata of the country (ranging from low and unstable to high) (Fig. 1b), varying *P. vivax* co-endemicity with *P. falciparum* (Fig. 1c) and areas prone to importation of parasites from neighbouring countries. To assess malaria transmission intensity and characterize the distribution of *P. vivax*, we utilized malaria surveillance data from the Public Health Emergency Management system. The dataset covers confirmed malaria cases at the district level in Ethiopia from 2020 to 2022. Population projections for the corresponding districts were obtained from OCHA^66^ to serve as denominators in prevalence calculations. The API was calculated as the number of total confirmed malaria cases per year divided by the population at risk, multiplied by 1,000 to express cases per 1,000 persons at risk per year. Malaria risk categories were assigned on the basis of the Ethiopian Ministry of Health stratification: malaria free (API 0), very low (0 < API ≤ 5), low (5 < AP ≤ 10), moderate (10 < API ≤ 50) and high (API >50). The proportion of *P. vivax* cases was calculated as the ratio of *P. vivax* confirmed cases to the total confirmed malaria cases within each district. These proportions were aggregated and spatially mapped to illustrate geographic variation across the country.

A total of 605 blood samples were collected from symptomatic *P. falciparum* between October 2019 and January 2023 (see Supplementary Methods for sample size determination). Finger-prick blood samples (0.5 ml) collected in EDTA microtainer tubes were used for malaria diagnosis by microscopy and to prepare dried blood spots on Whatman 3MM filter paper.

### Ethical considerations

The study protocol was approved by the Armauer Hansen Research Institute and ALERT Hospital Ethics Review Committee (PO/46/20) and the University of Notre Dame Institutional Review Board (approval no. 20-12-6350). Written informed consent was obtained from all study participants and their parents or guardians before recruitment. The study adhered to the Declaration of Helsinki principles.

### DNA extraction, *P. falciparum* species confirmation and parasite quantification

Genomic DNA was extracted from 6-mm diameter punches of dried blood spots using a MagMAX magnetic bead-based DNA multi-sample kit on a Kingfisher Flex robotic extractor machine (Thermo Fisher Scientific) following the manufacturer’s protocol and eluted in 150 µl low-salt elution buffer. Quantitative polymerase chain reaction (qPCR) targeting the *P. falciparum* 18S rRNA small subunit gene for *P. falciparum* was performed using primer and probe sequences as previously described^67^, using TaqMan Fast Advanced Master Mix (Applied Biosystems). *P. falciparum* parasites were quantified using standard curves generated from a serial dilution of NF54 ring-stage parasites (10^6^ to 10^3^ parasites ml^−1^). Samples with parasitaemia of 50 parasites μl^−1^ or higher were selected for drug resistance marker genotyping.

### Amplification of antimalarial drug resistance genes, sample and library preparation, and targeted deep-amplicon sequencing

Amplicon sequencing targeting the entire length of six antimalarial drug resistance genes (*Pfcrt*, *Pfmdr1*, *Pfk13*, *Pfdhfr* and *Pfdhps*) and the mitochondrial genome was employed as described before^68,69^, with a few modifications as detailed in the Supplementary Protocol. Single gene PCR amplification assays were modified into two multiplex PCR reactions: Multiplex-I for *Pfk13*, *Pfmdr1* and *Pfcytb* genes; and Multiplex-II for *PfMit*, *Pfcrt*, *Pfdhps* and *Pfdhfr* gene amplification. Samples were amplified and sequenced in two batches. In the second batch, *Pfcytb* was not included in the PCR; however, the *PfMit* amplicon also encompasses the *Pfcytb* gene. Normalized amplicons pooled from each patient sample were used for library preparation using the Nextera DNA Flex Library Preparation Kit (Illumina). During library preparation, PCR amplicons were tagmented with a transposome, simultaneously fragmenting the DNA and adding adaptor sequences in a single step. Seven PCR cycles were used for indexing followed by standard purification of the final libraries. Library quantification was performed using a Qubit 4.0 fluorometer with a DNA HS kit (Invitrogen), and fragment size distribution was assessed using a Bioanalyzer 2100 with a DNA HS assay kit (Agilent). All libraries were normalized to 4 nM, pooled and sequenced on a NextSeq 500/550 (Illumina) using 2 × 149-bp paired-end reads. In total, 605 *P. falciparum* isolates were successfully amplified and pooled, and sequencing data were obtained for 604 isolates.

### Sequence alignment, variant and haplotype identification

Raw Illumina paired-end reads were quality-controlled using the next-generation Sequence Analysis Toolkit (https://github.com/yyr4/Nf-NeST/). Reads with a Phred score below 20 and length below 100 bp were removed. SNPs were identified using the same pipeline, which employs an ensemble of four variant callers (Samtools, freeBayes, HaplotypeCaller and VarDict_v1.8.2) to increase the detection confidence. Known drug-resistance-associated SNPs were filtered for a minimum read coverage of 5× at the corresponding position. Novel SNPs were reported only if they were detected by at least two variant callers and were present in at least ten samples. After quality control and removal of low-depth coverage samples, 584 isolates were retained for downstream analysis. Haplotypes were constructed for *Pfcrt* (codons 72–76), *Pfmdr1* (codons 86, 184, 1034, 1,042 and 1,246), *Pfdhps* (codons 431, 436, 437, 540, 581 and 613) and *Pfdhfr* (codons 16, 51, 59, 108 and 164) using a variant allele frequency threshold of ≥95% for mutant alleles and ≤5% for wild-type alleles^70^. Samples that did not meet these criteria, which are potentially indicative of polyclonal infections, were excluded from the haplotype analysis. In the prevalence tables, *N* indicates the number of samples successfully genotyped for each specific locus. The sample size (*N*) varies between loci because prevalence was calculated using only samples with definitive genotype data for that position.

### Statistical and population genetic analyses

Field data were initially collected using paper-based forms and subsequently double-entered into RedCap (mobile application version 5.20.11). Any discrepancies identified during the data entry process were resolved by cross-referencing the original paper forms and reaching a consensus among the data managers.

Geographic variation in SNP and haplotype frequencies was assessed using Chi-squared tests (statistical significance: *P* < 0.05), and the Wilson score interval method was used to calculate 95% confidence intervals (CIs). Infections with mixed genotypes (a combination of wild-type and mutant alleles) were grouped with the mutant genotypes for frequency analysis. The median and IQR were used to summarize the age distribution across sites. Associations between haplotype frequencies, the API and the fraction of *P. vivax* and total infections were analysed using Spearman’s correlation. To adjust for small sample sizes and to achieve robust estimates, 200 bootstrap resampling iterations were used. The API was based on the total confirmed malaria cases and was calculated as follows: (total confirmed malaria cases per year ÷ population at risk) × 1,000.

The co-occurrence of WHO-reported missense mutations in *Pfcrt*, *Pfmdr1* and *Pfk13* was investigated across 584 samples. For each pairwise comparison, samples indicating mixed-genotype infections (variant allele frequency ≥5% and ≤95%) at the relevant loci were excluded. An overall independent mutation co-occurrence analysis was performed for *Pfcrt–Pfk13* (*n* = 329), *Pfmdr1–Pfk13* (*n* = 469) and *Pfcrt–Pfmdr1* (*n* = 497) gene pairs. To investigate co-occurrence patterns in different settings for sensitivity analyses, the dataset was stratified in three independent subgroup analyses on the basis of geographical location, grouping districts into three distinct regional clusters, with cluster 1 representing north/west (districts Dera, Guba, K. Humera, Metema, Pawe, Gondar and Tanqua Abergelle), cluster 2 representig the south/southwest (Dasenech, Mizan, Salamago and Gambella Zuria) and cluster 3 representing the east/south (Amibara, Batu/Zeway, Fedis and Fentale). The second stratification was based on the background prevalence of any *Pfcrt* mutation within each district. Districts were stratified into low (<10%), moderate (48–79%) and high (>85%) prevalence strata using K-means clustering (*k* = 3) based on overall frequencies of seven key *Pf*CRT variants (M74I, N75E, K76T, A220S, Q271E, N326S and R371I). The third stratification was based on API, with thresholds defining low (0–10), moderate (10–50) and high (≥50) transmission settings. Within each defined stratum, the statistical significance of co-occurrence for each mutation pair was assessed using a two-tailed Fisher’s Exact Test on 2 x 2 contingency tables, with a *P* value <0.05 considered significant.

To address the co-occurrence more directly, we undertook additional analyses restricted to districts with confirmed circulation of the *Pfk13* R622I mutation (nine sites; 302 samples, of which 297 had complete *Pfk13/Pfcrt/Pfdhfr/Pfdhps* genotyping data). Our objective was to evaluate genetic and ecological determinants of R622I occurrence while explicitly accounting for confounding by site-level factors. The primary outcome was *Pf*K13 R622I status and predictors were binary indicators for *Pf*CRT CVIET, *Pf*DHFR AICNI and *Pf*DHPS ISGEAA. To account for the strong interrelatedness of geography, transmission intensity and resistance-marker distributions, we applied mixed-effects logistic regression with a random intercept for site, jointly adjusting for standardized API and *P. vivax* proportion, to test whether associations persist after ecological control. Effects are reported as odds ratios with 95% CIs. As a sensitivity analysis, we further implemented Bayesian multilevel logistic regression with a random intercept for site using weakly informative priors, reporting posterior odds ratios and credible intervals to assess robustness under sparse outcome events. Bayesian multilevel logistic regression with a random intercept for site was performed as a sensitivity analysis using weakly informative normal (0,2.5) priors on fixed effects (log-odds scale); posterior odds rations with 95% credible intervals were used to assess the robustness of effect direction and uncertainty under sparse outcome events.

For haplotype analysis of *P. falciparum* drug resistance genes (*Pfcrt*, *Pfmdr1*, *Pfk13*, *Pfdhps* and *Pfdhfr*), full-gene amplicon sequences were aligned using MAFFT software^71^. To visualize gene-wide haplotype diversity and geographical spread, minimal-spanning networks were constructed using all observed polymorphisms within each gene. These haplotype networks were constructed using the R package pegas^72^. Publicly available *P. falciparum* genomic sequences were obtained from previous studies in East Africa and the Horn of Africa^73–75^. The variant caller GATK (version 4.1.4.1) was used to call SNPs and short indels. PCA was then conducted on SNPs (both synonymous and non-synonymous) from the multisample variant call format (VCF) using the scikit-allel Python package (version 1.3.13)^76^. A neighbour-joining tree was created using pairwise genetic distance matrices and the ape R package (version 1.3)^72^. IBD was used to assess relatedness between isolates by estimating the pairwise fraction of shared ancestry between the sequenced segments. This was done using the hmmIBD software, which applies a hidden Markov model-based approach to estimate shared ancestry while taking recombination into account^77^. This was run on a binary matrix including synonymous and non-synonymous SNPs. We applied the default parameter threshold of inbreeding coefficient >0.95 to restrict analysis to monoclonal infections. Network graphs were created using the igraph R package (version 1.3.5)^78^. Code used for the bioinformatics and statistical analyses is available via GitHub at https://github.com/AHRI-Malaria/EMAGEN.

### Reporting summary

Further information on research design is available in the Nature Portfolio Reporting Summary linked to this article.

## Supplementary information


Supplementary InformationSupplementary Tables 1–6, protocol and methods.
Reporting Summary
Peer Review File


## Source data


Source Data Fig. 1Source data for creation of maps.
Source Data Fig. 2Source data for pie charts in maps.
Source Data Fig. 3Source data for visualizations.
Source Data Fig. 4Source data for PCAs and network graphs.
Source Data Extended Data Figs. 1 and 2Source data for heat map visualizations.
Source Data Extended Data Fig. 3Source data for visualizations.
Source Data Extended Data Fig. 4Source data for pie charts.
Source Data Extended Data Fig. 6Statistical source data.
Source Data Extended Data Fig. 7Statistical source data.
Source Data Extended Data Fig./Table 6Source data underlying PCAs.


## Data Availability

Raw sequence data for the 604 sequenced isolates in this study can be found in the European Nucleotide Archives under project accession number PRJEB87415. Sample names, accession numbers and metadata are listed in Supplementary Table 6. Source data are provided with this paper.

## Extended data

**Extended Data Table 1:** Prevalence of main antimalarial drug resistance variants/haplotypes per district

Region	District	*Pfk13* R622I (n/N, %)	*Pfk13* A675V (n/N, %)	*Pfcrt* CVIET (n/N, %)	*Pfcrt* CVMNK (n/N, %)	*Pfmdr1* NFSND (n/N, %)	*Pfmdr1* NYSND (n/N, %)	*Pfdhfr* AIRNI (n/N, %)	*Pfdhfr* AICNI (n/N, %)	*Pfdhfr* ANCSI (n/N, %)	*Pfdhps* ISAKAA (n/N, %)	*Pfdhps* ISGEAA (n/N, %)
**Afar**	Amibara	(1/33, 3.0%)	(0/33, 0.0%)	(29/31, 93.5%)	(2/31, 6.5%)	(24/31, 77.4%)	(1/31, 3.2%)	(18/31, 58.1%)	(13/31, 41.9%)	(0/31, 0.0%)	(7/33, 21.2%)	(26/33, 78.8%)
**Amhara**	Dera	(10/28, 35.7%)	(0/27, 0.0%)	(21/23, 91.3%)	(2/23, 8.7%)	(22/22, 100.0%)	(0/22, 0.0%)	(1/27, 3.7%)	(26/27, 96.3%)	(0/27, 0.0%)	(2/26, 7.7%)	(24/26, 92.3%)
**Amhara**	Metema	(14/42, 33.3%)	(0/42, 0.0%)	(17/38, 44.7%)	(21/38, 55.3%)	(28/31, 90.3%)	(3/31, 9.7%)	(7/33, 21.2%)	(23/33, 69.7%)	(3/33, 9.1%)	(11/36, 30.6%)	(22/36, 61.1%)
**Benishangul**	Guba	(1/43, 2.3%)	(0/43, 0.0%)	(2/41, 4.9%)	(39/41, 95.1%)	(27/33, 81.8%)	(6/33, 18.2%)	(2/33, 6.1%)	(9/33, 27.3%)	(22/33, 66.7%)	(33/39, 84.6%)	(6/39, 15.4%)
**Benishangul**	Pawe	(2/41, 4.9%)	(2/41, 4.9%)	(2/31, 6.5%)	(29/31, 93.5%)	(25/30, 83.3%)	(5/30, 16.7%)	(5/26, 19.2%)	(5/26, 19.2%)	(16/26, 61.5%)	(31/35, 88.6%)	(3/35, 8.6%)
**Gambella**	Gambella zuria	(0/0, 0.0%)	(8/49, 16.3%)	(1/26, 3.8%)	(25/26, 96.2%)	(35/39, 89.7%)	(4/39, 10.3%)	(18/41, 43.9%)	(23/41, 56.1%)	(0/41, 0.0%)	(17/30, 56.7%)	(8/30, 26.7%)
**Gondar Zuriya**	Gondar	(17/35, 48.6%)	(0/0, 0.0%)	(18/21, 85.7%)	(3/21, 14.3%)	(29/30, 96.7%)	(1/30, 3.3%)	(2/21, 9.5%)	(19/21, 90.5%)	(0/21, 0.0%)	(2/24, 8.3%)	(22/24, 91.7%)
**Oromia**	Batu/Zeway	(0/0, 0.0%)	(0/0, 0.0%)	(36/37, 97.3%)	(1/37, 2.7%)	(40/40, 100.0%)	(0/40, 0.0%)	(34/40, 85.0%)	(1/40, 2.5%)	(0/40, 0.0%)	(1/40, 2.5%)	(39/40, 97.5%)
**Oromia**	Fedis	(0/0, 0.0%)	(0/0, 0.0%)	(44/44, 100%)	(0/44, 0.0%)	(44/44, 100.0%)	(0/44, 0.0%)	(39/43, 90.7%)	(4/43, 9.3%)	(0/43, 0.0%)	(0/45, 0.0%)	(45/45, 100.0%)
**Oromia**	Fentale	(1/42, 2.4%)	(0/44, 0.0%)	(36/39, 92.3%)	(3/39, 7.7%)	(30/31, 96.8%)	(1/31, 3.2%)	(32/42, 76.2%)	(10/42, 23.8%)	(0/42, 0.0%)	(4/43, 9.3%)	(38/43, 88.4%)
**SNNP**	Dasenech	(0/0, 0.0%)	(0/0, 0.0%)	(25/34, 73.5%)	(9/34, 26.5%)	(26/32, 81.2%)	(4/32, 12.5%)	(26/35, 74.3%)	(7/35, 20.0%)	(2/35, 5.7%)	(16/33, 48.5%)	(17/33, 51.5%)
**SNNP**	Salamago	(0/0, 0.0%)	(0/0, 0.0%)	(2/28, 7.1%)	(26/28, 92.9%)	(29/30, 96.7%)	(1/30, 3.3%)	(5/18, 27.8%)	(10/18, 55.6%)	(3/18, 16.7%)	(20/23, 87.0%)	(3/23, 13.0%)
**SW Region**	Mizan	(0/0, 0.0%)	(0/0, 0.0%)	(26/38, 68.4%)	(12/38, 31.6%)	(38/38, 100.0%)	(0/38, 0.0%)	(39/41, 95.1%)	(2/41, 4.9%)	(0/41, 0.0%)	(4/38, 10.5%)	(34/38, 89.5%)
**Tigray**	K. Humera	(7/42, 16.7%)	(0/42, 0.0%)	(27/42, 64.3%)	(15/42, 35.7%)	(33/33, 100.0%)	(0/42, 0.0%)	(14/40, 35.0%)	(26/40, 65.0%)	(0/40, 0.0%)	(6/38, 15.8%)	(26/38, 68.4%)
**Tigray**	Tanqua Abergelle	(4/19, 21.1%)	(0/19, 0.0%)	(15/19, 78.9%)	(4/19, 21.1%)	(19/19, 100.0%)	(0/19, 0.0%)	(3/19, 15.8%)	(16/19, 84.2%)	(0/19, 0.0%)	(1/19, 5.3%)	(17/19, 89.5%)
	Overall	**(57/572, 10.0%)**	**(10/572, 1.7%)**	**(301/492, 61.2%)**	**(191/492, 38.8%)**	**(449/483, 93.0%)**	**(26/483, 5.4%)**	**(194/490, 39.6%)**	**(194/490, 39.6%)**	**(46/490, 9.4%)**	**(155/502, 30.9%)**	**(330/502, 65.7%)**

**Extended Data Table 2:** Association of *PfK13* R622I with background resistance markers and site-level ecology

Marker / Variable	cOR^a^ (95% CI)	aOR (95% CI)	P value	Bayesian cOR^a^ (95% CI)	Bayesian aOR^b^ (95% CrI)	Posterior Probability^†^
*Pf*CRT CV**IET**	5.66 (1.90–16.89)^*^	3.19 (1.60–6.34)	0.001	6.24 (2.27–18.75)	3.49 (1.26–10.77)	99.35
*Pf*DHFR A**I**C**N**I	6.68 (1.76–25.37)^*^	5.16 (1.60–16.70)	0.006	7.12 (2.59–21.59)	5.45 (1.82–17.45)	99.94
*Pf*DHPS IS**GE**AA	5.55 (1.33–23.24)^*^	2.63 (0.88–7.88)	0.084	6.16 (2.08–20.80)	2.92 (0.91–10.85)	96.44
*P. vivax* proportion (standardized)^‡^	1.68 (0.97–2.91)	1.30 (0.75–2.27)	0.348		1.34 (0.61–3.15)	79.17
API (standardized)^‡^	1.75 (0.81–3.76)	1.80 (1.04–3.10)	0.036		1.80 (0.78–4.29)	93.01
Site-level variance (σ^2^)	-	0.17 (0.004–7.21)	-	-	1.02 (0.02–5.16)	-

^**a**^Crude odds ratio (cOR)

^**b**^Adjusted odds ratio (aOR)

^*^Statistically significant

^**†**^Posterior probabilities were derived from the multivariable Bayesian mixed-effects logistic regression model with a random intercept for site. Values represent the probability that the regression coefficient is greater than zero (OR > 1) on the log-odds scale.

^‡^models result included the genetic marker plus standardized API and *P. vivax* proportion, with a random intercept for site. Bayesian models used weakly informative Normal (0,2.5) priors on fixed effects (log-odds scale). Statistical significance of the frequentist analyses was assessed using a two-sided Wald test.

## Code availability

Custom scripts used for bioinformatics analysis are available via GitHub at https://github.com/AHRI-Malaria/EMAGEN. Code for the IBD analysis is available via GitHub at https://github.com/LSHTMPathogenSeqLab/malaria-hub.
