# Supplementary material for: Genomic surveillance reveals co-occurrence of Plasmodium falciparum drug resistance variants across diverse transmission settings in Ethiopia
Source: Nat Microbiol. 2026 Aug 5;11(9):2577–88. doi: 10.1038/s41564-026-02420-5 (PMC13524833; doi:10.1038/s41564-026-02420-5)
Supplement: Supplementary file 1 [file 41564_2026_2420_MOESM1_ESM.pdf]

nature microbiology

Supplementary information

https://doi.org/10.1038/s41564-026-02420-5

Genomic surveillance reveals co-occurrence of *Plasmodium falciparum* drug resistance variants across diverse transmission settings in Ethiopia

In the format provided by the authors and unedited

SUPPLEMENTARY TABLES

Supplementary Table 1. Prevalence of all known variants and haplotypes associated with antimalarial drug resistance in *P. falciparum*

Gene	Variant /Haplotype	Amibara (n/N, %, 95% CI)	Batu (n/N, %, 95% CI)	Dera (n/N, %, 95% CI)	Dasenech (n/N, %, 95% CI)	Fedis (n/N, %, 95% CI)	Fentale (n/N, %, 95% CI)	Gambella zuria (n/N, %, 95% CI)	Guba (n/N, %, 95% CI)	K. Humera (n/N, %, 95% CI)	Mizan (n/N, %, 95% CI)	Metema (n/N, %, 95% CI)	Pawe (n/N, %, 95% CI)	Salamago (n/N, %, 95% CI)	Gondar (n/N, %, 95% CI)	Tanqua Abergelle (n/N, %, 95% CI)	Overall (n/N, %, 95% CI)	Chi²	p-value
Pfdhfr	N51I	(33/33, 100.0%, 89.6%-100.0%)	(35/40, 87.5%, 73.9%-94.5%)	(27/27, 100.0%, 82.3%-99.4%)	(35/37, 94.6%, 82.3%-98.5%)	(45/45, 100.0%, 92.1%-100.0%)	(44/44, 100.0%, 92.0%-100.0%)	(48/48, 100.0%, 92.6%-100.0%)	(21/43, 48.8%, 33.9%-63.4%)	(42/42, 100.0%, 91.6%-100.0%)	(42/42, 100.0%, 91.6%-100.0%)	(39/42, 92.9%, 83.7%-98.7%)	(25/41, 61.0%, 46.1%-74.8%)	(30/33, 90.9%, 76.4%-96.9%)	(33/33, 100.0%, 86.7%-99.3%)	(19/19, 100.0%, 83.2%-100.0%)	(518/569, 91.0%, 88.6%-93.3%)	145	7.281e-24
	C59R	(20/33, 60.6%, 43.2%-76.2%)	(39/40, 97.5%, 87.6%-99.5%)	(1/27, 3.7%, 0.5%-17.7%)	(28/37, 75.7%, 59.5%-87.2%)	(41/45, 91.1%, 79.0%-96.6%)	(34/44, 77.3%, 62.3%-87.6%)	(25/48, 52.1%, 37.9%-65.8%)	(5/43, 11.6%, 5.0%-26.2%)	(15/42, 35.7%, 22.2%-50.7%)	(40/42, 95.2%, 84.1%-98.8%)	(13/42, 31.0%, 18.7%-45.2%)	(13/41, 31.7%, 19.4%-46.7%)	(17/33, 51.5%, 34.5%-67.8%)	(14/33, 42.4%, 25.7%-59.8%)	(3/19, 15.8%, 5.1%-37.8%)	(308/569, 54.1%, 50.0%-58.2%)	193.8	1.007e-33
	S108N	(33/33, 100.0%, 89.6%-100.0%)	(40/40, 100.0%, 91.2%-100.0%)	(27/27, 100.0%, 82.3%-99.4%)	(35/37, 94.6%, 82.3%-98.5%)	(45/45, 100.0%, 92.1%-100.0%)	(44/44, 100.0%, 92.0%-100.0%)	(48/48, 100.0%, 92.6%-100.0%)	(21/43, 48.8%, 33.9%-63.4%)	(41/42, 97.6%, 87.7%-99.6%)	(42/42, 100.0%, 91.6%-100.0%)	(39/42, 92.9%, 83.7%-98.7%)	(25/41, 61.0%, 46.1%-74.8%)	(30/33, 90.9%, 76.4%-96.9%)	(33/33, 100.0%, 86.7%-99.3%)	(19/19, 100.0%, 83.2%-100.0%)	(522/569, 91.7%, 89.4%-93.9%)	157.4	2.397e-26
	ANCSI(Wt)	(0/31, 0.0%, 0.0%-11.0%)	(0/40, 0.0%, 0.0%-8.8%)	(0/27, 0.0%, 0.0%-12.5%)	(2/35, 5.7%, 1.0%-18.8%)	(0/43, 0.0%, 0.0%-8.2%)	(0/42, 0.0%, 0.0%-8.4%)	(0/41, 0.0%, 0.0%-8.6%)	(22/33, 66.7%, 48.2%-81.3%)	(0/40, 0.0%, 0.0%-8.8%)	(0/41, 0.0%, 0.0%-8.6%)	(3/33, 9.1%, 2.7%-23.3%)	(16/26, 61.5%, 41.2%-78.1%)	(3/18, 16.7%, 4.9%-39.8%)	(0/21, 0.0%, 0.0%-15.5%)	(0/19, 0.0%, 0.0%-16.8%)	(46/490, 9.4%, 6.8%-12.5%)	247.8	8.079e-45
	AICNI	(13/31, 41.9%, 25.4%-59.0%)	(1/40, 2.5%, 0.1%-12.7%)	(26/27, 96.3%, 81.1%-99.4%)	(7/35, 20.0%, 9.0%-36.6%)	(4/43, 9.3%, 3.0%-22.1%)	(10/42, 23.8%, 12.7%-38.2%)	(23/41, 56.1%, 40.1%-70.6%)	(9/33, 27.3%, 13.6%-45.0%)	(26/40, 65.0%, 48.6%-78.6%)	(2/41, 4.9%, 1.0%-16.4%)	(23/33, 69.7%, 51.9%-83.5%)	(5/26, 19.2%, 7.3%-38.1%)	(10/18, 55.6%, 33.1%-75.2%)	(19/21, 90.5%, 70.3%-97.5%)	(16/19, 84.2%, 61.3%-94.0%)	(194/490, 39.6%, 35.0%-44.3%)	181.6	3.115e-31
	AN**RN**I	(0/31, 0.0%, 0.0%-11.0%)	(5/40, 12.5%, 4.9%-26.5%)	(0/27, 0.0%, 0.0%-12.5%)	(0/35, 0.0%, 0.0%-9.9%)	(0/43, 0.0%, 0.0%-8.2%)	(0/42, 0.0%, 0.0%-8.4%)	(0/41, 0.0%, 0.0%-8.6%)	(0/33, 0.0%, 0.0%-10.4%)	(0/40, 0.0%, 0.0%-8.8%)	(0/41, 0.0%, 0.0%-8.6%)	(0/33, 0.0%, 0.0%-10.4%)	(0/26, 0.0%, 0.0%-12.9%)	(0/18, 0.0%, 0.0%-17.6%)	(0/21, 0.0%, 0.0%-15.5%)	(0/19, 0.0%, 0.0%-16.8%)	(5/490, 1.0%, 0.3%-2.4%)	56.83	4.186e-07
	A**IRN**I	(18/31, 58.1%, 40.2%-74.1%)	(34/40, 85.0%, 70.4%-93.3%)	(1/27, 3.7%, 0.5%-18.3%)	(26/35, 74.3%, 57.0%-86.3%)	(39/43, 90.7%, 77.7%-96.6%)	(32/42, 76.2%, 60.6%-87.2%)	(18/41, 43.9%, 29.2%-59.6%)	(2/33, 6.1%, 1.3%-19.2%)	(14/40, 35.0%, 21.6%-49.4%)	(39/41, 95.1%, 83.9%-98.7%)	(7/33, 21.2%, 9.9%-39.2%)	(5/26, 19.2%, 7.3%-38.1%)	(5/18, 27.8%, 11.0%-50.2%)	(2/21, 9.5%, 2.4%-28.9%)	(3/19, 15.8%, 4.8%-37.1%)	(245/490, 50.0%, 45.4%-54.6%)	201.9	2.24e-35
Pfdhps	S436A	(0/33, 0.0%, 0.0%-10.4%)	(0/40, 0.0%, 0.0%-8.8%)	(0/27, 0.0%, 0.0%-12.1%)	(0/36, 0.0%, 0.0%-9.4%)	(0/45, 0.0%, 0.0%-7.9%)	(0/44, 0.0%, 0.0%-8.0%)	(0/36, 0.0%, 0.0%-7.4%)	(0/43, 0.0%, 0.0%-8.8%)	(1/40, 2.5%, 0.2%-12.3%)	(0/42, 0.0%, 0.0%-8.4%)	(0/42, 0.0%, 0.0%-8.6%)	(2/41, 4.9%, 0.7%-16.8%)	(0/32, 0.0%, 0.0%-10.4%)	(0/33, 0.0%, 0.0%-9.6%)	(0/19, 0.0%, 0.0%-16.8%)	(3/552, 0.5%, 0.1%-1.6%)	20.55	0.1137
	A437G	(26/33, 78.8%, 61.7%-89.7%)	(39/40, 97.5%, 87.6%-99.5%)	(25/27, 92.6%, 75.5%-97.4%)	(20/36, 55.6%, 38.0%-71.0%)	(45/45, 100.0%, 92.1%-100.0%)	(40/44, 90.9%, 78.4%-96.6%)	(19/36, 52.8%, 35.2%-69.1%)	(10/43, 23.3%, 12.6%-37.5%)	(34/40, 85.0%, 69.0%-93.6%)	(38/42, 90.5%, 77.7%-96.4%)	(30/41, 73.2%, 57.2%-85.1%)	(8/41, 19.5%, 10.0%-34.2%)	(12/32, 37.5%, 22.4%-53.4%)	(31/33, 93.9%, 79.8%-98.3%)	(18/19, 94.7%, 74.2%-99.2%)	(395/552, 71.6%, 67.7%-75.4%)	197.8	1.527e-34
	K540E	(26/33, 78.8%, 61.7%-89.7%)	(39/40, 97.5%, 87.6%-99.5%)	(25/27, 92.6%, 75.5%-97.4%)	(20/36, 55.6%, 38.0%-71.0%)	(45/45, 100.0%, 92.1%-100.0%)	(40/44, 90.9%, 78.4%-96.6%)	(18/35, 51.4%, 34.0%-68.0%)	(10/43, 23.3%, 12.6%-37.5%)	(34/40, 85.0%, 69.0%-93.6%)	(38/42, 90.5%, 77.7%-96.4%)	(29/42, 69.0%, 54.3%-81.1%)	(7/41, 17.1%, 8.2%-31.2%)	(12/32, 37.5%, 22.4%-53.4%)	(31/33, 93.9%, 79.8%-98.3%)	(18/19, 94.7%, 74.2%-99.2%)	(392/552, 71.0%, 67.7%-74.1%)	203.3	1.167e-35

	A581G	(0/33, 0.0%, 0.0%-10.4%)	(0/40, 0.0%, 0.0%-8.8%)	(0/27, 0.0%, 0.0%-12.1%)	(0/36, 0.0%, 0.0%-9.4%)	(0/45, 0.0%, 0.0%-7.9%)	(1/44, 2.3%, 0.1%-11.6%)	(9/36, 25.0%, 12.0%-41.5%)	(1/43, 2.3%, 0.1%-12.7%)	(6/40, 15.0%, 6.1%-28.5%)	(0/42, 0.0%, 0.0%-8.4%)	(5/41, 12.2%, 4.7%-24.9%)	(3/41, 7.3%, 2.1%-19.8%)	(0/32, 0.0%, 0.0%-10.4%)	(5/33, 15.2%, 5.5%-30.5%)	(1/19, 5.3%, 0.6%-24.3%)	(31/552, 5.6%, 3.9%-7.6%)	48.11	1.262e-05
	ISAKAA (Wt)	(7/33, 21.2%, 9.9%-39.2%)	(1/40, 2.5%, 0.1%-12.7%)	(2/26, 7.7%, 1.5%-24.3%)	(16/33, 48.5%, 31.2%-65.0%)	(0/45, 0.0%, 0.0%-7.9%)	(4/43, 9.3%, 3.0%-22.1%)	(17/30, 56.7%, 37.5%-73.2%)	(33/39, 84.6%, 70.2%-92.9%)	(6/38, 15.8%, 6.3%-30.8%)	(4/38, 10.5%, 3.6%-24.0%)	(11/36, 30.6%, 16.5%-47.6%)	(31/35, 88.6%, 73.0%-95.8%)	(20/23, 87.0%, 66.2%-95.4%)	(2/24, 8.3%, 2.0%-25.7%)	(1/19, 5.3%, 0.6%-24.3%)	(155/502, 30.9%, 26.7%-35.3%)	230.9	2.473e-41
	IS**GE**AA	(26/33, 78.8%, 61.7%-89.7%)	(39/40, 97.5%, 87.6%-99.5%)	(24/26, 92.3%, 75.6%-97.8%)	(17/33, 51.5%, 34.5%-67.8%)	(45/45, 100.0%, 92.1%-100.0%)	(38/43, 88.4%, 74.6%-95.0%)	(8/30, 26.7%, 12.0%-44.6%)	(6/39, 15.4%, 6.0%-29.6%)	(26/38, 68.4%, 51.3%-81.6%)	(34/38, 89.5%, 75.4%-96.0%)	(22/36, 61.1%, 44.3%-75.3%)	(3/35, 8.6%, 2.4%-22.0%)	(3/23, 13.0%, 4.0%-31.7%)	(22/24, 91.7%, 73.1%-97.7%)	(17/19, 89.5%, 68.4%-97.2%)	(330/502, 65.7%, 61.6%-69.7%)	230	3.827e-41
	IS**GEG**A	(0/33, 0.0%, 0.0%-10.4%)	(0/40, 0.0%, 0.0%-8.8%)	(0/26, 0.0%, 0.0%-12.9%)	(0/33, 0.0%, 0.0%-10.4%)	(0/45, 0.0%, 0.0%-7.9%)	(1/43, 2.3%, 0.1%-11.6%)	(5/30, 16.7%, 6.0%-34.0%)	(0/39, 0.0%, 0.0%-9.0%)	(6/38, 15.8%, 6.3%-30.8%)	(0/38, 0.0%, 0.0%-9.2%)	(3/36, 8.3%, 2.5%-21.7%)	(1/34, 2.9%, 0.3%-14.2%)	(0/23, 0.0%, 0.0%-14.3%)	(0/24, 0.0%, 0.0%-13.8%)	(1/19, 5.3%, 0.6%-24.3%)	(17/502, 3.4%, 2.0%-5.3%)	47.66	1.494e-05
	ANCSI/ISAKAA (Wt)	(0/31, 0.0%, 0.0-11.0)	(0/40, 0.0%, 0.0-8.8)	(0/26, 0.0%, 0.0-12.9)	(2/33, 6.1%, 1.7-19.6)	(0/43, 0.0%, 0.0-8.2)	(0/42, 0.0%, 0.0-8.4)	(0/26, 0.0%, 0.0-12.9)	(17/31, 54.8%, 37.8-70.8)	(0/36, 0.0%, 0.0-9.6)	(0/38, 0.0%, 0.0-9.2)	(3/31, 9.7%, 3.3-24.9)	(12/22, 54.5%, 34.7-73.1)	(1/15, 6.7%, 1.2-29.8)	(0/20, 0.0%, 0.0-16.1)	(0/19, 0.0%, 0.0-16.8)	(35/453, 7.7%, 5.6-10.6)	191.4	3.191e-33
	ANCSI/IS**GE**AA	(0/31, 0.0%, 0.0-11.0)	(0/40, 0.0%, 0.0-8.8)	(0/26, 0.0%, 0.0-12.9)	(0/33, 0.0%, 0.0-10.4)	(0/43, 0.0%, 0.0-8.2)	(0/42, 0.0%, 0.0-8.4)	(0/26, 0.0%, 0.0-12.9)	(3/31, 9.7%, 3.3-24.9)	(0/36, 0.0%, 0.0-9.6)	(0/38, 0.0%, 0.0-9.2)	(0/31, 0.0%, 0.0-11.0)	(1/22, 4.5%, 0.8-21.8)	(1/15, 6.7%, 1.2-29.8)	(0/20, 0.0%, 0.0-16.1)	(0/19, 0.0%, 0.0-16.8)	(5/453, 1.1%, 0.5-2.6)	31.81	0.004259
	AI**CN**I/ISAKAA	(7/31, 22.6%, 11.4-39.8)	(1/40, 2.5%, 0.4-12.9)	(2/26, 7.7%, 2.1-24.1)	(7/33, 21.2%, 10.7-37.8)	(0/43, 0.0%, 0.0-8.2)	(0/42, 0.0%, 0.0-8.4)	(9/26, 34.6%, 19.4-53.8)	(7/31, 22.6%, 11.4-39.8)	(3/36, 8.3%, 2.9-21.8)	(1/38, 2.6%, 0.5-13.5)	(5/31, 16.1%, 7.1-32.6)	(2/22, 9.1%, 2.5-27.8)	(7/15, 46.7%, 24.8-69.9)	(2/20, 10.0%, 2.8-30.1)	(1/19, 5.3%, 0.9-24.6)	(54/453, 11.9%, 9.3-15.2)	59.88	1.231e-07
	AI**CN**I/IS**GEG**A	(0/31, 0.0%, 0.0-11.0)	(0/40, 0.0%, 0.0-8.8)	(0/26, 0.0%, 0.0-12.9)	(0/33, 0.0%, 0.0-10.4)	(0/43, 0.0%, 0.0-8.2)	(1/42, 2.4%, 0.4-12.3)	(2/26, 7.7%, 2.1-24.1)	(0/31, 0.0%, 0.0-11.0)	(3/36, 8.3%, 2.9-21.8)	(0/38, 0.0%, 0.0-9.2)	(2/31, 6.5%, 1.8-20.7)	(0/22, 0.0%, 0.0-14.9)	(0/15, 0.0%, 0.0-20.4)	(0/20, 0.0%, 0.0-16.1)	(1/19, 5.3%, 0.9-24.6)	(9/453, 2.0%, 1.0-3.7)	22.11	0.07642
Pfdhfr_dhps	AI**CN**I/IS**GE**AA	(6/31, 19.4%, 9.2-36.3)	(0/40, 0.0%, 0.0-8.8)	(23/26, 88.5%, 71.0-96.0)	(0/33, 0.0%, 0.0-10.4)	(4/43, 9.3%, 3.7-21.6)	(9/42, 21.4%, 11.7-35.9)	(1/26, 3.8%, 0.7-18.9)	(2/31, 6.5%, 1.8-20.7)	(17/36, 47.2%, 32.0-63.0)	(1/38, 2.6%, 0.5-13.5)	(15/31, 48.4%, 32.0-65.2)	(2/22, 9.1%, 2.5-27.8)	(2/15, 13.3%, 3.7-37.9)	(16/20, 80.0%, 58.4-91.9)	(14/19, 73.7%, 51.2-88.2)	(112/453, 24.7%, 21.0-28.9)	188.9	9.957e-33
	AI**RN**I/IS**GE**AA	(18/31, 58.1%, 40.8-73.6)	(34/40, 85.0%, 70.9-92.9)	(1/26, 3.8%, 0.7-18.9)	(17/33, 51.5%, 35.2-67.5)	(39/43, 90.7%, 78.4-96.3)	(28/42, 66.7%, 51.6-79.0)	(6/26, 23.1%, 11.0-42.1)	(1/31, 3.2%, 0.6-16.2)	(8/36, 22.2%, 11.7-38.1)	(33/38, 86.8%, 72.7-94.2)	(4/31, 12.9%, 5.1-28.9)	(0/22, 0.0%, 0.0-14.9)	(0/15, 0.0%, 0.0-20.4)	(2/20, 10.0%, 2.8-30.1)	(3/19, 15.8%, 5.5-37.6)	(194/453, 42.8%, 38.3-47.4)	213	1.216e-37
	AN**RN**I/IS**GE**AA	(0/31, 0.0%, 0.0-11.0)	(5/40, 12.5%, 5.5-26.1)	(0/26, 0.0%, 0.0-12.9)	(0/33, 0.0%, 0.0-10.4)	(0/43, 0.0%, 0.0-8.2)	(0/42, 0.0%, 0.0-8.4)	(0/26, 0.0%, 0.0-12.9)	(0/31, 0.0%, 0.0-11.0)	(0/36, 0.0%, 0.0-9.6)	(0/38, 0.0%, 0.0-9.2)	(0/31, 0.0%, 0.0-11.0)	(0/22, 0.0%, 0.0-14.9)	(0/15, 0.0%, 0.0-20.4)	(0/20, 0.0%, 0.0-16.1)	(0/19, 0.0%, 0.0-16.8)	(5/453, 1.1%, 0.5-2.6)	52.2	2.6e-06
	AI**RN**I/ISAKAA	(0/31, 0.0%, 0.0-11.0)	(0/0, 0.0%, 0.0-8.8)	(0/26, 0.0%, 0.0-12.9)	(7/33, 21.2%, 10.7-37.8)	(0/43, 0.0%, 0.0-8.2)	(4/42, 9.5%, 3.8-22.1)	(5/26, 19.2%, 8.5-37.9)	(1/31, 3.2%, 0.6-16.2)	(2/36, 5.6%, 1.5-18.1)	(3/38, 7.9%, 2.7-20.8)	(1/31, 3.2%, 0.6-16.2)	(4/22, 18.2%, 7.3-38.5)	(4/15, 26.7%, 10.9-52.0)	(0/20, 0.0%, 0.0-16.1)	(0/19, 0.0%, 0.0-16.8)	(31/453, 6.8%, 4.9-9.5)	45.68	3.157e-05
	AI**RN**I/IS**GEG**A	(0/31, 0.0%, 0.0-11.0)	(0/40, 0.0%, 0.0-8.8)	(0/26, 0.0%, 0.0-12.9)	(0/33, 0.0%, 0.0-10.4)	(0/43, 0.0%, 0.0-8.2)	(0/42, 0.0%, 0.0-8.4)	(3/26, 11.5%, 4.0-29.0)	(0/31, 0.0%, 0.0-11.0)	(3/36, 8.3%, 2.9-21.8)	(0/38, 0.0%, 0.0-9.2)	(1/31, 3.2%, 0.6-16.2)	(1/22, 4.5%, 0.8-21.8)	(0/15, 0.0%, 0.0-20.4)	(0/20, 0.0%, 0.0-16.1)	(0/19, 0.0%, 0.0-16.8)	(8/453, 1.8%, 0.9-3.4)	30.7	0.006105

Pfcrt	M74I	(31/33, 93.9%, 79.8%-98.3%)	(38/39, 97.4%, 85.1%-99.6%)	(21/23, 91.3%, 71.3%-97.8%)	(27/36, 75.0%, 57.0%-87.7%)	(44/44, 100%, 92.0%-100%)	(38/41, 92.7%, 78.9%-97.5%)	(2/27, 7.4%, 1.8%-22.4%)	(2/40, 5.0%, 1.4%-16.5%)	(27/42, 64.3%, 49.2%-77.0%)	(28/40, 70%, 54.3%-82.2%)	(19/42, 45.2%, 31.2%-60.1%)	(5/35, 14.3%, 5.6%-29.6%)	(5/31, 16.1%, 6.9%-32.7%)	(27/30, 90.0%, 73.3%-96.6%)	(15/19, 78.9%, 56.7%-91.5%)	(329/523, 62.9%, 58.9%-66.8%)	**2.627e+02**	**6.877e-48**
	N75E	(31/33, 93.9%, 79.8%-98.3%)	(38/39, 97.4%, 85.1%-99.6%)	(21/23, 91.3%, 71.3%-97.8%)	(27/36, 75%, 57.0%-87.7%)	(44/44, 100%, 92.0%-100%)	(38/41, 92.7%, 78.9%-97.5%)	(2/27, 7.4%, 1.8%-22.4%)	(2/41, 4.9%, 1.3%-16.1%)	(27/42, 64.3%, 49.2%-77.0%)	(28/40, 70%, 54.3%-82.2%)	(19/42, 45.2%, 31.2%-60.1%)	(5/35, 14.3%, 5.6%-29.6%)	(5/31, 16.1%, 6.9%-32.7%)	(25/30, 83.3%, 65.6%-93.1%)	(15/19, 78.9%, 56.7%-91.5%)	(327/523, 62.5%, 58.5%-66.4%)	**2.575e+02**	**8.138e-47**
	K76T	(31/33, 93.9%, 79.8%-98.3%)	(38/39, 97.4%, 85.1%-99.6%)	(21/23, 91.3%, 71.3%-97.8%)	(27/36, 75.0%, 57.0%-87.7%)	(44/44, 100%, 92.0%-100%)	(38/41, 92.7%, 78.9%-97.5%)	(2/27, 7.4%, 1.8%-22.4%)	(2/41, 4.9%, 1.3%-16.1%)	(27/42, 64.3%, 49.2%-77.0%)	(28/40, 70%, 54.3%-82.2%)	(21/42, 50%, 35.5%-64.5%)	(6/35, 17.1%, 7.1%-32.3%)	(5/31, 16.1%, 6.9%-32.7%)	(27/30, 90%, 73.3%-96.6%)	(15/19, 78.9%, 56.7%-91.5%)	(332/523, 63.5%, 59.5%-67.4%)	**2.576e+02**	**7.720e-47**
	A220S	(31/33, 93.9%, 79.8%-98.3%)	(39/40, 97.5%, 87.1%-99.6%)	(21/23, 91.3%, 71.3%-97.8%)	(26/34, 76.5%, 59.6%-88.0%)	(44/45, 97.8%, 88.4%-99.6%)	(37/40, 92.5%, 79.3%-97.4%)	(2/27, 7.4%, 1.8%-22.4%)	(2/40, 5.0%, 1.4%-16.5%)	(27/42, 64.3%, 49.2%-77.0%)	(28/40, 70%, 54.3%-82.2%)	(23/42, 54.8%, 40.2%-68.9%)	(6/34, 17.1%, 7.1%-32.3%)	(6/31, 19.4%, 8.6%-36.3%)	(28/30, 93.3%, 77.1%-98.2%)	(15/19, 78.9%, 56.7%-91.5%)	(335/520, 64.4%, 60.4%-68.2%)	**2.556e+02**	**2.023e-46**
	Q271E	(31/33, 93.9%, 79.8%-98.3%)	(39/40, 97.5%, 87.1%-99.6%)	(22/24, 87.5%, 67.8%-95.4%)	(27/36, 75%, 57.0%-87.7%)	(43/43, 100%, 91.7%-100%)	(38/41, 92.7%, 78.9%-97.5%)	(2/27, 7.4%, 1.8%-22.4%)	(2/41, 4.9%, 1.3%-16.1%)	(27/42, 64.3%, 49.2%-77.0%)	(28/40, 70%, 54.3%-82.2%)	(22/42, 52.4%, 37.7%-66.8%)	(6/34, 17.6%, 7.5%-32.9%)	(6/31, 19.4%, 8.6%-36.3%)	(28/30, 93.3%, 77.1%-98.2%)	(15/19, 78.9%, 56.7%-91.5%)	(336/523, 64.3%, 60.3%-68.1%)	**2.562e+02**	**1.527e-46**
	N326S	(31/33, 93.9%, 79.8%-98.3%)	(37/39, 94.9%, 81.8%-98.6%)	(20/22, 90.9%, 70.7%-97.5%)	(21/35, 60%, 43.8%-74.1%)	(43/43, 100%, 91.7%-100%)	(36/39, 92.3%, 79.7%-97.3%)	(1/27, 3.7%, 0.7%-18.3%)	(1/40, 2.5%, 0.4%-12.9%)	(25/42, 59.5%, 44.5%-73.0%)	(27/40, 67.5%, 51.8%-80.2%)	(22/41, 53.7%, 38.7%-67.9%)	(2/34, 5.9%, 1.6%-17.3%)	(1/31, 3.2%, 0.6%-16.2%)	(28/30, 93.3%, 77.1%-98.2%)	(15/19, 78.9%, 56.7%-91.5%)	(310/515, 60.2%, 56.1%-64.1%)	**2.827e+02**	**4.781e-52**
	I356T	(0/33, 0.0%, 0.0%-10.4%)	(0/39, 0.0%, 0.0%-8.9%)	(0/23, 0.0%, 0.0%-15.1%)	(6/36, 16.7%, 7.4%-32.2%)	(0/44, 0.0%, 0.0%-8.0%)	(0/41, 0.0%, 0.0%-8.6%)	(0/27, 0.0%, 0.0%-12.8%)	(0/41, 0.0%, 0.0%-8.6%)	(0/42, 0.0%, 0.0%-8.4%)	(0/40, 0.0%, 0.0%-8.8%)	(1/42, 2.4%, 0.4%-12.6%)	(1/35, 2.9%, 0.5%-14.2%)	(1/31, 3.2%, 0.6%-16.2%)	(0/30, 0.0%, 0.0%-11.5%)	(0/19, 0.0%, 0.0%-17.6%)	(9/524, 1.7%, 0.9%-2.9%)	**5.510e+01**	**8.334e-07**
	R371I	(31/33, 93.9%, 79.8%-98.3%)	(38/39, 97.4%, 85.1%-99.6%)	(19/21, 85.7%, 63.3%-95.3%)	(27/36, 75.0%, 57.0%-87.7%)	(43/43, 100%, 91.7%-100%)	(39/42, 92.9%, 80.0%-97.6%)	(2/27, 7.4%, 1.8%-22.4%)	(2/41, 4.9%, 1.3%-16.1%)	(27/42, 64.3%, 49.2%-77.0%)	(28/40, 70%, 54.3%-82.2%)	(23/41, 56.1%, 41.0%-70.1%)	(6/35, 17.1%, 7.1%-32.3%)	(7/31, 22.6%, 10.9%-40.1%)	(27/29, 93.1%, 76.2%-98.3%)	(15/19, 78.9%, 56.7%-91.5%)	(334/519, 64.4%, 60.4%-68.2%)	**2.509e+02**	**1.884e-45**
	CVMNK(Wt)	(2/31, 6.5%, 1.8%-20.7%)	(1/37, 2.7%, 0.5%-13.8%)	(2/23, 8.7%, 2.4%-26.8%)	(9/34, 26.5%, 14.6%-43.1%)	(0/44, 0.0%, 0.0%-8.0%)	(3/39, 7.7%, 2.7%-20.3%)	(25/26, 96.2%, 81.1%-99.3%)	(39/41, 95.1%, 83.9%-98.7%)	(15/42, 35.7%, 23.0%-50.8%)	(12/38, 31.6%, 19.1%-47.5%)	(21/38, 55.3%, 39.7%-69.9%)	(29/31, 93.5%, 79.3%-98.2%)	(26/28, 92.9%, 77.4%-98.0%)	(3/21, 14.3%, 5.0%-34.6%)	(4/19, 21.1%, 8.5%-43.3%)	(191/492, 38.8%, 34.6%-43.2%)	266.2	1.263e-48
	CV**IET**	(29/31, 93.5%, 79.3%-98.2%)	(36/37, 97.3%, 86.2%-99.5%)	(21/23, 91.3%, 73.2%-97.6%)	(25/34, 73.5%, 56.9%-85.4%)	(44/44, 100%, 92.0%-100%)	(36/39, 92.3%, 79.7%-97.3%)	(1/26, 3.8%, 0.7%-18.9%)	(2/41, 4.9%, 1.3%-16.1%)	(27/42, 64.3%, 49.2%-77.0%)	(26/38, 68.4%, 52.5%-80.9%)	(17/38, 44.7%, 30.1%-60.3%)	(2/31, 6.5%, 1.8%-20.7%)	(2/28, 7.1%, 2.0%-22.6%)	(18/21, 85.7%, 65.4%-95.0%)	(15/19, 78.9%, 56.7%-91.5%)	(301/492, 61.2%, 56.8%-65.4%)	266.2	1.263e-48
Pfmdr1	N86	(26/33, 78.8%, 62.2%-89.3%)	(40/40, 100.0%, 91.2%-100.0%)	(27/28, 96.4%, 82.3%-99.4%)	(31/33, 93.9%, 79.8%-98.3%)	(45/45, 100.0%, 92.1%-100.0%)	(34/34, 100.0%, 89.8%-100.0%)	(46/47, 97.9%, 88.9%-99.6%)	(43/43, 100.0%, 91.8%-100.0%)	(36/36 100%, 90.4%-100%)	(42/42, 100.0%, 91.6%-100.0%)	(35/35, 100.0%, 90.2%-100.0%)	(41/41, 100.0%, 91.4%-100.0%)	(34/34, 100.0%, 89.8%-100.0%)	(32/32, 100.0%, 89.3%-100.0%)	(19/19, 100.0%, 83.2%-100.0%)	(531/542, 98.0%, 96.9%-98.8%)	**7.241e+01**	**7.034e-10**
	N86Y	(7/33, 21.2%, 9.9%-39.2%)	(0/40, 0.0%, 0.0%-8.8%)	(1/28, 3.6%, 0.6%-17.7%)	(2/33, 6.1%, 0.8%-20.2%)	(0/45, 0.0%, 0.0%-7.9%)	(0/34, 0.0%, 0.0%-10.3%)	(1/47, 2.1%, 0.4%-10.9%)	(0/43, 0.0%, 0.0%-8.2%)	(0/36, 0.0%, 0.0%-9.7%)	(0/42, 0.0%, 0.0%-8.4%)	(0/35, 0.0%, 0.0%-10.0%)	(0/41, 0.0%, 0.0%-8.8%)	(0/34, 0.0%, 0.0%-10.4%)	(0/32, 0.0%, 0.0%-11.0%)	(0/19, 0.0%, 0.0%-17.6%)	(11/542, 2.0%, 1.1%-3.4%)	7.241e+01	7.034e-10
	Y184	(8/33, 24.2%, 12.8%-41.0%)	(0/40, 0.0%, 0.0%-8.8%)	(0/28, 0.0%, 0.0%-12.1%)	(4/33, 12.1%, 4.8%-27.4%)	(0/45, 0.0%, 0.0%-7.9%)	(1/33, 3.0%, 0.5%-15.3%)	(4/47, 8.5%, 3.2%-20.3%)	(6/43, 14.0%, 6.6%-27.3%)	(0/35, 0.0%, 0.0%-10.0%)	(0/42, 0.0%, 0.0%-8.4%)	(5/36, 13.9%, 5.9%-29.4%)	(5/41, 12.2%, 5.3%-25.5%)	(1/33, 3.0%, 0.5%-15.3%)	(1/30, 3.3%, 0.6%-16.7%)	(0/19, 0.0%, 0.0%-17.6%)	(35/538, 6.5%, 4.7%-8.9%)	4.477e+01	4.445e-05

	Y184F	(25/33, 75.8%, 59.0%-87.2%)	(40/40, 100.0%, 91.2%-100.0%)	(28/28, 100.0%, 87.9%-100.0%)	(29/33, 87.9%, 72.6%-95.1%)	(45/45, 100.0%, 92.1%-100.0%)	(32/33, 97.0%, 82.8%-99.5%)	(43/47, 91.5%, 79.6%-96.6%)	(37/43, 86.0%, 72.1%-93.8%)	(35/35, 100.0%, 90.2%-100.0%)	(42/42, 100.0%, 91.6%-100.0%)	(31/36, 86.1%, 71.0%-93.9%)	(36/41, 87.8%, 74.5%-94.6%)	(32/33, 97.0%, 84.2%-99.5%)	(29/30, 96.7%, 80.5%-99.5%)	(19/19, 100.0%, 83.2%-100.0%)	(503/538, 93.5%, 91.0%-95.4%)	**4.477e+01**	**4.445e-05**
	S1034	(33/33, 100.0%, 89.6-100.0)	(40/40, 100.0%, 91.2-100.0)	(28/28, 100.0%, 87.9-100.0)	(33/33, 100.0%, 89.6-100.0)	(44/44, 100.0%, 92.0-100.0)	(33/33, 100.0%, 89.6-100.0)	(47/47, 100.0%, 92.4-100.0)	(43/43, 100.0%, 91.8-100.0)	(36/36, 100.0%, 90.4-100.0)	(41/41, 100.0%, 91.4-100.0)	(33/33, 100.0%, 89.6-100.0)	(41/41, 100.0%, 91.4-100.0)	(33/33, 100.0%, 89.6-100.0)	(31/31, 100.0%, 89.0-100.0)	(19/19, 100.0%, 83.2-100.0)	(535/535, 100.0%, 99.3-100.0)	**N/A**	**N/A**
	N1042	(33/33, 100.0%, 89.6-100.0)	(40/40, 100.0%, 91.2-100.0)	(28/28, 100.0%, 87.9-100.0)	(33/33, 100.0%, 89.6-100.0)	(43/43, 100.0%, 91.8-100.0)	(33/33, 100.0%, 89.6-100.0)	(47/47, 100.0%, 92.4-100.0)	(43/43, 100.0%, 91.8-100.0)	(36/36, 100.0%, 90.4-100.0)	(41/41, 100.0%, 91.4-100.0)	(31/31, 100.0%, 89.0-100.0)	(41/41, 100.0%, 91.4-100.0)	(33/33, 100.0%, 89.6-100.0)	(30/30, 100.0%, 88.6-100.0)	(19/19, 100.0%, 83.2-100.0)	(531/531, 100.0%, 99.3-100.0)	**N/A**	**N/A**
	D1246	(33/33, 100.0%, 89.6-100.0)	(40/40, 100.0%, 91.2-100.0)	(28/28, 100.0%, 87.9-100.0)	(34/34, 100.0%, 89.8-100.0)	(45/45, 100.0%, 92.1-100.0)	(33/33, 100.0%, 89.6-100.0)	(47/47, 100.0%, 92.4-100.0)	(43/43, 100.0%, 91.8-100.0)	(35/35, 100.0%, 90.1-100.0)	(41/41, 100.0%, 91.4-100.0)	(33/33, 100.0%, 89.6-100.0)	(41/41, 100.0%, 91.4-100.0)	(33/33, 100.0%, 89.6-100.0)	(31/31, 100.0%, 89.0-100.0)	(19/19, 100.0%, 83.2-100.0)	(536/536, 100.0%, 99.3-100.0)	**N/A**	**N/A**
	NYSND (Wt)	(1/31, 3.2%, 0.6-16.2)	(0/40, 0.0%, 0.0-8.8)	(0/22, 0.0%, 0.0-14.9)	(4/32, 12.5%, 5.0-28.1)	(0/44, 0.0%, 0.0-8.0)	(1/31, 3.2%, 0.6-16.2)	(4/39, 10.3%, 4.1-23.6)	(6/33, 18.2%, 8.6-34.4)	(0/33, 0.0%, 0.0-10.4)	(0/38, 0.0%, 0.0-9.2)	(3/31, 9.7%, 3.3-24.9)	(5/30, 16.7%, 7.3-33.6)	(1/30, 3.3%, 0.6-16.7)	(1/30, 3.3%, 0.6-16.7)	(0/19, 0.0%, 0.0-16.8)	(26/483, 5.4%, 3.7-7.8)	36.45	0.0008936
	**Y**FSND	(0/31, 0.0%, 0.0-11.0)	(0/40, 0.0%, 0.0-8.8)	(0/22, 0.0%, 0.0-14.9)	(2/32, 6.2%, 1.7-20.1)	(0/44, 0.0%, 0.0-8.0)	(0/31, 0.0%, 0.0-11.0)	(0/39, 0.0%, 0.0-9.0)	(0/33, 0.0%, 0.0-10.4)	(0/33, 0.0%, 0.0-10.4)	(0/38, 0.0%, 0.0-9.2)	(0/31, 0.0%, 0.0-11.0)	(0/30, 0.0%, 0.0-11.4)	(0/30, 0.0%, 0.0-11.4)	(0/30, 0.0%, 0.0-11.4)	(0/19, 0.0%, 0.0-16.8)	(2/483, 0.4%, 0.1-1.5)	28.3	0.01296
	NFSND	(24/31, 77.4%, 60.2-88.6)	(40/40, 100.0%, 91.2-100.0)	(22/22, 100.0%, 85.1-100.0)	(26/32, 81.2%, 64.7-91.1)	(44/44, 100.0%, 92.0-100.0)	(30/31, 96.8%, 83.8-99.4)	(35/39, 89.7%, 76.4-95.9)	(27/33, 81.8%, 65.6-91.4)	(33/33, 100.0%, 89.6-100.0)	(38/38, 100.0%, 90.8-100.0)	(28/31, 90.3%, 75.1-96.7)	(25/30, 83.3%, 66.4-92.7)	(29/30, 96.7%, 83.3-99.4)	(29/30, 96.7%, 83.3-99.4)	(19/19, 100.0%, 83.2-100.0)	(449/483, 93.0%, 90.3-94.9)	46.4	2.416e-05
	**Y**YSND	(6/31, 19.4%, 9.2-36.3)	(0/40, 0.0%, 0.0-8.8)	(0/22, 0.0%, 0.0-14.9)	(0/32, 0.0%, 0.0-10.7)	(0/44, 0.0%, 0.0-8.0)	(0/31, 0.0%, 0.0-11.0)	(0/39, 0.0%, 0.0-9.0)	(0/33, 0.0%, 0.0-10.4)	(0/33, 0.0%, 0.0-10.4)	(0/38, 0.0%, 0.0-9.2)	(0/31, 0.0%, 0.0-11.0)	(0/30, 0.0%, 0.0-11.4)	(0/30, 0.0%, 0.0-11.4)	(0/30, 0.0%, 0.0-11.4)	(0/19, 0.0%, 0.0-16.8)	(6/483, 1.2%, 0.6-2.7)	88.58	7.02e-13
Pfk13	P441L	(0/33, 0.0%, 0.0%-10.4%)	(5/40, 12.5%, 5.0%-26.4%)	(0/28, 0.0%, 0.0%-12.1%)	(0/37, 0.0%, 0.0%-9.4%)	(0/45, 0.0%, 0.0%-7.9%)	(0/41, 0.0%, 0.0%-8.0%)	(0/49, 0.0%, 0.0%-7.4%)	(0/43, 0.0%, 0.0%-8.8%)	(0/42, 0.0%, 0.0%-8.4%)	(0/42, 0.0%, 0.0%-8.4%)	(0/42, 0.0%, 0.0%-8.6%)	(0/41, 0.0%, 0.0%-8.8%)	(1/34, 2.9%, 0.2%-15.1%)	(0/35, 0.0%, 0.0%-9.6%)	(0/19, 0.0%, 0.0%-16.8%)	(6/571, 1.1%, 0.4%-2.2%)	56.63	4.527e-07
	A578S	(0/33, 0.0%, 0.0-10.4)	(0/40, 0.0%, 0.0-8.8)	(0/28, 0.0%, 0.0-12.1)	(0/37, 0.0%, 0.0-9.4)	(0/45, 0.0%, 0.0-7.9)	(0/42, 0.0%, 0.0-8.4)	(0/49, 0.0%, 0.0-7.3)	(0/43, 0.0%, 0.0-8.2)	(0/42, 0.0%, 0.0-8.4)	(0/42, 0.0%, 0.0-8.4)	(1/42, 2.4%, 0.1%-12.6%)	(0/41, 0.0%, 0.0-8.6)	(0/34, 0.0%, 0.0-10.2)	(0/35, 0.0%, 0.0-9.9)	(0/19, 0.0%, 0.0-16.8)	(1/572, 0.2%, 0.0%-1.0%)	12.88	0.5363
	R622I	(1/33, 3.0%, 0.2%-15.1%)	(0/40, 0.0%, 0.0%-8.8%)	(10/28, 35.7%, 20.0%-54.7%)	(0/37, 0.0%, 0.0%-9.4%)	(0/45, 0.0%, 0.0%-7.9%)	(1/42, 2.4%, 0.1%-11.8%)	(0/49, 0.0%, 0.0%-7.4%)	(1/43, 2.3%, 0.1%-12.7%)	(7/42, 16.7%, 7.4%-31.2%)	(0/42, 0.0%, 0.0%-8.4%)	(14/42, 33.3%, 19.9%-49.0%)	(2/41, 4.9%, 0.7%-16.8%)	(0/34, 0.0%, 0.0%-10.4%)	(17/35, 48.6%, 30.8%-65.2%)	(4/19, 21.1%, 7.1%-43.2%)	(57/572, 10.0%, 7.7%-12.9%)	142.3	2.482e-23
	A675V	(0/33, 0.0%, 0.0%-10.4%)	(0/40, 0.0%, 0.0%-8.8%)	(0/28, 0.0%, 0.0%-12.1%)	(0/37, 0.0%, 0.0%-9.4%)	(0/45, 0.0%, 0.0%-7.9%)	(0/42, 0.0%, 0.0%-8.0%)	(8/49, 16.3%, 7.6%-29.8%)	(0/43, 0.0%, 0.0%-8.8%)	(0/42, 0.0%, 0.0%-8.4%)	(0/42, 0.0%, 0.0%-8.4%)	(0/42, 0.0%, 0.0%-8.6%)	(2/41, 4.9%, 0.7%-16.8%)	(0/34, 0.0%, 0.0%-10.4%)	(0/35, 0.0%, 0.0%-9.6%)	(0/19, 0.0%, 0.0%-16.8%)	(10/572, 1.7%, 0.9%-3.1%)	72.69	6.253e-10

Geographic variation in SNP and haplotype frequencies was assessed using chi-squared tests (statistical significance: $p < 0.05$). N indicates the number of samples successfully genotyped for each marker. The Overall N is the aggregate count across all regions. This value may differ by marker, as samples without genotype data at a specific locus are excluded from the prevalence calculation for that marker. Wt : Wildtype

Supplementary Table 2. List of all the *Pf*DHFR and *Pf*DHPS haplotype combinations observed in this study.

Category - Standard convention	Haplotype	*Pf*DHFR haplotype (codons 16, 51, 59, 108, 164)	*Pf*DHPS haplotype (codons 431, 436, 437, 540, 581, 613)	Number of mutations	Resistance Phenotype	Typical distribution	References
Common	Wildtype	$(A_{16}N_{51}C_{59}S_{108}I_{164})$	$(I_{431}S_{436}A_{437}K_{540}A_{581}A_{613})$	0	Fully sensitive	Historical, pre-SP era	[1]
	Triple	$(A_{16}\mathbf{I_{51}R_{59}N_{108}}I_{164})$	$(I_{431}S_{436}A_{437}K_{540}A_{581}A_{613})$	3	High pyrimethamine resistance	Common across Africa	[2]
	Quintuple	$(A_{16}\mathbf{I_{51}R_{59}N_{108}}I_{164})$	$(I_{431}S_{436}\mathbf{G_{437}E_{540}}A_{581}A_{613})$	5	High-level SP resistance/fully resistant haplotype	Widely prevalent in East Africa (Ethiopia, Uganda, Kenya, Tanzania)	[3–6]
	Sextuple	$(A_{16}\mathbf{I_{51}R_{59}N_{108}}I_{164})$	$(I_{431}S_{436}\mathbf{G_{437}E_{540}G_{581}}A_{613})$	6	Very high resistance (SP failure likely)	Increasing in East Africa, especially Uganda, Rwanda and Tanzania	[6,7]
Uncommon	A**I**C**N**I/ISAKAA	$(A_{16}\mathbf{I_{51}}C_{59}\mathbf{N_{108}}I_{164})$	$(I_{431}S_{436}A_{437}K_{540}A_{581}A_{613})$	2	The N51**I**+S108**N** double mutant is a well-recognised *Pf*DHFR haplotype, though it is usually an intermediate form that appears on the evolutionary path towards the more common triple mutant (N51**I**+C59**R**+S108**N** = A**IRN**I). Studies from East Africa (e.g. Tanzania, Kenya, Uganda) have documented double mutants including N51**I**+S108**N**, but their frequency tends to be much lower than the triple mutant.		[8]
	A**I**C**N**I/IS**GE**AA	$(A_{16}\mathbf{I_{51}}C_{59}\mathbf{N_{108}}I_{164})$	$(I_{431}S_{436}\mathbf{G_{437}E_{540}}A_{581}A_{613})$	4			
	A**I**C**N**I/IS**GEG**A	$(A_{16}\mathbf{I_{51}}C_{59}\mathbf{N_{108}}I_{164})$	$(I_{431}S_{436}\mathbf{G_{437}E_{540}G_{581}}A_{613})$	5			
	AN**RN**I/IS**GE**AA	$(A_{16}N_{51}\mathbf{R_{59}N_{108}}I_{164})$	$(I_{431}S_{436}\mathbf{G_{437}E_{540}}A_{581}A_{613})$	4			
	ANCSI/IS**GE**AA	$(A_{16}N_{51}C_{59}S_{108}I_{164})$	$(I_{431}S_{436}\mathbf{G_{437}E_{540}}A_{581}A_{613})$	2			

Supplementary Table 3. Novel missense mutations

Gene	Variant	Amibara (n/N, %, 95% CI)	Batu/Zeway (n/N, %, 95% CI)	Dasenech (n/N, %, 95% CI)	Dera (n/N, %, 95% CI)	Fedis (n/N, %, 95% CI)	Fentale (n/N, %, 95% CI)	Gambella zuria (n/N, %, 95% CI)	Gondar (n/N, %, 95% CI)	Guba (n/N, %, 95% CI)	K. Humera (n/N, %, 95% CI)	Metema (n/N, %, 95% CI)	Mizan (n/N, %, 95% CI)	Pawe (n/N, %, 95% CI)	Salamago (n/N, %, 95% CI)	T. Abergelle (n/N, %, 95% CI)	Overall (n/N, %, 95% CI)	ChiX^2	p-value	Significance
Pfmdr1	N649D	0/33 (0.0%, 0.0-10.4)	0/40 (0.0%, 0.0-8.8)	0/34 (0.0%, 0.0-10.2)	1/28 (3.6%, 0.6-17.7)	0/45 (0.0%, 0.0-7.9)	0/36 (0.0%, 0.0-9.6)	0/48 (0.0%, 0.0-7.4)	0/41 (0.0%, 0.0-8.6)	3/43 (7.0%, 2.4-18.6)	2/36 (5.6%, 1.5-18.1)	1/36 (2.8%, 0.5-14.2)	0/42 (0.0%, 0.0-8.4)	2/41 (4.9%, 1.3-16.1)	1/34 (2.9%, 0.5-14.9)	1/19 (5.3%, 0.9-24.6)	11/556 (2.0%, 1.1-3.5)	17.84	0.214	ns
	D650N	0/33 (0.0%, 0.0-10.4)	0/40 (0.0%, 0.0-8.8)	3/34 (8.8%, 3.0-23.0)	1/28 (3.6%, 0.6-17.7)	0/45 (0.0%, 0.0-7.9)	0/36 (0.0%, 0.0-9.6)	9/48 (18.8%, 10.2-31.9)	0/41 (0.0%, 0.0-8.6)	2/43 (4.7%, 1.3-15.5)	0/36 (0.0%, 0.0-9.6)	1/36 (2.8%, 0.5-14.2)	1/42 (2.4%, 0.4-12.3)	4/41 (9.8%, 3.9-22.5)	0/34 (0.0%, 0.0-10.2)	0/19 (0.0%, 0.0-16.8)	21/556 (3.8%, 2.5-5.7)	47.59	1.54E-05	***
	N652D	2/33 (6.1%, 1.7-19.6)	16/40 (40.0%, 26.3-55.4)	0/34 (0.0%, 0.0-10.2)	5/28 (17.9%, 7.9-35.6)	3/45 (6.7%, 2.3-17.9)	5/36 (13.9%, 6.1-28.7)	1/48 (2.1%, 0.4-10.9)	5/41 (12.2%, 5.3-25.5)	0/43 (0.0%, 0.0-8.2)	1/36 (2.8%, 0.5-14.2)	1/36 (2.8%, 0.5-14.2)	0/42 (0.0%, 0.0-8.4)	1/41 (2.4%, 0.4-12.6)	4/34 (11.8%, 4.7-26.6)	4/19 (21.1%, 8.5-43.3)	48/556 (8.6%, 6.6-11.3)	78.45	5.48E-11	***
	S983N	0/33 (0.0%, 0.0-10.4)	0/40 (0.0%, 0.0-8.8)	2/34 (5.9%, 1.6-19.1)	7/28 (25.0%, 12.7-43.4)	0/45 (0.0%, 0.0-7.9)	0/36 (0.0%, 0.0-9.6)	0/48 (0.0%, 0.0-7.4)	0/41 (0.0%, 0.0-8.6)	2/43 (4.7%, 1.3-15.5)	0/36 (0.0%, 0.0-9.6)	0/36 (0.0%, 0.0-9.6)	1/42 (2.4%, 0.4-12.3)	0/41 (0.0%, 0.0-8.6)	6/34 (17.6%, 8.3-33.5)	0/19 (0.0%, 0.0-16.8)	18/556 (3.2%, 2.1-5.1)	78.55	5.26E-11	***
Pfk13	K108E	0/33 (0.0%, 0.0-10.4)	0/40 (0.0%, 0.0-8.8)	1/38 (2.6%, 0.5-13.5)	0/28 (0.0%, 0.0-12.1)	0/45 (0.0%, 0.0-7.9)	0/42 (0.0%, 0.0-8.4)	3/49 (6.1%, 2.1-16.5)	0/37 (0.0%, 0.0-9.4)	7/43 (16.3%, 8.1-30.0)	0/42 (0.0%, 0.0-8.4)	0/42 (0.0%, 0.0-8.4)	0/42 (0.0%, 0.0-8.4)	1/41 (2.4%, 0.4-12.6)	5/34 (14.7%, 6.4-30.1)	0/19 (0.0%, 0.0-16.8)	17/575 (3.0%, 1.9-4.7)	56	5.83E-07	***
	L143V	0/33 (0.0%, 0.0-10.4)	0/40 (0.0%, 0.0-8.8)	0/38 (0.0%, 0.0-9.2)	0/28 (0.0%, 0.0-12.1)	0/45 (0.0%, 0.0-7.9)	0/42 (0.0%, 0.0-8.4)	8/49 (16.3%, 8.5-29.0)	0/37 (0.0%, 0.0-9.4)	0/43 (0.0%, 0.0-8.2)	0/42 (0.0%, 0.0-8.4)	0/42 (0.0%, 0.0-8.4)	0/42 (0.0%, 0.0-8.4)	2/41 (4.9%, 1.3-16.1)	0/34 (0.0%, 0.0-10.2)	0/19 (0.0%, 0.0-16.8)	10/575 (1.7%, 0.9-3.2)	71.96	8.49E-10	***
	K189N	0/33 (0.0%, 0.0-10.4)	0/40 (0.0%, 0.0-8.8)	2/38 (5.3%, 1.5-17.3)	1/28 (3.6%, 0.6-17.7)	0/45 (0.0%, 0.0-7.9)	0/42 (0.0%, 0.0-8.4)	6/49 (12.2%, 5.7-24.2)	0/37 (0.0%, 0.0-9.4)	1/43 (2.3%, 0.4-12.1)	1/42 (2.4%, 0.4-12.3)	1/42 (2.4%, 0.4-12.3)	1/42 (2.4%, 0.4-12.3)	2/41 (4.9%, 1.3-16.1)	1/34 (2.9%, 0.5-14.9)	0/19 (0.0%, 0.0-16.8)	16/575 (2.8%, 1.7-4.5)	24.11	0.0445	*
	K189T	3/33 (9.1%, 3.1-23.6)	3/40 (7.5%, 2.6-19.9)	6/38 (15.8%, 7.4-30.4)	6/28 (21.4%, 10.2-39.5)	0/45 (0.0%, 0.0-7.9)	17/42 (40.5%, 27.0-55.5)	19/49 (38.8%, 26.4-52.8)	17/37 (45.9%, 31.0-61.6)	19/43 (44.2%, 30.4-58.9)	11/42 (26.2%, 15.3-41.1)	12/42 (28.6%, 17.2-43.6)	18/42 (42.9%, 29.1-57.8)	23/41 (56.1%, 41.0-70.1)	23/34 (67.6%, 50.8-80.9)	0/19 (0.0%, 0.0-16.8)	177/575 (30.8%, 27.1-34.7)	99.43	6.10E-15	***
	R255K	0/33 (0.0%, 0.0-10.4)	0/40 (0.0%, 0.0-8.8)	2/38 (5.3%, 1.5-17.3)	7/28 (25.0%, 12.7-43.4)	0/45 (0.0%, 0.0-7.9)	0/42 (0.0%, 0.0-8.4)	5/49 (10.2%, 4.4-21.8)	0/37 (0.0%, 0.0-9.4)	11/43 (25.6%, 14.9-40.2)	0/42 (0.0%, 0.0-8.4)	3/42 (7.1%, 2.5-19.0)	1/42 (2.4%, 0.4-12.3)	7/41 (17.1%, 8.5-31.3)	4/34 (11.8%, 4.7-26.6)	0/19 (0.0%, 0.0-16.8)	40/575 (7.0%, 5.2-9.3)	66.44	8.44E-09	***

	L258M	0/33 (0.0%, 0.0-10.4)	0/40 (0.0%, 0.0-8.8)	0/38 (0.0%, 0.0-9.2)	12/28 (42.9%, 26.5-60.9)	0/45 (0.0%, 0.0-7.9)	0/42 (0.0%, 0.0-8.4)	5/49 (10.2%, 4.4-21.8)	0/37 (0.0%, 0.0-9.4)	2/43 (4.7%, 1.3-15.5)	0/42 (0.0%, 0.0-8.4)	1/42 (2.4%, 0.4-12.3)	1/42 (2.4%, 0.4-12.3)	1/41 (2.4%, 0.4-12.6)	0/34 (0.0%, 0.0-10.2)	0/19 (0.0%, 0.0-16.8)	22/575 (3.8%, 2.5-5.7)	135.24	6.25E-22	***
	I352T	0/33 (0.0%, 0.0-10.4)	0/40 (0.0%, 0.0-8.8)	0/38 (0.0%, 0.0-9.2)	0/28 (0.0%, 0.0-12.1)	0/45 (0.0%, 0.0-7.9)	0/42 (0.0%, 0.0-8.4)	6/49 (12.2%, 5.7-24.2)	0/37 (0.0%, 0.0-9.4)	0/43 (0.0%, 0.0-8.2)	2/42 (4.8%, 1.3-15.8)	1/42 (2.4%, 0.4-12.3)	1/42 (2.4%, 0.4-12.3)	1/41 (2.4%, 0.4-12.6)	0/34 (0.0%, 0.0-10.2)	0/19 (0.0%, 0.0-16.8)	11/575 (1.9%, 1.1-3.4)	36.85	0.000777	***
	E433D	0/33 (0.0%, 0.0-10.4)	0/40 (0.0%, 0.0-8.8)	2/38 (5.3%, 1.5-17.3)	2/28 (7.1%, 2.0-22.6)	0/45 (0.0%, 0.0-7.9)	0/42 (0.0%, 0.0-8.4)	1/49 (2.0%, 0.4-10.7)	0/37 (0.0%, 0.0-9.4)	2/43 (4.7%, 1.3-15.5)	0/42 (0.0%, 0.0-8.4)	1/42 (2.4%, 0.4-12.3)	0/42 (0.0%, 0.0-8.4)	0/41 (0.0%, 0.0-8.6)	2/34 (5.9%, 1.6-19.1)	0/19 (0.0%, 0.0-16.8)	10/575 (1.7%, 0.9-3.2)	19.26	0.155	ns

Novel missense mutations, detected by at least two variant callers and were present in at least 10 samples. Geographic variation in variant frequencies was assessed using chi-squared tests (statistical significance: $p < 0.05$). N indicates the number of samples successfully genotyped for each marker. The Overall N is the aggregate count across all regions. This value may differ by marker, as samples without genotype data at a specific locus are excluded from the prevalence calculation for that marker.

Supplementary Table 4. List of synonymous mutations

Gene	Variant	Nucleotide change	Amibara (n/N, %, 95% CI)	Batu/Zeway (n/N, %, 95% CI)	Dera (n/N, %, 95% CI)	Dasenech (n/N, %, 95% CI)	Fedis (n/N, %, 95% CI)	Fentale (n/N, %, 95% CI)	Gambella zuria (n/N, %, 95% CI)	Guba (n/N, %, 95% CI)	K. Humera (n/N, %, 95% CI)	Mizan (n/N, %, 95% CI)	Metema (n/N, %, 95% CI)	Pawe (n/N, %, 95% CI)	Salamago (n/N, %, 95% CI)	Gondar (n/N, %, 95% CI)	Tanqua Abergelle (n/N, %, 95% CI)
Pfdhps	K142K	426 G->A	8/33 (24.2%, 11.1-42.3)	1/40 (2.5%, 0.1-13.2)	9/27 (33.3%, 16.5-54.0)	2/36 (5.6%, 0.7-18.7)	0/45 (0.0%, 0.0-7.9)	6/44 (13.6%, 5.2-27.4)	12/36 (33.3%, 18.6-51.0)	13/43 (30.2%, 17.2-46.1)	20/40 (50.0%, 33.8-66.2)	9/42 (21.4%, 10.3-36.8)	23/41 (56.1%, 39.7-71.5)	12/41 (29.3%, 16.1-45.5)	2/33 (6.1%, 0.7-20.2)	14/33 (42.4%, 25.5-60.8)	6/19 (31.6%, 12.6-56.6)
Pfmdr1	K770K	2310 A->G	0/33 (0.0%, 0.0-10.6)	0/40 (0.0%, 0.0-8.8)	9/28 (32.1%, 15.9-52.4)	0/34 (0.0%, 0.0-10.3)	0/45 (0.0%, 0.0-7.9)	0/34 (0.0%, 0.0-10.3)	0/47 (0.0%, 0.0-7.5)	0/43 (0.0%, 0.0-8.2)	1/36 (2.8%, 0.1-14.5)	0/42 (0.0%, 0.0-8.4)	0/36 (0.0%, 0.0-9.7)	0/41 (0.0%, 0.0-8.6)	0/34 (0.0%, 0.0-10.3)	0/33 (0.0%, 0.0-10.6)	0/19 (0.0%, 0.0-17.6)
	A840A	2520 T->C	0/33 (0.0%, 0.0-10.6)	1/40 (2.5%, 0.1-13.2)	14/28 (50.0%, 30.6-69.4)	0/34 (0.0%, 0.0-10.3)	0/45 (0.0%, 0.0-7.9)	0/34 (0.0%, 0.0-10.3)	11/47 (23.4%, 12.3-38.0)	4/43 (9.3%, 2.6-22.1)	0/36 (0.0%, 0.0-9.7)	0/42 (0.0%, 0.0-8.4)	2/36 (5.6%, 0.7-18.7)	8/41 (19.5%, 8.8-34.9)	1/34 (2.9%, 0.1-15.3)	0/33 (0.0%, 0.0-10.6)	0/19 (0.0%, 0.0-17.6)
	T1069T	3207 T->G	0/33 (0.0%, 0.0-10.6)	1/40 (2.5%, 0.1-13.2)	13/28 (46.4%, 27.5-66.1)	0/34 (0.0%, 0.0-10.3)	0/45 (0.0%, 0.0-7.9)	0/34 (0.0%, 0.0-10.3)	9/47 (19.1%, 9.1-33.3)	2/43 (4.7%, 0.6-15.8)	1/36 (2.8%, 0.1-14.5)	0/42 (0.0%, 0.0-8.4)	1/36 (2.8%, 0.1-14.5)	2/41 (4.9%, 0.6-16.5)	2/34 (5.9%, 0.7-19.7)	0/33 (0.0%, 0.0-10.6)	0/19 (0.0%, 0.0-17.6)
Pfk13	L119L	355 T->C	0/33 (0.0%, 0.0-10.6)	0/40 (0.0%, 0.0-8.8)	11/28 (39.3%, 21.5-59.4)	2/37 (5.4%, 0.7-18.2)	0/45 (0.0%, 0.0-7.9)	0/42 (0.0%, 0.0-8.4)	1/49 (2.0%, 0.1-10.9)	3/43 (7.0%, 1.5-19.1)	0/42 (0.0%, 0.0-8.4)	0/42 (0.0%, 0.0-8.4)	3/42 (7.1%, 1.5-19.5)	1/41 (2.4%, 0.1-12.9)	2/34 (5.9%, 0.7-19.7)	0/35 (0.0%, 0.0-10.0)	0/19 (0.0%, 0.0-17.6)

Synonymous mutations, detected by at least two variant callers and present in at least 10 samples, per study district. N indicates the number of samples successfully genotyped for each marker. The Overall N is the aggregate count across all regions. This value may differ by marker, as samples without genotype data at a specific locus are excluded from the prevalence calculation for that marker.

Supplementary Table 5. Prevalence of Wild-type variants for WHO known Mutation across Key Drug resistance Gene

Gene	Mutation	Amibara (n, %, 95% CI)	Batu/Zeway (n, %, 95% CI)	Dera (n, %, 95% CI)	Dasenech (n, %, 95% CI)	Fedis (n, %, 95% CI)	Fentale (n, %, 95% CI)	Gambella zuria (n, %, 95% CI)	Guba (n, %, 95% CI)	K. Humera (n, %, 95% CI)	Mizan (n, %, 95% CI)	Metema (n, %, 95% CI)	Pawe (n, %, 95% CI)	Salamago (n, %, 95% CI)	Gondar (n, %, 95% CI)	Tanqua Abergelle (n, %, 95% CI)	Overall (n, %, 95% CI)	Chi²	p-value
Pfdhfr	**A16**	(33/33, 100.0%, 89.6-100.0)	(40/40, 100.0%, 91.2-100.0)	(27/27, 100.0%, 87.5-100.0)	(38/38, 100.0%, 90.8-100.0)	(45/45, 100.0%, 92.1-100.0)	(44/44, 100.0%, 92.0-100.0)	(48/48, 100.0%, 92.6-100.0)	(43/43, 100.0%, 91.8-100.0)	(42/42, 100.0%, 91.6-100.0)	(42/42, 100.0%, 91.6-100.0)	(42/42, 100.0%, 91.6-100.0)	(41/41, 100.0%, 91.4-100.0)	(33/33, 100.0%, 89.6-100.0)	(33/33, 100.0%, 89.6-100.0)	(19/19, 100.0%, 83.2-100.0)	(570/570, 100.0%, 99.3-100.0)	N/A	N/A
	**N51**	(0/33, 0.0%, 0.0-10.4)	(5/40, 12.5%, 5.5-26.1)	(0/27, 0.0%, 0.0-12.5)	(2/37, 5.4%, 1.5-17.7)	(0/45, 0.0%, 0.0-7.9)	(0/44, 0.0%, 0.0-8.0)	(0/48, 0.0%, 0.0-7.4)	(22/43, 51.2%, 36.8-65.4)	(0/42, 0.0%, 0.0-8.4)	(0/42, 0.0%, 0.0-8.4)	(3/42, 7.1%, 2.5-19.0)	(16/41, 39.0%, 25.7-54.3)	(3/33, 9.1%, 3.1-23.6)	(0/33, 0.0%, 0.0-10.4)	(0/19, 0.0%, 0.0-16.8)	(51/569, 9.0%, 6.9-11.6)	1.734e+02	1.409e-29
	**C59**	(13/33, 39.4%, 24.7-56.3)	(1/40, 2.5%, 0.4-12.9)	(26/27, 96.3%, 81.7-99.3)	(9/37, 24.3%, 13.4-40.1)	(4/45, 8.9%, 3.5-20.7)	(10/44, 22.7%, 12.8-37.0)	(23/48, 47.9%, 34.5-61.7)	(38/43, 88.4%, 75.5-94.9)	(27/42, 64.3%, 49.2-77.0)	(2/42, 4.8%, 1.3-15.8)	(29/42, 69.0%, 54.0-80.9)	(28/41, 68.3%, 53.0-80.4)	(16/33, 48.5%, 32.5-64.8)	(19/33, 57.6%, 40.8-72.8)	(16/19, 84.2%, 62.4-94.5)	(261/569, 45.9%, 41.8-50.0)	1.959e+02	3.700e-34
	**S108**	(0/33, 0.0%, 0.0-10.4)	(0/40, 0.0%, 0.0-8.8)	(0/27, 0.0%, 0.0-12.5)	(2/37, 5.4%, 1.5-17.7)	(0/45, 0.0%, 0.0-7.9)	(0/44, 0.0%, 0.0-8.0)	(0/48, 0.0%, 0.0-7.4)	(22/43, 51.2%, 36.8-65.4)	(1/42, 2.4%, 0.4-12.3)	(0/42, 0.0%, 0.0-8.4)	(3/42, 7.1%, 2.5-19.0)	(16/41, 39.0%, 25.7-54.3)	(3/33, 9.1%, 3.1-23.6)	(0/33, 0.0%, 0.0-10.4)	(0/19, 0.0%, 0.0-16.8)	(47/569, 8.3%, 6.3-10.8)	1.879e+02	1.632e-32
	**I164**	(33/33, 100.0%, 89.6-100.0)	(40/40, 100.0%, 91.2-100.0)	(27/27, 100.0%, 87.5-100.0)	(37/37, 100.0%, 90.6-100.0)	(45/45, 100.0%, 92.1-100.0)	(44/44, 100.0%, 92.0-100.0)	(48/48, 100.0%, 92.6-100.0)	(43/43, 100.0%, 91.8-100.0)	(42/42, 100.0%, 91.6-100.0)	(42/42, 100.0%, 91.6-100.0)	(42/42, 100.0%, 91.6-100.0)	(41/41, 100.0%, 91.4-100.0)	(33/33, 100.0%, 89.6-100.0)	(33/33, 100.0%, 89.6-100.0)	(19/19, 100.0%, 83.2-100.0)	(569/569, 100.0%, 99.3-100.0)	N/A	N/A
Pfdhps	**I431**	(33/33, 100.0%, 89.6-100.0)	(40/40, 100.0%, 91.2-100.0)	(27/27, 100.0%, 87.5-100.0)	(36/36, 100.0%, 90.4-100.0)	(45/45, 100.0%, 92.1-100.0)	(44/44, 100.0%, 92.0-100.0)	(36/36, 100.0%, 90.4-100.0)	(43/43, 100.0%, 91.8-100.0)	(40/40, 100.0%, 91.2-100.0)	(42/42, 100.0%, 91.6-100.0)	(41/41, 100.0%, 91.4-100.0)	(41/41, 100.0%, 91.4-100.0)	(32/32, 100.0%, 89.3-100.0)	(33/33, 100.0%, 89.6-100.0)	(19/19, 100.0%, 83.2-100.0)	(552/552, 100.0%, 99.3-100.0)	N/A	N/A
	**S436**	(33/33, 100.0%, 89.6-100.0)	(40/40, 100.0%, 91.2-100.0)	(27/27, 100.0%, 87.5-100.0)	(36/36, 100.0%, 90.4-100.0)	(45/45, 100.0%, 92.1-100.0)	(44/44, 100.0%, 92.0-100.0)	(36/36, 100.0%, 90.4-100.0)	(43/43, 100.0%, 91.8-100.0)	(39/40, 97.5%, 87.1-99.6)	(42/42, 100.0%, 91.6-100.0)	(41/41, 100.0%, 91.4-100.0)	(39/41, 95.1%, 83.9-98.7)	(32/32, 100.0%, 89.3-100.0)	(33/33, 100.0%, 89.6-100.0)	(19/19, 100.0%, 83.2-100.0)	(549/552, 99.5%, 98.4-99.8)	1.966e+01	1.413e-01
	**A437**	(7/33, 21.2%, 10.7-37.8)	(1/40, 2.5%, 0.4-12.9)	(2/27, 7.4%, 2.1-23.4)	(16/36, 44.4%, 29.5-60.4)	(0/45, 0.0%, 0.0-7.9)	(4/44, 9.1%, 3.6-21.2)	(17/36, 47.2%, 32.0-63.0)	(33/43, 76.7%, 62.3-86.8)	(6/40, 15.0%, 7.1-29.1)	(4/42, 9.5%, 3.8-22.1)	(11/41, 26.8%, 15.7-41.9)	(33/41, 80.5%, 66.0-89.8)	(20/32, 62.5%, 45.3-77.1)	(2/33, 6.1%, 1.7-19.6)	(1/19, 5.3%, 0.9-24.6)	(157/552, 28.4%, 24.8-32.3)	2.029e+02	1.391e-35
	**K540**	(7/33, 21.2%, 10.7-37.8)	(1/40, 2.5%, 0.4-12.9)	(2/27, 7.4%, 2.1-23.4)	(16/36, 44.4%, 29.5-60.4)	(0/45, 0.0%, 0.0-7.9)	(4/44, 9.1%, 3.6-21.2)	(17/35, 48.6%, 33.0-64.4)	(33/43, 76.7%, 62.3-86.8)	(6/40, 15.0%, 7.1-29.1)	(4/42, 9.5%, 3.8-22.1)	(12/41, 29.3%, 17.6-44.5)	(34/41, 82.9%, 68.7-91.5)	(20/32, 62.5%, 45.3-77.1)	(2/33, 6.1%, 1.7-19.6)	(1/19, 5.3%, 0.9-24.6)	(159/551, 28.9%, 25.2-32.8)	2.070e+02	2.038e-36
	**A581**	(33/33, 100.0%, 89.6-100.0)	(40/40, 100.0%, 91.2-100.0)	(27/27, 100.0%, 87.5-100.0)	(36/36, 100.0%, 90.4-100.0)	(45/45, 100.0%, 92.1-100.0)	(43/44, 97.7%, 88.2-99.6)	(27/36, 75.0%, 58.9-86.2)	(42/43, 97.7%, 87.9-99.6)	(34/40, 85.0%, 70.9-92.9)	(42/42, 100.0%, 91.6-100.0)	(36/41, 87.8%, 74.5-94.7)	(38/41, 92.7%, 80.6-97.5)	(32/32, 100.0%, 89.3-100.0)	(28/33, 84.8%, 69.1-93.3)	(18/19, 94.7%, 75.4-99.1)	(521/552, 94.4%, 92.1-96.0)	5.838e+01	2.251e-07

	**A613**	(33/33, 100.0%, 89.6-100.0)	(40/40, 100.0%, 91.2-100.0)	(27/27, 100.0%, 87.5-100.0)	(36/36, 100.0%, 90.4-100.0)	(45/45, 100.0%, 92.1-100.0)	(44/44, 100.0%, 92.0-100.0)	(36/36, 100.0%, 90.4-100.0)	(43/43, 100.0%, 91.8-100.0)	(40/40, 100.0%, 91.2-100.0)	(42/42, 100.0%, 91.6-100.0)	(41/41, 100.0%, 91.4-100.0)	(41/41, 100.0%, 91.4-100.0)	(33/33, 100.0%, 89.6-100.0)	(33/33, 100.0%, 89.6-100.0)	(19/19, 100.0%, 83.2-100.0)	(553/553, 100.0%, 99.3-100.0)	N/A	N/A
Pfcrt	**C72**	(33/33, 100.0%, 89.6-100.0)	(39/39, 100.0%, 91.0-100.0)	(23/23, 100.0%, 85.7-100.0)	(36/36, 100.0%, 90.4-100.0)	(44/44, 100.0%, 92.0-100.0)	(41/41, 100.0%, 91.4-100.0)	(27/27, 100.0%, 87.5-100.0)	(41/41, 100.0%, 91.4-100.0)	(42/42, 100.0%, 91.6-100.0)	(40/40, 100.0%, 91.2-100.0)	(42/42, 100.0%, 91.6-100.0)	(35/35, 100.0%, 90.1-100.0)	(31/31, 100.0%, 89.0-100.0)	(30/30, 100.0%, 88.6-100.0)	(19/19, 100.0%, 83.2-100.0)	(523/523, 100.0%, 99.3-100.0)	N/A	N/A
	**V73**	(33/33, 100.0%, 89.6-100.0)	(39/39, 100.0%, 91.0-100.0)	(23/23, 100.0%, 85.7-100.0)	(36/36, 100.0%, 90.4-100.0)	(44/44, 100.0%, 92.0-100.0)	(41/41, 100.0%, 91.4-100.0)	(27/27, 100.0%, 87.5-100.0)	(41/41, 100.0%, 91.4-100.0)	(42/42, 100.0%, 91.6-100.0)	(40/40, 100.0%, 91.2-100.0)	(42/42, 100.0%, 91.6-100.0)	(35/35, 100.0%, 90.1-100.0)	(31/31, 100.0%, 89.0-100.0)	(30/30, 100.0%, 88.6-100.0)	(19/19, 100.0%, 83.2-100.0)	(523/523, 100.0%, 99.3-100.0)	N/A	N/A
	**M74**	(2/33, 6.1%, 1.7-19.6)	(1/39, 2.6%, 0.5-13.2)	(2/23, 8.7%, 2.4-26.8)	(9/36, 25.0%, 13.8-41.1)	(0/44, 0.0%, 0.0-8.0)	(3/41, 7.3%, 2.5-19.4)	(25/27, 92.6%, 76.6-97.9)	(39/41, 95.1%, 83.9-98.7)	(15/42, 35.7%, 23.0-50.8)	(12/40, 30.0%, 18.1-45.4)	(23/42, 54.8%, 39.9-68.8)	(30/35, 85.7%, 70.6-93.7)	(26/31, 83.9%, 67.4-92.9)	(3/30, 10.0%, 3.5-25.6)	(4/19, 21.1%, 8.5-43.3)	(194/523, 37.1%, 33.1-41.3)	2.627e+02	6.877e-48
	**N75**	(2/33, 6.1%, 1.7-19.6)	(1/39, 2.6%, 0.5-13.2)	(2/23, 8.7%, 2.4-26.8)	(9/36, 25.0%, 13.8-41.1)	(0/44, 0.0%, 0.0-8.0)	(3/41, 7.3%, 2.5-19.4)	(25/27, 92.6%, 76.6-97.9)	(39/41, 95.1%, 83.9-98.7)	(15/42, 35.7%, 23.0-50.8)	(12/40, 30.0%, 18.1-45.4)	(23/42, 54.8%, 39.9-68.8)	(30/35, 85.7%, 70.6-93.7)	(26/31, 83.9%, 67.4-92.9)	(5/30, 16.7%, 7.3-33.6)	(4/19, 21.1%, 8.5-43.3)	(196/523, 37.5%, 33.4-41.7)	2.575e+02	8.138e-47
	**K76**	(2/33, 6.1%, 1.7-19.6)	(1/39, 2.6%, 0.5-13.2)	(2/23, 8.7%, 2.4-26.8)	(9/36, 25.0%, 13.8-41.1)	(0/44, 0.0%, 0.0-8.0)	(3/41, 7.3%, 2.5-19.4)	(25/27, 92.6%, 76.6-97.9)	(39/41, 95.1%, 83.9-98.7)	(15/42, 35.7%, 23.0-50.8)	(12/40, 30.0%, 18.1-45.4)	(21/42, 50.0%, 35.5-64.5)	(29/35, 82.9%, 67.3-91.9)	(26/31, 83.9%, 67.4-92.9)	(3/30, 10.0%, 3.5-25.6)	(4/19, 21.1%, 8.5-43.3)	(191/523, 36.5%, 32.5-40.7)	2.576e+02	7.720e-47
	**A220**	(2/33, 6.1%, 1.7-19.6)	(1/40, 2.5%, 0.4-12.9)	(2/23, 8.7%, 2.4-26.8)	(8/34, 23.5%, 12.4-40.0)	(0/44, 0.0%, 0.0-8.0)	(3/40, 7.5%, 2.6-19.9)	(25/27, 92.6%, 76.6-97.9)	(38/40, 95.0%, 83.5-98.6)	(15/42, 35.7%, 23.0-50.8)	(12/40, 30.0%, 18.1-45.4)	(19/42, 45.2%, 31.2-60.1)	(29/35, 82.9%, 67.3-91.9)	(25/31, 80.6%, 63.7-90.8)	(2/30, 6.7%, 1.8-21.3)	(4/19, 21.1%, 8.5-43.3)	(185/520, 35.6%, 31.6-39.8)	2.556e+02	2.023e-46
	**Q271**	(2/33, 6.1%, 1.7-19.6)	(1/40, 2.5%, 0.4-12.9)	(2/24, 8.3%, 2.3-25.8)	(9/36, 25.0%, 13.8-41.1)	(0/43, 0.0%, 0.0-8.2)	(3/41, 7.3%, 2.5-19.4)	(25/27, 92.6%, 76.6-97.9)	(39/41, 95.1%, 83.9-98.7)	(15/42, 35.7%, 23.0-50.8)	(12/40, 30.0%, 18.1-45.4)	(20/42, 47.6%, 33.4-62.3)	(28/34, 82.4%, 66.5-91.7)	(25/31, 80.6%, 63.7-90.8)	(2/30, 6.7%, 1.8-21.3)	(4/19, 21.1%, 8.5-43.3)	(187/523, 35.8%, 31.8-40.0)	2.562e+02	1.527e-46
	**N326**	(2/33, 6.1%, 1.7-19.6)	(2/39, 5.1%, 1.4-16.9)	(2/22, 9.1%, 2.5-27.8)	(14/35, 40.0%, 25.6-56.4)	(0/43, 0.0%, 0.0-8.2)	(3/39, 7.7%, 2.7-20.3)	(26/27, 96.3%, 81.7-99.3)	(39/40, 97.5%, 87.1-99.6)	(17/42, 40.5%, 27.0-55.5)	(13/40, 32.5%, 20.1-48.0)	(19/41, 46.3%, 32.1-61.3)	(32/34, 94.1%, 80.9-98.4)	(30/31, 96.8%, 83.8-99.4)	(2/30, 6.7%, 1.8-21.3)	(4/19, 21.1%, 8.5-43.3)	(205/515, 39.8%, 35.7-44.1)	2.827e+02	4.781e-52
	**I356**	(33/33, 100.0%, 89.6-100.0)	(39/39, 100.0%, 91.0-100.0)	(23/23, 100.0%, 85.7-100.0)	(30/36, 83.3%, 68.1-92.1)	(44/44, 100.0%, 92.0-100.0)	(42/42, 100.0%, 91.6-100.0)	(27/27, 100.0%, 87.5-100.0)	(41/41, 100.0%, 91.4-100.0)	(42/42, 100.0%, 91.6-100.0)	(40/40, 100.0%, 91.2-100.0)	(41/42, 97.6%, 87.7-99.6)	(34/35, 97.1%, 85.5-99.5)	(30/31, 96.8%, 83.8-99.4)	(30/30, 100.0%, 88.6-100.0)	(19/19, 100.0%, 83.2-100.0)	(515/524, 98.3%, 96.8-99.1)	5.510e+01	8.334e-07
	**R371**	(2/33, 6.1%, 1.7-19.6)	(1/39, 2.6%, 0.5-13.2)	(2/21, 9.5%, 2.7-28.9)	(9/36, 25.0%, 13.8-41.1)	(0/43, 0.0%, 0.0-8.2)	(3/42, 7.1%, 2.5-19.0)	(25/27, 92.6%, 76.6-97.9)	(39/41, 95.1%, 83.9-98.7)	(15/42, 35.7%, 23.0-50.8)	(12/40, 30.0%, 18.1-45.4)	(18/41, 43.9%, 29.9-59.0)	(29/35, 82.9%, 67.3-91.9)	(24/31, 77.4%, 60.2-88.6)	(2/29, 6.9%, 1.9-22.0)	(4/19, 21.1%, 8.5-43.3)	(185/519, 35.6%,	2.509e+02	1.884e-45

																	31.6-39.9)		
	**N86**	(26/33, 78.8%, 62.2-89.3)	(40/40, 100.0%, 91.2-100.0)	(27/28, 96.4%, 82.3-99.4)	(31/33, 93.9%, 80.4-98.3)	(45/45, 100.0%, 92.1-100.0)	(34/34, 100.0%, 89.8-100.0)	(46/47, 97.9%, 88.9-99.6)	(43/43, 100.0%, 91.8-100.0)	(36/36, 100.0%, 90.4-100.0)	(42/42, 100.0%, 91.6-100.0)	(35/35, 100.0%, 90.1-100.0)	(41/41, 100.0%, 91.4-100.0)	(34/34, 100.0%, 89.8-100.0)	(32/32, 100.0%, 89.3-100.0)	(19/19, 100.0%, 83.2-100.0)	(531/542, 98.0%, 96.4-98.9)	7.241e+01	7.034e-10
	**Y184**	(8/33, 24.2%, 12.8-41.0)	(0/40, 0.0%, 0.0-8.8)	(0/28, 0.0%, 0.0-12.1)	(4/33, 12.1%, 4.8-27.3)	(0/45, 0.0%, 0.0-7.9)	(1/33, 3.0%, 0.5-15.3)	(4/47, 8.5%, 3.4-19.9)	(6/43, 14.0%, 6.6-27.3)	(0/35, 0.0%, 0.0-9.9)	(0/42, 0.0%, 0.0-8.4)	(5/36, 13.9%, 6.1-28.7)	(5/41, 12.2%, 5.3-25.5)	(1/33, 3.0%, 0.5-15.3)	(1/30, 3.3%, 0.6-16.7)	(0/19, 0.0%, 0.0-16.8)	(35/538, 6.5%, 4.7-8.9)	4.477e+01	4.445e-05
Pfmdr1	**S1034**	(33/33, 100.0%, 89.6-100.0)	(40/40, 100.0%, 91.2-100.0)	(28/28, 100.0%, 87.9-100.0)	(33/33, 100.0%, 89.6-100.0)	(44/44, 100.0%, 92.0-100.0)	(33/33, 100.0%, 89.6-100.0)	(47/47, 100.0%, 92.4-100.0)	(43/43, 100.0%, 91.8-100.0)	(36/36, 100.0%, 90.4-100.0)	(41/41, 100.0%, 91.4-100.0)	(33/33, 100.0%, 89.6-100.0)	(41/41, 100.0%, 91.4-100.0)	(33/33, 100.0%, 89.6-100.0)	(31/31, 100.0%, 89.0-100.0)	(19/19, 100.0%, 83.2-100.0)	(535/535, 100.0%, 99.3-100.0)	N/A	N/A
	**N1042**	(33/33, 100.0%, 89.6-100.0)	(40/40, 100.0%, 91.2-100.0)	(28/28, 100.0%, 87.9-100.0)	(33/33, 100.0%, 89.6-100.0)	(43/43, 100.0%, 91.8-100.0)	(33/33, 100.0%, 89.6-100.0)	(47/47, 100.0%, 92.4-100.0)	(43/43, 100.0%, 91.8-100.0)	(36/36, 100.0%, 90.4-100.0)	(41/41, 100.0%, 91.4-100.0)	(31/31, 100.0%, 89.0-100.0)	(41/41, 100.0%, 91.4-100.0)	(33/33, 100.0%, 89.6-100.0)	(30/30, 100.0%, 88.6-100.0)	(19/19, 100.0%, 83.2-100.0)	(531/531, 100.0%, 99.3-100.0)	N/A	N/A
	**D1246**	(33/33, 100.0%, 89.6-100.0)	(40/40, 100.0%, 91.2-100.0)	(28/28, 100.0%, 87.9-100.0)	(34/34, 100.0%, 89.8-100.0)	(45/45, 100.0%, 92.1-100.0)	(33/33, 100.0%, 89.6-100.0)	(47/47, 100.0%, 92.4-100.0)	(43/43, 100.0%, 91.8-100.0)	(35/35, 100.0%, 90.1-100.0)	(41/41, 100.0%, 91.4-100.0)	(33/33, 100.0%, 89.6-100.0)	(41/41, 100.0%, 91.4-100.0)	(33/33, 100.0%, 89.6-100.0)	(31/31, 100.0%, 89.0-100.0)	(19/19, 100.0%, 83.2-100.0)	(536/536, 100.0%, 99.3-100.0)	N/A	N/A
	**P441**	(33/33, 100.0%, 89.6-100.0)	(35/40, 87.5%, 73.9-94.5)	(28/28, 100.0%, 87.9-100.0)	(37/37, 100.0%, 90.6-100.0)	(45/45, 100.0%, 92.1-100.0)	(41/41, 100.0%, 91.4-100.0)	(49/49, 100.0%, 92.7-100.0)	(43/43, 100.0%, 91.8-100.0)	(42/42, 100.0%, 91.6-100.0)	(42/42, 100.0%, 91.6-100.0)	(42/42, 100.0%, 91.6-100.0)	(41/41, 100.0%, 91.4-100.0)	(33/34, 97.1%, 85.1-99.5)	(35/35, 100.0%, 90.1-100.0)	(19/19, 100.0%, 83.2-100.0)	(565/571, 98.9%, 97.7-99.5)	5.688e+01	4.110e-07
	**F446**	(33/33, 100.0%, 89.6-100.0)	(40/40, 100.0%, 91.2-100.0)	(28/28, 100.0%, 87.9-100.0)	(37/37, 100.0%, 90.6-100.0)	(45/45, 100.0%, 92.1-100.0)	(41/41, 100.0%, 91.4-100.0)	(49/49, 100.0%, 92.7-100.0)	(43/43, 100.0%, 91.8-100.0)	(42/42, 100.0%, 91.6-100.0)	(42/42, 100.0%, 91.6-100.0)	(42/42, 100.0%, 91.6-100.0)	(41/41, 100.0%, 91.4-100.0)	(34/34, 100.0%, 89.8-100.0)	(35/35, 100.0%, 90.1-100.0)	(19/19, 100.0%, 83.2-100.0)	(571/571, 100.0%, 99.3-100.0)	N/A	N/A
Pfk13	**G449**	(33/33, 100.0%, 89.6-100.0)	(40/40, 100.0%, 91.2-100.0)	(28/28, 100.0%, 87.9-100.0)	(37/37, 100.0%, 90.6-100.0)	(45/45, 100.0%, 92.1-100.0)	(41/41, 100.0%, 91.4-100.0)	(49/49, 100.0%, 92.7-100.0)	(43/43, 100.0%, 91.8-100.0)	(42/42, 100.0%, 91.6-100.0)	(42/42, 100.0%, 91.6-100.0)	(42/42, 100.0%, 91.6-100.0)	(41/41, 100.0%, 91.4-100.0)	(34/34, 100.0%, 89.8-100.0)	(35/35, 100.0%, 90.1-100.0)	(19/19, 100.0%, 83.2-100.0)	(571/571, 100.0%, 99.3-100.0)	N/A	N/A
	**N458**	(33/33, 100.0%, 89.6-100.0)	(40/40, 100.0%, 91.2-100.0)	(28/28, 100.0%, 87.9-100.0)	(37/37, 100.0%, 90.6-100.0)	(45/45, 100.0%, 92.1-100.0)	(41/41, 100.0%, 91.4-100.0)	(49/49, 100.0%, 92.7-100.0)	(43/43, 100.0%, 91.8-100.0)	(42/42, 100.0%, 91.6-100.0)	(42/42, 100.0%, 91.6-100.0)	(42/42, 100.0%, 91.6-100.0)	(41/41, 100.0%, 91.4-100.0)	(34/34, 100.0%, 89.8-100.0)	(35/35, 100.0%, 90.1-100.0)	(19/19, 100.0%, 83.2-100.0)	(571/571, 100.0%, 99.3-100.0)	N/A	N/A
	**C469**	(33/33, 100.0%, 89.6-100.0)	(40/40, 100.0%, 91.2-100.0)	(28/28, 100.0%, 87.9-100.0)	(37/37, 100.0%, 90.6-100.0)	(45/45, 100.0%, 92.1-100.0)	(41/41, 100.0%, 91.4-100.0)	(49/49, 100.0%, 92.7-100.0)	(43/43, 100.0%, 91.8-100.0)	(42/42, 100.0%, 91.6-100.0)	(42/42, 100.0%, 91.6-100.0)	(42/42, 100.0%, 91.6-100.0)	(41/41, 100.0%, 91.4-100.0)	(34/34, 100.0%, 89.8-100.0)	(35/35, 100.0%, 90.1-100.0)	(19/19, 100.0%, 83.2-100.0)	(571/571, 100.0%, 99.3-100.0)	N/A	N/A
	**A481**	(33/33, 100.0%,	(40/40, 100.0%,	(28/28, 100.0%,	(37/37, 100.0%,	(45/45, 100.0%,	(41/41, 100.0%,	(49/49, 100.0%,	(43/43, 100.0%,	(42/42, 100.0%,	(42/42, 100.0%,	(42/42, 100.0%,	(41/41, 100.0%,	(34/34, 100.0%,	(35/35, 100.0%,	(19/19, 100.0%,	(571/571, 100.0%,	N/A	N/A

		89.6-100.0)	91.2-100.0)	87.9-100.0)	90.6-100.0)	92.1-100.0)	91.4-100.0)	92.7-100.0)	91.8-100.0)	91.6-100.0)	91.6-100.0)	91.6-100.0)	91.4-100.0)	89.8-100.0)	90.1-100.0)	83.2-100.0)	99.3-100.0)		
	**M476**	(33/33, 100.0%, 89.6-100.0)	(40/40, 100.0%, 91.2-100.0)	(28/28, 100.0%, 87.9-100.0)	(37/37, 100.0%, 90.6-100.0)	(45/45, 100.0%, 92.1-100.0)	(41/41, 100.0%, 91.4-100.0)	(49/49, 100.0%, 92.7-100.0)	(43/43, 100.0%, 91.8-100.0)	(42/42, 100.0%, 91.6-100.0)	(42/42, 100.0%, 91.6-100.0)	(42/42, 100.0%, 91.6-100.0)	(41/41, 100.0%, 91.4-100.0)	(34/34, 100.0%, 89.8-100.0)	(35/35, 100.0%, 90.1-100.0)	(19/19, 100.0%, 83.2-100.0)	(571/571, 100.0%, 99.3-100.0)	N/A	N/A
	**Y493**	(33/33, 100.0%, 89.6-100.0)	(40/40, 100.0%, 91.2-100.0)	(28/28, 100.0%, 87.9-100.0)	(37/37, 100.0%, 90.6-100.0)	(45/45, 100.0%, 92.1-100.0)	(42/42, 100.0%, 91.6-100.0)	(49/49, 100.0%, 92.7-100.0)	(43/43, 100.0%, 91.8-100.0)	(42/42, 100.0%, 91.6-100.0)	(42/42, 100.0%, 91.6-100.0)	(42/42, 100.0%, 91.6-100.0)	(41/41, 100.0%, 91.4-100.0)	(34/34, 100.0%, 89.8-100.0)	(35/35, 100.0%, 90.1-100.0)	(19/19, 100.0%, 83.2-100.0)	(572/572, 100.0%, 99.3-100.0)	N/A	N/A
	**R515**	(33/33, 100.0%, 89.6-100.0)	(40/40, 100.0%, 91.2-100.0)	(28/28, 100.0%, 87.9-100.0)	(37/37, 100.0%, 90.6-100.0)	(45/45, 100.0%, 92.1-100.0)	(41/41, 100.0%, 91.4-100.0)	(49/49, 100.0%, 92.7-100.0)	(43/43, 100.0%, 91.8-100.0)	(42/42, 100.0%, 91.6-100.0)	(42/42, 100.0%, 91.6-100.0)	(42/42, 100.0%, 91.6-100.0)	(41/41, 100.0%, 91.4-100.0)	(34/34, 100.0%, 89.8-100.0)	(35/35, 100.0%, 90.1-100.0)	(19/19, 100.0%, 83.2-100.0)	(571/571, 100.0%, 99.3-100.0)	N/A	N/A
	**P527**	(33/33, 100.0%, 89.6-100.0)	(40/40, 100.0%, 91.2-100.0)	(28/28, 100.0%, 87.9-100.0)	(37/37, 100.0%, 90.6-100.0)	(45/45, 100.0%, 92.1-100.0)	(41/41, 100.0%, 91.4-100.0)	(49/49, 100.0%, 92.7-100.0)	(43/43, 100.0%, 91.8-100.0)	(42/42, 100.0%, 91.6-100.0)	(42/42, 100.0%, 91.6-100.0)	(42/42, 100.0%, 91.6-100.0)	(41/41, 100.0%, 91.4-100.0)	(34/34, 100.0%, 89.8-100.0)	(35/35, 100.0%, 90.1-100.0)	(19/19, 100.0%, 83.2-100.0)	(571/571, 100.0%, 99.3-100.0)	N/A	N/A
	**N537**	(33/33, 100.0%, 89.6-100.0)	(40/40, 100.0%, 91.2-100.0)	(28/28, 100.0%, 87.9-100.0)	(37/37, 100.0%, 90.6-100.0)	(45/45, 100.0%, 92.1-100.0)	(41/41, 100.0%, 91.4-100.0)	(48/48, 100.0%, 92.6-100.0)	(43/43, 100.0%, 91.8-100.0)	(42/42, 100.0%, 91.6-100.0)	(42/42, 100.0%, 91.6-100.0)	(42/42, 100.0%, 91.6-100.0)	(41/41, 100.0%, 91.4-100.0)	(34/34, 100.0%, 89.8-100.0)	(35/35, 100.0%, 90.1-100.0)	(19/19, 100.0%, 83.2-100.0)	(570/570, 100.0%, 99.3-100.0)	N/A	N/A
	**G538**	(33/33, 100.0%, 89.6-100.0)	(40/40, 100.0%, 91.2-100.0)	(28/28, 100.0%, 87.9-100.0)	(37/37, 100.0%, 90.6-100.0)	(45/45, 100.0%, 92.1-100.0)	(41/41, 100.0%, 91.4-100.0)	(49/49, 100.0%, 92.7-100.0)	(43/43, 100.0%, 91.8-100.0)	(42/42, 100.0%, 91.6-100.0)	(42/42, 100.0%, 91.6-100.0)	(42/42, 100.0%, 91.6-100.0)	(41/41, 100.0%, 91.4-100.0)	(34/34, 100.0%, 89.8-100.0)	(35/35, 100.0%, 90.1-100.0)	(19/19, 100.0%, 83.2-100.0)	(571/571, 100.0%, 99.3-100.0)	N/A	N/A
	**R539**	(33/33, 100.0%, 89.6-100.0)	(40/40, 100.0%, 91.2-100.0)	(28/28, 100.0%, 87.9-100.0)	(37/37, 100.0%, 90.6-100.0)	(45/45, 100.0%, 92.1-100.0)	(41/41, 100.0%, 91.4-100.0)	(49/49, 100.0%, 92.7-100.0)	(43/43, 100.0%, 91.8-100.0)	(42/42, 100.0%, 91.6-100.0)	(42/42, 100.0%, 91.6-100.0)	(42/42, 100.0%, 91.6-100.0)	(41/41, 100.0%, 91.4-100.0)	(34/34, 100.0%, 89.8-100.0)	(35/35, 100.0%, 90.1-100.0)	(19/19, 100.0%, 83.2-100.0)	(571/571, 100.0%, 99.3-100.0)	N/A	N/A
	**I543**	(33/33, 100.0%, 89.6-100.0)	(40/40, 100.0%, 91.2-100.0)	(28/28, 100.0%, 87.9-100.0)	(37/37, 100.0%, 90.6-100.0)	(45/45, 100.0%, 92.1-100.0)	(41/41, 100.0%, 91.4-100.0)	(49/49, 100.0%, 92.7-100.0)	(43/43, 100.0%, 91.8-100.0)	(42/42, 100.0%, 91.6-100.0)	(42/42, 100.0%, 91.6-100.0)	(42/42, 100.0%, 91.6-100.0)	(41/41, 100.0%, 91.4-100.0)	(34/34, 100.0%, 89.8-100.0)	(35/35, 100.0%, 90.1-100.0)	(19/19, 100.0%, 83.2-100.0)	(571/571, 100.0%, 99.3-100.0)	N/A	N/A
	**P553**	(33/33, 100.0%, 89.6-100.0)	(40/40, 100.0%, 91.2-100.0)	(28/28, 100.0%, 87.9-100.0)	(37/37, 100.0%, 90.6-100.0)	(45/45, 100.0%, 92.1-100.0)	(41/41, 100.0%, 91.4-100.0)	(49/49, 100.0%, 92.7-100.0)	(43/43, 100.0%, 91.8-100.0)	(42/42, 100.0%, 91.6-100.0)	(42/42, 100.0%, 91.6-100.0)	(42/42, 100.0%, 91.6-100.0)	(41/41, 100.0%, 91.4-100.0)	(34/34, 100.0%, 89.8-100.0)	(35/35, 100.0%, 90.1-100.0)	(19/19, 100.0%, 83.2-100.0)	(571/571, 100.0%, 99.3-100.0)	N/A	N/A
	**R561**	(33/33, 100.0%, 89.6-100.0)	(40/40, 100.0%, 91.2-100.0)	(28/28, 100.0%, 87.9-100.0)	(37/37, 100.0%, 90.6-100.0)	(45/45, 100.0%, 92.1-100.0)	(41/41, 100.0%, 91.4-100.0)	(49/49, 100.0%, 92.7-100.0)	(43/43, 100.0%, 91.8-100.0)	(42/42, 100.0%, 91.6-100.0)	(42/42, 100.0%, 91.6-100.0)	(42/42, 100.0%, 91.6-100.0)	(41/41, 100.0%, 91.4-100.0)	(34/34, 100.0%, 89.8-100.0)	(35/35, 100.0%, 90.1-100.0)	(19/19, 100.0%, 83.2-100.0)	(571/571, 100.0%, 99.3-100.0)	N/A	N/A
	**V568**	(33/33, 100.0%,	(40/40, 100.0%,	(28/28, 100.0%,	(37/37, 100.0%,	(45/45, 100.0%,	(41/41, 100.0%,	(49/49, 100.0%,	(43/43, 100.0%,	(42/42, 100.0%,	(42/42, 100.0%,	(42/42, 100.0%,	(41/41, 100.0%,	(34/34, 100.0%,	(35/35, 100.0%,	(19/19, 100.0%,	(571/571,	N/A	N/A

		89.6-100.0)	91.2-100.0)	87.9-100.0)	90.6-100.0)	92.1-100.0)	91.4-100.0)	92.7-100.0)	91.8-100.0)	91.6-100.0)	91.6-100.0)	91.6-100.0)	91.4-100.0)	89.8-100.0)	90.1-100.0)	83.2-100.0)	100.0%, 99.3-100.0)		
	**P574**	(33/33, 100.0%, 89.6-100.0)	(40/40, 100.0%, 91.2-100.0)	(28/28, 100.0%, 87.9-100.0)	(37/37, 100.0%, 90.6-100.0)	(45/45, 100.0%, 92.1-100.0)	(41/41, 100.0%, 91.4-100.0)	(49/49, 100.0%, 92.7-100.0)	(43/43, 100.0%, 91.8-100.0)	(42/42, 100.0%, 91.6-100.0)	(42/42, 100.0%, 91.6-100.0)	(42/42, 100.0%, 91.6-100.0)	(41/41, 100.0%, 91.4-100.0)	(34/34, 100.0%, 89.8-100.0)	(35/35, 100.0%, 90.1-100.0)	(19/19, 100.0%, 83.2-100.0)	(571/571, 100.0%, 99.3-100.0)	N/A	N/A
	**A578**	(33/33, 100.0%, 89.6-100.0)	(40/40, 100.0%, 91.2-100.0)	(28/28, 100.0%, 87.9-100.0)	(37/37, 100.0%, 90.6-100.0)	(45/45, 100.0%, 92.1-100.0)	(42/42, 100.0%, 91.6-100.0)	(49/49, 100.0%, 92.7-100.0)	(43/43, 100.0%, 91.8-100.0)	(42/42, 100.0%, 91.6-100.0)	(42/42, 100.0%, 91.6-100.0)	(41/42, 97.6%, 87.7-99.6)	(41/41, 100.0%, 91.4-100.0)	(34/34, 100.0%, 89.8-100.0)	(35/35, 100.0%, 90.1-100.0)	(19/19, 100.0%, 83.2-100.0)	(571/572, 99.8%, 99.0-100.0)	1.264e+01	5.550e-01
	**C580**	(33/33, 100.0%, 89.6-100.0)	(40/40, 100.0%, 91.2-100.0)	(28/28, 100.0%, 87.9-100.0)	(37/37, 100.0%, 90.6-100.0)	(45/45, 100.0%, 92.1-100.0)	(42/42, 100.0%, 91.6-100.0)	(49/49, 100.0%, 92.7-100.0)	(43/43, 100.0%, 91.8-100.0)	(42/42, 100.0%, 91.6-100.0)	(42/42, 100.0%, 91.6-100.0)	(42/42, 100.0%, 91.6-100.0)	(41/41, 100.0%, 91.4-100.0)	(34/34, 100.0%, 89.8-100.0)	(35/35, 100.0%, 90.1-100.0)	(19/19, 100.0%, 83.2-100.0)	(572/572, 100.0%, 99.3-100.0)	N/A	N/A
	**D584**	(33/33, 100.0%, 89.6-100.0)	(40/40, 100.0%, 91.2-100.0)	(28/28, 100.0%, 87.9-100.0)	(37/37, 100.0%, 90.6-100.0)	(45/45, 100.0%, 92.1-100.0)	(42/42, 100.0%, 91.6-100.0)	(49/49, 100.0%, 92.7-100.0)	(43/43, 100.0%, 91.8-100.0)	(42/42, 100.0%, 91.6-100.0)	(42/42, 100.0%, 91.6-100.0)	(42/42, 100.0%, 91.6-100.0)	(41/41, 100.0%, 91.4-100.0)	(34/34, 100.0%, 89.8-100.0)	(35/35, 100.0%, 90.1-100.0)	(19/19, 100.0%, 83.2-100.0)	(572/572, 100.0%, 99.3-100.0)	N/A	N/A
	**R622**	(32/33, 97.0%, 84.7-99.5)	(40/40, 100.0%, 91.2-100.0)	(28/28, 100.0%, 87.9-100.0)	(37/37, 100.0%, 90.6-100.0)	(45/45, 100.0%, 92.1-100.0)	(42/42, 100.0%, 91.6-100.0)	(49/49, 100.0%, 92.7-100.0)	(42/43, 97.7%, 87.9-99.6)	(35/42, 83.3%, 69.4-91.7)	(42/42, 100.0%, 91.6-100.0)	(28/42, 66.7%, 51.6-79.0)	(39/41, 95.1%, 83.9-98.7)	(34/34, 100.0%, 89.8-100.0)	(18/35, 51.4%, 35.6-67.0)	(15/19, 78.9%, 56.7-91.5)	(515/572, 90.0%, 87.3-92.2)	1.45E+02	7.564e-24
	**A675**	(32/33, 97.0%, 84.7-99.5)	(40/40, 100.0%, 91.2-100.0)	(18/28, 64.3%, 45.8-79.3)	(37/37, 100.0%, 90.6-100.0)	(45/45, 100.0%, 92.1-100.0)	(41/42, 97.6%, 87.7-99.6)	(41/49, 83.7%, 71.0-91.5)	(43/43, 100.0%, 91.8-100.0)	(42/42, 100.0%, 91.6-100.0)	(42/42, 100.0%, 91.6-100.0)	(42/42, 100.0%, 91.6-100.0)	(39/41, 95.1%, 83.9-98.7)	(34/34, 100.0%, 89.8-100.0)	(18/35, 51.4%, 35.6-67.0)	(15/19, 78.9%, 56.7-91.5)	(562/572, 98.3%, 96.8-99.0)	7.154e+01	1.014e-09
Pfcytb	**I258**	(33/33, 100.0%, 89.6-100.0)	(40/40, 100.0%, 91.2-100.0)	(28/28, 100.0%, 87.9-100.0)	(37/37, 100.0%, 90.6-100.0)	(45/45, 100.0%, 92.1-100.0)	(42/42, 100.0%, 91.6-100.0)	(41/49, 83.7%, 71.0-91.5)	(43/43, 100.0%, 91.8-100.0)	(42/42, 100.0%, 91.6-100.0)	(42/42, 100.0%, 91.6-100.0)	(42/42, 100.0%, 91.6-100.0)	(39/41, 95.1%, 83.9-98.7)	(34/34, 100.0%, 89.8-100.0)	(35/35, 100.0%, 90.1-100.0)	(19/19, 100.0%, 83.2-100.0)	(562/572, 98.3%, 96.8-99.0)	7.154e+01	1.014e-09
	**Y268**	(33/33, 100.0%, 89.6-100.0)	(40/40, 100.0%, 91.2-100.0)	(28/28, 100.0%, 87.9-100.0)	(26/26, 100.0%, 87.1-100.0)	(45/45, 100.0%, 92.1-100.0)	(44/44, 100.0%, 92.0-100.0)	(44/44, 100.0%, 92.0-100.0)	(29/29, 100.0%, 88.3-100.0)	(30/30, 100.0%, 88.6-100.0)	(35/35, 100.0%, 90.1-100.0)	(38/38, 100.0%, 90.8-100.0)	(14/14, 100.0%, 78.5-100.0)	(33/33, 100.0%, 89.6-100.0)	(44/44, 100.0%, 92.0-100.0)	(19/19, 100.0%, 83.2-100.0)	(502/502, 100.0%, 99.2-100.0)	N/A	N/A

Geographic variation in SNP and haplotype frequencies was assessed using chi-squared tests (statistical significance: $p < 0.05$). N/A: Not Applicable: the Chi-square test could not be performed as the mutant allele was not observed in the study population. N indicates the number of samples successfully genotyped for each marker. The Overall N is the aggregate count across all regions. This value may differ by marker, as samples without genotype data at a specific locus are excluded from the prevalence calculation for that marker.

Supplementary Table 6. Sample IDs and metadata

ENA_id	Sample name	Collection year	District
ERS23986588	ACH-21-BB-42	2021	Batu
ERS23986587	ACH21-MK101	2021	Metema
ERS23986586	ACH-21-FB-043	2021	Fedis
ERS23986585	ACH-21-BD-11	2021	Batu
ERS23986584	ACH-21-FB-021	2021	Fedis
ERS23986583	ACH-21-FB-032	2021	Fedis
ERS23986582	ACH21-DO006	2021	Dasenech
ERS23986581	TG01-003	2019	T.Abergelle
ERS23986580	ACH-21-BB-31	2021	Batu
ERS23986579	ACH21-GU017	2021	Guba
ERS23986578	ACH21-AA041	2021	Amibara
ERS23986577	ACH22-PK008	2022	Pawe
ERS23986576	ACH21-DL064	2021	Dasenech
ERS23986575	GMTP052	2023	Gondar
ERS23986574	ACH21-FM070	2021	Fentale
ERS23986573	ACH-21-BB-39	2021	Batu
ERS23986572	ACH-21-BS-082	2021	Batu
ERS23986571	ACH21-DL069	2021	Dasenech
ERS23986570	ACH21-AA032	2021	Amibara
ERS23986569	ACH21-DO019	2021	Dasenech
ERS23986568	TG01-023	2019	T.Abergelle
ERS23986567	GMTP049	2023	Gondar
ERS23986566	ACH22-GU011	2022	Guba
ERS23986565	GMTP040	2023	Gondar
ERS23986564	ACH21-MA019	2022	Mizan
ERS23986563	ACH-21-BB-81	2021	Batu
ERS23986562	ACH-21-BS-012	2021	Batu
ERS23986561	ACH22-PF018	2022	Pawe
ERS23986560	ACH22-EE009	2022	Gambella
ERS23986559	ACH22-GA015	2022	Gambella
ERS23986558	ACH-21-DH-141	2021	Dera
ERS23986557	ACH21-DL083	2021	Dasenech
ERS23986556	ACH21-SH285	2021	Salamago
ERS23986555	ACH22-GA016	2022	Gambella
ERS23986554	ACH21-HH038	2021	K.Humera
ERS23986553	ACH22-PF009	2022	Pawe
ERS23986552	ACH-21-BS-027	2021	Batu
ERS23986551	ACH21-HH029	2021	K.Humera
ERS23986550	ACH21-HH004	2021	K.Humera
ERS23986549	TG01-020	2019	T.Abergelle
ERS23986548	ACH21-DL019	2021	Dasenech
ERS23986547	ACH-21-FB-071	2021	Fedis
ERS23986546	ACH-21-BS-008	2021	Batu
ERS23986545	ACH21-MK089	2021	Metema
ERS23986544	ACH21-AA049	2021	Amibara
ERS23986543	ACH22-GU013	2022	Guba
ERS23986542	ACH21-FM011	2021	Fentale
ERS23986541	ACH21-MA027	2022	Mizan
ERS23986540	ACH21-AA045	2021	Amibara
ERS23986539	ACH21-DL086	2021	Dasenech
ERS23986538	ACH22-GB016	2022	Gambella

ERS23986537	ACH21-GA036	2021	Gambella
ERS23986536	ACH-21-FB-004	2021	Fedis
ERS23986535	ACH21-FM046	2021	Fentale
ERS23986701	ACH21-AA026	2021	Amibara
ERS23986700	ACH-21-BS-032	2021	Batu
ERS23986699	ACH-21-FB-049	2021	Fedis
ERS23986698	ACH21-DO001	2021	Dasenech
ERS23986697	ACH-21-DH-130	2021	Dera
ERS23986696	ACH21-MA031	2022	Mizan
ERS23986695	ACH-21-FB-001	2021	Fedis
ERS23986694	ACH21-MA020	2022	Mizan
ERS23986693	ACH22-MK001	2022	Metema
ERS23986692	ACH21-AA039	2021	Amibara
ERS23986691	ACH21-AA010	2021	Amibara
ERS23986690	ACH21-SH031	2021	Salamago
ERS23986689	ACH21-FM079	2021	Fentale
ERS23986688	ACH-21-DH-159	2021	Dera
ERS23986687	GMTP003	2022	Gondar
ERS23986686	ACH21-MM018	2022	Mizan
ERS23986685	ACH21-FM054	2021	Fentale
ERS23986684	ACH21-GU227	2022	Guba
ERS23986683	ACH22-PK004	2022	Pawe
ERS23986534	TG01-021	2019	T.Abergelle
ERS23986533	ACH21-AA046	2021	Amibara
ERS23986532	ACH21-SH273	2021	Salamago
ERS23986531	ACH21-MK061	2021	Metema
ERS23986530	ACH-21-BS-081	2021	Batu
ERS23986529	ACH21-SH156	2021	Salamago
ERS23986528	ACH-21-BS-028	2021	Batu
ERS23986527	ACH-21-BS-053	2021	Batu
ERS23986526	ACH21-GU214	2021	Guba
ERS23986525	ACH21-GU204	2021	Guba
ERS23986524	ACH21-GB061	2021	Gambella
ERS23986523	ACH21-GB022	2021	Gambella
ERS23986522	ACH22-PK011	2022	Pawe
ERS23986521	ACH21-SH235	2021	Salamago
ERS23986682	ACH21-GA029	2021	Gambella
ERS23986681	TG01-002	2019	T.Abergelle
ERS23986680	ACH21-DO151	2021	Dasenech
ERS23986679	ACH-21-BB-79	2021	Batu
ERS23986678	ACH21-AA030	2021	Amibara
ERS23986677	ACH21-FM096	2021	Fentale
ERS23986676	ACH21-MY296	2021	Metema
ERS23986675	ACH21-AA003	2021	Amibara
ERS23986674	ACH21-SH160	2021	Salamago
ERS23986673	ACH21-GA013	2021	Gambella
ERS23986672	GMTP007	2022	Gondar
ERS23986671	ACH21-GU202	2021	Guba
ERS23986670	ACH21-DL020	2021	Dasenech
ERS23986669	ACH22-PK006	2022	Pawe
ERS23986668	ACH21-MM034	2022	Mizan
ERS23986667	ACH-21-BS-015	2021	Batu
ERS23986666	ACH21-MA032	2022	Mizan

ERS23986665	ACH21-AA008	2021	Amibara
ERS23986664	TG01-024	2019	T.Abergelle
ERS23986663	ACH-21-DH-149	2021	Dera
ERS23986662	ACH-21-DH-135	2021	Dera
ERS23986661	ACH-21-FB-026	2021	Fedis
ERS23986660	ACH21-PF005	2021	Pawe
ERS23986659	ACH21-SK065	2021	Salamago
ERS23986658	TG01-018	2019	T.Abergelle
ERS23986657	ACH-21-FB-014	2021	Fedis
ERS23986656	ACH21-FM040	2021	Fentale
ERS23986655	ACH21-SH169	2021	Salamago
ERS23986654	ACH21-DL012	2021	Dasenech
ERS23986653	ACH22-PK016	2022	Pawe
ERS23986652	ACH21-MM036	2022	Mizan
ERS23986651	ACH-21-DH-180	2021	Dera
ERS23986650	ACH21-FM055	2021	Fentale
ERS23986649	ACH21-MM037	2022	Mizan
ERS23986648	ACH21-FM081	2021	Fentale
ERS23986647	ACH22-MY019	2022	Metema
ERS23986646	ACH21-AA009	2021	Amibara
ERS23986645	ACH-21-DH-184	2021	Dera
ERS23986644	ACH-21-FB-072	2021	Fedis
ERS23986643	ACH21-MM014	2022	Mizan
ERS23986642	ACH22-GU007	2022	Guba
ERS23986641	ACH-21-BD-28	2021	Batu
ERS23986640	GMTP055	2023	Gondar
ERS23986639	ACH21-HH043	2021	K.Humera
ERS23986638	ACH-21-FB-082	2021	Fedis
ERS23986637	ACH21-AA037	2021	Amibara
ERS23986636	ACH22-PM009	2022	Pawe
ERS23986635	ACH21-GU222	2021	Guba
ERS23986634	ACH-21-BB-57	2021	Batu
ERS23986633	ACH-21-FB-047	2021	Fedis
ERS23986632	ACH22-PK010	2022	Pawe
ERS23986631	ACH22-GU010	2022	Guba
ERS23986630	ACH-21-BD-18	2021	Batu
ERS23986629	ACH-21-DH-136	2021	Dera
ERS23986628	TG01-008	2019	T.Abergelle
ERS23986627	GMTP011	2022	Gondar
ERS23986626	ACH21-MM002	2022	Mizan
ERS23986625	ACH21-PF003	2021	Pawe
ERS23986624	GMTP044	2023	Gondar
ERS23986623	ACH-21-SK-017	2021	Salamago
ERS23986622	ACH21-FM098	2021	Fentale
ERS23986621	ACH21-AA004	2021	Amibara
ERS23986620	ACH21-HH046	2021	K.Humera
ERS23986619	ACH21-MY292	2021	Metema
ERS23986618	ACH21-DL002	2021	Dasenech
ERS23986617	ACH21-SH102	2021	Salamago
ERS23986616	ACH21-MY290	2021	Metema
ERS23986615	ACH21-AA006	2021	Amibara
ERS23986614	ACH-21-FB-038	2021	Fedis
ERS23986613	ACH-21-FB-059	2021	Fedis

ERS23986612	ACH21-HH041	2021	K.Humera
ERS23986611	ACH21-GU228	2022	Guba
ERS23986610	ACH21-GU020	2021	Guba
ERS23986609	GMTP042	2023	Gondar
ERS23986608	ACH22-PM005	2022	Pawe
ERS23986607	ACH22-PF008	2022	Pawe
ERS23986606	ACH21-MK105	2021	Metema
ERS23986605	ACH21-GU001	2021	Guba
ERS23986604	ACH21-GU215	2021	Guba
ERS23986603	ACH22-PF005	2022	Pawe
ERS23986602	ACH22-PF017	2022	Pawe
ERS23986601	ACH-21-FB-051	2021	Fedis
ERS23986600	ACH-21-FB-009	2021	Fedis
ERS23986599	ACH-21-BB-68	2021	Batu
ERS23986598	ACH21-HH023	2021	K.Humera
ERS23986597	ACH21-MY293	2021	Metema
ERS23986596	ACH22-PF003	2022	Pawe
ERS23986595	ACH21-FM023	2021	Fentale
ERS23986594	ACH-21-FB-040	2021	Fedis
ERS23986593	ACH21-PM017	2021	Pawe
ERS23986592	ACH21-FM062	2021	Fentale
ERS23986591	ACH21-GU007	2021	Guba
ERS23986590	ACH21-SH047	2021	Salamago
ERS23986589	ACH-21-DH-181	2021	Dera
ERS23986421	ACH21-GU233	2022	Guba
ERS23986420	ACH22-PK017	2022	Pawe
ERS23986419	ACH22-GU019	2022	Guba
ERS23986418	ACH22-GA005	2022	Gambella
ERS23986417	ACH21-GU019	2021	Guba
ERS23986416	ACH21-GA034	2021	Gambella
ERS23986415	ACH21-DO005	2021	Dasenech
ERS23986414	ACH21-SH132	2021	Salamago
ERS23986413	GMTP050	2023	Gondar
ERS23986412	ACH21-FM044	2021	Fentale
ERS23986411	GMTP004	2022	Gondar
ERS23986410	ACH22-GA003	2022	Gambella
ERS23986409	ACH22-GB014	2022	Gambella
ERS23986408	ACH-21-DH-200	2021	Dera
ERS23986407	ACH22-PK003	2022	Pawe
ERS23986406	ACH22-PF012	2022	Pawe
ERS23986405	ACH21-MM015	2022	Mizan
ERS23986404	ACH-21-FB-006	2021	Fedis
ERS23986403	ACH21-HH003	2021	K.Humera
ERS23986402	ACH21-MK076	2021	Metema
ERS23986401	ACH22-GB005	2022	Gambella
ERS23986400	ACH-21-BB-46	2021	Batu
ERS23986399	ACH21-GA008	2021	Gambella
ERS23986398	ACH21-DO008	2021	Dasenech
ERS23986397	ACH21-MK037	2021	Metema
ERS23986396	ACH-21-BS-052	2021	Batu
ERS23986395	ACH21-GU206	2021	Guba
ERS23986394	ACH21-GA027	2021	Gambella
ERS23986393	ACH21-SH295	2021	Salamago

ERS23986392	ACH21-FM052	2021	Fentale
ERS23986391	ACH21-DO165	2021	Dasenech
ERS23986390	ACH21-SH287	2021	Salamago
ERS23986389	ACH22-MK003	2022	Metema
ERS23986388	ACH21-HH044	2021	K.Humera
ERS23986387	ACH21-FM088	2021	Fentale
ERS23986386	ACH21-AA024	2021	Amibara
ERS23986385	ACH-21-DH-201	2021	Dera
ERS23986520	ACH21-MM042	2022	Mizan
ERS23986519	ACH21-MK102	2021	Metema
ERS23986518	ACH21-HH006	2021	K.Humera
ERS23986517	ACH21-SH241	2021	Salamago
ERS23986516	ACH-21-DH-167	2021	Dera
ERS23986515	ACH22-MK008	2022	Metema
ERS23986514	ACH-21-FB-029	2021	Fedis
ERS23986513	GMTP027	2022	Gondar
ERS23986512	ACH21-DO152	2021	Dasenech
ERS23986511	ACH-21-BS-084	2021	Batu
ERS23986510	ACH21-GA022	2021	Gambella
ERS23986509	ACH-21-DH-185	2021	Dera
ERS23986508	ACH-21-BS-048	2021	Batu
ERS23986507	ACH21-MA026	2022	Mizan
ERS23986506	ACH21-FM049	2021	Fentale
ERS23986505	ACH22-MY020	2022	Metema
ERS23986504	ACH21-MA040	2022	Mizan
ERS23986503	ACH21-GA009	2021	Gambella
ERS23986502	GMTP056	2023	Gondar
ERS23986501	ACH21-FM064	2021	Fentale
ERS23986500	GMTP047	2023	Gondar
ERS23986499	TG01-010	2019	T.Abergelle
ERS23986498	ACH21-DO022	2021	Dasenech
ERS23986497	GMTP002	2022	Gondar
ERS23986496	GMTP022	2022	Gondar
ERS23986495	GMTP020	2022	Gondar
ERS23986494	GMTP048	2023	Gondar
ERS23986493	ACH-21-FB-079	2021	Fedis
ERS23986492	ACH21-HH028	2021	K.Humera
ERS23986491	ACH22-EE003	2022	Gambella
ERS23986490	ACH21-SH072	2021	Salamago
ERS23986489	ACH21-GU225	2022	Guba
ERS23986488	ACH-21-SK-018	2021	Salamago
ERS23986487	ACH22-PK007	2022	Pawe
ERS23986486	ACH-21-FB-036	2021	Fedis
ERS23986485	ACH22-PK015	2022	Pawe
ERS23986484	ACH21-HH025	2021	K.Humera
ERS23986483	ACH-21-BB-37	2021	Batu
ERS23986482	ACH22-MY007	2022	Metema
ERS23986481	ACH22-PF004	2022	Pawe
ERS23986480	GMTP031	2022	Gondar
ERS23986479	TG01-012	2019	T.Abergelle
ERS23986478	GMTP009	2022	Gondar
ERS23986477	ACH21-SH237	2021	Salamago
ERS23986476	ACH21-MK020	2021	Metema

ERS23986475	ACH21-AA048	2021	Amibara
ERS23986474	ACH21-GU210	2021	Guba
ERS23986473	ACH21-HH027	2021	K.Humera
ERS23986472	ACH22-GU006	2022	Guba
ERS23986471	ACH21-MY282	2021	Metema
ERS23986470	ACH21-FM071	2021	Fentale
ERS23986469	ACH21-SH238	2021	Salamago
ERS23986468	ACH21-MA017	2022	Mizan
ERS23986467	ACH21-SH142	2021	Salamago
ERS23986466	ACH21-MM043	2022	Mizan
ERS23986465	ACH21-FM083	2021	Fentale
ERS23986464	ACH-21-DH-193	2021	Dera
ERS23986463	ACH21-MM016	2022	Mizan
ERS23986462	ACH-21-DH-147	2021	Dera
ERS23986461	GMTP021	2022	Gondar
ERS23986460	ACH-21-FB-061	2021	Fedis
ERS23986459	ACH21-GU213	2021	Guba
ERS23986458	GMTP051	2023	Gondar
ERS23986457	ACH22-GU004	2022	Guba
ERS23986456	ACH21-MA010	2022	Mizan
ERS23986455	ACH21-MK103	2021	Metema
ERS23986454	ACH-21-FB-008	2021	Fedis
ERS23986453	ACH22-MY006	2022	Metema
ERS23986452	ACH-21-FB-003	2021	Fedis
ERS23986451	ACH21-DL017	2021	Dasenech
ERS23986450	ACH21-DO004	2021	Dasenech
ERS23986449	ACH21-MY291	2021	Metema
ERS23986448	GMTP029	2022	Gondar
ERS23986447	ACH22-GU005	2022	Guba
ERS23986446	GMTP041	2023	Gondar
ERS23986445	GMTP001	2022	Gondar
ERS23986444	ACH21-AA033	2021	Amibara
ERS23986443	ACH-21-DH-189	2021	Dera
ERS23986442	ACH21-SH122	2021	Salamago
ERS23986441	GMTP032	2022	Gondar
ERS23986440	ACH21-SH003	2021	Salamago
ERS23986439	ACH-21-FB-050	2021	Fedis
ERS23986438	ACH-21-DH-138	2021	Dera
ERS23986437	ACH21-HH033	2021	K.Humera
ERS23986436	ACH-21-DH-160	2021	Dera
ERS23986435	ACH21-SH264	2021	Salamago
ERS23986434	ACH22-GB020	2022	Gambella
ERS23986433	ACH21-DL007	2021	Dasenech
ERS23986432	ACH21-FM086	2021	Fentale
ERS23986431	ACH21-DO007	2021	Dasenech
ERS23986430	ACH21-FM059	2021	Fentale
ERS23986429	ACH21-GB120	2021	Gambella
ERS23986428	ACH21-HH015	2021	K.Humera
ERS23986427	ACH21-GU008	2021	Guba
ERS23986426	ACH21-MM040	2022	Mizan
ERS23986425	ACH21-GU004	2021	Guba
ERS23986424	ACH21-GB116	2021	Gambella
ERS23986423	ACH21-MK032	2021	Metema

ERS23986422	ACH21-HH036	2021	K.Humera
ERS23986254	ACH21-HH002	2021	K.Humera
ERS23986384	ACH-21-FB-024	2021	Fedis
ERS23986383	ACH21-AA023	2021	Amibara
ERS23986253	ACH21-PF006	2021	Pawe
ERS23986252	ACH21-DL004	2021	Dasenech
ERS23986251	ACH-21-BS-074	2021	Batu
ERS23986250	ACH21-HH040	2021	K.Humera
ERS23986249	ACH22-MK007	2022	Metema
ERS23986248	ACH-21-FB-081	2021	Fedis
ERS23986247	ACH21-HH013	2021	K.Humera
ERS23986246	ACH-21-FB-084	2021	Fedis
ERS23986244	ACH21-HH030	2021	K.Humera
ERS23986243	ACH21-FM097	2021	Fentale
ERS23986242	ACH22-PK005	2022	Pawe
ERS23986241	ACH21-MM044	2022	Mizan
ERS23986240	ACH21-MA012	2022	Mizan
ERS23986239	ACH21-GA028	2021	Gambella
ERS23986238	ACH21-AA025	2021	Amibara
ERS23986237	ACH21-SH044	2021	Salamago
ERS23986236	GMTP024	2022	Gondar
ERS23986235	ACH22-PF011	2022	Pawe
ERS23986234	ACH-21-FB-023	2021	Fedis
ERS23986382	TG01-019	2019	T.Abergelle
ERS23986381	ACH22-PF002	2022	Pawe
ERS23986380	GMTP053	2023	Gondar
ERS23986379	ACH21-FM074	2021	Fentale
ERS23986377	ACH21-HH026	2021	K.Humera
ERS23986375	ACH22-GA010	2022	Gambella
ERS23986373	ACH22-EE008	2022	Gambella
ERS23986371	GMTP054	2023	Gondar
ERS23986370	ACH21-HH001	2021	K.Humera
ERS23986367	ACH-21-FB-028	2021	Fedis
ERS23986359	ACH22-MK018	2022	Metema
ERS23986356	ACH21-DO154	2021	Dasenech
ERS23986355	ACH21-GU223	2021	Guba
ERS23986354	ACH21-MA005	2022	Mizan
ERS23986353	ACH21-GU011	2021	Guba
ERS23986352	ACH22-MY015	2022	Metema
ERS23986351	ACH21-MM017	2022	Mizan
ERS23986350	ACH22-GD005	2022	Gambella
ERS23986349	TG01-005	2019	T.Abergelle
ERS23986233	ACH21-MK118	2021	Metema
ERS23986232	ACH21-GB073	2021	Gambella
ERS23986231	ACH21-DO156	2021	Dasenech
ERS23986230	ACH-21-BS-003	2021	Batu
ERS23986229	ACH21-GU003	2021	Guba
ERS23986228	ACH22-GA020	2022	Gambella
ERS23986227	ACH21-GU218	2021	Guba
ERS23986226	ACH-21-BS-009	2021	Batu
ERS23986225	ACH22-PF019	2022	Pawe
ERS23986224	ACH22-GB006	2022	Gambella
ERS23986223	ACH21-DL089	2021	Dasenech

ERS23986222	ACH21-GU209	2021	Guba
ERS23986221	ACH21-GB090	2021	Gambella
ERS23986220	ACH21-MY297	2021	Metema
ERS23986219	ACH21-HH021	2021	K.Humera
ERS23986218	ACH-21-FB-085	2021	Fedis
ERS23986217	ACH-21-DH-204	2021	Dera
ERS23986216	ACH-21-BS-077	2021	Batu
ERS23986215	ACH22-GA019	2022	Gambella
ERS23986348	ACH21-HH034	2021	K.Humera
ERS23986347	ACH21-MA039	2022	Mizan
ERS23986346	ACH21-MK057	2021	Metema
ERS23986345	ACH21-GU216	2021	Guba
ERS23986344	ACH21-AA034	2021	Amibara
ERS23986343	ACH-21-DH-196	2021	Dera
ERS23986342	ACH21-DO023	2021	Dasenech
ERS23986341	ACH21-DO012	2021	Dasenech
ERS23986340	ACH-21-BB-75	2021	Batu
ERS23986339	ACH21-HH024	2021	K.Humera
ERS23986338	ACH21-HH009	2021	K.Humera
ERS23986337	ACH-21-DH-145	2021	Dera
ERS23986336	ACH21-MM005	2022	Mizan
ERS23986335	ACH21-DL015	2021	Dasenech
ERS23986334	ACH-21-BS-075	2021	Batu
ERS23986333	ACH21-MA033	2022	Mizan
ERS23986332	ACH21-GU203	2021	Guba
ERS23986331	ACH-21-SH-070	2021	Salamago
ERS23986330	ACH21-GU219	2021	Guba
ERS23986214	ACH21-PF010	2021	Pawe
ERS23986213	GMTP006	2022	Gondar
ERS23986212	ACH21-MK054	2021	Metema
ERS23986211	ACH21-MM004	2022	Mizan
ERS23986210	ACH21-SH250	2021	Salamago
ERS23986209	ACH-21-BB-92	2021	Batu
ERS23986208	ACH21-FM012	2021	Fentale
ERS23986207	ACH22-GB008	2022	Gambella
ERS23986206	ACH21-FM095	2021	Fentale
ERS23986205	ACH22-GU015	2022	Guba
ERS23986204	ACH-21-FB-078	2021	Fedis
ERS23986203	ACH21-HH012	2021	K.Humera
ERS23986202	GMTP039	2023	Gondar
ERS23986201	ACH21-AA044	2021	Amibara
ERS23986200	ACH21-MK038	2021	Metema
ERS23986199	ACH21-HH035	2021	K.Humera
ERS23986198	ACH21-HH019	2021	K.Humera
ERS23986197	ACH-21-FB-067	2021	Fedis
ERS23986196	ACH21-AA042	2021	Amibara
ERS23986329	ACH-21-BS-083	2021	Batu
ERS23986328	GMTP017	2022	Gondar
ERS23986327	ACH21-AA031	2021	Amibara
ERS23986326	ACH-21-FB-053	2021	Fedis
ERS23986325	ACH22-PK021	2022	Pawe
ERS23986324	ACH21-DO011	2021	Dasenech
ERS23986323	ACH22-PF016	2022	Pawe

ERS23986322	ACH21-HH005	2021	K.Humera
ERS23986321	ACH21-GA024	2021	Gambella
ERS23986320	ACH-21-BS-024	2021	Batu
ERS23986319	ACH22-EE005	2022	Gambella
ERS23986318	ACH-21-BD-27	2021	Batu
ERS23986317	ACH21-AA001	2021	Amibara
ERS23986316	ACH-21-SH-065	2021	Salamago
ERS23986315	ACH21-MM033	2022	Mizan
ERS23986314	ACH-21-FB-064	2021	Fedis
ERS23986313	ACH21-MK080	2021	Metema
ERS23986312	ACH22-PK019	2022	Pawe
ERS23986311	ACH21-MM010	2022	Mizan
ERS23986195	ACH22-GU014	2022	Guba
ERS23986194	ACH-21-BB-86	2021	Batu
ERS23986193	ACH21-FM092	2021	Fentale
ERS23986192	ACH21-FM089	2021	Fentale
ERS23986191	ACH21-HH007	2021	K.Humera
ERS23986190	ACH21-DO002	2021	Dasenech
ERS23986189	ACH21-GB100	2021	Gambella
ERS23986188	ACH-21-BB-33	2021	Batu
ERS23986187	GMTP015	2022	Gondar
ERS23986186	ACH21-SH185	2021	Salamago
ERS23986185	ACH21-AA012	2021	Amibara
ERS23986184	ACH21-MA015	2022	Mizan
ERS23986183	ACH21-FM033	2021	Fentale
ERS23986182	ACH21-FM025	2021	Fentale
ERS23986181	ACH21-GU018	2021	Guba
ERS23986180	ACH-21-BS-070	2021	Batu
ERS23986179	ACH21-MK055	2021	Metema
ERS23986178	ACH21-GU016	2021	Guba
ERS23986177	ACH21-SH120	2021	Salamago
ERS23986310	ACH21-AA007	2021	Amibara
ERS23986309	ACH21-FM024	2021	Fentale
ERS23986308	ACH21-AA028	2021	Amibara
ERS23986307	ACH-21-FB-016	2021	Fedis
ERS23986306	ACH21-AA029	2021	Amibara
ERS23986305	ACH22-PK001	2022	Pawe
ERS23986304	ACH-21-SH-069	2021	Salamago
ERS23986303	ACH-21-BB-89	2021	Batu
ERS23986302	ACH22-GA018	2022	Gambella
ERS23986301	ACH21-GU006	2021	Guba
ERS23986300	ACH21-DL067	2021	Dasenech
ERS23986299	ACH-21-DH-137	2021	Dera
ERS23986298	TG01-011	2019	T.Abergelle
ERS23986297	ACH22-MY011	2022	Metema
ERS23986296	ACH21-MK031	2021	Metema
ERS23986295	ACH21-HH018	2021	K.Humera
ERS23986294	ACH21-PF011	2021	Pawe
ERS23986293	ACH-21-FB-012	2021	Fedis
ERS23986292	TG01-016	2019	T.Abergelle
ERS23986176	ACH21-DO021	2021	Dasenech
ERS23986175	ACH21-AA018	2021	Amibara
ERS23986174	ACH22-GD007	2022	Gambella

ERS23986173	TG01-017	2019	T.Abergelle
ERS23986291	ACH22-PM003	2022	Pawe
ERS23986290	ACH21-PF001	2021	Pawe
ERS23986289	ACH21-MK028	2021	Metema
ERS23986288	ACH22-MK005	2022	Metema
ERS23986287	ACH21-FM014	2021	Fentale
ERS23986286	ACH21-DO003	2021	Dasenech
ERS23986285	ACH21-AA005	2021	Amibara
ERS23986284	ACH21-MA036	2022	Mizan
ERS23986283	GMTP046	2023	Gondar
ERS23986282	ACH21-AA015	2021	Amibara
ERS23986281	ACH21-DL022	2021	Dasenech
ERS23986280	ACH21-FM051	2021	Fentale
ERS23986279	ACH-21-FB-027	2021	Fedis
ERS23986278	ACH22-GA008	2022	Gambella
ERS23986277	ACH21-FM015	2021	Fentale
ERS23986276	ACH21-AA043	2021	Amibara
ERS23986275	ACH22-PK020	2022	Pawe
ERS23986274	ACH21-HH037	2021	K.Humera
ERS23986273	ACH21-GB025	2021	Gambella
ERS23986272	ACH21-SH155	2021	Salamago
ERS23986271	GMTP034	2022	Gondar
ERS23986270	ACH21-MM021	2022	Mizan
ERS23986269	ACH21-DL014	2021	Dasenech
ERS23986268	ACH21-SH257	2021	Salamago
ERS23986267	ACH-21-BB-53	2021	Batu
ERS23986266	ACH21-GA026	2021	Gambella
ERS23986265	ACH21-MM001	2022	Mizan
ERS23986264	ACH21-AA047	2021	Amibara
ERS23986263	ACH21-HH008	2021	K.Humera
ERS23986262	ACH21-SH260	2021	Salamago
ERS23986261	ACH-21-FB-031	2021	Fedis
ERS23986260	ACH-21-BB-77	2021	Batu
ERS23986259	GMTP025	2022	Gondar
ERS23986258	ACH21-MM012	2022	Mizan
ERS23986257	ACH21-MA030	2022	Mizan
ERS23986256	ACH21-HH016	2021	K.Humera
ERS23986255	GMTP026	2022	Gondar
ERS23986082	TG01-022	2019	T.Abergelle
ERS23986083	ACH21-MY288	2021	Metema
ERS23986084	ACH21-DL005	2021	Dasenech
ERS23986085	ACH21-FM028	2021	Fentale
ERS23986086	ACH-21-DH-132	2021	Dera
ERS23986087	ACH22-PK013	2022	Pawe
ERS23986088	ACH22-MK010	2022	Metema
ERS23986089	ACH21-HH042	2021	K.Humera
ERS23986090	ACH21-FM027	2021	Fentale
ERS23986091	ACH21-HH022	2021	K.Humera
ERS23986092	ACH21-MY281	2021	Metema
ERS23986093	ACH-21-DH-144	2021	Dera
ERS23986094	ACH21-HH017	2021	K.Humera
ERS23986095	ACH21-HH031	2021	K.Humera
ERS23986096	ACH21-MM038	2022	Mizan

ERS23986097	ACH21-GA030	2021	Gambella
ERS23986098	GMTP008	2022	Gondar
ERS23986099	ACH-21-FB-077	2021	Fedis
ERS23986120	ACH21-MM009	2022	Mizan
ERS23986121	ACH22-PK012	2022	Pawe
ERS23986122	ACH-21-FB-057	2021	Fedis
ERS23986123	ACH-21-BS-080	2021	Batu
ERS23986124	ACH-21-DH-203	2021	Dera
ERS23986125	ACH21-FM060	2021	Fentale
ERS23986126	ACH21-PF008	2021	Pawe
ERS23986127	ACH21-SH103	2021	Salamago
ERS23986128	ACH-21-FB-088	2021	Fedis
ERS23986129	ACH21-SK002	2021	Salamago
ERS23986130	ACH21-GB089	2021	Gambella
ERS23986131	ACH21-AA035	2021	Amibara
ERS23986132	ACH21-GB069	2021	Gambella
ERS23986133	ACH22-GD003	2022	Gambella
ERS23986134	ACH21-AA011	2021	Amibara
ERS23986135	GMTP013	2022	Gondar
ERS23986136	ACH21-GB004	2021	Gambella
ERS23986137	ACH21-FM026	2021	Fentale
ERS23986138	ACH-21-BB-67	2021	Batu
ERS23986139	ACH-21-DH-133	2021	Dera
ERS23986140	ACH21-FM066	2021	Fentale
ERS23986141	ACH21-DO009	2021	Dasenech
ERS23986142	ACH21-SK070	2021	Salamago
ERS23986143	ACH22-MY004	2022	Metema
ERS23986144	ACH21-MM041	2022	Mizan
ERS23986145	ACH22-GA017	2022	Gambella
ERS23986146	ACH22-GD001	2022	Gambella
ERS23986147	GMTP033	2022	Gondar
ERS23986148	ACH22-GB007	2022	Gambella
ERS23986149	GMTP005	2022	Gondar
ERS23986150	ACH21-FM058	2021	Fentale
ERS23986151	ACH-21-FB-080	2021	Fedis
ERS23986152	ACH21-HH039	2021	K.Humera
ERS23986153	ACH21-FM034	2021	Fentale
ERS23986154	ACH21-SH137	2021	Salamago
ERS23986155	GMTP035	2022	Gondar
ERS23986156	ACH21-PM007	2021	Pawe
ERS23986157	ACH21-HH020	2021	K.Humera
ERS23986158	ACH21-GU014	2021	Guba
ERS23986159	GMTP018	2022	Gondar
ERS23986160	GMTP045	2023	Gondar
ERS23986161	ACH21-GU207	2021	Guba
ERS23986162	ACH-21-FB-011	2021	Fedis
ERS23986163	ACH-21-DH-188	2021	Dera
ERS23986164	ACH21-GB094	2021	Gambella
ERS23986165	ACH21-GU010	2021	Guba
ERS23986166	ACH21-AA022	2021	Amibara
ERS23986167	ACH-21-BS-076	2021	Batu
ERS23986168	ACH21-AA014	2021	Amibara
ERS23986169	ACH21-MA011	2022	Mizan

ERS23986170	ACH-21-FB-063	2021	Fedis
ERS23986171	ACH-21-FB-044	2021	Fedis
ERS23986172	GMTP037	2022	Gondar
ERS23986100	ACH-21-FB-042	2021	Fedis
ERS23986101	ACH21-FM069	2021	Fentale
ERS23986102	ACH21-HH011	2021	K.Humera
ERS23986103	ACH21-FM050	2021	Fentale
ERS23986104	ACH21-FM094	2021	Fentale
ERS23986105	ACH21-MM039	2022	Mizan
ERS23986106	ACH21-GA019	2021	Gambella
ERS23986107	ACH22-MK020	2022	Metema
ERS23986108	ACH21-DL021	2021	Dasenech
ERS23986109	TG01-009	2019	T.Abergelle
ERS23986110	ACH-21-BD-05	2021	Batu
ERS23986111	TG01-028	2019	T.Abergelle
ERS23986112	ACH21-MA023	2022	Mizan
ERS23986113	GMTP030	2022	Gondar
ERS23986114	GMTP016	2022	Gondar
ERS23986115	TG01-001	2019	T.Abergelle
ERS23986116	ACH21-AA036	2021	Amibara
ERS23986117	ACH-21-BB-91	2021	Batu
ERS23986118	ACH21-FM080	2021	Fentale
ERS23986119	ACH-21-DH-148	2021	Dera

REFERENCES

1. Peterson DS, Walliker D, Wellems TE. Evidence that a point mutation in dihydrofolate reductase-thymidylate synthase confers resistance to pyrimethamine in falciparum malaria. Proc Natl Acad Sci USA. 1988 Dec;85(23):9114–8.

2. Plowe CV, Cortese JF, Djimde A, Nwanyanwu OC, Watkins WM, Winstanley PA, et al. Mutations in Plasmodium falciparum dihydrofolate reductase and dihydropteroate synthase and epidemiologic patterns of pyrimethamine-sulfadoxine use and resistance. J Infect Dis. 1997 Dec;176(6):1590–6.

3. Brhane BG, Fola AA, Nigussie H, Leonetti A, Kassa M, Hailgiorgis H, et al. Rising prevalence of Plasmodium falciparum Artemisinin partial resistance mutations in Ethiopia. Commun Med. 2025 July 18;5(1):297.

4. Kublin JG, Dzinjalamala FK, Kamwendo DD, Malkin EM, Cortese JF, Martino LM, et al. Molecular Markers for Failure of Sulfadoxine-Pyrimethamine and Chlorproguanil-Dapsone Treatment of *Plasmodium falciparum* Malaria. J INFECT DIS. 2002 Feb;185(3):380–8.

5. Spalding MD, Eyase FL, Akala HM, Bedno SA, Prigge ST, Coldren RL, et al. Increased prevalence of the pfdhfr/phdhps quintuple mutant and rapid emergence of pfdhps resistance mutations at codons 581 and 613 in Kisumu, Kenya. Malar J. 2010 Nov 24;9:338.

6. White NFD, Whitton G, Wasakul V, Amenga-Etego L, Dara A, Andrianaranjaka V, et al. Disparate co-evolution and prevalence of sulfadoxine and pyrimethamine resistance alleles and haplotypes at dhfr and dhps genes across Africa. Sci Rep. 2025 Apr 17;15(1):13222.

7. Juliano JJ, Giesbrecht DJ, Simkin A, Fola AA, Lyimo BM, Pereus D, et al. Prevalence of mutations associated with artemisinin partial resistance and sulfadoxine–pyrimethamine resistance in 13 regions in Tanzania in 2021: a cross-sectional survey. The Lancet Microbe. 2024;5(10).

8. Roper C, Pearce R, Bredenkamp B, Gumede J, Drakeley C, Mosha F, et al. Antifolate antimalarial resistance in southeast Africa: a population-based analysis. Lancet. 2003 Apr 5;361(9364):1174–81.

SUPPLEMENTARY PROTOCOL

Multiplex PCR for Amplifying *P. falciparum* Antimalarial Drug Resistance Markers

Introduction

This protocol describes two multiplex polymerase chain reactions (PCR) to amplify seven key genetic regions associated with antimalarial drug resistance in *Plasmodium falciparum*. Assay I targeted full-length *kelch13*, *mdr1*, and *cytb* using Platinum™ Taq DNA Polymerase High Fidelity. Assay II targeted full-length *crt*, *dhfr*, *dhps*, and the mitochondrial genome (*Mt*) using Phusion® High-Fidelity DNA Polymerase. The method was designed for use with purified DNA from patient samples and includes initial quality control guidance based on qPCR Ct values. Amplified products are typically verified by agarose gel electrophoresis prior to downstream applications such as sequencing for resistance allele identification. This approach facilitates the efficient molecular surveillance of drug resistance patterns.

MATERIALS

REAGENTS

- Purified *P. falciparum* genomic DNA from patient samples (inhibitor-free recommended)
- *P. falciparum* 3D7 genomic DNA standard (e.g., ATCC/MR4, Cat# MRA-102G) or equivalent (for positive control/assay validation, optional)
- Nuclease-free water (molecular biology grade, Qiagen, Cat. # 129114)
- **Multiplex Assay I Reagents:**
  - Platinum™ Taq DNA Polymerase High Fidelity (5 U/µL) (Thermo Fisher Scientific, Cat# 11304029)
  - 10X High Fidelity PCR Buffer (supplied with Platinum™ Taq)
  - 50 mM $MgSO_4$ (supplied with Platinum™ Taq)
  - KB Extender (supplied with Platinum™ Taq)
  - dNTP Mix (10 mM each of dATP, dCTP, dGTP, and dTTP) (e.g., NEB, Cat# N0447S)
  - Primers for Assay I (*pfk13*, *pfmdr1*, *pfcytb* - see **Reagent Setup, Table 1**)
- **Multiplex Assay II Reagents:**
  - Phusion® High-Fidelity DNA Polymerase (2 U/µL) (NEB, Cat# M0530S/L)
  - 5X Phusion HF Buffer (supplied with Phusion® Pol) *or* 5X Phusion GC Buffer (NEB, Cat# B0518S / B0519S) (HF Buffer used in the example protocol)
  - dNTP Mix (10 mM each, as above)
  - DMSO (Optional, supplied with Phusion® Pol, e.g., NEB Cat# B0515A)
  - Primers for Assay II (*pfcrt*, *pfdhfr*, *pfdhps*, *pfMt* - pfMt-see **Reagent Setup, Table 2**)
- **Gel Electrophoresis Reagents:**
  - Agarose (molecular biology grade, e.g., Invitrogen, Cat# 16500500)
  - 10X TBE Buffer (e.g., Thermo Fisher Scientific, Cat# AM9863) or Tris base, boric acid, and EDTA for preparing custom buffer.
  - 6X DNA Loading Dye (e.g., NEB, Cat# B7024S)
  - Ethidium Bromide (EtBr) solution (10 mg/mL) (e.g., Sigma-Aldrich, Cat# E1510) **CAUTION:** Mutagen. *or* an alternative safer nucleic acid stain (SYBR™ Safe DNA Gel Stain, Invitrogen, Cat# S33102).
  - 1 kb Plus DNA Ladder (e.g., Invitrogen, Cat# 10787018) or similar suitable range ladder.

EQUIPMENT

- Micropipettes (P10, P200, P1000)
- Sterile filtered pipette tips
- Microcentrifuge
- Vortex mixer

- Thermocycler
- PCR cooler racks (-20°C) or ice buckets
- UV workstations (separate for master mix preparation and template addition)
- Gel electrophoresis system (casting tray, chamber, power supply)
- UV transilluminator or gel documentation system (appropriate for chosen stain)
- Microwave oven or heating plate
- Computer with internet access (for primer design/checking, protocol access)
- Freezer (-20°C)
- Refrigerator (4°C)
- Sterile 1.5 mL microcentrifuge tubes
- Sterile 0.2 mL PCR tubes or 96-well PCR plates
- PCR tube caps or plate sealers
- Fine-tip permanent markers
- Disposable gloves (nitrile recommended)
- Erlenmeyer flask (100-250 mL)
- Magnetic stirrer and stir bar (optional, for gel prep)
- Parafilm (optional)

REAGENT SETUP

1. **Primer Working Stocks:** Prepare 10 µM working stocks for all primers listed in **Tables 1 & 2**, *except* for *pfk13* primers, which require a 5 µM working stock by diluting concentrated stocks with nuclease-free water. The formula is C1V1 = C2V2. Aliquots were stored at -20°C. Labels with primer names, concentrations, dates, and initials. **TIMING:** 30 minutes.
2. **1X TBE Buffer:** Prepare 1X TBE buffer by diluting 10X TBE stock with deionized water. To 1 L of 1X TBE, 100 mL of 10X TBE with 900 mL of deionized water. The samples were stored at room temperature. **TIMING:** 10 minutes.

Table 1: Primers for Multiplex Assay I

Target Gene	Primer Name	Working Stock Conc.	Sequence (5'-3')	Expected Amplicon (bp)
pfkelch13	pfk13_f	5 µM	CTATGACGTATGATAGGGAATCTGG	2120
	pfk13_r	5 µM	CTGGGAACTAATAAAGATGGGCC	
pfmdr1	pfmdr1 f	10 µM	TGGTAACCTCAGTATCAAAG	4155
	pfmdr1 r	10 µM	CATCTTGTGCTGATAATAATTC	
pfcytb	pfcyt-b f	10 µM	CTAATTAATTTAGTTAAAGCACAC	937
	pfcyt-b r	10 µM	ACAGAATAATCTCTAGCACCA	

Table 2: Primers for Multiplex Assay II

Target Gene	Primer Name	Working Stock Conc.	Sequence (5'-3')	Expected Amplicon (bp)
pfcrt	pfcrt_f	10 µM	TTACATATAACAAAATGAAATTCGC	3109
	pfcrt_r	10 µM	TATTGTGTAATAATTGAATCGACG	
pfdhfr	pfdhfr_f	10 µM	TTTTTACTAGCCATTTTTGTATTCC	2067
	pfdhfr_r	10 µM	TTAACCGTTCAGGTAATTTTGTCA	
pfdhps	pfdhps_f	10 µM	AATATTTGCGCCAAACTTTTTA	2817
	pfdhps_r	10 µM	TTTATTTCGTAATAGTCCACTTTTGAT	
pfMt	Mt F	10 µM	AAGCTTTTGGTATCTCGTAAT	5967
	Mt R	10 µM	TATTATAATATAACTCTACAAAGTTGAAC	

PROCEDURE

1. Sample Quality Control (Optional but Recommended) ● TIMING: Variable (depends on qPCR assay, ~2-3 hours)

- **1|** Assess the quality and quantity of parasite DNA using a *P. falciparum*-specific qPCR assay (refer to specific qPCR protocol).
- **2| CRITICAL STEP:** Samples with Ct values > 34 may indicate very low parasite DNA concentrations or poor quality, potentially leading to amplification failure or bias in multiplex PCR. We excluded these samples or ran them separately with potential optimization. Therefore, gel verification was essential.

2. PCR Preparation and Setup ● TIMING: ~ 1 hour

- **3| Decontaminate Work Areas:** Thoroughly clean dedicated PCR setup areas (No-DNA/Master Mix station, DNA-only/template station), pipettes, and racks with 10% bleach followed by 70% ethanol. Expose the surfaces and non-autoclavable equipment to UV light for ≥ 30 min.
- **4| Thaw Reagents**: Thaw primer working stocks, dNTPs, PCR buffers (10X High Fidelity Buffer, 5X Phusion Buffer), KB Extender, and nuclease-free water on ice or at room temperature (allow ~10-15 min). **CRITICAL STEP:** Keep polymerase enzymes (Platinum Taq HiFi, Phusion) at -20°C until immediately before use.
- **5| Mix Reagents**: Once thawed, gently vortex and briefly centrifuge the primers, dNTPs, and buffers. **CRITICAL STEP:** Do *not* vortex polymerase enzymes or KB Extender. Mix enzymes gently by flicking or pipetting immediately before adding them to the master mix. All reagents were kept on ice or a pre-chilled PCR cooler rack (-20°C).
- **6| Prepare Consumables**: Place the required number of sterile PCR tubes/plates and PCR cooler racks in the UV workstations during decontamination. Label tubes/wells clearly for samples, No Template Controls (NTCs; use water instead of DNA), and Positive Controls (optional; use reference DNA).
- **7| Program Thermocycler(s)**: Ensure that thermocyclers are programmed with the correct cycling conditions for assays I (step 11) and II (step 15).

3. Multiplex Assay I (*pfk13*, *pfmdr1*, *pfcytb*) ● TIMING: ~ 20 min setup + ~3-4 hr PCR run

- **8| Prepare Master Mix I**: The master Mix UV workstation, prepared on a cold block, according to the table below. Calculate the volumes for the number of reactions + 10% excess.

Component	Stock Conc.	Vol per 50 µL rxn (µL)	Final Conc.
Nuclease-free water	-	26.475	-
10X High Fidelity PCR Buffer	10X	5.0	1X
50 mM $MgSO_4$	50 mM	1.5	1.5 mM
10 mM dNTP Mix	10 mM each	1.0	0.2 mM each
KB Extender	100%	1.5	3%
Fw Primer K13	5 µM	0.3125	0.03125 µM
Rv Primer K13	5 µM	0.3125	0.03125 µM
Fw Primer mdr1	10 µM	2.25	0.45 µM
Rv Primer mdr1	10 µM	2.25	0.45 µM
Fw Primer cyt-b	10 µM	2.5	0.5 µM
Rv Primer cyt-b	10 µM	2.5	0.5 µM
**Subtotal Volume**		**45.6**	
Platinum™ Taq HiFi	5 U/µL	0.4	2 U / rxn
**Total Master Mix / rxn**		**46.0**	

- **9| CRITICAL STEP:** Add Platinum™ Taq HiFi polymerase last. Mix master mix gently by pipetting (do not vortex). Briefly centrifuge.
- **10| Assemble Reactions**: In the DNA-only workstation, aliquot 46.0 µL of Master Mix I was added to each labeled PCR tube/well on a cold block. Add 4.0 µL of template DNA (target, ~1 ng/µL input concentration; ~4 ng total DNA) or nuclease-free water (NTCs) was added. **CRITICAL STEP:** Change the tips between samples. Avoiding cross-contamination.

- **11| Run PCR** I: Securely seal tubes/plates Centrifuge briefly (~10 s) to collect the contents. Place the preprogrammed thermocycler and start the run.
    - Initial Denaturation: 94°C, 2 min
    - 30 Cycles:
        - 94°C, 30 sec
        - 56°C, 30 sec
        - 65°C, 5 min
    - Final Extension: 65°C, 10 min
    - Hold: 4°C
- 12| ■ **PAUSE POINT:** PCR products can be stored at 4°C overnight or at -20°C for longer periods before gel analysis.

4. Multiplex Assay II (*pfcrt*, *pfdhfr*, *pfdhps*, *pfMt*) ● TIMING: ~ 20 min setup + ~3-4 hr PCR run

- **13| Prepare Master Mix II**: The master Mix UV workstation, prepared on a cold block, according to the table below. Calculate the volumes for the number of reactions + 10% excess.

Component	Stock Conc.	Vol per 50 µL rxn (µL)	Final Conc.
Nuclease-free water	-	27.0	-
5X Phusion HF Buffer	5X	10.0	1X
10 mM dNTP Mix	10 mM each	1.0	0.2 mM each
Fw Primer crt	10 µM	1.125	0.225 µM
Rv Primer crt	10 µM	1.125	0.225 µM
Fw Primer pfdhps	10 µM	0.875	0.175 µM
Rv Primer pfdhps	10 µM	0.875	0.175 µM
Fw Primer pfdhfr	10 µM	0.625	0.125 µM
Rv Primer pfdhfr	10 µM	0.625	0.125 µM
Fw Primer pfMt	10 µM	1.125	0.225 µM
Rv Primer pfMt	10 µM	1.125	0.225 µM
**Subtotal Volume**		**45.5**	
Phusion® Polymerase	2 U/µL	0.5	1 U / rxn
**Total Master Mix / rxn**		**46.0**	

    - ***Optional**: If using DMSO (e.g., 3% final), add 1.5 µL DMSO and reduce water volume to 25.5 µL.*
- **14| CRITICAL STEP:** Add Phusion® polymerase last. Mix gently by pipetting (do not vortex). Briefly centrifuge.
- **15| Assemble Reactions**: In the DNA-only workstation, aliquot 46.0 µL of Master Mix II was added to each labeled PCR tube/well on a cold block. Add 4.0 µL of template DNA (source suggests 50-250 ng total DNA; this is quite high and may need optimization–start lower, e.g., 5-20 ng, if samples are of good quality) or nuclease-free water (NTCs). **CRITICAL STEP:** Avoid cross-contamination.
- **16| Run PCR II**: Securely seal tubes/plates Centrifuge briefly. Place in the pre-programmed thermocycler (can pre-heat the block to 98°C) and start the run:
    - Initial Denaturation: 98°C, 3 min
    - 28 Cycles:
        - 98°C, 10 sec
        - 64°C, 20 sec
        - 65°C, 5 min
    - Final Extension: 65°C, 10 min
    - Hold: 4°C
- **17| ■ PAUSE POINT:** PCR products can be stored at 4°C overnight or at -20°C for longer periods before gel analysis.

5. Agarose Gel Electrophoresis (Verification) ● TIMING: ~ 1.5 - 2 hours

- **18| Prepare Agarose Gel:**
    - **a.** Weigh agarose for desired concentration (e.g., 1.0 g for 1% or 1.5 g for 1.5% gel) and add to 100 mL of 1X TBE buffer in an Erlenmeyer flask.

- **b.** Heat (microwave or heating plate) by swirling until fully dissolved. **CAUTION:** The solution is hot.
- **c.** Cool solution to ~50-60°C (comfortable holding flask).
- **d. CAUTION:** Working in a designated area/fume hood, add nucleic acid stain (e.g., 3-5 µL of 10 mg/mL EtBr per 100 mL gel or SYBR Safe per manufacturer's instructions). **CRITICAL STEP:** Mix gently by swirling to avoid bubbles.
- **e.** Insert the comb into the gel casting tray. The cooled agarose solution was then poured into the tray. Allow complete (~20-30 min at RT).

- **19| Load Gel**:
  - **a**. Carefully remove the comb The gel tray was then placed in the electrophoresis chamber. Add 1X TBE buffer was added to cover the gel surface (~1 cm depth).
  - **b**. Prepare samples: mix 8 µL of PCR product with 2 µL of 6X loading dye.
  - **c**. Load the prepared samples (8-10 µL) into the wells. Load the DNA ladder (5 µL) into at least one well. **CRITICAL STEP:** Use at least 25% of samples from each assay plus NTCs and any positive controls.
- **20| Run Gel**: Place lid on the chamber, connect electrodes (**black**=negative/cathode, red = positive/anode). **Run** at 100-130 V for ~45-60 min, or until the dye front nears the end of the gel. **CRITICAL STEP:** Monitor runs to prevent samples from running off.
- **21| Visualize Gel**: Turn off power and disconnect the electrodes. Carefully remove gel. **CAUTION:** Wear UV-protective eyewear using a UV transilluminator. The bands were visualized using an appropriate imaging system. Capture image.
- **22| Analyze**: Check for bands of expected sizes in sample lanes. Confirm absence of bands in NTC lanes. Assess amplification success and specificity.
- **23| ■PAUSE POINT:** Gels stained with EtBr can sometimes be stored wrapped in plastic wrap with a little buffer at 4°C for short periods before imaging, but diffusion may occur. The best practice is to immediately imagine.

6. Downstream Processing (Optional) ● TIMING: Variable

- **24|** If amplification is successful, proceed with desired downstream applications (amplicon pooling, purification, normalization using methods such as SequalPrep™ Normalization Plates, and sequencing). Follow protocols specific to the downstream method.

TIMING

- **Reagent Setup:** ~40 minutes
- **Sample QC (qPCR):** ~2-3 hours (if performed)
- **PCR Setup (Both Assays):** ~1 hour
- **Multiplex Assay I PCR Run:** ~3-4 hours
- **Multiplex Assay II PCR Run:** ~3-4 hours
- **Agarose Gel Electrophoresis:** ~1.5 - 2 hours
- **Total Time (excluding QC):** ~ 6–8 h (excluding overnight holds).

TROUBLESHOOTING

Step(s)	Problem	Possible Cause	Solution
11, 16	No bands or very weak bands	Insufficient/poor quality template DNA (esp. if Ct > 34)	Increase template amount (within limits); re-extract DNA; use positive control DNA to check reagents; optimize annealing temperature (esp. Assay II); increase cycle number slightly (e.g., 30->35 for Assay I, 28->32 for Assay II); verify primer integrity.
		PCR inhibition	Ensure template DNA is clean; dilute template DNA (e.g., 1:5, 1:10) to reduce inhibitor concentration.

		Incorrect reagent concentration/degradation	Use fresh reagents; verify primer/dNTP concentrations; ensure enzyme activity (use positive control).
		Incorrect annealing temperature (Ta)	Optimize Ta using gradient PCR, especially for Assay II (try 60°C-62°C range).
		Incorrect extension time/temp	Ensure extension time is sufficient for largest amplicon (esp. *pfmdr1*, *pfMt*). For Assay II (Phusion), consider testing 72°C extension temp with adjusted time (15-30 sec/kb).
11, 16	Non-specific bands (extra bands)	Ta too low; primers non-specific; too much template/enzyme; contamination	Increase Ta (try 2°C increments); optimize MgSO4 (Assay I) or MgCl2 (inherent in Phusion buffer); reduce template/primer/enzyme concentrations; redesign primers if necessary; ensure clean setup procedures. Consider DMSO for Assay II (if not already used).
11, 16	Bands in NTC lane	Contamination (reagents, water, tips, environment)	Use fresh aliquots of reagents (esp. water, primers); use filter tips; strictly follow separate area procedures (clean benches, dedicated pipettes); run reagent-only controls to identify source.
21	Difficulty resolving all bands on gel	Wide range of amplicon sizes (esp. Assay II); inappropriate gel concentration	Use appropriate gel % (e.g., 1% might be okay, 0.8% for better large band resolution, 1.5% for better small band resolution); run gel longer at lower voltage for better separation; consider running assays on separate gels if needed.

ANTICIPATED RESULTS

- Successful amplification, confirmed by agarose gel electrophoresis, showed bands corresponding to the expected sizes for each target within a given multiplex reaction (**Tables 1 & 2**).
- **Assay I:** Bands at ~4155 bp (*pfmdr1*), ~2120 bp (*pfk13*), and ~937 bp (*pfcytb*).
- **Assay II:** Bands at ~5967 bp (*pfMt*), ~3109 bp (*pfcrt*), ~2817 bp (*pfdhps*), and ~2067 bp (*pfdhfr*).
- Band intensities may vary between targets within a multiplex reaction and between samples.
- NTC lanes should be free of amplification products.
- Positive control lanes (if used) should show all expected bands for the respective assays.

SUPPLEMENTARY METHODS

Sample size determination

We did not perform a formal sample size calculation before the study. Instead, we chose a target sample size of approximately 50 isolates per site pragmatically, based on available resources and logistics, but is sufficient to identify the distribution of *Pfk13* mutants at moderate frequencies. This sample size is widely considered a practical benchmark in population genomics for capturing local haplotype diversity and enabling meaningful comparisons across sites.

A useful calculation is the probability of detecting at least one mutant isolate if the mutation is truly present in the population at frequency p[1]. The probability of detecting at least one mutant when the true population frequency is [p] and [n]:

Detection probability: $C = 1 - (1 - p)^n$
Required sample size: $n = \frac{log(1 - C)}{log(1 - p)}$

Where:

- C = desired confidence/probability of detecting at least one mutation (for example, 0.95 for 95% confidence).
- p = expected true mutation frequency in the population (for example, 0.05 for 5%).
- n = required number of isolates to sample.

Using this framework, approximately, 59 isolates per site are required to have 95% desired confidence of detecting at least one mutation if the true mutation frequency is 5%. These calculations show that ~50 isolates per site are well suited to detect common and moderately frequent variants (≈5–10%) at high probability but are underpowered to reliably detect very rare variants (<2%) at the individual-site level. Using the achieved/pragmatic target of n = 50 isolates per site.

True mutation frequency (p)	Formula	Detection probability (C)
5%	1 - (0.95)^50	92.3%
10%	1 - (0.90)^50	99.5%

The sample size required per-site at 95% detection probability was

True mutation frequency (p)	Required sample size for 95% detection
10%	29 isolates
5%	59 isolates
2%	149 isolates
1%	299 isolates

At the overall study level, the sample size achieved (605) provides very high probability (9.8%) of detecting variants present at 1% frequency in the sampled population.

Reference

1. Wohl, S., Lee, E.C., DiPrete, B.L. & Lessler, J. Sample size calculations for pathogen variant surveillance in the presence of biological and systematic biases. *Cell Rep Med* **4**, 101022 (2023).
