# Supplementary material for: Genomic surveillance reveals co-occurrence of Plasmodium falciparum drug resistance variants across diverse transmission settings in Ethiopia
Source: Nat Microbiol. 2026 Aug 5;11(9):2577–88. doi: 10.1038/s41564-026-02420-5 (PMC13524833; doi:10.1038/s41564-026-02420-5)
Supplement: Supplementary file 2 [file 41564_2026_2420_MOESM2_ESM.pdf]

nature portfolio

Corresponding author(s): Fitsum Girma Tadesse

Last updated by author(s): 25/02/2026

Reporting Summary

Nature Portfolio wishes to improve the reproducibility of the work that we publish. This form provides structure for consistency and transparency in reporting. For further information on Nature Portfolio policies, see our Editorial Policies and the Editorial Policy Checklist.

Statistics

For all statistical analyses, confirm that the following items are present in the figure legend, table legend, main text, or Methods section.

n/a	Confirmed	
☐	☒	The exact sample size (*n*) for each experimental group/condition, given as a discrete number and unit of measurement
☐	☒	A statement on whether measurements were taken from distinct samples or whether the same sample was measured repeatedly
☐	☒	The statistical test(s) used AND whether they are one- or two-sided<br>*Only common tests should be described solely by name; describe more complex techniques in the Methods section.*
☐	☒	A description of all covariates tested
☐	☒	A description of any assumptions or corrections, such as tests of normality and adjustment for multiple comparisons
☐	☒	A full description of the statistical parameters including central tendency (e.g. means) or other basic estimates (e.g. regression coefficient) AND variation (e.g. standard deviation) or associated estimates of uncertainty (e.g. confidence intervals)
☐	☒	For null hypothesis testing, the test statistic (e.g. *F*, *t*, *r*) with confidence intervals, effect sizes, degrees of freedom and *P* value noted<br>*Give P values as exact values whenever suitable.*
☐	☒	For Bayesian analysis, information on the choice of priors and Markov chain Monte Carlo settings
☒	☐	For hierarchical and complex designs, identification of the appropriate level for tests and full reporting of outcomes
☐	☒	Estimates of effect sizes (e.g. Cohen's *d*, Pearson's *r*), indicating how they were calculated

Our web collection on statistics for biologists contains articles on many of the points above.

Software and code

Policy information about availability of computer code

Data collection	Field data were initially collected using paper-based forms and subsequently double-entered into Redcap (mobile application version 5.20.11). Any discrepancies identified during the data entry process were resolved by cross-referencing the original paper forms and reaching a consensus among the data managers
Data analysis	Code used for the bioinformatics and statistical analyses is available at https://github.com/AHRI-Malaria/EMAGEN and https://github.com/LSHTMPathogenSeqLab/malaria-hub. Raw Illumina paired-end reads were quality-controlled using the next-generation Sequence Analysis Toolkit (https://github.com/yyr4/Nf-NeST/). Associations between haplotype frequencies, the annual parasite index (API), and the fraction of P. vivax and total infections were analyzed using Spearman's correlation. For haplotype analysis of P. falciparum drug resistance genes (Pfcrt, Pfmdr1, Pfk13, Pfdhps, Pfdhfr), full-gene amplicon sequences were aligned using MAFFT software. To visualise gene-wide haplotype diversity and geographical spread, minimal-spanning networks were constructed using all observed synonymous non-synonymous polymorphisms within coding sequence of each gene. These haplotype networks were constructed using R package pegas (version 1.3). SNP-based principal component analysis (PCA) was conducted using the scikit-allel (version 1.3.13) Python package. Identity-by-descent (IBD) were used to assess relatedness between isolates, by estimating the pairwise fraction of shared ancestry between the sequenced segments using the hmmIBD software which applies a hidden Markov model-based approach (https://github.com/LSHTMPathogenSeqLab/malaria-hub/tree/master/ibd).This was run on a binary matrix including synonymous and non-synonymous SNPs. We applied the default parameter threshold of inbreeding coefficient >0.95 to restrict analysis to monoclonal infections. Network graphs were created using the igraph R package (v.1.3.5)

For manuscripts utilizing custom algorithms or software that are central to the research but not yet described in published literature, software must be made available to editors and reviewers. We strongly encourage code deposition in a community repository (e.g. GitHub). See the Nature Portfolio guidelines for submitting code & software for further information.

Data

Policy information about availability of data

All manuscripts must include a data availability statement. This statement should provide the following information, where applicable:

- Accession codes, unique identifiers, or web links for publicly available datasets
- A description of any restrictions on data availability
- For clinical datasets or third party data, please ensure that the statement adheres to our policy

All sequencing data are available in the European Nucleotide Archive (ENA) under project accession PRJEB87415. Sample names, accession numbers, and metadata are available in Supplementary Table 6.

Publicly available P. falciparum genomic sequences used for context were obtained from previous studies, cited in the main text file. The study also references data available from PlasmoDB (https://plasmodb.org/plasmo/app) and malaria incidence data from the Malaria Atlas Project (https://data.malariaatlas.org) and total confirmed malaria cases detected and population projections from the Ethiopia office of the United Nations for the Coordination of Humanitarian Affairs (https://data.humdata.org/organization/ocha-ethiopia). All de-identified datasets generated during the current study and used to make all figures are stated to be available as supplementary files or tables.
Public reference data: PlasmoDB (https://plasmodb.org/plasmo/app), Malaria Atlas Project (https://data.malariaatlas.org), and previous studies. All metadata are available through these links. No restrictions on the availability of the data generated in this study are described. The study utilized de-identified samples from symptomatic patients..

Research involving human participants, their data, or biological material

Policy information about studies with human participants or human data. See also policy information about sex, gender (identity/presentation), and sexual orientation and race, ethnicity and racism.

Reporting on sex and gender	Gender and Sex were self reported
Reporting on race, ethnicity, or other socially relevant groupings	The study did not use race, ethnicity, or any other socially relevant groupings in the current analysis.
Population characteristics	Demography of the study participants is provided in Table 1.
Recruitment	Volunteer patients who presented at the study clinics with malaria suspected signs and symptoms were recruited in this study until the set sample number was obtained with no exclusion criteria except age below 6 months.
Ethics oversight	Approved by Armauer Hansen Research Institute and ALERT Hospital Ethics Review Committee (PO/46/20) and University of Notre Dame IRB (20-12-6350)

Note that full information on the approval of the study protocol must also be provided in the manuscript.

Field-specific reporting

Please select the one below that is the best fit for your research. If you are not sure, read the appropriate sections before making your selection.

☒ Life sciences ☐ Behavioural & social sciences ☐ Ecological, evolutionary & environmental sciences

For a reference copy of the document with all sections, see nature.com/documents/nr-reporting-summary-flat.pdf

Life sciences study design

All studies must disclose on these points even when the disclosure is negative.

Sample size	No formal a priori power calculation was performed. We set a pragmatic target of ~50 isolates per site (605 isolates total across 15 districts) based on field logistics, sequencing capacity and available resources, and to ensure broad geographic and transmission setting representation. A per site target of ~50 is a commonly used benchmark in population genomic surveillance to capture local haplotype diversity and enable between site comparisons, while retaining reasonable sensitivity to detect moderately frequent variants: for example, with [n=50] the probability of detecting at least one mutant present at 5% frequency is [~92.3%] and for a 10% variant [~99.5%]; across the full dataset [n=605] the probability of detecting a variant present at 1% overall is [~99.8%]. Thus, our design is well suited to describe the distribution of common and moderately frequent resistance markers (including Pfk13 variants) and to support multilevel association analyses, while acknowledging limited sensitivity for reliably detecting very rare variants at the individual site level. (See Wohl et al. for approaches to genomic surveillance sample size planning.)<br><br>A sample size determination sections is provided as Supplementary Methods.

Wohl, S., Lee, E.C., DiPrete, B.L. & Lessler, J. Sample size calculations for pathogen variant surveillance in the presence of biological and systematic biases. Cell Rep Med 4, 101022 (2023).

Data exclusions: Samples excluded due to low-depth coverage or potential polyclonal infections (VAF 5-95%) for haplotype analysis.

Replication: Targeted amplicon sequencing was performed once per sample; reproducibility was ensured through systematic quality controls at each stage rather than technical replication. Positive (Pf3D7 reference strain) and negative (no-template) controls were included across DNA extraction, P. falciparum confirmation, parasite quantification, amplification, and sequencing. Target amplification was validated using laboratory reference isolates with defined phenotypic backgrounds alongside field isolates. Amplicon generation was verified by gel electrophoresis prior to sequencing, with no-template and negative controls included to monitor contamination. Sequencing quality was enforced through read-quality filters, minimum coverage thresholds, ensemble SNP calling, and predefined haplotype-calling criteria; variant calls were further validated against control samples with known genotypes. All validation checks supported the reported findings.

Randomization: This was not relevant to the study as it was a descriptive genomic prevalence surveillance.

Blinding: Not applicable. This is an observational molecular surveillance study with no experimental intervention or treatment allocation. Our study compared sequence data to reference genome using standardized pipelines.

Reporting for specific materials, systems and methods

We require information from authors about some types of materials, experimental systems and methods used in many studies. Here, indicate whether each material, system or method listed is relevant to your study. If you are not sure if a list item applies to your research, read the appropriate section before selecting a response.

Materials & experimental systems

n/a	Involved in the study
☒	☐ Antibodies
☒	☐ Eukaryotic cell lines
☒	☐ Palaeontology and archaeology
☒	☐ Animals and other organisms
☒	☐ Clinical data
☒	☐ Dual use research of concern
☒	☐ Plants

Methods

n/a	Involved in the study
☒	☐ ChIP-seq
☒	☐ Flow cytometry
☒	☐ MRI-based neuroimaging

Plants

Seed stocks: NA

Novel plant genotypes: NA

Authentication: NA
