# Supplementary material for: Genomic surveillance reveals co-occurrence of Plasmodium falciparum drug resistance variants across diverse transmission settings in Ethiopia
Source: Nat Microbiol. 2026 Aug 5;11(9):2577–88. doi: 10.1038/s41564-026-02420-5 (PMC13524833; doi:10.1038/s41564-026-02420-5)
Supplement: Supplementary file 3 [file 41564_2026_2420_MOESM3_ESM.pdf]

nature portfolio

Peer Review File

Genomic surveillance reveals co-occurrence of Plasmodium falciparum drug resistance variants across diverse transmission settings in Ethiopia

Corresponding Author: Dr Fitsum Tadesse

Version 0:

Reviewer comments:

Reviewer #1

(Remarks to the Author)
This is an interesting article documenting the diversity of genetic changes associated with drug resistance in a panel of samples derived from 15 regions across Ethiopia. The work demonstrates the value of molecular surveillance in defining the presence and prevalence of genetic markers associated with drug resistance. Interesting observations include the high frequency of mutations associated with resistance to chloroquine or SP in regions where these drugs are no longer recommended for treatment of P. falciparum malaria. It would have been helpful if the authors had explored potential confounding factors including the availability and use of these drugs in the private sector, potential role for other dhfr inhibitors used for bacterial infections to impact the malaria parasite and issues of parasite fitness.
These authors have previously reported the emergence of mutations in the Pfkelch13 gene associated with delayed clearance of parasites in Ethiopia. This work adds some incremental information on the distribution of those mutations in the 15 sites but provides limited new information. One of the most interesting previous observations is the high frequency of the R622I mutation in a specific subset of geographic locations. A much more in-depth analysis of a larger number of samples from these sites, including historic samples, if available, could have provided key insights. However, this work only includes a few samples from these regions.
The authors imply with the title and the abstract that there is “Convergent Evolution of Artemisinin and Chloroquine Resistance in Ethiopian Plasmodium falciparum Parasites”, however the data presented in the manuscript fails to rigorously demonstrate convergent evolution. These resistances mechanism emerged overly vastly different time scales – chloroquine was in common use from the middle of the twentieth century and independent resistance emerged in two geographic locations – southeast Asia and South America. Previous work by many other groups have demonstrated that the chloroquine resistant alleles found in African parasites had their origin in Southeast Asia – they did not independently occur in parasites of African origin. In contrast, the Pfkelch13 mutations associated with delayed parasite clearance have emerged independently in Africa in several distinct locations. The population history and evolution of these two resistance mechanisms are distinct. While both mutations lead to an altered response to drugs, the drugs have different mechanisms of action and resistance mechanisms.
The manuscript is primarily descriptive without functional validation of resistance using either in vivo or in vitro tests. This is particularly relevant for the Pfkelch13 locus – the authors include a well-documented polymorphism at position 189 – this is present at high allele frequencies (30-50% in some studies) of all P. falciparum parasites and has be shown to lack association or causation of delayed clinical response to artemisinin or is negative in the in vitro RSA assay. Including this locus seems unwise and it could skew some of the analyses. Similarly, several other polymorphisms in Pfkelch13 have been detected but validated. These should be handled separately from the known resistance markers. For the other loci examined, only know resistance mediating polymorphisms have been included in the analysis.
The data presentation could be significantly improved. Even using a high resolution 48 inch computer monitor, the data presentation in Figures 3 and 4 is difficult to analyze. Since some of the major conclusions the authors would like to make are based on these Figures, I suggest a major redo of fonts and colors and the transfer of some of the less critical data to supplemental figures. Figure 3D and 3E address the issue of co-occurrence of mutations in one of the drug resistance genes and it is difficult to independently analyze the data to determine if the conclusions presented by the authors are valid. Because of the high frequency of mutations in PfCRT and PfDHFR/DHPS in the parasite population, how valid are these association studies? As mentioned above, it would have been interesting to do this analysis at much greater depth in sites where the Pfkelch13 mutations are at the highest frequency.
It would be useful to know the distribution of samples that contain more than one parasite genome. This is particularly important in areas of higher transmission – in those samples that contain more than one genome, it is not possible to conclude that a single parasite contains multiple mutations and this could impact the conclusions drawn from the data in Figures 3 and 4. Since the data includes sites with a range of transmission intensity, the data cannot be combined in a single analysis.
The manuscript would be greatly strengthened by the inclusion of either clinical response data or in vitro drug resistance studies. It would also be strengthened by an in-depth analysis of a few of the sites where Pfkelch13 mutations have high frequency,

(Remarks on code availability)

Reviewer #2

(Remarks to the Author)
This paper presents a detailed study of key drug resistance genes following genomic surveillance, their prevalence and geographic location within Ethiopia. They demonstrate evidence of multi-drug resistance including persisting mutations to discontinued drugs, identify epidemiologically distinct P. falciparum isolates, and further document the spread of ART-R markers in Africa.
These results contribute to the understanding of emerging drug resistance in particular ART-R in Africa which presents a real threat to the effectiveness of many frontline antimalarials. The findings can also guide anti-malarial policy decision making in Ethiopia.
Overall the data is of high quality, generally well presented and with convincing conclusions. The referencing is also well done and appropriate. There seem to be a few minor inconsistencies between the data as discussed in the main text and shown in the supplementary and extended tables. Lots of Track Changes are still unresolved in the manuscript file.

Specific remarks:
• At the end of the introduction, lines 80-82, it would be helpful to already specify between which years the samples were collected and analyzed (2020-2022?).
• The text in Figures 1B and C is hard to read on the darker colors. Consider putting a white outline on the letters or adjusting the background colors.
• Line 99, median age is stated as 20.2 years across the participants, but from Table 1 this appears to be the median of medians not the true median.
• Figure 2 appears to be duplicated. Track changes need to be resolved.
• Line 103, this subtitle could read better as “The persistence of pfdhfr and pfdhps mutations associated with antifolate resistance”.
• Lines 117 – 121, discussion around Extended Data 4A, there seem to be discrepancies. What criteria was used for a mutation to be considered prominent in a region? The coloured regional sections on the pie chart visually are not in a clear majority for the regions being called prominent. For example:
o Line 118: “the triple mutant ARINI (Cluster II, 43.1%) predominating in Batu, Fedis, and Mizan”. Why not also Fentale?
o Line 119: “AICNI (Cluster I, 38.7%) in Gambella and Gondar”. Why Gambella but not K. Humera and Metema?
o Line 119: “the wild-type (Cluster III, 16.1%) in Guba”. Why not also Pawe?
o Line 120-121: “For PfDHPS, the K540E-containing Cluster I was frequent in Amibara, Batu, and Fedis”. Should this instead be Fentale ahead of Amibara?
o Line 121: “G437A-dominant Clusters II and V were more common in Guba (Extended Data 4B)”. Why not also Pawe and Salamango?
In the caption of Extended Data 4 different conclusions are drawn:
o Caption states “PfDHFR clusters II (257) and I (231) were predominantly found in Fentale”, yet in the main text Line 118 and 119 make no mention of Fentale.
o Caption states “PfDHPS clusters I (285) and II (136) were shared between Fedis and Fentale” yet the main text Line 120-121 make no mention of Fentale and mention other locations as being predominant. It is also not clear from the graphs that these are the predominant regions. For example, cluster II for PfDHPS (Panel B) does not show Fentale or Fedis (orange colors) at all.

• Line 145 – 147, the data referring to the Y184F codon is not found in Extended Table 3. Perhaps it should be Supplementary Table 1. But within Supplementary Table 1 the Y184F values range from 75.8% in Amibara to 100% in others, why was the 75.8% in Amibara disregarded in the main text which states that the range is from 97% in Fentale: “the Y184F codon, appearing in 93.5% (503/538) of samples and detected in all districts, with prevalence ranging from 97.0% in Fentale (32/33) to 100% in several districts (Batu, Dera, Fedis, Guba, K. Humera, Mizan)”
• Line 167, 17% should be 1.7%
• Line 200, SNP abbreviation has not been defined in the main text.
• Line 675, Table 1 heading, Sequenced samples (n)*, what does the ‘*’ refer to?
• Line 702, is a duplicate of Figure 2
• Line 717, Figure 3B, D and E are too small to see the data. Also Figure 3 has 2 captions, the second is more detailed and more informative than the first. Within the caption Line 752 SNP abbreviation has not been defined, and the SNPs were referred to as “resistance markers” in part A of the figure so it becomes confusing.
• Supplementary Table 1. How is the Overall N calculated? For example, in the row for pfmdr1 N1042, the sum of N for each of the regions is 584, while the N for the overall column is 531, how is this possible? This then led to a query in Line 148-149 where the codon N1042 is described as entirely wildtype which is correct for the Overall column, but when considered by region some areas such as Gondar are as low as 68%.

(Remarks on code availability)
The code is generally comprehensive, suitably annotated and should be a usable resource for the community. The code does provide a README file, although the README could contain a more detailed description of the various codes and more information on how to use/install/run the codes. There could also be more information on which prerequisite files and software are needed to allow the full running of the code. The current README appears to be more of an index.

Reviewer #3

(Remarks to the Author)
Overall, I appreciate the data presented and its importance in understanding selection of artemisinin partial resistance and ACT partner drug resistance in Ethiopia, particularly given the opportunity it provides to gleam information on how the use of multiple first line therapies (in this case the use of AL to treat Pf and CQ to treat Pv in the same locations) may impact on the selection of ACT resistance. However, I believe that the presentation of the data should be greatly simplified to better communicate key take away messages. In addition, the authors need to use more nuance language in describing the impact of certain mutations on drug susceptibilities and in explaining interpretations of data. Also, the deep exploration of the data using different analytical methods is admirable, but presenting more analysis without providing more information on the motivation and data interpretation can actually be more confusing. I spent a lot of time trying to understand what additional information complementary analyses added, especially when methods were not clearly described.

Specifically, I found the focus on haplotypes to be unnecessarily complicated, especially as
found some of the analyses to be a bit redundant. It was unclear to me what some of the analyses added to the story. For example,
1. Can the authors clarify the origin of panels 1b and 1c? No reference is given indicating they are generated from previously published data, nor is there any information in the methods section to indicate how the data were collected/generated/analyzed. I recognize that more information is included in the legend of the figure, but would prefer there is at least some mention of this in the methods section as well.
2. It is indicated that the mitochondrial genome was sequenced, but these data are never mentioned in the text. Extended data 1 suggests relatively poor coverage and a lack of variation. This should be mentioned in the results to explain there is no further analysis of the gene.
3. I would suggest mentioning the “tagment” step in your methods section for the targeted sequencing.
4. Section starting on line 104: I would suggest simplifying the haplotype. For example, since 16 and 164 are all WT, including these in the haplotype is not particular helpful, and remembering which amino acid is WT and which is mutant is a bit of a burden. In some of the tables/figures the mutant amino acids are bolded and underlined. Consider doing this in the text, or defining and using terms such as “dhfr triple mutant” or “dhps double mutant”.
5. In addition, the haplotypes are not clearly defined in the results section, so a reader who has not memorized the dhfr/dhps mutants would have difficulty knowing which locus each amino acid in the haplotype belongs to. It should be clearly explained that AIRNI means PfDHFR A16, 51I, 59R, 108N and I164 and so on.
6. I suggest adding gene labels to figure 2. It is unclear why the dhfr and crt data is presented in one color scheme and the dhps and mdr1 data in another. I think it would be more clear to use the same color scheme across panels. Also, the inconsistency in the legend of whether the panel label comes before or after the description of the panel is confusing
7. Section starting on line 104: I recommend adding a sentence trying to summarize the geographical distribution (or lack there of) of the antifolate mutations (regional level, rather than site level). I feel that this will strengthen your point in the section starting at 156 when the association between SP and CQ markers and Pv prevalence is made.
8. I found it confusing that the minimal-spanning networks show novel missense mutations that are not reported in Supplementary Table 2. Also, I think it would be helpful to explicitly state that this analysis included all polymorphisms in the gene where as the haplotypes included drug resistance loci of interest, and what additional information you intend to share (what does this add to your initial discussion of the haplotypes defined by drug resistance markers of interest).
9. Line 118: I believe there is a type. ARINI should be AIRNI
10. Section starting at 140. I find the characterization of NFSND as “mutant” and a known secondary modulator of chloroquine resistance a bit misleading. Its actually the N86Y mutation that is most strongly associated with lumefantrine/chloroquine sensitivity in east Africa, while the role of Y184F is a bit less clear. In fact a recent meta-analysis suggests that it is not associated with susceptibility to the drug at all (https://pubmed.ncbi.nlm.nih.gov/40506728/). I think the more accurate message is that following the switch to AL, mdr1 86Y mutations have become less prevalent (a quick look at published papers showing earlier prevalences make me believe this is true), consistent with lumefantrine selection. Any discussion of 184 needs to be much more nuanced and recognize the limitations of this marker.
11. While I appreciate the efforts the authors took to present data in a visual, I do not think Extended Data 5 was helpful. I recommend sticking to Supplementary Table 3a/b. Also, it is not standard to have separate “panels” for a table, so rename them Supplementary Table 3 and 4 or combine them into a single table. Finally, why are the K13 genotypes not included in the analysis?
12. Section starting on line 184. Is this “linkage” driven by interaction/fitness/adaptation or is it incidental because certain mutations are more prevalent in certain geographical regions?
13. Line 193: I would be hesitant to use the term “genomic population structure” here, as it is really just based off of polymorphisms in drug resistance genes, isn’t it? Maybe molecularly defined resistance profiles would be more accurate?
14. Line 112: consider changing from “district-specific patterns” to “site-specific patterns”, since there is only 1 site per district.
15. It is hard to interpret some of the data shown in figure 3 because the small size/resolution makes it hard to read text. Simplifying may help, as key panels can be made larger. Consider dropping panel a (data can be or is described in text or supplemental figures)
16. In general, I dislike the term “mutant” being used for haplotypes where there are multiple different “mutant” combinations that confer different levels of resistance. I think it would be more helpful to make your point using individual SNPs or more precise terms like double or triple mutant in the case of dhfr and dhps.
17. Section beginning at line 184. What statistical test was used here?
18. For the section starting on line 202: which SNPs were used to make this neighbour joining tree? I would be hesitant to claim gene flow and connectedness if only using drug resistance genes, as relationships are likely driven more by drug selection pressure than identity by decent. You’d need more neutral markers, and there is no mention of additional genotyping/sequencing of the samples analyzed in this study. The same comment applies to the section starting with 209. This is more appropriately done with flanking haplotypes, but it is not clear this was done.
19. Discussion section starting at 250. I’m not so convinced that CVIET creates a “permissive genetic background” as much as there is geographical overlap in selective pressure.

20. Again, I’m not convinced of distinct parasite clusters based purely on drug resistance gene sequences. More precise language is needed if you are referring to “molecularly defined resistance patterns” as opposed to identity by descent.
21.

(Remarks on code availability)

Decision Letter:

11th July 2025

*Please ensure you delete the link to your author homepage in this e-mail if you wish to forward it to your co-authors.

Dear Fitsum,

Thank you for your patience while your manuscript "Convergent Evolution of Artemisinin and Chloroquine Resistance in Ethiopian Plasmodium falciparum Parasites" was under peer-review at Nature Microbiology. It has now been seen by 3 referees, whose expertise and comments you will find at the end of this email. Although they find your work of some potential interest, they have raised a number of concerns that will need to be addressed before we can consider publication of the work in Nature Microbiology.

In particular, you will see that the referees ask for some further validation of the findings, to refine the analysis, to make the text more accessible, and to better describe the novelty of the work. Editorially, we will need you to address all referee comments.

Should further experimental data and text changes allow you to address these criticisms, we would be happy to look at a revised manuscript.

We are committed to providing a fair and constructive peer-review process. Please do not hesitate to contact us if there are specific requests from the reviewers that you believe are technically impossible or unlikely to yield a meaningful outcome.

We strongly support public availability of data. Please place the data used in your paper into a public data repository, if one exists, or alternatively, present the data as Source Data or Supplementary Information. If data can only be shared on request, please explain why in your Data Availability Statement, and also in the correspondence with your editor. For some data types, deposition in a public repository is mandatory - more information on our data deposition policies and available repositories can be found at https://www.nature.com/nature-research/editorial-policies/reporting-standards#availability-of-data.

Please include a data availability statement as a separate section after Methods but before references, under the heading "Data Availability”. This section should inform readers about the availability of the data used to support the conclusions of your study. This information includes accession codes to public repositories (data banks for protein, DNA or RNA sequences, microarray, proteomics data etc…), references to source data published alongside the paper, unique identifiers such as URLs to data repository entries, or data set DOIs, and any other statement about data availability. At a minimum, you should include the following statement: “The data that support the findings of this study are available from the corresponding author upon request”, mentioning any restrictions on availability. If DOIs are provided, we also strongly encourage including these in the Reference list (authors, title, publisher (repository name), identifier, year). For more guidance on how to write this section please see:
http://www.nature.com/authors/policies/data/data-availability-statements-data-citations.pdf

When revising your manuscript:

* Include a “Response to referees” document detailing, point-by-point, how you addressed each referee comment. If no action was taken to address a point, you must provide a compelling argument. This response will be sent back to the referees along with the revised manuscript.

* If you have not done so already we suggest that you begin to revise your manuscript so that it conforms to our Article format instructions at http://www.nature.com/nmicrobiol/info/final-submission. Refer also to any guidelines provided in this letter.

* Include a revised version of any required reporting checklist. It will be available to referees (and, potentially, statisticians) to aid in their evaluation if the manuscript goes back for peer review. A revised checklist is essential for re-review of the paper.

When submitting the revised version of your manuscript, please pay close attention to our href="https://www.nature.com/nature-portfolio/editorial-policies/image-integrity">Digital Image Integrity Guidelines. and to the following points below:

-- that unprocessed scans are clearly labelled and match the gels and western blots presented in figures.
-- that control panels for gels and western blots are appropriately described as loading on sample processing controls
-- all images in the paper are checked for duplication of panels and for splicing of gel lanes.

EXTENDED DATA FIGURES

When re-submitting your manuscript, please ensure that any supplementary figures and tables that are crucial to the

manuscript's conclusions are converted into Extended Data figures and tables to increase visibility of these data. Extended Data figures and tables are online-only (present in the online PDF and full-text HTML versions of the paper), peer-reviewed display items that provide essential background to the article but are not included in the main article due to space constraints. A maximum of ten Extended Data display items (figures and tables) is permitted.

Finally, please ensure that you retain unprocessed data and metadata files after publication, ideally archiving data in perpetuity, as these may be requested during the peer review and production process or after publication if any issues arise.

Please use the link below to submit a revised paper:

Link Redacted

Note: This url links to your confidential homepage and associated information about manuscripts you may have submitted or be reviewing for us. If you wish to forward this e-mail to co-authors, please delete this link to your homepage first.

Nature Microbiology is committed to improving transparency in authorship. As part of our efforts in this direction, we are now requesting that all authors identified as 'corresponding author' on published papers create and link their Open Researcher and Contributor Identifier (ORCID) with their account on the Manuscript Tracking System (MTS), prior to acceptance. This applies to primary research papers only. ORCID helps the scientific community achieve unambiguous attribution of all scholarly contributions. You can create and link your ORCID from the home page of the MTS by clicking on 'Modify my Springer Nature account'. For more information please visit please visit www.springernature.com/orcid.

If you wish to submit a suitably revised manuscript we would hope to receive it within 6 months. If you cannot send it within this time, please let us know.

In the meantime we hope that you find our referees' comments helpful.

Yours sincerely,

****
Reviewer Expertise:

Referee #1: P. falciparum resistance
Referee #2: P. falciparum resistance, genomics, drug discovery
Referee #3: P. falciparum genomics, evolution of antimalarial drug resistance

Reviewer Comments:

Reviewer #1 (Remarks to the Author):

This is an interesting article documenting the diversity of genetic changes associated with drug resistance in a panel of samples derived from 15 regions across Ethiopia. The work demonstrates the value of molecular surveillance in defining the presence and prevalence of genetic markers associated with drug resistance. Interesting observations include the high frequency of mutations associated with resistance to chloroquine or SP in regions where these drugs are no longer recommended for treatment of P. falciparum malaria. It would have been helpful if the authors had explored potential confounding factors including the availability and use of these drugs in the private sector, potential role for other dhfr inhibitors used for bacterial infections to impact the malaria parasite and issues of parasite fitness.
These authors have previously reported the emergence of mutations in the Pfkelch13 gene associated with delayed clearance of parasites in Ethiopia. This work adds some incremental information on the distribution of those mutations in the 15 sites but provides limited new information. One of the most interesting previous observations is the high frequency of the R622I mutation in a specific subset of geographic locations. A much more in-depth analysis of a larger number of samples from these sites, including historic samples, if available, could have provided key insights. However, this work only includes a few samples from these regions.
The authors imply with the title and the abstract that there is "Convergent Evolution of Artemisinin and Chloroquine Resistance in Ethiopian Plasmodium falciparum Parasites", however the data presented in the manuscript fails to rigorously demonstrate convergent evolution. These resistances mechanism emerged overly vastly different time scales – chloroquine was in common use from the middle of the twentieth century and independent resistance emerged in two geographic locations – southeast Asia and South America. Previous work by many other groups have demonstrated that the chloroquine resistant alleles found in African parasites had their origin in Southeast Asia – they did not independently occur in parasites of African origin. In contrast, the Pfkelch13 mutations associated with delayed parasite clearance have emerged independently in Africa in several distinct locations. The population history and evolution of these two resistance mechanisms are distinct. While both mutations lead to an altered response to drugs, the drugs have different mechanisms of action and resistance mechanisms.
The manuscript is primarily descriptive without functional validation of resistance using either in vivo or in vitro tests. This is particularly relevant for the Pfkelch13 locus – the authors include a well-documented polymorphism at position 189 – this is present at high allele frequencies (30-50% in some studies) of all P. falciparum parasites and has be shown to lack association or causation of delayed clinical response to artemisinin or is negative in the in vitro RSA assay. Including this locus seems unwise and it could skew some of the analyses. Similarly, several other polymorphisms in Pfkelch13 have been detected but validated. These should be handled separately from the known resistance markers. For the other loci examined, only know resistance mediating polymorphisms have been included in the analysis.

The data presentation could be significantly improved. Even using a high resolution 48 inch computer monitor, the data presentation in Figures 3 and 4 is difficult to analyze. Since some of the major conclusions the authors would like to make are based on these Figures, I suggest a major redo of fonts and colors and the transfer of some of the less critical data to supplemental figures. Figure 3D and 3E address the issue of co-occurrence of mutations in one of the drug resistance genes and it is difficult to independently analyze the data to determine if the conclusions presented by the authors are valid. Because of the high frequency of mutations in PfCRT and PfDHFR/DHPS in the parasite population, how valid are these association studies? As mentioned above, it would have been interesting to do this analysis at much greater depth in sites where the Pfkelch13 mutations are at the highest frequency.

It would be useful to know the distribution of samples that contain more than one parasite genome. This is particularly important in areas of higher transmission – in those samples that contain more than one genome, it is not possible to conclude that a single parasite contains multiple mutations and this could impact the conclusions drawn from the data in Figures 3 and 4. Since the data includes sites with a range of transmission intensity, the data cannot be combined in a single analysis.

The manuscript would be greatly strengthened by the inclusion of either clinical response data or in vitro drug resistance studies. It would also be strengthened by an in-depth analysis of a few of the sites where Pfkelch13 mutations have high frequency,

Reviewer #2 (Remarks to the Author):

This paper presents a detailed study of key drug resistance genes following genomic surveillance, their prevalence and geographic location within Ethiopia. They demonstrate evidence of multi-drug resistance including persisting mutations to discontinued drugs, identify epidemiologically distinct P. falciparum isolates, and further document the spread of ART-R markers in Africa.

These results contribute to the understanding of emerging drug resistance in particular ART-R in Africa which presents a real threat to the effectiveness of many frontline antimalarials. The findings can also guide anti-malarial policy decision making in Ethiopia.

Overall the data is of high quality, generally well presented and with convincing conclusions. The referencing is also well done and appropriate. There seem to be a few minor inconsistencies between the data as discussed in the main text and shown in the supplementary and extended tables. Lots of Track Changes are still unresolved in the manuscript file.

Specific remarks:

• At the end of the introduction, lines 80-82, it would be helpful to already specify between which years the samples were collected and analyzed (2020-2022?).
• The text in Figures 1B and C is hard to read on the darker colors. Consider putting a white outline on the letters or adjusting the background colors.
• Line 99, median age is stated as 20.2 years across the participants, but from Table 1 this appears to be the median of medians not the true median.
• Figure 2 appears to be duplicated. Track changes need to be resolved.
• Line 103, this subtitle could read better as “The persistence of pfdhfr and pfdhps mutations associated with antifolate resistance”.
• Lines 117 – 121, discussion around Extended Data 4A, there seem to be discrepancies. What criteria was used for a mutation to be considered prominent in a region? The coloured regional sections on the pie chart visually are not in a clear majority for the regions being called prominent. For example:
o Line 118: “the triple mutant ARINI (Cluster II, 43.1%) predominating in Batu, Fedis, and Mizan”. Why not also Fentale?
o Line 119: “AICNI (Cluster I, 38.7%) in Gambella and Gondar”. Why Gambella but not K. Humera and Metema?
o Line 119: “the wild-type (Cluster III, 16.1%) in Guba”. Why not also Pawe?
o Line 120-121: “For PfDHPS, the K540E-containing Cluster I was frequent in Amibara, Batu, and Fedis”. Should this instead be Fentale ahead of Amibara?
o Line 121: “G437A-dominant Clusters II and V were more common in Guba (Extended Data 4B)”. Why not also Pawe and Salamango?
In the caption of Extended Data 4 different conclusions are drawn:
o Caption states “PfDHFR clusters II (257) and I (231) were predominantly found in Fentale”, yet in the main text Line 118 and 119 make no mention of Fentale.
o Caption states “PfDHPS clusters I (285) and II (136) were shared between Fedis and Fentale” yet the main text Line 120-121 make no mention of Fentale and mention other locations as being predominant. It is also not clear from the graphs that these are the predominant regions. For example, cluster II for PfDHPS (Panel B) does not show Fentale or Fedis (orange colors) at all.

• Line 145 – 147, the data referring to the Y184F codon is not found in Extended Table 3. Perhaps it should be Supplementary Table 1. But within Supplementary Table 1 the Y184F values range from 75.8% in Amibara to 100% in others, why was the 75.8% in Amibara disregarded in the main text which states that the range is from 97% in Fentale: “the Y184F codon, appearing in 93.5% (503/538) of samples and detected in all districts, with prevalence ranging from 97.0% in Fentale (32/33) to 100% in several districts (Batu, Dera, Fedis, Guba, K. Humera, Mizan)”
• Line 167, 17% should be 1.7%
• Line 200, SNP abbreviation has not been defined in the main text.
• Line 675, Table 1 heading, Sequenced samples (n)*, what does the ‘*’ refer to?
• Line 702, is a duplicate of Figure 2
• Line 717, Figure 3B, D and E are too small to see the data. Also Figure 3 has 2 captions, the second is more detailed and more informative than the first. Within the caption Line 752 SNP abbreviation has not been defined, and the SNPs were referred to as “resistance markers” in part A of the figure so it becomes confusing.
• Supplementary Table 1. How is the Overall N calculated? For example, in the row for pfmdr1 N1042, the sum of N for each of

the regions is 584, while the N for the overall column is 531, how is this possible? This then led to a query in Line 148-149 where the codon N1042 is described as entirely wildtype which is correct for the Overall column, but when considered by region some areas such as Gondar are as low as 68%.

Reviewer #2 (Remarks on code availability):

The code is generally comprehensive, suitably annotated and should be a usable resource for the community. The code does provide a README file, although the README could contain a more detailed description of the various codes and more information on how to use/install/run the codes. There could also be more information on which prerequisite files and software are needed to allow the full running of the code. The current README appears to be more of an index.

Reviewer #3 (Remarks to the Author):

Overall, I appreciate the data presented and its importance in understanding selection of artemisinin partial resistance and ACT partner drug resistance in Ethiopia, particularly given the opportunity it provides to gleam information on how the use of multiple first line therapies (in this case the use of AL to treat Pf and CQ to treat Pv in the same locations) may impact on the selection of ACT resistance. However, I believe that the presentation of the data should be greatly simplified to better communicate key take away messages. In addition, the authors need to use more nuance language in describing the impact of certain mutations on drug susceptibilities and in explaining interpretations of data. Also, the deep exploration of the data using different analytical methods is admirable, but presenting more analysis without providing more information on the motivation and data interpretation can actually be more confusing. I spent a lot of time trying to understand what additional information complementary analyses added, especially when methods were not clearly described.

Specifically, I found the focus on haplotypes to be unnecessarily complicated, especially as
found some of the analyses to be a bit redundant. It was unclear to me what some of the analyses added to the story. For example,
1. Can the authors clarify the origin of panels 1b and 1c? No reference is given indicating they are generated from previously published data, nor is there any information in the methods section to indicate how the data were collected/generated/analyzed. I recognize that more information is included in the legend of the figure, but would prefer there is at least some mention of this in the methods section as well.
2. It is indicated that the mitochondrial genome was sequenced, but these data are never mentioned in the text. Extended data 1 suggests relatively poor coverage and a lack of variation. This should be mentioned in the results to explain there is no further analysis of the gene.
3. I would suggest mentioning the “tagment” step in your methods section for the targeted sequencing.
4. Section starting on line 104: I would suggest simplifying the haplotype. For example, since 16 and 164 are all WT, including these in the haplotype is not particular helpful, and remembering which amino acid is WT and which is mutant is a bit of a burden. In some of the tables/figures the mutant amino acids are bolded and underlined. Consider doing this in the text, or defining and using terms such as “dhfr triple mutant” or “dhps double mutant”.
5. In addition, the haplotypes are not clearly defined in the results section, so a reader who has not memorized the dhfr/dhps mutants would have difficulty knowing which locus each amino acid in the haplotype belongs to. It should be clearly explained that AIRNI means PfDHFR A16, 51I, 59R, 108N and I164 and so on.
6. I suggest adding gene labels to figure 2. It is unclear why the dhfr and crt data is presented in one color scheme and the dhps and mdr1 data in another. I think it would be more clear to use the same color scheme across panels. Also, the inconsistency in the legend of whether the panel label comes before or after the description of the panel is confusing
7. Section starting on line 104: I recommend adding a sentence trying to summarize the geographical distribution (or lack there of) of the antifolate mutations (regional level, rather than site level). I feel that this will strengthen your point in the section starting at 156 when the association between SP and CQ markers and Pv prevalence is made.
8. I found it confusing that the minimal-spanning networks show novel missense mutations that are not reported in Supplementary Table 2. Also, I think it would be helpful to explicitly state that this analysis included all polymorphisms in the gene where as the haplotypes included drug resistance loci of interest, and what additional information you intend to share (what does this add to your initial discussion of the haplotypes defined by drug resistance markers of interest).
9. Line 118: I believe there is a type. ARINI should be AIRNI
10. Section starting at 140. I find the characterization of NFSND as “mutant” and a known secondary modulator of chloroquine resistance a bit misleading. Its actually the N86Y mutation that is most strongly associated with lumefantrine/chloroquine sensitivity in east Africa, while the role of Y184F is a bit less clear. In fact a recent meta-analysis suggests that it is not associated with susceptibility to the drug at all (https://pubmed.ncbi.nlm.nih.gov/40506728/). I think the more accurate message is that following the switch to AL, mdr1 86Y mutations have become less prevalent (a quick look at published papers showing earlier prevalences make me believe this is true), consistent with lumefantrine selection. Any discussion of 184 needs to be much more nuanced and recognize the limitations of this marker.
11. While I appreciate the efforts the authors took to present data in a visual, I do not think Extended Data 5 was helpful. I recommend sticking to Supplementary Table 3a/b. Also, it is not standard to have separate “panels” for a table, so rename them Supplementary Table 3 and 4 or combine them into a single table. Finally, why are the K13 genotypes not included in the analysis?
12. Section starting on line 184. Is this “linkage” driven by interaction/fitness/adaptation or is it incidental because certain mutations are more prevalent in certain geographical regions?
13. Line 193: I would be hesitant to use the term “genomic population structure” here, as it is really just based off of polymorphisms in drug resistance genes, isn’t it? Maybe molecularly defined resistance profiles would be more accurate?

14. Line 112: consider changing from “district-specific patterns” to “site-specific patterns”, since there is only 1 site per district.
15. It is hard to interpret some of the data shown in figure 3 because the small size/resolution makes it hard to read text. Simplifying may help, as key panels can be made larger. Consider dropping panel a (data can be or is described in text or supplemental figures)
16. In general, I dislike the term “mutant” being used for haplotypes where there are multiple different “mutant” combinations that confer different levels of resistance. I think it would be more helpful to make your point using individual SNPs or more precise terms like double or triple mutant in the case of dhfr and dhps.
17. Section beginning at line 184. What statistical test was used here?
18. For the section starting on line 202: which SNPs were used to make this neighbour joining tree? I would be hesitant to claim gene flow and connectedness if only using drug resistance genes, as relationships are likely driven more by drug selection pressure than identity by decent. You’d need more neutral markers, and there is no mention of additional genotyping/sequencing of the samples analyzed in this study. The same comment applies to the section starting with 209. This is more appropriately done with flanking haplotypes, but it is not clear this was done.
19. Discussion section starting at 250. I’m not so convinced that CVIET creates a “permissive genetic background” as much as there is geographical overlap in selective pressure.
20. Again, I’m not convinced of distinct parasite clusters based purely on drug resistance gene sequences. More precise language is needed if you are referring to “molecularly defined resistance patterns” as opposed to identity by descent.
21.

****

Version 1:

Reviewer comments:

Reviewer #1

(Remarks to the Author)
The author's response to comments was extensive- almost as long as the manuscript. The changes in the manuscript were more modest but did included new and revised analysis of the data which clarified some points raised in the review. Most of the changes were modifications that put into perspective some of the previous over-reaching conclusions.
The major point which remains unresolved is the question of the relationship of existing chloroquine resistance with the emergence of K13 mutations associated with emerging resistance to artemisinin. This was a major point raised in the original critique. The authors decided to subset the data based on geographic distribution and found that this association only existed in one region. They then subset the data on transmission intensity and separately by PfCRT allele frequency. The analysis showed that population structure impacted the conclusion of an association with K13 emergence and other drug resistant genotypes. Instead of recognizing this point, the authors conclude that this association only occurs in certain unique settings. They fail to acknowledge the interrelatedness of API, geography and PfCRT distribution and fail to control for these confounders in their analysis. Therefore, their conclusion is not valid. Should the authors want to directly address this issue they need a more robust approach including modeling. As it stands, the data do not support the conclusion that there is a propensity for K13 mutations to occur in parasites with existing drug resistance mutations. They continue to make this a major conclusion of the paper and have retained this concept in the title.

(Remarks on code availability)

Reviewer #2

(Remarks to the Author)
Thank you for clearly addressing my comments in so much detail. All previous issues have been corrected and I would support the publication of this study.

(Remarks on code availability)
The code files are improved and appear to provide adequate information.

Reviewer #3

(Remarks to the Author)
I really appreciate how much effort the authors put into addressing my concerns. I feel the paper is much clearer and more directly addresses what I see as one of its most interesting points: what happens in terms of drug resistance when AL and CQ are both used in the same setting. This mirrors one the proposed policy changes that stakeholders hope will prolong ACT efficacy -- using multiple first line therapies -- and this provides useful data to help model expectations.
I think this is an interesting, informative paper, but I still have a few concerns/suggestions.
I think both the current title and the proposed revised title are confusing and unclear
1. lines 137-144. I continue to be concerned that exploration of gene-wide diversity and evolutionary relationships may still be confounded by selection rather than genetic relatedness. Is there additional variation in synonymous SNPs? Could the analyses

be repeated with these more neutral markers? In fact, all minimal-spanning haplotype networks would benefit from use of microhaplotypes including all SNPs, not just NS SNPs.
2. I think it would be worth mentioning the lack of significant correlations between prevalence of validated K13 mutations and API and Pv proportion in the main text (Sup table 4). This provides information on how multiple first line therapy may influence the success of ART-R. It doesn't seem to help here, but still may be protective of the partner drug. Sorry for my obsession with this topic.
3. For the population structure shown in the PCA analysis: I had voiced concerns that the population structure may not be reflective of IBD because it was driven by drug resistance genes, which are more likely to reflect selective pressure than genetic relatedness. The authors addressed this concern by indicating that mitochondrial sequences were also used. However, it is also reported that variation in the mitochondrial genome was limited. What does the structure look like when just considering the mitochondrial genome? Is there sufficient variation to define populations? Also, were synonymous SNPs used in this analysis or just NS SNPs as was done for minimal.-spanning networks. I couldn't quite tell from the methods section. If not, I think it would be a good idea to include non-coding polymorphisms to reduce the impact of convergent evolution resulting from drug selection, providing a more accurate inference of relatedness.
4. Line 315. The emergence of different K13 mutations in different regions is not limited to SEA. The same is true of Uganda. May be worth mentioning to give additional African context.
5. Section starting at 320. Does there have to epistatic interaction? Could this just be explained by the fact that parasites need to survive exposure to DHA, lumefantrine and chloroquine, so its good to have resistance to all these drugs when possible? The fitness costs of having resistance to multiple drugs is likely to vary by likelihood of exposure to AL and CQ, by competition with other parasites or evading immune responses in higher transmission areas.
6. Line 345: The N86 allele is associated with decreased susceptibility to lumefantrine an increased susceptibility to chloroquine, not reduced susceptibility to both drugs.
7. Line 362. I think it would be wise to acknowledge the limitations of the IBD analysis as it is done primarily with variation under selective pressure. Including all SNPs
8. Line 368. I think it would be more appropriate to say "and there is limited evidence for lumefantrine failure in Africa" rather than no reported failures. A recent TES showed <90% AL efficacy at 2 sites in Uganda (Kamya 2025 Lancet ID) and there have been reports of AL treatment failures when travelers returned to their home countries after being in Africa.

CRT I356T is associated with piperaquine resistance in SEA, where dihydroartemisinin-piperaquine was widely used.

(Remarks on code availability)

Decision Letter:

5th December 2025

*Please ensure you delete the link to your author homepage in this e-mail if you wish to forward it to your co-authors.

Dear Fitsum,

Thank you for your patience while your manuscript "Distinct Genetic Histories, Convergent Drug Resistance in Ethiopian Plasmodium falciparum: Artemisinin and Chloroquine" was under peer-review at Nature Microbiology. It has now been seen by 3 referees, whose expertise and comments you will find at the end of this email. Although they find your work of some potential interest, they have raised a number of concerns that will need to be addressed before we can consider publication of the work in Nature Microbiology.

In particular, while referee #2 is now satisfied, referees #1 and #3 still have some important concerns. Referee #1 says that, as it stands, the data do not support the conclusion that there is a propensity for K13 mutations to occur in parasites with existing drug resistance mutations. The referee suggests to remove the concept of convergent evolution and provide a neutral reporting of the data. Editorially, we would encourage you to do this as otherwise, we will need you to perform more robust methodology, including modeling, for the subsetting analysis. Referee #3 says the title needs to be modified to better reflect the findings of this study and says they are concerned that exploration of gene-wide diversity and evolutionary relationships may still be confounded by selection rather than genetic relatedness. Editorially, we will need you to address the concerns from both referees in the manuscript and ensure that analyses are appropriate and reporting is in line with the data. I should note that this will be the last chance to revise the manuscript accordingly.

Should further experimental data and/or text changes allow you to address these criticisms, we would be happy to look at a revised manuscript.

We are committed to providing a fair and constructive peer-review process. Please do not hesitate to contact us if there are specific requests from the reviewers that you believe are technically impossible or unlikely to yield a meaningful outcome.

We strongly support public availability of data. Please place the data used in your paper into a public data repository, if one exists, or alternatively, present the data as Source Data or Supplementary Information. If data can only be shared on request, please explain why in your Data Availability Statement, and also in the correspondence with your editor. For some data types, deposition in a public repository is mandatory - more information on our data deposition policies and available repositories can be found at https://www.nature.com/nature-research/editorial-policies/reporting-standards#availability-of-data.

Please include a data availability statement as a separate section after Methods but before references, under the heading "Data Availability". This section should inform readers about the availability of the data used to support the conclusions of your study.

This information includes accession codes to public repositories (data banks for protein, DNA or RNA sequences, microarray, proteomics data etc…), references to source data published alongside the paper, unique identifiers such as URLs to data repository entries, or data set DOIs, and any other statement about data availability. At a minimum, you should include the following statement: “The data that support the findings of this study are available from the corresponding author upon request”, mentioning any restrictions on availability. If DOIs are provided, we also strongly encourage including these in the Reference list (authors, title, publisher (repository name), identifier, year). For more guidance on how to write this section please see: http://www.nature.com/authors/policies/data/data-availability-statements-data-citations.pdf

When revising your manuscript:

* Include a “Response to referees” document detailing, point-by-point, how you addressed each referee comment. If no action was taken to address a point, you must provide a compelling argument. This response will be sent back to the referees along with the revised manuscript.

* If you have not done so already we suggest that you begin to revise your manuscript so that it conforms to our Article format instructions at http://www.nature.com/nmicrobiol/info/final-submission. Refer also to any guidelines provided in this letter.

* Include a revised version of any required reporting checklist. It will be available to referees (and, potentially, statisticians) to aid in their evaluation if the manuscript goes back for peer review. A revised checklist is essential for re-review of the paper.

When submitting the revised version of your manuscript, please pay close attention to our href="https://www.nature.com/nature-portfolio/editorial-policies/image-integrity">Digital Image Integrity Guidelines. and to the following points below:

-- that unprocessed scans are clearly labelled and match the gels and western blots presented in figures.
-- that control panels for gels and western blots are appropriately described as loading on sample processing controls
-- all images in the paper are checked for duplication of panels and for splicing of gel lanes.

EXTENDED DATA FIGURES

When re-submitting your manuscript, please ensure that any supplementary figures and tables that are crucial to the manuscript’s conclusions are converted into Extended Data figures and tables to increase visibility of these data. Extended Data figures and tables are online-only (present in the online PDF and full-text HTML versions of the paper), peer-reviewed display items that provide essential background to the article but are not included in the main article due to space constraints. A maximum of ten Extended Data display items (figures and tables) is permitted.

Finally, please ensure that you retain unprocessed data and metadata files after publication, ideally archiving data in perpetuity, as these may be requested during the peer review and production process or after publication if any issues arise.

Please use the link below to submit a revised paper:

Link Redacted

Note: This url links to your confidential homepage and associated information about manuscripts you may have submitted or be reviewing for us. If you wish to forward this e-mail to co-authors, please delete this link to your homepage first.

Nature Microbiology is committed to improving transparency in authorship. As part of our efforts in this direction, we are now requesting that all authors identified as ‘corresponding author’ on published papers create and link their Open Researcher and Contributor Identifier (ORCID) with their account on the Manuscript Tracking System (MTS), prior to acceptance. This applies to primary research papers only. ORCID helps the scientific community achieve unambiguous attribution of all scholarly contributions. You can create and link your ORCID from the home page of the MTS by clicking on ‘Modify my Springer Nature account’. For more information please visit please visit www.springernature.com/orcid.

If you wish to submit a suitably revised manuscript we would hope to receive it within 2 months. If you cannot send it within this time, please let us know.

In the meantime we hope that you find our referees' comments helpful.

Yours sincerely,

****
Reviewer Expertise:

Referee #1: P. falciparum resistance
Referee #2: P. falciparum resistance, genomics, drug discovery
Referee #3: P. falciparum genomics, evolution of antimalarial drug resistance

Reviewer Comments:

Reviewer #1 (Remarks to the Author):

The author's response to comments was extensive- almost as long as the manuscript. The changes in the manuscript were more modest but did included new and revised analysis of the data which clarified some points raised in the review. Most of the changes were modifications that put into perspective some of the previous over-reaching conclusions.
The major point which remains unresolved is the question of the relationship of existing chloroquine resistance with the emergence of K13 mutations associated with emerging resistance to artemisinin. This was a major point raised in the original critique. The authors decided to subset the data based on geographic distribution and found that this association only existed in one region. They then subset the data on transmission intensity and separately by PfCRT allele frequency. The analysis showed that population structure impacted the conclusion of an association with K13 emergence and other drug resistant genotypes. Instead of recognizing this point, the authors conclude that this association only occurs in certain unique settings. They fail to acknowledge the interrelatedness of API, geography and PfCRT distribution and fail to control for these confounders in their analysis. Therefore, their conclusion is not valid. Should the authors want to directly address this issue they need a more robust approach including modeling. As it stands, the data do not support the conclusion that there is a propensity for K13 mutations to occur in parasites with existing drug resistance mutations. They continue to make this a major conclusion of the paper and have retained this concept in the title.

Reviewer #2 (Remarks to the Author):

Thank you for clearly addressing my comments in so much detail. All previous issues have been corrected and I would support the publication of this study.

Reviewer #2 (Remarks on code availability):

The code files are improved and appear to provide adequate information.

Reviewer #3 (Remarks to the Author):

I really appreciate how much effort the authors put into addressing my concerns. I feel the paper is much clearer and more directly addresses what I see as one of its most interesting points: what happens in terms of drug resistance when AL and CQ are both used in the same setting. This mirrors one the proposed policy changes that stakeholders hope will prolong ACT efficacy -- using multiple first line therapies -- and this provides useful data to help model expectations.
I think this is an interesting, informative paper, but I still have a few concerns/suggestions.
I think both the current title and the proposed revised title are confusing and unclear
1. lines 137-144. I continue to be concerned that exploration of gene-wide diversity and evolutionary relationships may still be confounded by selection rather than genetic relatedness. Is there additional variation in synonymous SNPs? Could the analyses be repeated with these more neutral markers? In fact, all minimal-spanning haplotype networks would benefit from use of microhaplotypes including all SNPs, not just NS SNPs.
2. I think it would be worth mentioning the lack of significant correlations between prevalence of validated K13 mutations and API and Pv proportion in the main text (Sup table 4). This provides information on how multiple first line therapy may influence the success of ART-R. It doesn't seem to help here, but still may be protective of the partner drug. Sorry for my obsession with this topic.
3. For the population structure shown in the PCA analysis: I had voiced concerns that the population structure may not be reflective of IBD because it was driven by drug resistance genes, which are more likely to reflect selective pressure than genetic relatedness. The authors addressed this concern by indicating that mitochondrial sequences were also used. However, it is also reported that variation in the mitochondrial genome was limited. What does the structure look like when just considering the mitochondrial genome? Is there sufficient variation to define populations? Also, were synonymous SNPs used in this analysis or just NS SNPs as was done for minimal.-spanning networks. I couldn't quite tell from the methods section. If not, I think it would be a good idea to include non-coding polymorphisms to reduce the impact of convergent evolution resulting from drug selection, providing a more accurate inference of relatedness.
4. Line 315. The emergence of different K13 mutations in different regions is not limited to SEA. The same is true of Uganda. May be worth mentioning to give additional African context.
5. Section starting at 320. Does there have to epistatic interaction? Could this just be explained by the fact that parasites need to survive exposure to DHA, lumefantrine and chloroquine, so its good to have resistance to all these drugs when possible? The fitness costs of having resistance to multiple drugs is likely to vary by likelihood of exposure to AL and CQ, by competition with other parasites or evading immune responses in higher transmission areas.
6. Line 345: The N86 allele is associated with decreased susceptibility to lumefantrine an increased susceptibility to chloroquine, not reduced susceptibility to both drugs.
7. Line 362. I think it would be wise to acknowledge the limitations of the IBD analysis as it is done primarily with variation under selective pressure. Including all SNPs
8. Line 368. I think it would be more appropriate to say "and there is limited evidence for lumefantrine failure in Africa" rather than no reported failures. A recent TES showed <90% AL efficacy at 2 sites in Uganda (Kamya 2025 Lancet ID) and there have been reports of AL treatment failures when travelers returned to their home countries after being in Africa.

CRT I356T is associated with piperaquine resistance in SEA, where dihydroartemisinin-piperaquine was widely used.

****

Version 2:

Reviewer comments:

Reviewer #1

(Remarks to the Author)
This third revision of the manuscript has addressed many of the concerns raised with regard to the methodology and over-reaching conclusions. The authors now emphasize the diversity across Ethiopia with regard to drug resistance associated mutations and this is an interesting point and one worthy of reporting. The change in the title more clearly reflects the data presented in the manuscript.

(Remarks on code availability)

Decision Letter:

Our ref: NMICROBIOL-25041264B

15th May 2026

Dear Fitsum,

Thank you for submitting your revised manuscript "Genomic surveillance of Ethiopian Plasmodium falciparum reveals co-occurrence of Pfcrt and Pfk13 mutations in vivax–falciparum co-endemic settings" (NMICROBIOL-25041264B). It has now been seen by the original referees and their comments are below. The reviewers find that the paper has improved in revision, and therefore we'll be happy in principle to publish it in Nature Microbiology, pending minor revisions to comply with our editorial and formatting guidelines.

We are now performing detailed checks on your paper and will send you a checklist detailing our editorial and formatting requirements in about a week. Please do not upload the final materials and make any revisions until you receive this additional information from us.

Thank you again for your interest in Nature Microbiology. Please do not hesitate to contact me if you have any questions.

Sincerely,

Reviewer #1 (Remarks to the Author):

This third revision of the manuscript has addressed many of the concerns raised with regard to the methodology and over-reaching conclusions. The authors now emphasize the diversity across Ethiopia with regard to drug resistance associated mutations and this is an interesting point and one worthy of reporting. The change in the title more clearly reflects the data presented in the manuscript.

Version 3:

Decision Letter:

15th June 2026

Dear Fitsum,

I am pleased to accept your Article "Genomic surveillance reveals co-occurrence of Plasmodium falciparum drug resistance variants across diverse transmission settings in Ethiopia" for publication in Nature Microbiology. Thank you for having chosen to submit your work to us and many congratulations.

Over the next few weeks, your paper will be copyedited to ensure that it conforms to Nature Microbiology style. We look particularly carefully at the titles of all papers to ensure that they are relatively brief and understandable.

Once your paper is typeset, you will receive an email with a link to choose the appropriate publishing options for your paper and

our Author Services team will be in touch regarding any additional information that may be required. Once your paper has been scheduled for online publication, the Nature press office will be in touch to confirm the details.

You may wish to make your media relations office aware of your accepted publication, in case they consider it appropriate to organize some internal or external publicity. Once your paper has been scheduled you will receive an email confirming the publication details. This is normally 3-4 working days in advance of publication. If you need additional notice of the date and time of publication, please let the production team know when you receive the proof of your article to ensure there is sufficient time to coordinate. Further information on our embargo policies can be found here:
https://www.nature.com/authors/policies/embargo.html

After the grant of rights is completed, you will receive a link to your electronic proof via email with a request to make any corrections within 48 hours. If, when you receive your proof, you cannot meet this deadline, please inform us at rjsproduction@springernature.com immediately. You will not receive your proofs until the publishing agreement has been received through our system

Due to the importance of these deadlines, we ask you please us know now whether you will be difficult to contact over the next month. If this is the case, we ask you provide us with the contact information (email, phone and fax) of someone who will be able to check the proofs on your behalf, and who will be available to address any last-minute problems.

Acceptance of your manuscript is conditional on all authors' agreement with our publication policies (see https://www.nature.com/nmicrobiol/editorial-policies). In particular your manuscript must not be published elsewhere.

Authors may need to take specific actions to achieve compliance with funder and institutional open access mandates. If your research is supported by a funder that requires immediate open access (e.g. according to Plan S principles or the NIH public access policy) then you should select the gold OA route, and we will direct you to the compliant route where possible. Because authors warrant under our subscription licensing terms that they haven't committed to licensing any version of their article under a licence inconsistent with the terms of our agreement – including the applicable embargo period – publication under the subscription model isn't suitable for authors whose funders require no embargo.

If you have any questions about our publishing options, costs, Open Access requirements, or our legal forms, please contact ASJournals@springernature.com

An online order form for reprints of your paper is available at https://www.nature.com/reprints/author-reprints.html. All co-authors, authors' institutions and authors' funding agencies can order reprints using the form appropriate to their geographical region.

We welcome the submission of potential cover material (including a short caption of around 40 words) related to your manuscript; suggestions should be sent to Nature Microbiology as electronic files (the image should be 300 dpi at 210 x 297 mm in either TIFF or JPEG format). Please note that such pictures should be selected more for their aesthetic appeal than for their scientific content, and that colour images work better than black and white or grayscale images. Please do not try to design a cover with the Nature Microbiology logo etc., and please do not submit composites of images related to your work. I am sure you will understand that we cannot make any promise as to whether any of your suggestions might be selected for the cover of the journal.

You can now use a single sign-on for all your accounts, view the status of all your manuscript submissions and reviews, access usage statistics for your published articles and download a record of your refereeing activity for the Nature journals.

To assist our authors in disseminating their research to the broader community, our SharedIt initiative provides you with a unique shareable link that will allow anyone (with or without a subscription) to read the published article. Recipients of the link with a subscription will also be able to download and print the PDF.

As soon as your article is published, you will receive an automated email with your shareable link.

Congratulations once again and I look forward to seeing the article published.

With kind regards,

P.S. Click on the following link if you would like to recommend Nature Microbiology to your librarian
http://www.nature.com/subscriptions/recommend.html#forms

** Visit the Springer Nature Editorial and Publishing website at www.springernature.com/editorial-and-publishing-jobs for more information about our career opportunities. If you have any questions please click here.**

Open Access This Peer Review File is licensed under a Creative Commons Attribution 4.0 International License, which permits use, sharing, adaptation, distribution and reproduction in any medium or format, as long as you give appropriate credit to the original author(s) and the source, provide a link to the Creative Commons license, and indicate if changes were made.
In cases where reviewers are anonymous, credit should be given to 'Anonymous Referee' and the source.
The images or other third party material in this Peer Review File are included in the article’s Creative Commons license, unless indicated otherwise in a credit line to the material. If material is not included in the article’s Creative Commons license and your intended use is not permitted by statutory regulation or exceeds the permitted use, you will need to obtain permission directly from the copyright holder.
To view a copy of this license, visit https://creativecommons.org/licenses/by/4.0/

20 October 2025

Re: Manuscript NMICROBIOL-25041264.

Title: "Distinct Genetic Histories, Convergent Drug Resistance in Ethiopian Plasmodium falciparum: Artemisinin and Chloroquine"

On behalf of the co-authors, I would like to extend our sincere gratitude to you and the reviewers for the detailed and constructive feedback on our manuscript. We deeply appreciate the time and expertise invested in strengthening the clarity, rigor, and overall quality of our work.

A point-by-point response to all comments is provided in the accompanying document. For clarity, responses are highlighted in blue, with line numbers referencing the corresponding revisions in the clean version of the manuscript.

We hope the revisions satisfactorily address all concerns, and we remain happy to provide any further clarifications if needed. We look forward to your consideration as the editorial process moves forward.

In this submission, we have included the following documents:

1. Revised manuscript with track changes
2. Revised clean manuscript
3. Extended data
4. Supplementary tables
5. Supplementary Information

Thank you once again for your thoughtful guidance and support in improving our study.

Sincerely,

Fitsum Girma Tadesse (on behalf of all co-authors)

Malaria and NTD Division, Armauer Hansen Research Institute
Jimma Road, ALERT-AHRI Health Village, POBOx 1005
Addis Ababa, Ethiopia
Email: fitsum.girma@ahri.gov.et / fitsum.tadesse@lshtm.ac.uk

Reviewer #1 (Remarks to the Author):

This is an interesting article documenting the diversity of genetic changes associated with drug resistance in a panel of samples derived from 15 regions across Ethiopia. The work demonstrates the value of molecular surveillance in defining the presence and prevalence of genetic markers associated with drug resistance. Interesting observations include the high frequency of mutations associated with resistance to chloroquine or SP in regions where these drugs are no longer recommended for treatment of P. falciparum malaria. It would have been helpful if the authors had explored potential confounding factors including the availability and use of these drugs in the private sector, potential role for other dhfr inhibitors used for bacterial infections to impact the malaria parasite and issues of parasite fitness.

Response 1.1: We thank the reviewer for these insightful suggestions. While CQ and SP are no longer recommended for *P. falciparum* in Ethiopia, several factors may explain the persistence of resistance markers. We have addressed the co-endemicity with *P. vivax* and continued usage of CQ to treat *P. vivax* in these settings playing an important driving role starting from the introduction to the discussion sections of the manuscript. We also partially addressed the potential role antibiotics could play in sustaining the resistance genotypes of *dhfr* and *dhps* in the previous version. In response to the reviewer's comment, we have now expanded on the potential role of Trimethoprim–sulfamethoxazole (TMP-SMX) usage, citing studies done in Ethiopia. TMP-SMX prophylaxis is widely used in HIV treatment programs in Ethiopia. Although effective at preventing malaria, TMP-SMX targets DHFR/DHPS pathways shared with SP, raising the possibility of maintaining SP-resistant haplotypes even without SP use in malaria therapy (Staedke et al., 2001; Tessema et al., 2015; Huang et al., 2014). Data from Ethiopian HIV cohorts show TMP-SMX coverage rates up to 80% with marked impact on malaria, with a significant difference in TMP-SMX use observed among malaria positive (46 %) and malaria negative (82 %) HIV positive patients. Reduced malaria infection was observed among patients who were HIV positive, re-affirming the importance of TMP-SMX in preventing malaria symptoms and parasitaemia among HIV-positive patients (Alemayehu et al., 2015).

A section has been added to the discussion to elaborate the usage of TMP-SMX in Ethiopia, which could impact the SP resistance prevalence (lines 273-279): *"...This persistence may stem from selective pressure from Trimethoprim–Sulfamethoxazole (TMP-SMX) used to treat and prevent bacterial infections, which targets the same metabolic pathway as SP. In Ethiopia, TMP-SMX is widely used as prophylaxis within HIV care, in accordance with national and WHO guidelines (WHO, 2014). With an estimated 610,000 people living with HIV in 2023 (UNAIDS, 2025), TMP-SMX coverage in Ethiopian cohorts reaches up to 80%, significantly impacting malaria outcomes. HIV-positive individuals receiving TMP-SMX prophylaxis show markedly lower malaria infection prevalence (Alemayehu et al., 2015)."*

In response to the reviewer's additional comments, we also further expanded the discussion to explicitly address availability of CQ and SP at the private-sector and parasite fitness costs related with these changes. As the reviewer suggested, studies indicate that CQ and SP remain accessible via the private healthcare sector and informal drug outlets, even after policy withdrawal, creating ongoing drug pressure especially through use of CQ for *P. vivax* under the prevailing widescale misdiagnoses or empirical fever treatment (Argaw et al., 2016; Gindola et al., 2022). We previously quantified that the private sector in Ethiopia plays a role to diagnose and treat 16% of malaria cases (Emiru et al 2023 Nat Med). Furthermore, certain resistance mutations may impose minimal biological cost, enabling them to persist even after official withdrawal of CQ/SP. Ethiopian studies demonstrate that SP-resistant haplotypes remain stable in the population (Tessema et al., 2015).

We have now expanded the manuscript discussion to address these confounding factors explicitly and added references to support each point (see lines 279-285): *"Alternative explanations for the persistence of SP resistance markers include minimal fitness costs associated with these mutations (Gregson et a., 2025; Iyer et al., 2001) and the potential for SP-resistant parasites to exhibit greater mosquito infectivity, enhancing their transmission and maintenance (Segovia et al, 2025; Mendez et al., 2002; Barnes et al., 2008). Additionally, the continued availability of SP and CQ in the private sector may sustain residual drug pressure. In Ethiopia, up to 40% of private clinic health workers have not received training on national malaria guidelines (Gindola et al., 2022), and recent data quantified that the private sector accounts for approximately 16% of malaria diagnoses and treatments (Emiru et al., 2023). "*

These authors have previously reported the emergence of mutations in the Pfkelch13 gene associated with delayed clearance of parasites in Ethiopia. This work adds some incremental information on the distribution of those mutations in the 15 sites but provides limited new information. One of the most interesting previous observations is the high frequency of the R622I mutation in a specific subset of geographic locations. A much more in-depth analysis of a larger number of samples from these sites, including historic samples, if available, could have provided key insights. However, this work only includes a few samples from these regions.

Response 1.2: We thank the reviewer for this thoughtful comment. Our study aimed to conduct nationwide molecular surveillance to map the geographic spread of antimalarial resistance markers, rather than focus solely on known hotspots. Our site selection intentionally balanced breadth and depth by including 15 sites across Ethiopia, 7 of which (totalling n = 262 samples out of the 604 samples collected, representing 43.4% of the total samples) were from the Northwest region, where the *Pfkelch13* R622I mutation was previously identified. In five of those sites, R622I frequency remains high (16–48%), confirming its geographic persistence. This strategy ensures we capture the full spectrum of Ethiopia's heterogeneity in malaria transmission, while still prioritizing regions historically associated with resistance, as the reviewer suggested. The country's malaria landscape is highly heterogeneous, both spatially and temporally, for example, unstable transmission zones in the northwest show large spatiotemporal clusters over time (Alemu et al., 2013).

Notably, Ethiopia has experienced ten-fold increase in malaria cases between 2019 (~0.9M) and 2024 (>7.3M), the highest annual burden in recent years (WHO World Malaria Report 2023; FMoH Ethiopia, 2024). Whether this surge stems from evolving drug resistance, vector shifts, health system disruptions, or other drivers remains unclear. The previous study has examined samples collected in 2019, 2022, and 2023 (https://pubmed.ncbi.nlm.nih.gov/40681807/), whilst our study analysed samples mostly collected in 2021, the missing year, that is at the start of the resurgence and fills the temporal gap (Table 1). Taken together, the added values of these two studies include (i) cross-sectional data from these two studies provide an essential baseline against which future spatiotemporal analyses can identify evolving patterns and drivers of resistance; (ii) the national framework in our study complements previous regional analyses, offering broader insights into where future longitudinal or mechanistic studies should be prioritized; (iii) the rising malaria burden and increasing heterogeneity underscore the importance of tracking resistance markers in local context, not just in known hotspots; and (iv) temporal follow-up studies will be critical for understanding how drug resistance, ecological shifts, and human mobility interact to shape malaria dynamics in Ethiopia and across the Horn of Africa. We have included a section in the discussion to emphasize the added values of spatiotemporal data in supporting local evidence generation (lines 406-408): *"By including data from 2021, an inflection point in Ethiopia's malaria resurgence, our study helps fill the temporal gap and may provide useful context for understanding how resistance patterns evolved over the past few years."*

The authors imply with the title and the abstract that there is "Convergent Evolution of Artemisinin and Chloroquine Resistance in Ethiopian Plasmodium falciparum Parasites", however the data presented in the manuscript fails to rigorously demonstrate convergent evolution. These resistances mechanism emerged overly vastly different time scales – chloroquine was in common use from the middle of the twentieth century and independent resistance emerged in two geographic locations – southeast Asia and South America. Previous work by many other groups have demonstrated that the chloroquine resistant alleles found in African parasites had their origin in Southeast Asia – they did not independently occur in parasites of African origin. In contrast, the Pfkelch13 mutations associated with delayed parasite clearance have emerged independently in Africa in several distinct locations. The population history and evolution of these two resistance mechanisms are distinct. While both mutations lead to an altered response to drugs, the drugs have different mechanisms of action and resistance mechanisms.
Response 1.3: We thank the reviewer for this insightful comment and for highlighting the distinction between the evolutionary histories of chloroquine (CQ) and artemisinin (ART) resistance. We agree that chloroquine resistance in African *P. falciparum* primarily originated from Southeast Asia, while *Pf*Kelch13 mutations associated with ART resistance have emerged independently in multiple African locations. Our use of the term "convergent evolution" was intended to describe phenotypic convergence, i.e., the emergence of parasite populations exhibiting reduced susceptibility to both drugs, rather than implying dependent genetic origins for both resistance mechanisms in Africa. We acknowledge that our work is purely genomics and the original title and abstract could be interpreted as suggesting true convergent genetic evolution, which may be misleading. To address this, we propose revising the title to better reflect the distinction between genetic origin and functional convergence. We provided alternative title as suggested, *"Distinct Genetic Histories, Convergent Drug Resistance in Ethiopian Plasmodium falciparum: Artemisinin and Chloroquine",* if the editor approves this change. We believe this option accurately conveys the main finding, that Ethiopian parasite populations show similar drug resistance phenotypes, although not measured directly in this study, despite distinct evolutionary histories, while avoiding overstatement of convergent genetic evolution.

The manuscript is primarily descriptive without functional validation of resistance using either in vivo or in vitro tests. This is particularly relevant for the Pfkelch13 locus – the authors include a well-documented polymorphism at position 189 – this is present at high allele frequencies (30-50% in some studies) of all P. falciparum parasites and has be shown to lack association or causation of delayed clinical response to artemisinin or is negative in the in vitro RSA assay. Including this locus seems unwise and it could skew some of the analyses. Similarly, several other polymorphisms in Pfkelch13 have been detected but validated. These should be handled separately from the known resistance markers. For the other loci examined, only know resistance mediating polymorphisms have been included in the analysis.
Response 1.4: We thank the reviewer for this comment. We agree that some of the variants we detected in *Pfkelch13* are not associated with artemisinin partial resistance; therefore, we have clearly labelled this in Figure 3A, distinguishing between 'WHO validated ART-R marker', 'WHO candidate ART-R marker', 'variants not associated with ART-R', and 'variants unlikely associated with ART-R'. The latter is indicated for variants that have not been tested in *in vitro* or *in vivo* assays, but are located outside of the *Pfkelch13* propeller domain and therefore unlikely to mediate ART-R. While Figure 2 includes only known drug resistance markers for the other genes, new variants in *Pfkelch13* are emerging in Africa; therefore, we choose to report all variants detected in this gene considering the dynamism.

A sentence has been added to manuscript to clarify this (lines 204-207): "*While some variants have previously shown a lack of association with ART-R, such as K189N/T [44] and L258M [45], others have*

not been tested in vitro, but are unlikely to be associated with ART-R given their location outside of the Pfk13 beta-propeller domain (amino acids 440-726)."

Acknowledging the limitations of our study and the added values of future studies that combine phenotyping and genotyping evidence, we also addressed this comment of the reviewer in the discussion (lines 396-397): *"Our findings are based on established genotype–phenotype associations, but future research integrating clinical, in vitro, and genomic data will be crucial to validate and expand these insights."*

The data presentation could be significantly improved. Even using a high resolution 48 inch computer monitor, the data presentation in Figures 3 and 4 is difficult to analyze. Since some of the major conclusions the authors would like to make are based on these Figures, I suggest a major redo of fonts and colors and the transfer of some of the less critical data to supplemental figures.

Response 1.5: Our figures have now been revised, simplified where possible, and exported in high resolution. Waffle charts have been removed and the *Pfkelch13* haplotype network has been moved to the supplementary data. In Figure 4, IBD network graphs for IBD >=23.75% have been removed, since they did not provide much additional information (all samples were connected).

Figure 3D and 3E address the issue of co-occurrence of mutations in one of the drug resistance genes and it is difficult to independently analyze the data to determine if the conclusions presented by the authors are valid. Because of the high frequency of mutations in PfCRT and PfDHFR/DHPS in the parasite population, how valid are these association studies? As mentioned above, it would have been interesting to do this analysis at much greater depth in sites where the Pfkelch13 mutations are at the highest frequency.

Response 1.6: We agree with the reviewer that the high background frequency of *Pf*CRT and *Pf*DHFR/DHPS variants needs to be taken into account in the interpretation of co-occurrence analyses. To address this gap and assess the robustness of the findings we reported, we performed three complementary sensitivity analyses:

First, given the reviewer's concern that high background frequencies of *Pf*CRT and *Pf*DHFR/*Pf*DHPS variants could drive spurious co-occurrence, we re-ran the analysis after stratifying the isolates into three geographical clusters, of which cluster 1 is restricted to sites in northwestern Ethiopia, where *Pf*K13 variants (including R622I) were most frequent (the seven sites where R622I is concentrated; n ≈ 260 samples). This approach minimizes confounding by regional population structure and background allele frequency. Results confirm that several of the national co-occurrence signals including the co-occurrence of *Pf*K13 R622I with *Pf*CRT variants and *Pf*MDR1 Y184F persist in this subset. In the other geographical clusters (cluster 2 representing regions in the south/southwest (Dasenech, Mizan, Salamago and Gambella zuria) and cluster 3 in the east/south (Amibara, Batu/Zeway, Fedis, and Fentale)), no significant associations between *Pf*K13 variants and *Pf*CRT and *Pf*MDR1 variants were found, mostly because of the near-absence of *Pf*K13 variants in these regions (**Extended Data 8, Supplementary Table 6**). These findings suggest that part of the co-occurrence signal is due to population structure as the reviewer pointed out.

Second, to further address the reviewer's concern that associations maybe driven by the high background prevalence or near-fixation of *Pf*CRT alleles, we repeated the co-occurrence analysis stratifying districts into low (n=117), medium (n=197), and high (n=185) *Pf*CRT prevalence groups (**Extended Data 8, Supplementary Table 6**). This stratification provided important additional insights. In the low-prevalence stratum, *Pf*K13 R622I significantly co-occurred with several *Pf*CRT

markers, including N326S (FDR-adjusted $p < 0.0001$) and K76T, M74I, N75E, A220S, Q271E, and R371I (all FDR-adjusted $p = 0.0001$). In the moderate-prevalence stratum, *Pf*MDR1 Y184F co-occurred significantly with *Pf*K13 R622I (FDR-adjusted $p = 0.035$), whereas associations with *Pf*CRT variants were no longer significant. In the high-prevalence stratum, no significant associations were detected. These results support the reviewer's point: in settings where *Pf*CRT variants are at moderate to high prevalence, associations with *Pf*K13 weaken or disappear, suggesting that part of the national-level signal reflects population structure and background allele frequencies. However, the persistence of strong co-occurrence signals in the low-prevalence stratum indicates that the associations involving R622I are not entirely artifacts of *Pf*CRT near-fixation. Together, these complementary analyses suggest that while population structure explains part of the signal, the co-occurrence of R622I with multidrug-resistance backgrounds remains detectable under lower background prevalence, raising the possibility of a biological interaction that warrants further investigation.

Third, we stratified study sites by transmission intensity (low, moderate, and high). *Pf*K13 R622I showed strong co-occurrence with multiple *Pf*CRT variants in moderate transmission settings, but was linked only to A220S and N326S in low transmission areas and showed no association at high transmission. In contrast, *Pf*MDR1 Y184F was strongly associated with R622I and all PfCRT variants primarily in high transmission settings, with fewer associations observed in moderate transmission sites.

Together, the geographic restriction, transmission intensity and *Pf*CRT prevalence-based stratified analyses provided a more nuanced picture of co-occurrence, showing that most associations persist locally in the northwest where *Pf*K13 variants are frequent, and that signals are not purely driven by *Pf*CRT's overall abundance.

These additional analyses are now included in the results section (lines 220-238): *"When stratifying the analysis based on geographical clusters, this significant co-occurrence between PfK13 R622I and PfCRT and PfMDR1 variants only persisted in the north-western districts, where R622I is present. In addition, as PfCRT variants were highly prevalent in our dataset, we repeated this analysis by stratifying districts based on PfCRT variant frequency (low: n=117; moderate: n=197; high: n=185) to confirm that the observed co-occurrence was not simply driven by its overall abundance (**Supplementary Table 6**). In the low-prevalence stratum, the association between PfK13 R622I and PfCRT resistance markers was especially strong. PfK13 R622I significantly co-occurred with PfCRT N326S (false discovery rate (FDR)-adjusted p<0.0001) as well as with the variants K76T, M74I, N75E, A220S, Q271E, and R371I (all FDR-adjusted p=0.0001). In the moderate-prevalence stratum, the co-occurrence of PfMDR1 Y184F and PfK13 R622I was significant (FDR-adjusted p = 0.035), but associations with PfCRT variants were not significant. In the high-prevalence stratum, no statistically significant co-occurrence between any tested mutation pairs was detected except for PfMDR1 Y184F, that showed strong co-occurrence with R622I and all variants of PfCRT. Lastly, as the districts in this study have a range of malaria transmission intensity, we also repeated this analysis stratifying sites based on API into low (0-10), moderate (10-50), and high (≥50) transmission settings. This revealed that PfK13 R622I co-occurred significantly with PfCRT resistance markers primarily in moderate-API areas, while PfMDR1 Y184F co-occurred with PfCRT variants mainly in high-API settings, indicating that transmission intensity influences variant co-occurrence patterns (**Extended Data 8, Supplementary Table 6**)."*

We have added a statement to the Discussion to interpret these locus-specific findings in light of regional population history and transmission intensity (lines 327-336): *"Our findings of frequent co-occurrence between PfK13 R622I and multidrug-resistance markers must be interpreted in the context of high*

background frequencies of PfCRT and PfDHFR/PfDHPS variants in Ethiopia. To address this, we conducted three complementary sensitivity analyses: stratifying the sites by geography, including a cluster with exclusively north-western sites, where R622I is most frequent, and stratifying districts by PfCRT prevalence and by API. Both geographical and PfCRT prevalence grouping approaches confirmed that the observed associations were not solely driven by background allele frequencies. The persistence of associations in low-PfCRT districts and in the north-western subset suggests that the linkage between R622I and multidrug-resistance backgrounds may reflect underlying selective or biological interactions rather than random co-occurrence. These results highlight the importance of geographically stratified surveillance to disentangle population structure from genuine genetic associations. Patterns of significant co-occurrence varied by API, suggesting that transmission intensity shapes variant associations."

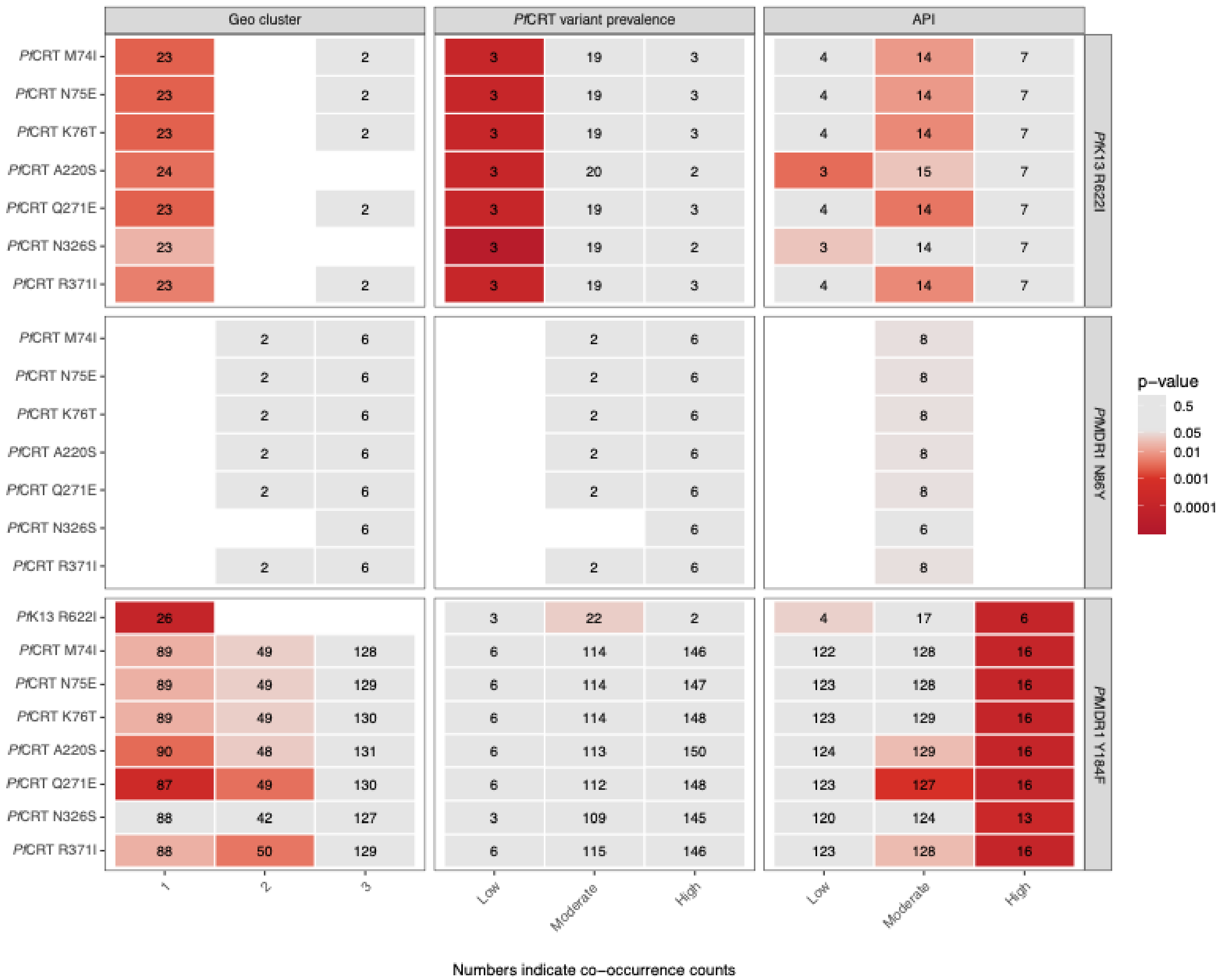

Extended Data 8: Heatmap illustrating the statistical significance of variant co-occurrence across different strata, including geographical cluster, *Pf*CRT variant prevalence and API. Each tile represents a specific variant pair within a stratum group, with variant 1 on the left-hand side and variant on the right-hand side of the heatmap. The color intensity of the tiles signifies the statistical significance (p-value) of the co-occurrence, with darker red indicating more significant P-values. Numbers indicate co-occurrence counts in each tile. Tiles where there is a co-occurrence count of ≤1 are shown in white. The x-axis displays the various groups within each stratum type, such as low, moderate, and high for API and *Pf*CRT variant prevalence (determined by seven key *Pf*CRT variants M74I, N75E, K76T, A220S, Q271E, N326S, and R371I), and clusters 1, 2, and 3 for geography. Geographical cluster 1 included areas in the North/West (districts Dera, Guba, K. Humera, Metema, Pawe, Gondar, and Tanqua Abergelle), cluster 2 includes districts Dasenech, Mizan, Salamago and Gambella zuria, while cluster 3 includes Amibara,

Batu/Zeway, Fedis, and Fentale. The underlying data are presented in **Supplementary Table 6.** API = Annual Parasite Index.

It would be useful to know the distribution of samples that contain more than one parasite genome. This is particularly important in areas of higher transmission – in those samples that contain more than one genome, it is not possible to conclude that a single parasite contains multiple mutations and this could impact the conclusions drawn from the data in Figures 3 and 4. Since the data includes sites with a range of transmission intensity, the data cannot be combined in a single analysis.

Response 1.7: We thank the reviewer for highlighting this important point. We agree that mixed infections can complicate the interpretation of co-occurring mutations. Unfortunately, due to the absence of highly variable genomic regions in our sequencing data, we are unable to accurately estimate the multiplicity of infection (MOI) for each sample. To mitigate this issue, we have revised our analysis to exclude potential mixed infections from the co-occurrence analysis. Specifically, samples were excluded for a given position if their variant allele frequency was between 5% and 95%, effectively removing positions with evidence of multiple genomes. We have added the following to the Methods section to clarify this approach (lines 497-498):

"For each pairwise comparison, samples indicating mixed-genotype infections (variant allele frequency ≥5% and ≤95%) at the relevant loci were excluded."

While we cannot directly assess MOI, this filtering step minimizes the impact of mixed infections on our conclusions. We have also added a text in the legend of Figure 3 "*To reduce the impact of mixed infections, positions with variant allele frequencies between 5% and 95% were excluded from the co-occurrence analysis. This ensures that the co-occurring mutations depicted are likely present within a single parasite genome. Samples with evidence of multiple genomes at a given position were therefore not included, minimizing potential bias from higher transmission areas where mixed infections are more common*."

In the identity-by-descent analysis in Figure 4**,** only samples deemed monoclonal by calculating the inbreeding coefficient (Fws>0.95) are included in the analysis. This has now been added to the methods section and Figure 4 legend.

In order to analyse variant co-occurrence in different intensities, we stratified the districts by annual parasite index (API) into low (0-10), moderate (10-50), and high (≥50) transmission settings (**Extended Data 8, Supplementary Table 6**). We found that co-occurrences between *Pf*K13 R622I and *Pf*CRT resistance markers were mostly significant in the moderate API group, while co-occurrence between *Pf*MDR1 Y184F and *Pf*CRT variants were mostly significant in the high API stratum, agreeing with the reviewer's point of the effect of transmission intensity on analysis results. The results from this stratified analysis have now been added to the results section (lines 233-238): *"Lastly, as the districts in this study have a range of malaria transmission intensity, we also stratified this analysis by annual parasite index (API) into low (0-10), moderate (10-50), and high (≥50) transmission settings. This revealed that PfK13 R622I co-occurred significantly with PfCRT resistance markers primarily in moderate-API areas, while PfMDR1 Y184F co-occurred with PfCRT variants mainly in high-API settings, indicating that transmission intensity influences variant co-occurrence patterns (**Extended Data 8, Supplementary Table 6**)."*

This is also discussed in the discussion (lines 327-336), as mentioned in **response 1.6**.

The manuscript would be greatly strengthened by the inclusion of either clinical response data or in vitro drug resistance studies. It would also be strengthened by an in-depth analysis of a few of the sites where Pfkelch13 mutations have high frequency,

Response 1.8: We thank the reviewer for this suggestion. In our study, we identified three non-synonymous *Pf*K13 propeller domain variants with allele frequencies above 1%: R622I, A675V, and P441L. Each has been previously assessed clinically and/or in vitro and is recognized by the WHO as validated (R622I, A675V) or candidate (P441L) marker of artemisinin resistance (ART-R). To highlight phenotypic evidence, we added to the Discussion (lines 307-312): *"Our findings on the emergence and dissemination of PfK13 variants linked to partial ART-R, notably R622I (present in 10% of samples overall, with peaks of 48.6% in Gondar and 16.7% in K. Humera) and A675V (1.7% overall, primarily detected in Gambella Zuria at 16.3%), add to the growing body of evidence on partial ART-R in Africa [8,13,34,35]. These mutations have been clinically and experimentally assessed and are WHO validated markers of ART-R [60]. We also identified a WHO candidate marker of ART-R, P441L, to be present in 1.1% of samples, primarily in the centre of the country."*

We acknowledge the absence of phenotypic data in our study and have clarified this in the limitations (lines 396-397): *"Our findings are based on established genotype–phenotype associations, but future research integrating clinical, in vitro, and genomic data will be crucial to validate and expand these insights."*

We have responded (**Response 1.2**) to the sampling method and representation of the northwest settings in the country. In brief, samples were collected from 15 sites nationwide, including seven sites in the northwest where R622I was previously reported, totalling 260 samples. In five of these sites, R622I occurs at high frequency (16–48%), representing a robust sample for analysis. To help address these comments of the reviewer, we added the following (lines 393-396): *"Given Ethiopia's complex malaria ecology, broader approaches such as multiplex or whole-genome sequencing, particularly in areas with high frequencies of resistance-conferring PfK13 variants, could uncover additional background factors not addressed in this study."*

These additions clarify the relevance of the identified variants, the robustness of our sample size, and the scope for future phenotypic studies. Following comments from the reviewer, we now have analyzed the northwestern datasets separately in the co-occurrence analysis and this unveiled new insights, and we are grateful for the comments the reviewer provided.

Reviewer #2 (Remarks to the Author):

This paper presents a detailed study of key drug resistance genes following genomic surveillance, their prevalence and geographic location within Ethiopia. They demonstrate evidence of multi-drug resistance including persisting mutations to discontinued drugs, identify epidemiologically distinct P. falciparum isolates, and further document the spread of ART-R markers in Africa. These results contribute to the understanding of emerging drug resistance in particular ART-R in Africa which presents a real threat to the effectiveness of many frontline antimalarials. The findings can also guide anti-malarial policy decision making in Ethiopia.
Overall the data is of high quality, generally well presented and with convincing conclusions. The referencing is also well done and appropriate. There seem to be a few minor inconsistencies between the data as discussed in the main text and shown in the supplementary and extended tables. Lots of Track Changes are still unresolved in the manuscript file.
Response 2.1: We apologize for the lingering tracked changes. All tracked changes have now been resolved in the clean version of the manuscript. We checked and resolved any minor inconsistencies that we detected.

Specific remarks:
• At the end of the introduction, lines 80-82, it would be helpful to already specify between which years the samples were collected and analyzed (2020-2022?).
Response 2.2: Specification on sample collection years has been added at the end of the introduction (line 82-84):
"We conducted a nationwide genomic surveillance of P. falciparum between 2019 and 2023 in 15 Ethiopian districts with varying disease ecology and risk status, focusing on markers of resistance to chloroquine, sulfadoxine-pyrimethamine (SP), artemisinin, and ACT partner drugs."

In addition, an indication of collection years has been added to Table 1 as well.

• The text in Figures 1B and C is hard to read on the darker colors. Consider putting a white outline on the letters or adjusting the background colors.
Response 2.3: We have now revised Figures 1B and 1C. As the study health facilities are the same as in Figure 1A and those are mentioned in a clear background, we decided to remove the labelling in 1B and 1C. Only the points indicating the specific locations of the study sites are now indicated in black dots. We believe this change improves clarity of the figure.

• Line 99, median age is stated as 20.2 years across the participants, but from Table 1 this appears to be the median of medians not the true median.
Response 2.4: We thank the reviewer for pointing this out. We have recalculated the true median age across all participants, which is 20.0 years. This value has been updated in Table 1 and corrected in the text (Line 104).

• Figure 2 appears to be duplicated. Track changes need to be resolved.
Response 2.5: The figure 2 duplicate has now been removed.

• Line 103, this subtitle could read better as "The persistence of pfdhfr and pfdhps mutations associated with antifolate resistance".
Response 2.6: This subtitle has now been rephrased accordingly.

• Lines 117 – 121, discussion around Extended Data 4A, there seem to be discrepancies. What criteria was used for a mutation to be considered prominent in a region? The coloured regional sections on the pie chart visually are not in a clear majority for the regions being called prominent. For example:
o Line 118: "the triple mutant ARINI (Cluster II, 43.1%) predominating in Batu, Fedis, and Mizan". Why not also Fentale?
o Line 119: "AICNI (Cluster I, 38.7%) in Gambella and Gondar". Why Gambella but not K. Humera and Metema?
o Line 119: "the wild-type (Cluster III, 16.1%) in Guba". Why not also Pawe?
o Line 120-121: "For PfDHPS, the K540E-containing Cluster I was frequent in Amibara, Batu, and Fedis". Should this instead be Fentale ahead of Amibara?
o Line 121: "G437A-dominant Clusters II and V were more common in Guba (Extended Data 4B)". Why not also Pawe and Salamango?
Response 2.7: we thank the reviewer for these questions and apologise for the oversight. Upon review, we revised this section to accurately reflect the regions in which these haplotypes are dominant. The updated text now reads (lines 138-144) :
*"This network revealed three main PfDHFR clusters: the triple mutant A**IRN**I (Cluster II, 43.1%) predominating in Batu, Fedis, Fentale, Mizan, Amibara, Dasenech and Selamago; the double mutant A**I**C**N**I (Cluster I, 38.7%) in Dera, Gondar, Gambella, Metema, K. Humera and Abergele; and the wild-type (Cluster III, 16.1%) in Guba and Pawe **(Extended Data 6A)**. For PfDHPS, K540E-containing Cluster I was frequent in Fentale, Batu, and Fedis, whereas G437A-dominant Clusters II and V were more common in Guba, Pawe and Salamago **(Extended Data 6B)**."*

This revision ensures that all regions with significant representation of the respective haplotypes are correctly acknowledged and aligns the text with the data shown in the Extended Data figures.

In the caption of Extended Data 4 different conclusions are drawn:
o Caption states "PfDHFR clusters II (257) and I (231) were predominantly found in Fentale", yet in the main text Line 118 and 119 make no mention of Fentale.
o Caption states "PfDHPS clusters I (285) and II (136) were shared between Fedis and Fentale" yet the main text Line 120-121 make no mention of Fentale and mention other locations as being predominant. It is also not clear from the graphs that these are the predominant regions. For example, cluster II for PfDHPS (Panel B) does not show Fentale or Fedis (orange colors) at all.
Response 2.8: We thank the reviewer for highlighting these inconsistencies. In line with our response in **Response 2.7**, we have now revised the caption of **Extended Data 6** and in agreement with the main to accurately reflect the regions where each haplotype is dominant. The updated caption now correctly lists the regions with significant representation of each cluster and aligns with the visual data in the figure:

Extended Data 6. *Median-joining networks of A) PfDHFR (N=596), B) PfDHPS (N=582), C) PfCRT (N=488), D) PfMDR1 (N=546) and E) PfK13 (N=584) haplotypes. Circle size represents haplotype frequency and colors indicate geographic origin. Connecting lines indicate single nucleotide changes and arrows denote key mutations associated with resistance. Haplotypes are indicated using Roman numerals. PfDHFR cluster II (the triple mutant A**IRN**I, 43.1%)) was predominantly found in Batu, Fedis, Fentale, Mizan, Amibara, Dasenech and Selamago , while cluster I (the double mutant A**I**C**N**I, 38.7%) was predominantly found in Dera, Gondar, Gambella, Metema, K. Humera and Abergele, and the wild-type (Cluster III, 16.1%) in Guba and Pawe. PfDHPS cluster I containing K540E was frequent in Fentale, Batu, and Fedis, whereas G437A-dominant Clusters II and V were more common in Guba,*

Pawe and Salamago. PfCRT haplotype clusters V (n=117) and X (n=42), along with PfMDR1 haplotype clusters III (n=434) and I (n=75) were found across several districts. Major PfK13 haplotypes were I (n=375) and III (n=104). PfK13 R622I (n=57) concentrated in Gondar (n=14), K. Humera (n=7), Amibara (n=1), Pawe (n=2), Dera (n=9), Metema (n=10). R255K (n=40): Guba (n=11), Dera (n=7), Pawe (n=7), Salamago (n=4). A675V (n=10): Gambella, Mizan, Salamago. P441L (n=6): Batu/Zeway (n=5), Salamago (n=1). R622I, R255K, A675V, P441L clustered in north-central, west, southwest, south-central regions, respectively.

• Line 145 – 147, the data referring to the Y184F codon is not found in Extended Table 3. Perhaps it should be Supplementary Table 1. But within Supplementary Table 1 the Y184F values range from 75.8% in Amibara to 100% in others, why was the 75.8% in Amibara disregarded in the main text which states that the range is from 97% in Fentale: "the Y184F codon, appearing in 93.5% (503/538) of samples and detected in all districts, with prevalence ranging from 97.0% in Fentale (32/33) to 100% in several districts (Batu, Dera, Fedis, Guba, K. Humera, Mizan)"
Response 2.9: We thank the reviewer for this comment and apologise for the oversight. The sentence has now been revised to accurately reflect the full observed range, including the 75.8% observed in Amibara (lines 170-173):
"The variation in the PfMDR1 haplotype was primarily driven by the Y184F codon, appearing in 93.5% (503/538) of samples and detected in all districts, with prevalence ranging from 75.8% in Amibara (25/33) to 100% in several districts (Batu, Dera, Fedis, Guba, K. Humera, Mizan, Tanqua Abergelle) ***(Supplementary Table 1).****"*

• Line 167, 17% should be 1.7%
Response 2.10: This has now been corrected to 1.7%.

• Line 200, SNP abbreviation has not been defined in the main text.
Response 2.11: This has now been written in full at this point 'Single Nucleotide Polymorphism' and the abbreviation used afterwards.

• Line 675, Table 1 heading, Sequenced samples (n)*, what does the '*' refer to?
Response 2.12: The * in table 1 was a typo and has now been removed.

• Line 702, is a duplicate of Figure 2
Response 2.13: The Figure 2 duplicate has now been removed

• Line 717, Figure 3B, D and E are too small to see the data. Also Figure 3 has 2 captions, the second is more detailed and more informative than the first. Within the caption Line 752 SNP abbreviation has not been defined, and the SNPs were referred to as "resistance markers" in part A of the figure so it becomes confusing.
Response 2.14: We thank the reviewer for these observations. Figure 3 has been revised to improve visibility and clarity of panels B, D, and E. The caption has been reworked to remove the redundant version, retaining the more detailed and informative text.

• Supplementary Table 1. How is the Overall N calculated? For example, in the row for pfmdr1 N1042, the sum of N for each of the regions is 584, while the N for the overall column is 531, how is this possible? This then led to a query in Line 148-149 where the codon N1042 is described as entirely

wildtype which is correct for the Overall column, but when considered by region some areas such as Gondar are as low as 68%.

Response 2.15: Our methodology defines 'N' as the number of samples with a successful genotype call at a given marker position. This explains why the overall N for N1042 (531) does not equal the sum of all regional samples analysed in this study (584); specifically, 53 samples could not be genotyped at this position and were therefore excluded from the prevalence calculation.

As the reviewer pointed out, it is not possible for the overall wildtype prevalence to be 100% if, for example, Gondar was reported at 68.2%. The 68.2% figure in the original Gondar column was due to a calculation error, which we have now corrected.

The revised **Supplementary Table 1** accurately reflects the data for the *Pf*MDR1 N1042 wildtype variant at both District and overall levels. Across *Pf*MDR1 variant type successfully genotyped, we detected no mutant alleles at codon 1042 (N1042), 1034 (S1034) and 1246 (D1246) positions.

Accordingly, we corrected and revised prevalence of wildtype type variant across District, with N representing the numbers of samples successfully genotyped for specific marker and, consequently. The corrected overall column now accurately reflects this.

We have also clarified how N is calculated in the methods section (lines 483-486):

"In the prevalence tables, N indicates the number of samples successfully genotyped for each specific loci. The sample size (N) varies between loci because prevalence was calculated using only samples with definitive genotype data for that position."

We thank the reviewer again for their valuable input, which helped improve the accuracy and clarity of our supplementary materials.

Reviewer #2 (Remarks on code availability):

The code is generally comprehensive, suitably annotated and should be a usable resource for the community. The code does provide a README file, although the README could contain a more detailed description of the various codes and more information on how to use/install/run the codes. There could also be more information on which prerequisite files and software are needed to allow the full running of the code. The current README appears to be more of an index.

Response 2.16: The github repository has now been revised to categorise files into bioinformatics analysis, statistical analysis and visualisations, with the aim of improving oversight. Information on how to run the scripts, as well as information on prerequisite files has been added.

Reviewer #3 (Remarks to the Author):

Overall, I appreciate the data presented and its importance in understanding selection of artemisinin partial resistance and ACT partner drug resistance in Ethiopia, particularly given the opportunity it provides to gleam information on how the use of multiple first line therapies (in this case the use of AL to treat Pf and CQ to treat Pv in the same locations) may impact on the selection of ACT resistance. However, I believe that the presentation of the data should be greatly simplified to better communicate key take away messages. In addition, the authors need to use more nuance language in describing the impact of certain mutations on drug susceptibilities and in explaining interpretations of data. Also, the deep exploration of the data using different analytical methods is admirable, but presenting more analysis without providing more information on the motivation and data interpretation can actually be more confusing. I spent a lot of time trying to understand what additional information complementary analyses added, especially when methods were not clearly described. Specifically, I found the focus on haplotypes to be unnecessarily complicated, especially as found some of the analyses to be a bit redundant. It was unclear to me what some of the analyses added to the story. For example,
Response 3.0: We thank the reviewer for the thoughtful and detailed assessment of our manuscript. We greatly appreciate the recognition of the importance of our data in understanding the selection of partial artemisinin resistance and ACT partner drug resistance in Ethiopia, particularly in the context of multiple first-line therapies used within the same regions. We acknowledge the reviewer's concerns regarding the complexity of data presentation, the need for nuanced language when describing the impact of specific mutations, and the necessity of clearer explanation of the motivation and interpretation of complementary analyses. In response, we have undertaken a comprehensive revision to: simplify and clarify data presentation, focusing on key take-home messages; revise language throughout the manuscript to ensure careful and nuanced discussion of mutations and their effects on drug susceptibilities; provide detailed explanations in the Methods section for all analytical approaches, including the origin of datasets, calculation methods, and rationale for complementary analyses; ensure consistency between main text, figures, and supplementary materials. We believe these revisions improve clarity, facilitate interpretation, and enhance the accessibility of the key findings to readers. We carefully addressed the specific comments the reviewer provided below.

1. Can the authors clarify the origin of panels 1b and 1c? No reference is given indicating they are generated from previously published data, nor is there any information in the methods section to indicate how the data were collected/generated/analyzed. I recognize that more information is included in the legend of the figure, but would prefer there is at least some mention of this in the methods section as well.
Response 3.1: We thank the reviewer for this feedback. Information on how these maps were generated has now been added to the methods section (lines 419-430):
"To assess malaria transmission intensity and characterise the distribution of P. vivax, we utilized malaria surveillance data from the Public Health Emergency Management (PHEM) system. The dataset covers confirmed malaria cases at the district level in Ethiopia from 2020 to 2022. Population projections for the corresponding districts were obtained from the Ethiopia Office of OCHA (https://data.humdata.org/organization/ocha-ethiopia) to serve as denominators in prevalence calculations. The Annual Parasite Index (API) was calculated as the number of total confirmed malaria cases per year divided by the population at risk, multiplied by 1,000 to express cases per 1,000 persons at risk per year. Malaria risk categories were assigned based on the Ethiopian Ministry of Health stratification: malaria-free (API = 0), very low ($0 < API \leq 5$), low ($5 < API \leq 10$), moderate ($10 < API \leq 50$), and high ($API > 50$). The proportion of P. vivax cases was calculated as the ratio of P. vivax

confirmed cases to the total confirmed malaria cases within each district. These proportions were aggregated and spatially mapped to illustrate geographic variation across the country."
This ensures that the origin, processing, and analytical approach for panels 1B and 1C are fully described, enhancing transparency and reproducibility as the reviewer suggested.

2. It is indicated that the mitochondrial genome was sequenced, but these data are never mentioned in the text. Extended data 1 suggests relatively poor coverage and a lack of variation. This should be mentioned in the results to explain there is no further analysis of the gene.

Response 3.2: We thank the reviewer for this observation. Extended Data 1 and 2 only include coverage and variation at positions of known drug resistance markers, which for the mitochondrial genome are only in the Cytochrome b gene. However, the entire mitochondrial genome was sequenced, and this data has been included in the population genetic analyses reported in the manuscript.
To improve clarity, we have now included a separate figure (Extended Data 3) showing coverage and variation across the entire mitochondrial genome. We have also added a brief statement in the Results section referencing Extended Data 3, highlighting mitochondrial genome coverage and variation, and explaining why no further gene-specific analyses are presented (lines 100-102).
"While the mitochondrial genome was sequenced with adequate coverage, variation was limited. ***Extended Data 3*** *illustrates coverage and detected variation across the mitochondrial genome, providing context for why further gene-specific analyses were not presented."*

3. I would suggest mentioning the "tagment" step in your methods section for the targeted sequencing.

Response 3.3: We have now added this step to the methods section (lines 461-463): *"During library preparation, PCR amplicons were tagmented with a transposome, simultaneously fragmenting the DNA and adding adaptor sequences in a single step."*

4. Section starting on line 104: I would suggest simplifying the haplotype. For example, since 16 and 164 are all WT, including these in the haplotype is not particular helpful, and remembering which amino acid is WT and which is mutant is a bit of a burden. In some of the tables/figures the mutant amino acids are bolded and underlined. Consider doing this in the text, or defining and using terms such as "dhfr triple mutant" or "dhps double mutant".

Response 3.4: We thank the reviewer for this comment. We have now bolded and underlined the mutant amico acids in all the haplotypes mentioned in the text and figures to improve clarity. Codons 16 and 164 are retained in the *Pf*DHFR haplotype designations (ANCSI/AN**RN**I/A**I**C**N**I/A**IRN**I) to maintain consistency with commonly used nomenclature in the literature as some of these are reported from other settings, while the emphasis on mutants ensures that readers can easily identify the relevant substitutions. This approach balances clarity for readers with standardized terminology for scientific communication.

5. In addition, the haplotypes are not clearly defined in the results section, so a reader who has not memorized the dhfr/dhps mutants would have difficulty knowing which locus each amino acid in the haplotype belongs to. It should be clearly explained that AIRNI means PfDHFR A16, 51I, 59R, 108N and I164 and so on.

Response 3.5: We have now included an explanation of each haplotype in the results section when it first appears in the text, with the aim of improving readability. We have also provided an additional table in the supplementary data (**Supplementary table 2**) that clearly lists all the haplotype combinations observed in this study for both PfDHFR and PfDHPS.
One important observation peculiar about this study is the unique widescale presence of haplotypes based on A**I**C**N**I (A16/N51I/C59/S108N/I164) which are uncommon in the literature, combined with

wild type (ISAKAA), double (IS**GE**AA) or triple (IS**GEG**A) *Pf*DHPS mutants and rare presence of *Pf*DHPS double mutant (IS**GE**AA) with the wild type of *Pf*DHFR (ANCSI). We have categorized these haplotype combinations as "uncommon" variants and provided a table in the supplementary (**Supplementary table 2**) and Figure **Extended data 5** to summarize the relative prevalence of the most common and uncommon variants and definitions.
The N51I+S108N double mutant observed in these uncommon haplotype combinations is is reported from East Africa, but the frequency tends to be much lower than the triple mutant. This combination is usually an intermediate form that appears on the evolutionary path toward the more common triple mutant (N51I+C59R+S108N = A**IRN**I). A stepwise accumulation of mutations in *Pf*DHFR, including intermediate double mutants (like N51I+S108N) before the fixation of the classic triple mutant is reported from South and East Africa (Roper et al. 2003 Lancet - https://pubmed.ncbi.nlm.nih.gov/12686039/) and in an *in vitro* study (Sirawaraporn et al 1997 Proc Natl Acad Scie - https://pmc.ncbi.nlm.nih.gov/articles/PMC19755/). The premise is that the emergence of drug resistance involved sequential selection of single point mutations, starting from the least resistant single mutant that survived selection, S108N, and proceeding stepwise to the more resistant multiple mutants.

Supplementary Table 2. List of all the *Pf*DHFR and *Pf*DHPS haplotype combinations observed in this study.

Category - Standard convention	Haplotype	*Pf*DHFR haplotype (codons 16, 51, 59, 108, 164)	*Pf*DHPS haplotype (codons 431, 436, 437, 540, 581, 613)	Number of mutations	Resistance Phenotype	Typical distribution	References
Common	Wildtype	$(A_{16}N_{51}C_{59}S_{108}I_{164})$	$(I_{431}S_{436}A_{437}K_{540}A_{581}A_{613})$	0	Fully sensitive	Historical, pre-SP era	Peterson, et al. Proc Natl Acad Sci (1998)
	Triple	$(A_{16}\underline{\mathbf{I_{51}R_{59}N_{108}}}I_{164})$	$(I_{431}S_{436}A_{437}K_{540}A_{581}A_{613})$	3	High pyrimethamine resistance	Common across Africa	Plowe et al. J Infect Dis. (1997)
	Quintuple	$(A_{16}\underline{\mathbf{I_{51}R_{59}N_{108}}}I_{164})$	$(I_{431}S_{436}\underline{\mathbf{G_{437}E_{540}}}A_{581}A_{613})$	5	High-level SP resistance/fully resistant haplotype	Widely prevalent in East Africa (Ethiopia, Uganda, Kenya, Tanzania)	Brhane et al. Commun Med (2025), Kublin et al. J Infect Dis (2002), Spalding et al. Mal J (2010)
	Sextuple	$(A_{16}\underline{\mathbf{I_{51}R_{59}N_{108}}}I_{164})$	$(I_{431}S_{436}\underline{\mathbf{G_{437}E_{540}G_{581}}}A_{613})$	6	Very high resistance (SP failure likely)	Increasing in East Africa, especially Uganda, Rwanda and Tanzania	White et al. Sci Rep (2025), Juliano et al. Lancet Microbe (2024)
Uncommon	A**I**C**N**I/ISAKAA	$(A_{16}\underline{\mathbf{I_{51}}}C_{59}\underline{\mathbf{N_{108}}}I_{164})$	$(I_{431}S_{436}A_{437}K_{540}A_{581}A_{613})$	2	The N51**I**+S108**N** double mutant is a well-recognised *Pf*DHFR haplotype, though it is usually an intermediate form that appears on the evolutionary path towards the more common triple mutant (N51**I**+C59**R**+S108**N** = A**IRN**I). Studies from East Africa (e.g. Tanzania, Kenya, Uganda) have documented double mutants including N51**I**+S108**N**, but their frequency tends to be much lower than the triple mutant.		Roper et al. Lancet (2003)
	A**I**C**N**I/IS**GE**AA	$(A_{16}\underline{\mathbf{I_{51}}}C_{59}\underline{\mathbf{N_{108}}}I_{164})$	$(I_{431}S_{436}\underline{\mathbf{G_{437}E_{540}}}A_{581}A_{613})$	4			
	A**I**C**N**I/IS**GEG**A	$(A_{16}\underline{\mathbf{I_{51}}}C_{59}\underline{\mathbf{N_{108}}}I_{164})$	$(I_{431}S_{436}\underline{\mathbf{G_{437}E_{540}G_{581}}}A_{613})$	5			
	AN**RN**I/IS**GE**AA	$(A_{16}N_{51}\underline{\mathbf{R_{59}N_{108}}}I_{164})$	$(I_{431}S_{436}\underline{\mathbf{G_{437}E_{540}}}A_{581}A_{613})$	4			
	ANCSI/IS**GE**AA	$(A_{16}N_{51}C_{59}S_{108}I_{164})$	$(I_{431}S_{436}\underline{\mathbf{G_{437}E_{540}}}A_{581}A_{613})$	2			

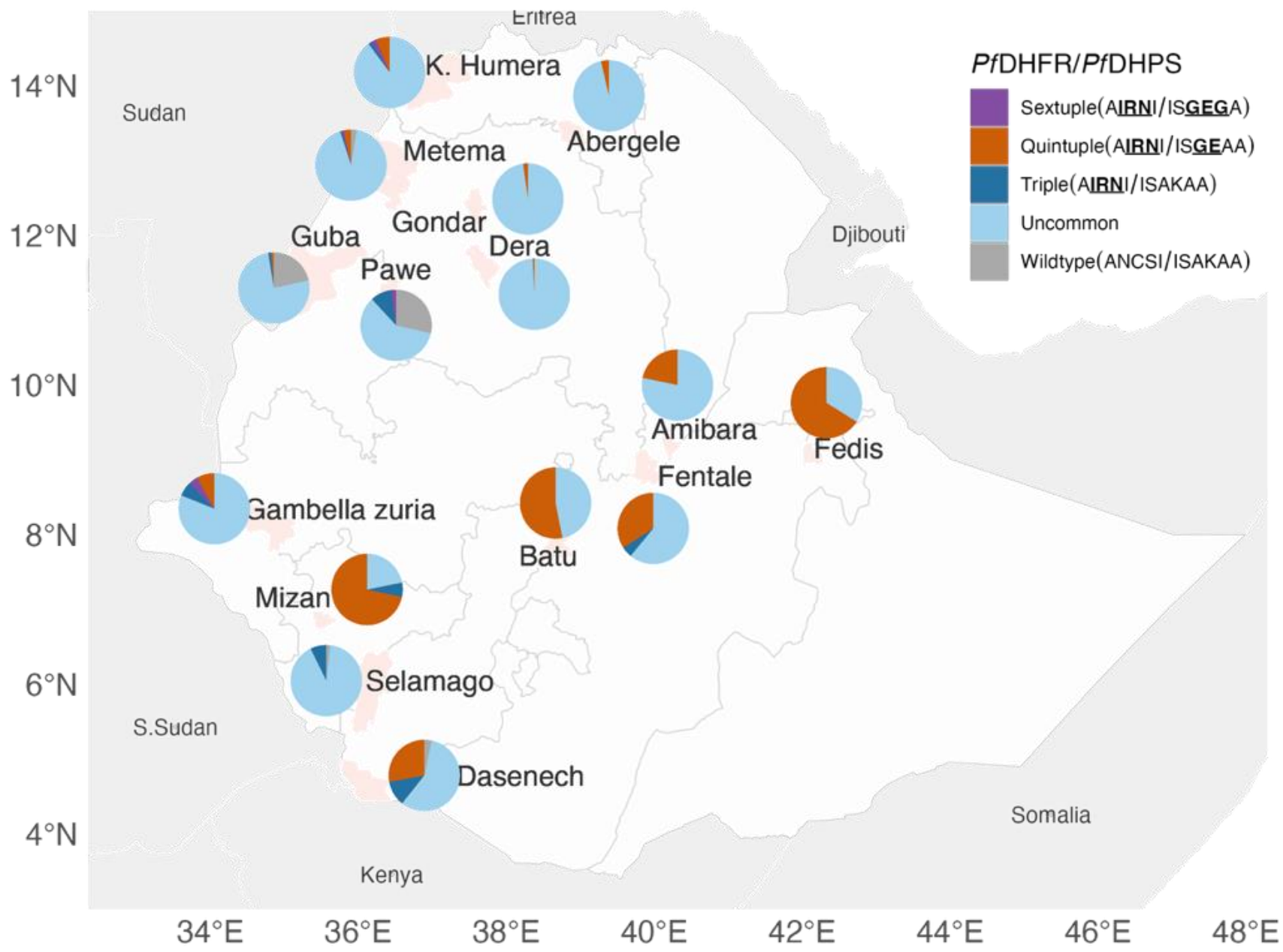

Extended data 5. Geographic distribution and prevalence of combined *Pf*DHFR and *Pf*DHPS haplotypes in Ethiopian *P. falciparum* populations.

6. I suggest adding gene labels to figure 2. It is unclear why the dhfr and crt data is presented in one color scheme and the dhps and mdr1 data in another. I think it would be more clear to use the same color scheme across panels. Also, the inconsistency in the legend of whether the panel label comes before or after the description of the panel is confusing

Response 3.6: We thank the reviewer for these suggestions. We have now added gene labels to figure 2 and the figure has been revised so that the same colour scheme is used consistently across all panels. Specifically, in all panels (2A-2D): light blue represents wild-type haplotypes, dark blue corresponds to the lowest number of mutant variants present (typically single-mutant haplotypes; if absent, then double-mutant haplotypes, etc) and orange and violet colours represent the remaining mutant combinations in more diverse genes. Additionally, the figure legends have been standardized, ensuring consistent placement of panel labels before the corresponding description throughout all panels. We hope these changes improve clarity and facilitate interpretation of the data.

7. Section starting on line 104: I recommend adding a sentence trying to summarize the geographical distribution (or lack there of) of the antifolate mutations (regional level, rather than site level). I feel that this will strengthen your point in the section starting at 156 when the association between SP and CQ markers and Pv prevalence is made.

Response 3.7: Thank you for this constructive suggestion. Following comments 4 and 5 from the same reviewer, we revised our presentation of the SP resistance markers to better illustrate their spatial distribution. We have now added an additional map (**Extended data 5**) presenting the combined geographic distribution of *Pf*DHFR and *Pf*DHPS haplotypes, highlighting the most epidemiologically relevant variants (i.e., *triple*, *quintuple*, and *sextuple* haplotypes) separately from uncommon local variants.

Our updated analysis shows that the quintuple haplotype (N51I + C59R + S108N + A437G + K540E) predominantly clusters in sites located along major transportation corridors, notably the Djibouti to Addis Ababa and Kenya to Addis Ababa routes, where historical drug pressure and population mobility have likely facilitated gene flow and persistence of resistant lineages. These areas, however, were less affected by the recent malaria resurgence observed since 2019. In contrast, uncommon *Pf*DHFR and

*Pf*DHPS variants were more frequent in northwestern and southwestern Ethiopia, regions that have experienced substantial transmission increases in recent years. These geographical trends are consistent with the single-gene haplotype distributions observed for both *Pf*DHFR and *Pf*DHPS loci.
These spatial patterns align with prior molecular surveillance studies indicating regional differentiation in SP resistance markers across Ethiopia, shaped by historic SP use, variations in transmission intensity, and gene flow from neighboring countries.

We have now added a section that briefly describes this (lines 132-136): *"The PfDHFR–PfDHPS quintuple haplotype (N51I + C59R + S108N + A437G + K540E) predominated along major transportation corridors, particularly the Djibouti–Addis Ababa and Kenya–Addis Ababa routes, while uncommon variants carrying A**I**C**N**I were concentrated in northwestern and southwestern regions that experienced recent malaria resurgence."*

And also in the discussion (lines 287-298): *"The high-resistance PfDHFR–PfDHPS quintuple mutant (N51I + C59R + S108N + A437G + K540E), a key determinant of SP resistance, was primarily concentrated along major transport routes, likely reflecting historical drug use and human-mediated gene flow. In contrast, globally uncommon haplotypes were more prevalent (40.8% nationwide) in northwestern and southwestern regions that have recently experienced malaria resurgence, consistent with previous reports of heterogeneous selection pressures across ecological zones indicating regional differentiation in SP resistance markers across Ethiopia and East Africa [56,57]. Notably, the N51I+S108N PfDHFR double mutant dominated these uncommon haplotypes (A**I**C**N**I). These intermediate or novel variants, often co-occurring with wild-type or canonical PfDHPS alleles, support the stepwise model of PfDHFR evolution in which S108N arises first, followed by N51I and C59R, before fixation of the triple mutant [58,59]. Together, these patterns highlight the complex interplay of drug pressure, parasite evolution, and regional transmission in shaping the local landscape of antifolate resistance."*

8. I found it confusing that the minimal-spanning networks show novel missense mutations that are not reported in Supplementary Table 2. Also, I think it would be helpful to explicitly state that this analysis included all polymorphisms in the gene whereas the haplotypes included drug resistance loci of interest, and what additional information you intend to share (what does this add to your initial discussion of the haplotypes defined by drug resistance markers of interest).
Response 3.8: We thank the reviewer for this comment. This table is now presented as **Supplementary Table 3** and lists novel missense mutations identified by at least two variant callers and detected in a minimum of ten samples. In contrast, the minimal-spanning haplotype network analysis includes all detected non-synonymous polymorphisms to capture the full extent of genetic diversity, thereby providing a more comprehensive view of haplotype evolution and gene-wide variation.To clarify this, we have added the following to the Methods section (lines 518-520):
"To visualise gene-wide haplotype diversity and geographical spread, minimal-spanning networks were constructed using all observed non-synonymous polymorphisms within coding sequence of each gene."

This sentence in the methods section explains the criteria for reporting of novel SNPs (lines 477-478): *"Novel SNPs were reported only if they were detected by at least two variant callers and were present in at least ten samples."*
Additionally, the following statement has been added to the results section, at the first mention of the minimal-spanning networks (line 137-138):

"To explore gene-wide diversity and evolutionary relationships among variants, we constructed a minimal-spanning network using all non-synonymous polymorphisms."
These additions clarify the distinction between the targeted haplotype analysis of resistance loci and the broader gene-wide haplotype analysis, helping readers understand the complementary insights provided by the two approaches.

9. Line 118: I believe there is a type. ARINI should be AIRNI
Response 3.9: Thank you, this typo has now been corrected.

10. Section starting at 140. I find the characterization of NFSND as "mutant" and a known secondary modulator of chloroquine resistance a bit misleading. Its actually the N86Y mutation that is most strongly associated with lumefantrine/chloroquine sensitivity in east Africa, while the role of Y184F is a bit less clear. In fact a recent meta-analysis suggests that it is not associated with susceptibility to the drug at all (https://pubmed.ncbi.nlm.nih.gov/40506728/). I think the more accurate message is that following the switch to AL, mdr1 86Y mutations have become less prevalent (a quick look at published papers showing earlier prevalences make me believe this is true), consistent with lumefantrine selection. Any discussion of 184 needs to be much more nuanced and recognize the limitations of this marker.
Response 3.10: We thank the reviewer for this feedback. While the NFSND haplotype was originally marked as "mutant" because of its difference with the reference genome, we agree that referring to this haplotype especially when discussing its impacts on drug resistance/susceptibility can be misleading. In particular, the role of Y184F in lumefantrine/chloroquine resistance is less pronounced than that of N86Y.
In response, we have:

1. Rephrased all mentions of the NFSND haplotype to be more nuanced
2. Emphasized the observed decline in the 86Y variant, consistent with lumefantrine selection following AL rollout.
3. Added nuanced discussion of Y184F, highlighting the uncertainty of its impact on drug susceptibility especially in settings like Ethiopia.

We hope these changes provide a more accurate and balanced interpretation of *Pf*MDR1 variants in the context of antimalarial selection.

11. While I appreciate the efforts the authors took to present data in a visual, I do not think Extended Data 5 was helpful. I recommend sticking to Supplementary Table 3a/b. Also, it is not standard to have separate "panels" for a table, so rename them Supplementary Table 3 and 4 or combine them into a single table. Finally, why are the K13 genotypes not included in the analysis?
Response 3.11: We thank the reviewer for these suggestions. This table is now **Supplementary Table 4**. In response, we have done the following: (i) The supplementary tables 4 have now been combined into one table as suggested, and the K13 genotypes have now been included. (ii) Regarding **Extended Data 7**, we included this visualization intentionally, as extended data is presented alongside the main text, enhancing readability and accessibility for the reader. While we understand the reviewer's preference for tables, we respectfully believe that the visual format in **Extended Data 7** more effectively communicates the distribution and patterns of haplotypes than a table alone. We believe these updates ensure that the data are both comprehensive and clearly accessible, while retaining the visual representation for improved interpretability.

12. Section starting on line 184. Is this "linkage" driven by interaction/fitness/adaptation or is it incidental because certain mutations are more prevalent in certain geographical regions?

Response 3.13: As we mentioned in **Response 1.6**, we agree that the high background frequency of *Pf*CRT variants can complicate interpretation of co-occurrence analyses. To address this concern, we have done sensitivity analysis; repeated the analysis stratified by *Pf*CRT prevalence at the district level (low: n=117; medium: n=197; high: n=185). This additional analysis, now included in the Results (**Supplementary Table 6** and **Extended Data 8**), demonstrates that the observed associations are not solely driven by the overall abundance of *Pf*CRT variants. Specifically, in the low-prevalence stratum, *Pf*K13 R622I significantly co-occurred with several *Pf*CRT markers, including N326S (FDR-adjusted $p < 0.0001$) and K76T, M74I, N75E, A220S, Q271E, and R371I (all FDR-adjusted $p = 0.0001$). Therefore, we believe that factors other than co-incidence is driving this co-occurrence.

In addition, we repeated the analysis after stratifying the isolates into three geographical clusters, with cluster 1 restricted to sites in northwest Ethiopia, where *Pf*K13 variants (including R622I) were most frequent (the seven sites where R622I is concentrated; n ≈ 260 samples). This approach minimizes confounding by regional population structure and background allele frequency. Results confirm that several of the national co-occurrence signals including the co-occurrence of *Pf*K13 R622I with *Pf*CRT variants and *Pf*MDR1 Y184F persist in this subset. In the other geographical clusters (cluster 2 representing regions in the South/Southwest (Dasenech, Mizan, Salamago and Gambella zuria) and cluster 3 in the East/South (Amibara, Batu/Zeway, Fedis, and Fentale)), no significant associations between PfK13 variants and *Pf*CRT and *Pf*MDR1 variants were found, mostly because of the near-absence of *Pf*K13 variants in these regions (**Extended Data 8**, **Supplementary Table 6**). These findings suggest that part of the co-occurrence signal is due to population structure.

As outlined in **response 1.6**, these findings have now been added to the results and discussion in the manuscript.

13. Line 193: I would be hesitant to use the term "genomic population structure" here, as it is really just based off of polymorphisms in drug resistance genes, isn't it? Maybe molecularly defined resistance profiles would be more accurate?
Response 3.14: The mitochondrial genome was included in this analysis as well. To clarify this to the readers we have now rephrased this as "Population genetic analysis based on mitochondrial genome sequences and molecularly defined drug resistance profiles."

14. Line 112: consider changing from "district-specific patterns" to "site-specific patterns", since there is only 1 site per district.
Response 3.15: This has now been updated to "site-specific" patterns.

15. It is hard to interpret some of the data shown in figure 3 because the small size/resolution makes it hard to read text. Simplifying may help, as key panels can be made larger. Consider dropping panel a (data can be or is described in text or supplemental figures)
Response 3.16: We have now revised this figure and moved the haplotype network from the figure to supplementary information. The figure has now been provided in high resolution.

16. In general, I dislike the term "mutant" being used for haplotypes where there are multiple different "mutant" combinations that confer different levels of resistance. I think it would be more helpful to make your point using individual SNPs or more precise terms like double or triple mutant in the case of dhfr and dhps.
Response 3.17: We thank the reviewer for this comment. We have now clarified this in the text, as detailed in **Response 3.4** and **3.5**.

17. Section beginning at line 184. What statistical test was used here?

Response 3.18: Associations of drug resistance mutation prevalence with epidemiological factors (overall transmission intensity by API and *P. vivax* co-endemicity) were evaluated using Spearman's rank correlation coefficient (ρ). The non-parametric method was used as our epidemiological and prevalence data was non-normally distributed.

We also improved the analysis by computing 95% confidence intervals for Spearman coefficients with 5000 bootstrap iterations, improving the reliability of correlation strength estimates, which is important due to different (effective) sample sizes across transmission strata.

This is described in the methods section (lines 492-496):

"Associations between haplotype frequencies, the annual parasite index (API), and the fraction of P. vivax and total infections were analyzed using Spearman's correlation. To adjust for small sample sizes and to achieve robust estimates, 200 bootstrap resampling iterations were used. The API was based on the total confirmed malaria cases and was calculated as follows: (total confirmed malaria cases per year ÷ population at risk) × 1,000."

18. For the section starting on line 202: which SNPs were used to make this neighbour joining tree? I would be hesitant to claim gene flow and connectedness if only using drug resistance genes, as relationships are likely driven more by drug selection pressure than identity by decent. You'd need more neutral markers, and there is no mention of additional genotyping/sequencing of the samples analyzed in this study. The same comment applies to the section starting with 209. This is more appropriately done with flanking haplotypes, but it is not clear this was done.

Response 3.19: SNPs from the mitochondrial genome were included in this analysis as well. However, to clarify this to the readers we have now rephrased this as (lines 247-250):

*"When isolates were placed in the context of publicly available P. falciparum genetic data from East Africa and Horn of Africa (**Fig. 4C-D**), a Single Nucleotide Polymorphism (SNP)-based neighbour-joining tree based on mitochondrial genome sequences and molecularly defined drug resistance profiles of 966 isolates showed four clusters."*

19. Discussion section starting at 250. I'm not so convinced that CVIET creates a "permissive genetic background" as much as there is geographical overlap in selective pressure.

Response 3.20: We thank the reviewer for this insightful comment and agree that the evidence around this argument is inconclusive. Previous studies support the hypothesis that some genetic backgrounds in Southeast Asia may have contributed to resistance (Miotto et al 2015). They found that certain positions, including the chloroquine resistance transporter, have been associated with resistance, but the overall role of these backgrounds is influenced by regional variations, including geographical factors and selective pressures. Additional research is needed to determine whether specific genetic backgrounds, such as CVIET, facilitate resistance development and to understand the mechanisms involved in the emergence and persistence of resistance, particularly in regions with different epidemiological and drug use patterns. Our hypothesis was based on the fact that we observed a significant co-occurrence of *Pf*K13 R622I variant and *Pf*CRT variants even after stratifying by *Pf*CRT variant frequency (low/medium/high categories), as mentioned in **response 3.13**. This co-occurrence remained significant in the low *Pf*CRT variant frequency group. Therefore, we conclude that this observation is not just a result of a high frequency of *Pf*CRT variants in the country. The significant co-occurrence of *Pf*K13 R622I variant and *Pf*CRT variants are less likely to be caused solely by an overlap in selective pressures. We have revised the manuscript to address the uncertainities in this regard, clarifying these points and to emphasize that the observed genetic patterns are likely influenced by multiple factors, including regional differences and existing genotype frequencies.

20. Again, I'm not convinced of distinct parasite clusters based purely on drug resistance gene sequences. More precise language is needed if you are referring to "molecularly defined resistance patterns" as opposed to identity by descent.

Response 3.21: As mentioned above (**Response 3.14**), the mitochondrial genome was included in this analysis as well. To clarify this to the readers we have now rephrased this as (line 255):

"Ancestry analysis using identity-by-descent (IBD) of the mitochondrial genome and drug resistance genes showed that…"

25 February 2026

Re: Manuscript NMICROBIOL-25041264.

Revised Title: "Genomic surveillance of Ethiopian *Plasmodium falciparum* reveals co-occurrence of *Pfcrt* and *Pfk13* mutations in vivax–falciparum co-endemic settings "

On behalf of my co-authors, I would like to thank you and the reviewers once again for the thoughtful and constructive feedback provided throughout the review process. The comments from both rounds have been invaluable in substantially improving the clarity, rigor, and balance of our manuscript.

In this revised version, we have carefully addressed all remaining concerns. The messaging has been refined to ensure greater nuance, and the ambitious interpretations noted in the earlier draft have been moderated in line with reviewer guidance and our further analysis. We believe these changes have strengthened the manuscript considerably, both in terms of analytical transparency and interpretive restraint. We have substantially reduced the word count as the changes have introduced extra word counts than the recommendations by the journal.

A detailed, point-by-point response to all comments is provided in the accompanying document, with responses highlighted in blue and line numbers referencing the clean revised manuscript.

This submission includes:

1. Revised manuscript with track changes
2. Clean revised manuscript
3. Extended data
4. Supplementary tables
5. Supplementary information

We hope the revisions satisfactorily address all outstanding concerns and demonstrate the substantial improvement of the work. We are grateful for the reviewers' and your guidance, which has helped us sharpen the manuscript's contribution, and we look forward to your consideration as the editorial process moves forward.

Kind regards,

Fitsum Girma Tadesse (on behalf of all co-authors)
Malaria and NTD Division, Armauer Hansen Research Institute
Jimma Road, ALERT-AHRI Health Village, POBOx 1005
Addis Ababa, Ethiopia
Email: fitsum.girma@ahri.gov.et / fitsum.tadesse@lshtm.ac.uk

Dear Fitsum,

Thank you for your patience while your manuscript "Distinct Genetic Histories, Convergent Drug Resistance in Ethiopian Plasmodium falciparum: Artemisinin and Chloroquine" was under peer-review at Nature Microbiology. It has now been seen by 3 referees, whose expertise and comments you will find at the end of this email. Although they find your work of some potential interest, they have raised a number of concerns that will need to be addressed before we can consider publication of the work in Nature Microbiology.

In particular, while referee #2 is now satisfied, referees #1 and #3 still have some important concerns. Referee #1 says that, as it stands, the data do not support the conclusion that there is a propensity for K13 mutations to occur in parasites with existing drug resistance mutations. The referee suggests to remove the concept of convergent evolution and provide a neutral reporting of the data. Editorially, we would encourage you to do this as otherwise, we will need you to perform more robust methodology, including modeling, for the subsetting analysis. Referee #3 says the title needs to be modified to better reflect the findings of this study and says they are concerned that exploration of gene-wide diversity and evolutionary relationships may still be confounded by selection rather than genetic relatedness. Editorially, we will need you to address the concerns from both referees in the manuscript and ensure that analyses are appropriate and reporting is in line with the data. I should note that this will be the last chance to revise the manuscript accordingly.

Should further experimental data and/or text changes allow you to address these criticisms, we would be happy to look at a revised manuscript.

Response: We are grateful to the editor and reviewers for their constructive feedback. In line with Referee #1's critique, we have revised the manuscript to ensure neutral and objective reporting of our findings, removing reference to convergent evolution. As recommended by Referee #3, we have modified the title to better reflect the scope and outcomes of the study. Furthermore, we have incorporated additional analyses and textual revisions to address concerns regarding gene-wide diversity and evolutionary relationships, ensuring that the data presentation and conclusions on population genetics are robust and appropriately supported.

We are committed to providing a fair and constructive peer-review process. Please do not hesitate to contact us if there are specific requests from the reviewers that you believe are technically impossible or unlikely to yield a meaningful outcome.

We strongly support public availability of data. Please place the data used in your paper into a public data repository, if one exists, or alternatively, present the data as Source Data or Supplementary Information. If data can only be shared on request, please explain why in your Data Availability Statement, and also in the correspondence with your editor. For some data types, deposition in a public repository is mandatory - more information on our data deposition policies and available repositories can be found at https://www.nature.com/nature-research/editorial-policies/reporting-standards#availability-of-data.

Response: Raw FASTQ files with sequence data have been made available on the European Nucleotide Archives (ENA, project accession number PRJEB87415). Sample names, accession numbers, and metadata are listed in Supplementary Table 8.

Please include a data availability statement as a separate section after Methods but before references, under the heading "Data Availability". This section should inform readers about the availability of the data used to support the conclusions of your study. This information includes

accession codes to public repositories (data banks for protein, DNA or RNA sequences, microarray, proteomics data etc…), references to source data published alongside the paper, unique identifiers such as URLs to data repository entries, or data set DOIs, and any other statement about data availability. At a minimum, you should include the following statement: "The data that support the findings of this study are available from the corresponding author upon request", mentioning any restrictions on availability. If DOIs are provided, we also strongly encourage including these in the Reference list (authors, title, publisher (repository name), identifier, year). For more guidance on how to write this section please see:
http://www.nature.com/authors/policies/data/data-availability-statements-data-citations.pdf

Response: A data availability statement is included as a separate section after the Methods section and reads:

"Data and code availability statement
Raw sequence data for the 604 sequenced isolates in this study can be found in the European Nucleotide Archives (ENA), project accession number PRJEB87415. Sample names, accession numbers, and metadata are listed in ***Supplementary Table 8.*** *The custom scripts used for bioinformatics analysis can be found at https://github.com/AHRI-Malaria/EMAGEN. Code for the IBD analysis can be found at https://github.com/LSHTMPathogenSeqLab/malaria-hub."*

When revising your manuscript:

* Include a "Response to referees" document detailing, point-by-point, how you addressed each referee comment. If no action was taken to address a point, you must provide a compelling argument. This response will be sent back to the referees along with the revised manuscript.

Response: A point-by-point response to reviewers' comments is included below.

* If you have not done so already we suggest that you begin to revise your manuscript so that it conforms to our Article format instructions at http://www.nature.com/nmicrobiol/info/final-submission. Refer also to any guidelines provided in this letter.

Response: We have reviewed the Article format instructions and believe our manuscript aligns with these guidelines. Kindly let us know if this is not the case.

* Include a revised version of any required reporting checklist. It will be available to referees (and, potentially, statisticians) to aid in their evaluation if the manuscript goes back for peer review. A revised checklist is essential for re-review of the paper.

Response: We have revised the reporting checklists where applicable and have submitted these alongside our revisions.

When submitting the revised version of your manuscript, please pay close attention to our href="https://www.nature.com/nature-portfolio/editorial-policies/image-integrity">Digital Image Integrity Guidelines. and to the following points below:
-- that unprocessed scans are clearly labelled and match the gels and western blots presented in figures.
-- that control panels for gels and western blots are appropriately described as loading on sample processing controls
-- all images in the paper are checked for duplication of panels and for splicing of gel lanes.

Response: We have reviewed the Digital Image Integrity Guidelines and believe our figures conform with these guidelines. Kindly inform us if this is not the case.

EXTENDED DATA FIGURES

When re-submitting your manuscript, please ensure that any supplementary figures and tables that are crucial to the manuscript's conclusions are converted into Extended Data figures and tables to increase visibility of these data. Extended Data figures and tables are online-only (present in the online PDF and full-text HTML versions of the paper), peer-reviewed display items that provide essential background to the article but are not included in the main article due to space constraints. A maximum of ten Extended Data display items (figures and tables) is permitted.

Response: We have reviewed our Supplementary and Extended Data figures and tables and believe that all items that are crucial to the manuscript's conclusions are included as Extended Data figures and tables.

Finally, please ensure that you retain unprocessed data and metadata files after publication, ideally archiving data in perpetuity, as these may be requested during the peer review and production process or after publication if any issues arise.

Nature Microbiology is committed to improving transparency in authorship. As part of our efforts in this direction, we are now requesting that all authors identified as 'corresponding author' on published papers create and link their Open Researcher and Contributor Identifier (ORCID) with their account on the Manuscript Tracking System (MTS), prior to acceptance. This applies to primary research papers only. ORCID helps the scientific community achieve unambiguous attribution of all scholarly contributions. You can create and link your ORCID from the home page of the MTS by clicking on 'Modify my Springer Natureaccount'. For more information please visit please visit www.springernature.com/orcid.

If you wish to submit a suitably revised manuscript we would hope to receive it within 2 months. If you cannot send it within this time, please let us know.

In the meantime we hope that you find our referees' comments helpful.

Yours sincerely,

****

Reviewer Expertise:

Referee #1: P. falciparum resistance
Referee #2: P. falciparum resistance, genomics, drug discovery
Referee #3: P. falciparum genomics, evolution of antimalarial drug resistance

Reviewer Comments:

Reviewer #1 (Remarks to the Author):

The author's response to comments was extensive- almost as long as the manuscript. The changes in the manuscript were more modest but did included new and revised analysis of the data which clarified some points raised in the review. Most of the changes were modifications that put into perspective some of the previous over-reaching conclusions.
The major point which remains unresolved is the question of the relationship of existing chloroquine resistance with the emergence of K13 mutations associated with emerging resistance to artemisinin. This was a major point raised in the original critique. The authors decided to subset the data based on geographic distribution and found that this association only existed in one region. They then subset the data on transmission intensity and separately by PfCRT allele frequency. The analysis showed that population structure impacted the conclusion of an association with K13 emergence and other drug resistant genotypes. Instead of recognizing this point, the authors conclude that this association only occurs in certain unique settings. They fail to acknowledge the interrelatedness of API, geography and PfCRT distribution and fail to control for these confounders in their analysis. Therefore, their conclusion is not valid. Should the authors want to directly address this issue they need a more robust approach including modeling. As it stands, the data do not support the conclusion that there is a propensity for K13 mutations to occur in parasites with existing drug resistance mutations. They continue to make this a major conclusion of the paper and have retained this concept in the title.
Response: We thank the reviewer for the detailed follow-up and for emphasizing the critical issue of disentangling the relationship between pre-existing chloroquine resistance and the emergence of *Pfk13* mutations. We fully acknowledge the interrelatedness of geography, transmission intensity, and background allele frequencies, and agree that stratified analyses alone cannot resolve these confounding effects. To address this concern more directly, we undertook additional analyses restricted to Ethiopian districts with confirmed circulation of the *Pf*K*13* R622I variant (9 sites; 302 samples, of which 297 had complete genotyping data). Our objective was to evaluate genetic and ecological determinants of R622I occurrence while explicitly accounting for confounding by site-level factors as the reviewer correctly pointed out. To do this, we applied mixed-effects logistic regression with a random intercept for site, jointly adjusting for standardized API and *P. vivax* proportion, to test whether associations persist after ecological control. As a sensitivity analysis, we further implemented Bayesian multilevel logistic regression with a random intercept for site using weakly informative priors, reporting posterior odds ratios and credible intervals to assess robustness under sparse outcome events.

Across nine Ethiopian districts with confirmed *Pfk13* R622I circulation, 32 of 297 samples (10.8%) carried the mutation. When ecological confounders were incorporated in mixed-effects models, these associations persisted: R622I remained linked to *Pf*CRT CV**IET** (aOR 3.19, 95% CI 1.60–6.34) and *Pf*DHFR A**I**C**N**I (aOR 5.16, 95% CI 1.60–16.70). Bayesian multilevel sensitivity analyses confirmed the robustness of these findings, with posterior odds ratios consistent in direction and magnitude. Associations with *Pf*CRT CV**IET** and *Pf*DHFR A**I**C**N**I remained strong and ecological predictors were more uncertain, reflecting sparse outcome events and site-level correlation.

These approaches were designed to move beyond stratification and provide preliminary evidence on whether associations between *Pfk13* R622I and background resistance markers remain after controlling for geography and transmission ecology. We emphasize that these analyses are exploratory and intended to clarify the limitations of current data, while pointing toward more robust modelling frameworks for future work. While context-specific signals and sensitivity to population structure underscore that these associations are not universally generalizable, the consistency across

frequentist and Bayesian models strengthens confidence in the robustness of the observed associations. In particular, the independent and reproducible links of R622I with *Pf*CRT CV**IET** and *Pf*DHFR A**I**C**N**I highlight strong genetic associations that merit further investigation. We note that fully disentangling these relationships will require more comprehensive datasets, including whole-genome sequencing and longitudinal genomic surveillance, to establish causality and inform policy-relevant conclusions.

We have presented these findings in the results part of the revised version of the manuscript alongside the stratification analysis (line 206-220). The additional analysis is now presented in table (Extended Data 9), which is presented here.

Extended Data 9. Association of *Pfk13* R622I with background resistance markers and site-level ecology

Marker / Variable	cOR[a] (95% CI)	aOR (95% CI)	P value	Bayesian cOR[a] (95% CI)	Bayesian aOR[b] (95% CrI)	Posterior Probability[†]
*Pf*CRT CVIET	5.66 (1.90–16.89) *	3.19 (1.60–6.34)	0.001	6.24 (2.27–18.75)	3.49 (1.26–10.77)	99.35
*Pf*DHFR AICNI	6.68 (1.76–25.37) *	5.16 (1.60–16.70)	0.006	7.12 (2.59–21.59)	5.45 (1.82–17.45)	99.94
*Pf*DHPS ISGEAA	5.55 (1.33–23.24) *	2.63 (0.88–7.88)	0.084	6.16 (2.08–20.80)	2.92 (0.91–10.85)	96.44
P. vivax proportion (standardized)[‡]	1.68 (0.97–2.91)	1.30 (0.75–2.27)	0.348		1.34 (0.61–3.15)	79.17
API (standardized)[‡]	1.75 (0.81–3.76)	1.80 (1.04–3.10)	0.036		1.80 (0.78–4.29)	93.01
Site-level variance (σ^2)	-	0.17 (0.004–7.21)	-	-	1.02 (0.02–5.16)	-

[a] Crude odds ratio (cOR)
[b] Adjusted odds ratio (aOR)
[*]Statistically significant
[†] Posterior probabilities were derived from the multivariable Bayesian mixed-effects logistic regression model with a random intercept for site. Values represent the probability that the regression coefficient is greater than zero (OR > 1) on the log-odds scale.
[‡]Models result included the genetic marker plus standardized API and *P. vivax* proportion, with a random intercept for site. Bayesian models used weakly informative Normal (0,2.5) priors on fixed effects (log-odds scale).

We have revised the discussion part of the revised manuscript to ensure that these results are presented descriptively rather than as evidence of a biological propensity for *Pfk13* mutations to arise on resistant background (lines 285-293; 314-321). Specifically, we have:

- Removed the convergent co-occurrence concept from the title and abstract, ensuring that it is no longer presented as a major finding of the study. The manuscript title has now been revised to *"***Genomic surveillance of Ethiopian *Plasmodium falciparum* reveals co-occurrence of *Pfcrt* and *Pfk13* mutations in vivax–falciparum co-endemic settings".**
- Removed any strong interpretation regarding a propensity for *Pfk*13 mutations to arise on chloroquine-resistant or multidrug-resistant backgrounds.

- Reframed the results descriptively, noting that while certain associations appear in specific geographical or epidemiological contexts, these signals are highly sensitive to population structure and do not support a generalizable conclusion.
- Added explicit text to the Discussion acknowledging that disentangling these relationships requires more robust inferential methods and richer datasets (whole-genome sequencing, diversity markers, and temporal genomic data).
- Clarified that our current dataset cannot determine whether the observed patterns reflect true biological interactions or are driven by unmeasured structure or sampling variation.

We believe these revisions directly address the reviewer's concern and strengthen the clarity and rigor of the manuscript by aligning conclusions with the scope of the available data.

Reviewer #2 (Remarks to the Author):

Thank you for clearly addressing my comments in so much detail. All previous issues have been corrected and I would support the publication of this study.

Response: Thank you

Reviewer #2 (Remarks on code availability):

The code files are improved and appear to provide adequate information.

Response: Thank you

Reviewer #3 (Remarks to the Author):

I really appreciate how much effort the authors put into addressing my concerns. I feel the paper is much clearer and more directly addresses what I see as one of its most interesting points: what happens in terms of drug resistance when AL and CQ are both used in the same setting. This mirrors one the proposed policy changes that stakeholders hope will prolong ACT efficacy -- using multiple first line therapies -- and this provides useful data to help model expectations.
I think this is an interesting, informative paper, but I still have a few concerns/suggestions.

I think both the current title and the proposed revised title are confusing and unclear
Response: Thank you. We revised the title of the manuscript to reflect the major findings. *"***Genomic surveillance of Ethiopian *Plasmodium falciparum* reveals co-occurrence of *Pfcrt* and *Pfk13* mutations in vivax-falciparum co-endemic settings".**

1. lines 137-144. I continue to be concerned that exploration of gene-wide diversity and evolutionary relationships may still be confounded by selection rather than genetic relatedness. Is there additional variation in synonymous SNPs? Could the analyses be repeated with these more neutral markers? In fact, all minimal-spanning haplotype networks would benefit from use of microhaplotypes including all SNPs, not just NS SNPs.
Response: We thank the reviewer for raising this important point. We clarify that the minimal-spanning networks in this manuscript already included synonymous SNPs (described in the methods; lines 457-464), the figure legend incorrectly stated otherwise (**Extended data 6**). This oversight has now been corrected. To further support transparency, we have provided a list of synonymous SNPs and their district-level frequencies in **supplementary table 4.** Minor differences between the networks and the table reflect differences in filtering criteria: the table includes only variants detected by at least two callers and present in at least 10 samples, whereas the minimal-spanning networks excluded only variants observed in fewer than 2 samples. We have also re-checked the network annotations and corrected duplications.

Finally, we agree with the reviewer that gene-wide diversity may be confounded by selection, and we have made this limitation more explicit (lines 317-321) in the revised manuscript (see responses to related comments below). We also acknowledge the reviewer's suggestion that microhaplotypes incorporating all SNPs could provide a more neutral framework for future analyses. While our current dataset does not allow us to fully implement this approach, we recognize its value and will consider it in subsequent work. We believe these corrections and clarifications strengthen the manuscript and improve the accuracy of our analyses.

2. I think it would be worth mentioning the lack of significant correlations between prevalence of validated K13 mutations and API and Pv proportion in the main text (Sup table 4). This provides information on how multiple first line therapy may influence the success of ART-R. It doesn't seem to help here, but still may be protective of the partner drug. Sorry for my obsession with this topic.
Response: We thank the reviewer for this thoughtful observation and for highlighting the importance of considering ecological correlates in the broader context of artemisinin and partner drug resistance. As noted, our site-level correlation analysis (presented in the previous version) did not identify significant associations between *Pfk13* mutation prevalence and either API or *P. vivax* proportion (**Supplementary Table 5**).
In response to the earlier concern regarding confounding by Reviewer #1, we extended our analyses beyond univariate site-level correlations to individual-level multivariate models. These revealed associations with ecological predictors were uncertain using Bayesian modelling (lines 440-454 methods). The results of these models are now incorporated into the main text (lines 213-220 results). We agree that these results underscore the complexity of disentangling ecological and genetic

determinants of resistance, and we have emphasized this point in the revised discussion (lines 286-294).

3. For the population structure shown in the PCA analysis: I had voiced concerns that the population structure may not be reflective of IBD because it was driven by drug resistance genes, which are more likely to reflect selective pressure than genetic relatedness. The authors addressed this concern by indicating that mitochondrial sequences were also used. However, it is also reported that variation in the mitochondrial genome was limited. What does the structure look like when just considering the mitochondrial genome? Is there sufficient variation to define populations? Also, were synonymous SNPs used in this analysis or just NS SNPs as was done for minimal-spanning networks. I couldn't quite tell from the methods section. If not, I think it would be a good idea to include non-coding polymorphisms to reduce the impact of convergent evolution resulting from drug selection, providing a more accurate inference of relatedness.
Response: We thank the reviewer for this insightful comment. PCA and IBD analyses included both synonymous and missense SNPs. This has now been clarified in the methods section (lines 462-463). As noted, variation in the mitochondrial genome was limited. For completeness, we conducted PCA using mitochondrial genome only (see **Extended Data 10**). Due to the low level of variation, clustering was minimal and did not show consistent association with geography or *P. vivax* endemicity. This confirms that the broader population structure observed is primarily driven by drug resistance loci under selective pressure rather than neutral genetic relatedness.

We have revised the relevant text to clarify this point and to emphasize that the observed patterns reflect molecular resistance profiles shaped by selection (lines 224-237). In addition, Figure 4 has been updated after reprocessing with GATK v4.1.4.1 (lines 461-463) to ensure accurate handling of missing genotype calls. The previous version (4.5.0.0) applied a default parameter that imputed missing calls as reference alleles, which could artificially reduce apparent genetic variation. The methods section has been corrected accordingly.

4. Line 315. The emergence of different K13 mutations in different regions is not limited to SEA. The same is true of Uganda. May be worth mentioning to give additional African context.
Response: Thank you. A mention of this with a reference has now been added to this sentence (line 278-279).

5. Section starting at 320. Does there have to epistatic interaction? Could this just be explained by the fact that parasites need to survive exposure to DHA, lumefantrine and chloroquine, so it's good to have resistance to all these drugs when possible? The fitness costs of having resistance to multiple drugs is likely to vary by likelihood of exposure to AL and CQ, by competition with other parasites or evading immune responses in higher transmission areas.
Response: We thank the reviewer for this important point. We agree that the co-occurrence of resistance markers does not necessarily imply epistatic interaction. While epistasis has been suggested in prior studies, our data are not sufficient to establish this. We have therefore revised the discussion to present epistasis as one possible explanation, but emphasize alternative causes such as overlapping drug pressures, variation in transmission intensity, and parasite population structure to provide a nuanced discussion of our findings (lines 286-294). This framing better reflects the complexity of selective environments in Ethiopia and aligns with the reviewer's suggestion.

6. Line 345: The N86 allele is associated with decreased susceptibility to lumefantrine an increased susceptibility to chloroquine, not reduced susceptibility to both drugs.
Response: Thank you for rectifying this. We have now corrected this sentence, and a reference has been added (lines 300-306).

7. Line 362. I think it would be wise to acknowledge the limitations of the IBD analysis as it is done primarily with variation under selective pressure. Including all SNPs

Response: Thank you. We acknowledged the limitations in this section (lines 310-312).

8. Line 368. I think it would be more appropriate to say “and there is limited evidence for lumefantrine failure in Africa” rather than no reported failures. A recent TES showed <90% AL efficacy at 2 sites in Uganda (Kamya 2025 Lancet ID) and there have been reports of AL treatment failures when travelers returned to their home countries after being in Africa.

Response: Thank you. This sentence has been edited to the proposed phrasing, and the reference has been included (lines 59-63).

CRT I356T is associated with piperaquine resistance in SEA, where dihydroartemisinin-piperaquine was widely used.

Response: Due to word count limits, this discussion was excluded, partly because DP has not been used in Ethiopia, it was introduced in 2025 as a second line treatment. Future studies will benefit from longitudinal monitoring in areas where it is being implemented. In line with this it was detected rarely (1.7%, 9/524).
